# A Neural-Network-Optimized Hydrogen Peroxide Pairwise Additive Model for Classical Simulations

**DOI:** 10.1021/acs.jctc.3c00287

**Published:** 2023-06-12

**Authors:** Alvaro Ramos Peralta, Gerardo Odriozola

**Affiliations:** Área de Física de Procesos Irreversibles, División de Ciencias Básicas e Ingeniería, Universidad Autónoma Metropolitana-Azcapotzalco, Av. San Pablo 180, 02200 Ciudad de México, Mexico

## Abstract

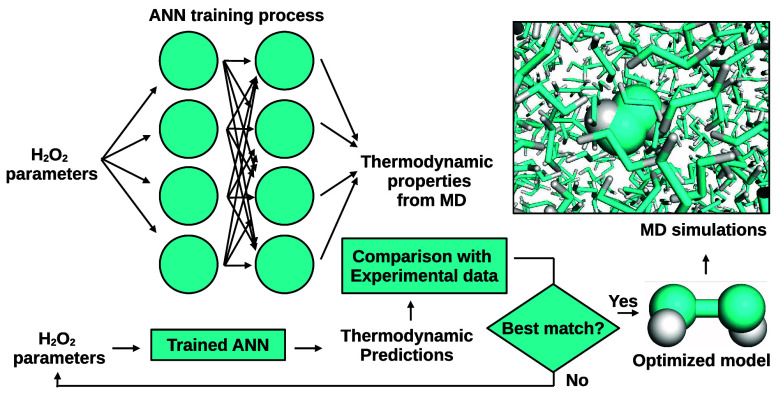

We have developed an all-atom pairwise additive model for hydrogen peroxide using an optimization procedure based on artificial neural networks (ANNs). The model is based on experimental molecular geometry and includes a dihedral potential that hinders the *cis*-type configuration and allows for crossing the *trans* one, defined between the planes that have the two oxygen atoms and each hydrogen. The model’s parametrization is achieved by training simple ANNs to minimize a target function that measures the differences between various thermodynamic and transport properties and the corresponding experimental values. Finally, we evaluated a range of properties for the optimized model and its mixtures with SPC/E water, including bulk-liquid properties (density, thermal expansion coefficient, adiabatic compressibility, etc.) and properties of systems at equilibrium (vapor and liquid density, vapor pressure and composition, surface tension, etc.). Overall, we obtained good agreement with experimental data.

## Introduction

Hydrogen peroxide^1^ is the simplest of the peroxides and the smallest molecule that exhibits enantiomeric isomerism, making it a chiral compound.^2^ Its pure liquid state is denser than water but only slightly more viscous. Like water, it forms hydrogen bonds, which give it a wide temperature window for the liquid state. In fact, the difference between its boiling point, 423.3 K, and melting point, 272.72 K,^3^ exceeds that of water, and its vapor pressure is indeed lower, 5 mmHg at 303 K.^4^ Given its chemical similarity to water, it is not surprising that the liquids are miscible for all concentrations and temperatures. It is worth mentioning that it produces a eutectic mixture with water showing a strong dependent melting point with composition.^4^

There are several uses for hydrogen peroxide in daily life and in industry. When diluted in water, it is used as a bleaching agent, including in hair bleach, and as an antiseptic.^5^ It is commonly used at home to disinfect sores and is part of some products to whiten teeth.^6^ In industry, it is often used at higher concentrations, and most notably, it can be used as a rocket propellant.^7^ These applications are based on the fact that peroxide is unstable and an oxidizing agent.

**Figure 1:**
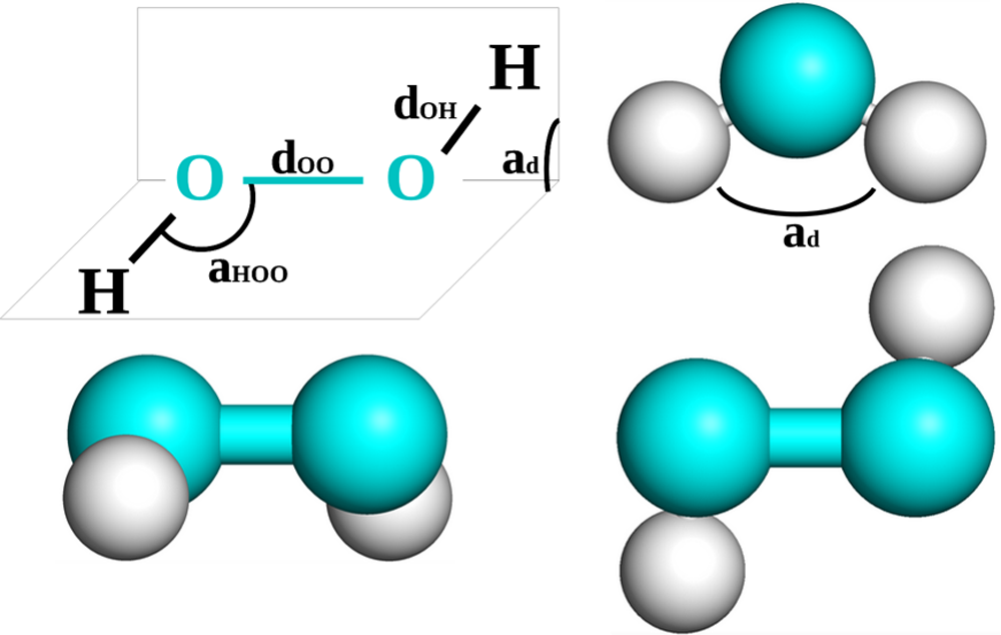
(Top left) Scheme showing the geometrical parameters of the H_2_O_2_ model. Side (bottom left), top (bottom right), and front (top right) views of the ball-and-stick representation of the model. The labels *d*_OO_ and *d*_OH_ correspond to the oxygen–oxygen and oxygen–hydrogen distances, respectively. The *a*_HOO_ and *a*_d_ labels refer to the hydrogen–oxygen–oxygen and dihedral angles, respectively.

Its molecular structure has two oxygen atoms joined by a single bond, each bonded to its corresponding hydrogen atom (see Figure 1). Thus, its chemical formula is H_2_O_2_. The molecule is not planar and at its configuration of minimum energy shows a certain dihedral angle, defined by the planes containing the oxygen atoms and each hydrogen, close to 110°. This configuration is compatible with two enantiomers, structures that are mirror images of each other, which can be transformed one into the other by crossing a small potential barrier associated with the *trans* (planar) configuration.^8,9^ In principle, the enantiomers are also connected through the *cis* (also planar) configuration, but the height of the energetic barrier is much larger through this path.^8,9^

Although interesting and small, the hydrogen peroxide molecule has not been extensively studied by simulation techniques. There are works that take into account its quantum character^10−14^ and few that make use of classical simulations.^15−18^ In the latter lists, the classical models given by Chung^16^ were employed to compute residence times in crowded protein environments and diffusion on the surface of a protein. As they claim, classical models have not been fully tested against thermodynamic data in water solutions, and partial charges placed on hydrogen sites range from 0.36 up to 0.54 elementary charges.^11,19−21^ From the possible models, we would like to highlight the one recently given by Orabi and English.^18^ The model is based on the CHARMM force field,^22,23^ where the geometrical parameters are taken from an ab initio geometry optimization of the H_2_O_2_ single molecule, the partial charges are adjusted to match the dipole moment and energy as a function of the dihedral angle (also from ab initio computations), the hydrogen Lennard-Jones (LJ) parameters correspond to the TIP3P model^24^ (CHARMM force field^22,23^), and the oxygen LJ parameters are fitted to reproduce the density and heat of vaporization at 0 °C. That is, the authors of this model followed a well-accepted and popular path to yield their H_2_O_2_ model (there are other ways such as fitting the relative dielectric constant and the temperature of maximum density,^25^ for instance). However, to our knowledge, there are still no simulations providing thermodynamic data such as boiling point, vapor pressure, surface tension, critical point, and viscosity, among other important properties for pure peroxide and water solutions.

In this study, we propose an alternative model for H_2_O_2_ based on the OPLS-aa force field^26−28^ to address the lack of an intensively tested H_2_O_2_ model. Note that, unlike the CHARMM force field, the OPLS-aa does not include LJ parameters for the H sites, simplifying the model. We parametrize the pairwise additive model using artificial neural networks (ANNs) trained on corresponding sets of molecular dynamics results. To do so, we define ranges for the model’s geometrical structure, including bond distances and angles, LJ parameters of the oxygen atoms, partial charges, and the energy contribution associated with the H_2_O_2_ dihedral angle. We use the molecular geometry of the solid and vapor phases as a basis, but, in contrast to the method used by Orabi and English, we sweep all possible parameter values into ranges to minimize differences between the ANNs’ predictions and experimental data. Our approach aims to optimize the parameters by considering several properties of pure H_2_O_2_ and water mixtures simultaneously, targeting 22 different values. Notably, ANNs have recently been employed for optimization purposes of pairwise additive molecular models,^29−32^ extracting forces and dynamic laws from active-matter trajectories^33,34^ and even serving as complex potential functions for molecular simulations.^35−41^ We present several liquid and vapor properties of the obtained pairwise additive model as a function of the weight percentage of the water–peroxide mixture and temperature.

## Model

**Figure 2:**
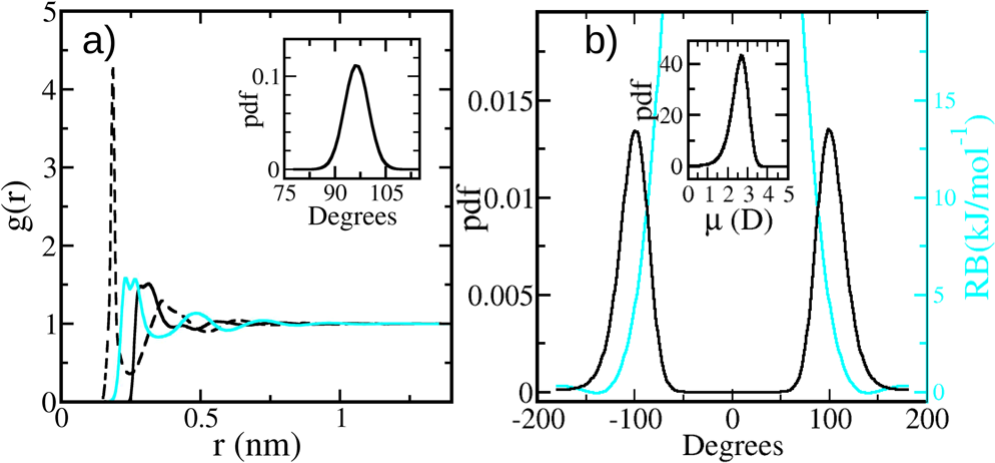
(a) Radial distribution function, *g*(*r*), for the O–O (solid black line), O–H (black dashed line), and H–H (solid cyan line) pairs (note that the peaks corresponding to the internal O–O and O–H bonds are not considered to gain clarity). The inset shows the *a*_HOO_ probability distribution function, pdf. (b) Probability distribution function, pdf, for the dihedral angle *a*_d_ (black) and the Ryckaert-Bellemans potential contribution (cyan line and right *y*-axis). The inset depicts the probability distribution function of H_2_O_2_ dipole moments. All data correspond to a bulk liquid of pure H_2_O_2_, *T* = 293 K, and *P* = 1 bar.

The geometrical parameters necessary to define the model are the oxygen–oxygen distance, *d*_OO_, the oxygen–hydrogen distance, *d*_OH_, the angle formed between the lines joining the oxygen–hydrogen and oxygen–oxygen sites, *a*_HOO_, and the dihedral angle given by the position of all atoms, *a*_d_. These parameters and the corresponding force constants define the so-called bonded interactions. We use the OPLS-aa force field^26−28^ potential functions, including a Ryckaert-Bellemans (RB) dihedral contribution. Thus, we employ harmonic potentials for bonds and angles and the dihedral function , with the constraints *c*_0_ = 0.312*c*_2_, *c*_1_ = −*c*_2_, *c*_3_ = 0.300*c*_2_, and *c*_4_ = *c*_5_ = 0. With these constraints and for *c*_2_ ranging from 25.5 kJ mol^–1^ to 36.0 kJ mol^–1^, we get a function with minima at *a*_d_ ≈ ±130°, a large maximum at *a*_d_ = 0°, and a tiny barrier at *a*_d_ = ± 180° (see the cyan line inside panel b of Figure 2). Note that the large and tiny barriers correspond to the *cis* and *trans* configurations, respectively. *c*_2_ controls the height of the large barrier, which translates into the position of the *a*_d_ peaks. Thus, the selected range of *c*_2_ is such that the *a*_d_ peaks are located close to the experimental values for the solid and vapor phases.^8,42^ The so-called nonbonded interactions are given by the LJ potential acting only on the oxygen sites, with parameters ϵ_OO_ and σ_OO_, and the Coulomb potential acting on every site. We are excluding all internal LJ contributions to the model. The values of the partial charges located at hydrogen sites, *q*_H_, and oxygen sites, −*q*_H_, define the intra and intermolecule Coulomb interactions.

The geometrical parameters corresponding to the solid^42^ and vapor^8^ phases are 0.1453 nm, 0.0988 nm, 101.9°, 90.2°, and 0.1474 nm, 0.0970 nm, 94.8°, 115.5°, corresponding to *d*_OO_, *d*_OH_, *a*_HOO_, and *a*_d_, respectively. Thus, we are setting the ranges for the geometrical parameters in 0.145–0.148 nm for *d*_OO_, 0.094–0.099 nm for *d*_OH_, and 94–102° for *a*_HOO_. This way, we cover the solid–vapor experimental range while still allowing for some flexibility in the parameters. For the case of *a*_d_, as mentioned in the previous paragraph, the selected range of *c*_2_ combined with the other parameters yields *a*_d_ values inside the experimental range. In addition, the *q*_H_ range found in the literature is very large,^11,19−21^ and we need to narrow it down. We have noticed that the Atomic Charge Calculator II,^43^ using empirical methods along with parameters from literature, leads to values of *q*_H_ between 2 and 10% larger for H_2_O_2_ than for H_2_O when employing the same method. Also, Martins-Costa and Ruiz-López yields similar results.^11^ In addition, three-point water models such as SPC,^44^ SPC/E,^45^ and TIP3P^24^ have *q*_H_ values in the 0.41–0.4238 e range. Hence, we find it reasonable to set the *q*_H_ range in 0.43–0.47 e. We have also noticed that this range yields relatively good results when combined with the other parameter ranges. Finally, we observe that larger partial charges produce very large viscosities. We set the σ_OO_ range in 0.296–0.300 nm to get densities close to the experimental ones, since the impact of σ_OO_ is strong on this property. The ϵ_OO_ range in 0.7–0.9 kJ mol^–1^ is set by taking into account other types of oxygen atoms of the OPLS-aa force field.^26−28^ Finally, we set the single bond O–O force constant equal to that of the N–O single bond of the OPLS-aa force field and the H–O force constant equal to the one of the TIP4F^46^ water model. It should be noted that there is no O–O single bond defined in the OPLS-aa force field, but the C–O and N–O bonds have the same force constant values due to the similarity in size and electronegativity between C and N. As O is also similar to N and C in size and electronegativity, we expect the O–O bond to have a similar strength.

The model *itp* file, defining the optimized parameters and the force constants, is included in the first section of the Supporting Information (SI). The optimized parameters are *d*_OO_ = 0.1463 nm, *d*_OH_ = 0.0979 nm, *a*_HOO_ = 95.79°, *q*_H_ = 0.4323 e, σ_OO_ = 0.29989 nm, ϵ_OO_ = 0.8912 kJ mol^–1^, and *c*_2_ = 26.91 kJ mol^–1^. In the following sections, we describe the route we followed to reach these optimized values.

### Simulations Details

We employ the gromacs 2022.4 molecular dynamics package^47−50^ to produce and analyze trajectories of H_2_O_2_–H_2_O mixtures. In all cases, we use the SPC/E rigid water model.^45^ This model has a good performance considering it has only three sites.^51,52^ The weight percentage of H_2_O_2_, wt %, in the solutions is 0, 10, 20, 30, 40, 60, 80, and 100. The number of H_2_O_2_ and H_2_O molecules for each box is given in such a way as to yield similar cell sizes at room conditions and a minimum of 6000 oxygen atoms. Hence, we use 6000 H_2_O molecules for 0 wt % and 4350 molecules for 100 wt %. Note that the number of oxygen atoms increases with wt % due to the higher density of H_2_O_2_. Before conducting the formal runs described below, we built the system and performed the necessary tasks to achieve thermalized initial configurations.

To access densities, ρ, the relative dielectric constant, ϵ, diffusion coefficients, *D*, enthalpy of vapor formation, Δ*H*_vap_, coefficient of thermal expansion, α_P_, isothermal and adiabatic compressibilities, κ_T_ and κ_S_, number of hydrogen bonds, *n*_h_ and structural properties, we produce 100 ns trajectories with 0.5 fs time steps for bulk-liquid systems. For properties derived from fluctuations,^53^ we carry out a block-averaging error analysis and discard those blocks far (more than three standard deviations apart) from the average. We discarded only three blocks in all runs for the determination of ϵ, all placed at their beginning (the thermalization length was probably not enough in these cases). The trajectories are written with a frequency of one each of 1000 steps. For this purpose, we set the v-rescale algorithm with a 0.1 ps coupling time for temperature coupling and the c-rescale isotropic algorithm with a 1.0 ps coupling time for pressure coupling. We set a 1.2 nm cutoff length for nonbonded interactions, considered dispersion corrections for energy and pressure, and used a Fourier spacing of 0.12 nm to handle the long-range Coulomb interactions with the PME method. The properties are obtained by using the *energy*, *dipoles*, *msd*, *hbond*, and *rdf* gromacs tools with the proper flags. These relatively large runs are needed to avoid large uncertainties for the relative dielectric constant.

Shear viscosities, μ, are accessed through independent 50 ns trajectories, setting the amplitude of the acceleration profile to 0.05 nm ps^–2^ (nonequilibrium dynamics) and turning off the pressure coupling.^54^ For these runs, we have also turned on the constraints to *h-angles*, meaning that *d*_OH_ and *a*_HOO_ get fixed but not the dihedral angle nor the *d*_OO_ distance. By doing so, we get results much closer to the experimental values reported by Phibbs and Giguère^55^ (around 30% smaller). For all the explored values of the parameters, the model most frequently produces viscosity values above the experimental ones^55^ for *T* = 293 K.

Finally, we access the surface tension, λ, vapor pressure, *P*_v_, vapor composition, wt %_v_, boiling temperature, *T*_b_, critical point temperature, *T*_c_, pressure, *P*_c_, and density, ρ_*c*_, by setting 1 × 1 × 3 aspect ratio prismatic simulation cells containing a liquid slab in equilibrium with its corresponding vapor phase.^56,57^ In this case, we sampled from the NVT ensemble, turned off the energy and pressure dispersion corrections (the system is not homogeneous), increased the cutoff distance to 1.6 nm (to partially compensate for turning off the dispersion corrections), and set a number of Fourier wavevectors along the largest side of the prismatic cell 3 times larger than those of the other two sides. In addition to these details, the system size must be sufficiently large to avoid spurious effects^58^ (the minimum of 6000 oxygen atoms is well-above the limit where these undesired effects appear).

The above-given paragraphs describe the simulation details to produce the formal runs for the final results of the model. However, since we need a large number of determinations to carry out the parametrization, we performed much shorter runs (20 ns with a time step of 1.0 fs) with smaller system sizes for this purpose. To study bulk-liquid systems, we consider 1424 peroxide and 1152 water molecules to yield a 70 wt % peroxide–water mixture and 2000 peroxide molecules for pureperoxide. To access the liquid–vapor properties, we set 2136 peroxide and 1728 water molecules and runs of only 10 ns. From these simulations, we get ρ, ϵ, μ, *P*_v_, λ, and wt %_v_, for pure and wt % = 70 mixtures at different temperatures and as a function of the varying parameters to train and validate the ANNs (see Table S1 and S2 of the SI).

### Model Parametrization

We produced 1000 determinations (Table S1 of the SI) of the target properties to train the ANNs. We normalized the input and output vectors (composed of 7 and 22 items each) to [0,1] and employed the backpropagation algorithm. The target properties correspond to pure H_2_O_2_ and 70 wt % of water–peroxide mixtures. We considered the evaluation of ρ, ϵ, μ, *P*_v_, λ, and wt %_v_. The last property applies only with the 70 wt % mixture. The corresponding experimental data and their sources are given in Table S1 at the SI.

Once trained, the predictions of the ANNs are compared with another set of 300 determinations of the target properties to evaluate their behavior. These 300 determinations (Table S2 of the SI) are obtained following the same procedure as for Table S1. We tried sequential ANNs having from 1 to 3 layers, with 2, 4, 8, and 16 neurons per layer, *N*_L_. We observed that further increasing *N*_L_ worsens the results. Also, we tried ReLu and Sigmoid functions following the linear steps to introduce nonlinearity. We use the PyTorch framework^59^ to implement the ANNs. Table S3 of the SI shows the deviations of their predictions from Table S2. Arguably, the best results are observed for ReLu/1/8, ReLu/1/16, ReLu/2/4, ReLu/2/8, Sigm/1/8, and Sigm/2/4 ANNs, where we follow the notation nonlinear-function/number of layers/*N*_L_. Note that a relatively small number of neurons can capture the general behavior of the data, something not easily observed by the naked eye. We believe that the relatively large dispersion of the simulation data, due to the lack of sufficient statistics (short runs), is smoothed out by the ANN training process. Note that the number of parameters, *N*_P_, increases with *N*_L_ and the number of layers. Thus, a large number of neurons tend to capture not only the genuine trend but also the noise of the training set, which negatively impacts its predictions.

As mentioned, the seven input parameters, **p**, sweep the following data ranges: 25.5 kJ mol^–1^ ≤ *c*_2_ ≤ 36.0 kJ mol^–1^, 0.43 e ≤ *q*_H_ ≤ 0.47 e, 0.296 nm ≤ σ_OO_ ≤ 0.300 nm, 0.7 kJ mol^–1^ ≤ ϵ_OO_ ≤ 0.9 kJ mol^–1^, 0.145 nm ≤ *d*_OO_ ≤ 0.148 nm, 0.094 nm ≤ *d*_OH_ ≤ 0.099 nm, and 94° ≤ *a*_HOO_ ≤ 102°. We hoped that, with these ranges, a clear local minimum of a target function could be found far from the borders of the ranges. We define the following target function
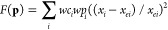
1where *x*_*i*_ is the value obtained for the property at given conditions, *x*_*ei*_ is its corresponding experimental value, and *i* runs on all (22) properties and conditions. In general, *wc*_*i*_ is 1 for the pure substance and 0.25 for the mixture with the SPC/E model, *wp*_*i*_ is 1 for ρ, ϵ, *P*_v_, and λ and 0.5 for μ. The rationale behind our *wc*_*i*_ selection is to incorporate the water–peroxide interaction in the training process while avoiding a large dependence of the specific choice of a water model. In addition, we have decreased some weights where we have observed strong fluctuations from the simulation results (the second row of Table S5 at the SI shows the weights). The weights are somewhat arbitrary, but the idea is to obtain a *holistic* model capable of capturing several properties at the same time. The same procedure with other weights could lead to a model able to better approach some particular properties of the list.

For all trained ANNs, we generate 1 × 10^6^ random **p** vectors to make predictions that are evaluated through eq 1. From this procedure, it turns clear the existence of several *F*(**p**) local minima for all ANNs. Next, we select the **p** reaching the *F*(**p**) minimum and locally search for even lower values by slightly changing **p**. Table S4 of the SI shows the final outcomes. Note that some but not all **p** from different ANNs are similar. Again, this occurs since there are several combinations of parameters leading to similar *F*(**p**) values. Note also that some **p** are very close to the borders of the defined ranges. This may suggest that a better fit to the experimental data could be obtained outside the boundaries we have imposed. However, it should be noted that for all of these cases, the predicted *a*_HOO_ values were either 94 or 102 degrees, which are beyond the experimental determinations for the solid and vapor phases.

Finally, starting from the values given in Table S4 that are not at the border of the ranges (we avoid getting outside the ranges), **p**_start_, we perform a local search by carrying out more MD simulations with **p**_try_ = **p**_start_ + **ξ** ⊙ Δ**p**, where **ξ** is a vector of [−0.5,0.5] homogeneously distributed random numbers, ⊙ is the Hadamard product, and Δ**p** has equal nonzero components in normalized space and a small magnitude. We present the best six **p**_try_ in Table S5. It should be mentioned that all these **p**_try_ produce low *F*(**p**_try_) with all trained ANNs. From this list, we have selected the **p**_try_ from the first row, i.e., from the model with the smallest error. However, due to fluctuations in the results and given the similar values of the errors, any of the six combinations should behave similarly. Note also that rows 1 and 6 and 2–5 are akin.

## Results

### Structural Properties of the H_2_O_2_ Bulk Liquid

For pure H_2_O_2_, *T* = 293 K, *P* = 1 bar, and by following the procedure described in the second paragraph of the Simulations Details section, we build Figure 2. The figure depicts the most important structural properties obtained for the bulk-liquid phase. These are the radial distribution functions for all sites (obtained by using the *rdf* tool), the probability density functions corresponding to the distribution of angles *a*_HOO_ and *a*_d_ (*angle* tool), and the dipole moment distribution (*dipoles* tool). We have also added the Ryckaert-Bellemans (RB) potential to show its effect on the *a*_d_ distribution.

The O–O *g*(*r*) shows a broad main peak around 0.30 nm, followed by a valley and a second-shell shallow maximum. Indeed, the peak shape, its height, and the complete *g*(*r*) are similar to those shown by Orabi and English,^18^ although the temperature is *T* = 273 K in their case. In contrast, our *g*(*r*) strongly differs from the one shown by Yu et al.^60^ The broad main peak appears to be the result of two overlapping peaks, indicating a parallel configuration of oxygen atoms where the two pairs are positioned closely to each other. Therefore, the cross-neighbors contribute to a peak that appears slightly further away. This finding aligns with the two most stable conformers of two-molecule aggregates observed in reference (18), as shown in Figure 2 of that reference for snapshots of several aggregates in the vapor phase of the pure substance. The H–H *g*(*r*) is also similar to the one shown by Orabi and English,^18^ having two close peaks at 0.23 and 0.27 nm. The former is due to the intramolecular contribution, whereas the second is due to the H–H distance involving a hydrogen bond.^18^ Again, the general shape, locations, and heights of the peaks are much closer to those given by Orabi and English than from the work of Yu et al.^60^ However, the principal peak of our O–H *g*(*r*), the one corresponding to the hydrogen bonds, has a height close to the value given by Yu et al. (around 4.5), contrasting with the smaller value from Orabi and English (around 3.5). The location of this sharp peak is close to 0.188. Finally, the general shape of the O–H *g*(*r*) resembles the one presented by by Orabi and English.^18^

The *a*_HOO_ probability distribution function, pdf, is given in the inset of Figure 2a. The peak of the distribution is placed around 96°, in good agreement with the minima set for this angle. In addition, it is closer to the value of the vapor phase than the one of the solid, close to the values set by Caputo et al.^13^ and to the experimental values given by Redington et al.^61^Figure 2b depicts the dihedral *a*_d_ pdf and the RB contribution to the energy, both even functions peaking at ±104 and 0°, respectively. The peak of *a*_d_ is close to the one found by Moin et al.^12^ Note that there are no *cis* configurations, given the large RB peak, but *trans* configurations are allowed. For our model, the *trans* state turns a 100% pure right enantiomer liquid in a 50–50% mixture in a few tens of picoseconds. Finally, the inset of Figure 2b shows the dipole pdf of H_2_O_2_ molecules. The distribution peaks at 2.69 D and is asymmetric, showing a tail toward zero that correspond to the *trans* configuration. Note that μ monotonously increases for decreasing |*a*_d_|.

### Water–H_2_O_2_ Bulk-Liquid Mixtures

**Figure 3:**
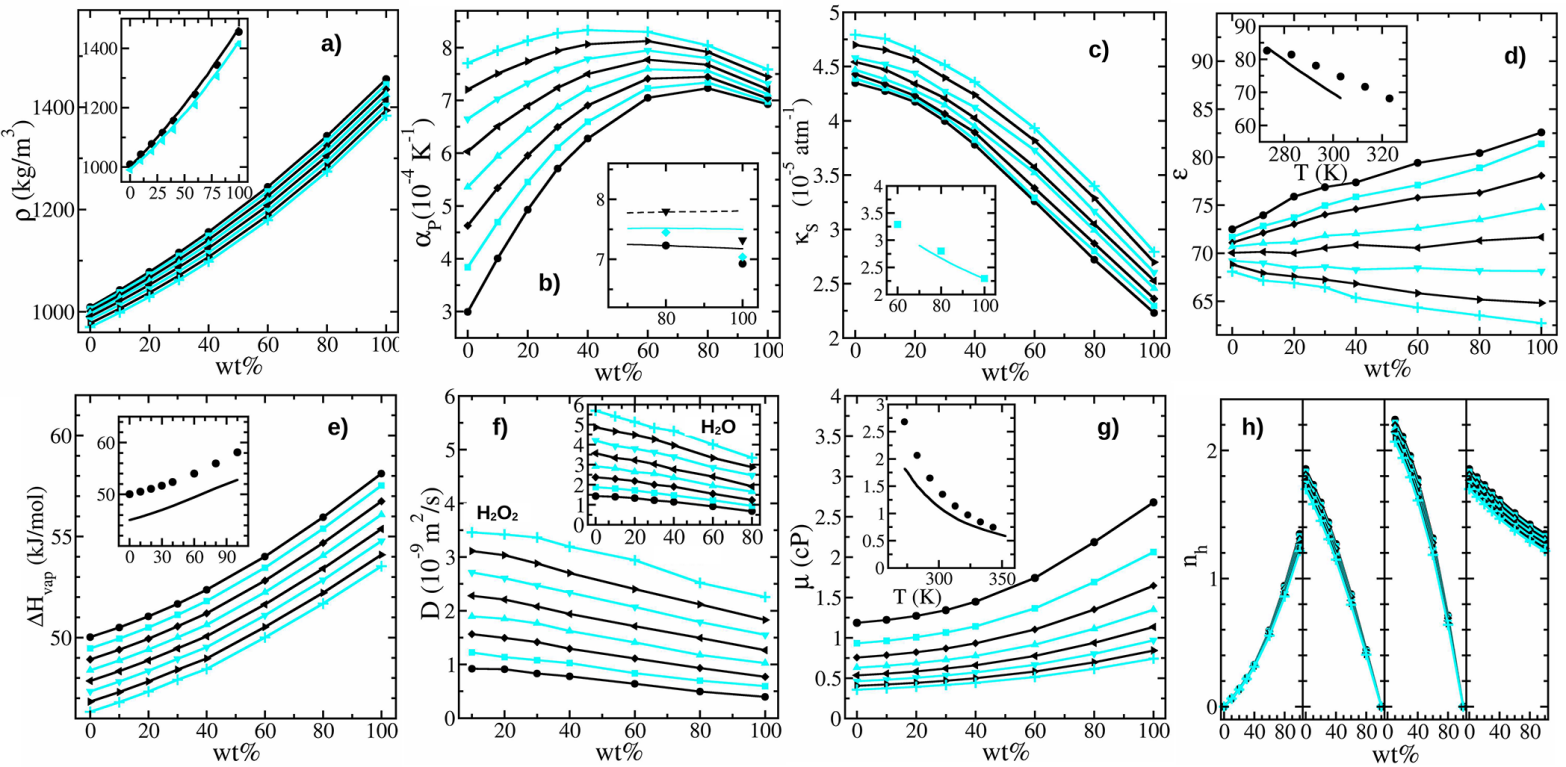
(a) Density, (b) thermal expansion coefficient, (c) adiabatic compressibility, (d) relative dielectric constant, (e) enthalpy of vaporization, (f) diffusion coefficient, (g) viscosity, and (h) the number of hydrogen bonds per oxygen atom as a function of the weight percentage of H_2_O_2_ in the water–peroxide mixture. For all curves, circles, squares, diamonds, triangles (up, left, down, right), and plus symbols correspond to *T* = 273, 283, 293, 303, 313, 323, 333, and 343 K, respectively. The insets compare the simulation results (symbols) with experimental data (lines) for (a) *T* = 273 and 313 K, (b) 273, 298, and 323 K, (c) 283 K, (d) pure H_2_O_2_, (e) 273 K, and (f) pure H_2_O_2_. The main panel (f) shows the diffusion coefficient for H_2_O_2_ molecules and the inset for H_2_O. The four plots of panel (h) depict, from left to right, *n*_h_ for H_2_O_2_ - H_2_O_2_, H_2_O - H_2_O, H_2_O_2_ - H_2_O, and the complete system. *n*_h_ is given per number of oxygen atoms of H_2_O_2_ molecules except for H_2_O - H_2_O, which is given in terms of the number of H_2_O molecules, and the total, given in terms of the total number of oxygen atoms. Insets without labeled axes share the same axes as the main panel. Experimental data in insets of (a) and (e) are taken from Easton et al.^62^ and Giguère et al.,^63^ respectively. Experimental data for all other insets are taken from the Constantine and Cain handbook,^4^ which summarizes and provides fits from data of different sources.^55,64−66^ Simulation outcomes are given in Tables S6–S13 of the SI.

Figure 3 shows several properties of the bulk-liquid mixtures of H_2_O_2_ and H_2_O as a function of the weight percentage of H_2_O_2_, wt %, and *T*. These are the density, ρ, the thermal expansion coefficient, α_P_, the adiabatic compressibility, κ_S_, the relative dielectric constant, ϵ, the enthalpy of vaporization, Δ*H*_v_, the diffusion coefficient of H_2_O_2_ and H_2_O, *D*, the viscosity, μ, and the number of hydrogen bonds per oxygen atom, *n*_h_.

As expected, ρ increases with wt % and decreases with *T*. Furthermore, the ρ increasing rate with wt % also augments with wt %. The inset of panel a of Figure 3 compares the simulation results with experimental data from Easton et al.^62^ for *T* = 273 K (circles and black line) and *T* = 313 K (triangles-left and cyan line). The agreement is very good. In addition, the obtained results for the SPC/E water model agree well with those reported in previous works.^52^ The ρ dependency with temperature allows us to compute
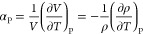
2by fitting a cubic polynomial to ρ(*T*) from where we obtain its partial derivative appearing at the right-hand side of eq 2. α_P_ can also be computed from fluctuations^53^ by using the *energy* tool, which leads to a general good agreement with the fitting procedure. However, we have noticed that the fitting procedure yields smoother trends, the ones reported in Figure 3b. The inset shows a comparison with experimental data from the Constantine and Cain handbook^4^ for large wt % and for *T* = 273 K (solid black line), 298 K (solid cyan line), and 323 K (black dashed line). Note that simulations correspond to *T* = 273 K (black circles), 293 K (cyan diamonds), and 323 K (black triangles-down).

Fluctuations in the NPT ensemble^53^ allow to compute the isothermal compressibility, κ_T_, the heat capacity, *C*_P_, and the heat capacity difference, *C*_P_ – *C*_V_ = *TVα*_P_^2^/κ_T_. Hence, it is possible to calculate the adiabatic compressibility from κ_S_ = κ_T_*C*_V_/*C*_P_ and compare it with experimental data. Note that *C*_V_ is always lower than *C*_P_, otherwise it would imply κ_T_ < 0 and a violation of the second law.^67^ For pure H_2_O_2_ and *T* = 283 K, *C*_V_/*C*_P_ yields 0.980 and decreases slightly with wt % at constant *T*. The model results for κ_S_ are given in the main panel c of Figure 3, whereas the comparison with experiments^4^ at *T* = 283 K and large wt % is given in the corresponding inset. The matching of both series is very good.

The *dipoles* gromacs tool computes the total dipole moment, **M**, of the simulation box as a function of time, its mean squared value, ⟨**M**^2^⟩, and its mean value to the square, ⟨**M**⟩^2^. From these quantities, it follows the relative dielectric constant, ϵ,^53,68^ a property that impacts the penetration of the electric field into the liquid bulk and, hence, the long-range electrostatic interactions. The main panel d of Figure 3 shows the simulation results and the inset a comparison with experimental data^4,66^ for pure H_2_O_2_. Note that, despite performing relatively large runs, the data are noisy. We estimate error bars close to 5% (not shown to gain clarity). There is a general decreasing trend of ϵ with increasing temperature for all mixtures. However, the ϵ trend with wt % changes from increasing at low temperatures to decreasing at higher ones. In fact, for *T* = 313 and 323 K, the ϵ(wt %) curves are practically flat. Consequently, the ϵ window for the SPC/E model is smaller than for the H_2_O_2_ one. We have found a very good agreement between simulations and the experimental results^4,66^ for pure H_2_O_2_ at 273 K, which worsens a little with increasing *T*. The agreement between our data and previous SPC/E results is also good.^69^

The enthalpy of vapor formation, Δ*H*_vap_, measures the overall attractive interaction between molecules, and it is frequently used to calibrate molecular models. It is given by
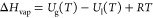
3where *U*_g_(*T*) is the potential energy of an isolated (gas) molecule at *T*, *U*_l_(*T*) is the potential energy of the bulk liquid per molecule at *T*, and *R* = 8.31446 J mol^–1^. Note that this expression assumes that the specific volume of the liquid is much smaller than the corresponding gas and that the kinetic energy of vapor and liquid are the same. In addition, it does not account for quantum corrections. We can also assume that *U*_g_(*T*) = *U*_g_^min^ + *RT*(3*N*_sites_ – 6 – *N*_c_)/2, with *N*_sites_ = 4, *N*_c_ = 0, and *U*_g_^min^ = −1.53 kJ mol^–1^ for our H_2_O_2_ model (the minimum potential energy), is the gas potential energy (the center of mass displacement and the rotation of the molecule around its principal axes do not contribute to the potential energy)^70^ or compute *U*_g_(*T*) directly from an isolated molecule. In this last case, we constrain the displacement and rotation of the molecule so that only the degrees of freedom that interact with the internal potential energy are allowed. Both methods yield similar results.^71^ Note also that *U*_g_(*T*) is a constant for a rigid molecule such as the SPC/E. Our results compare well with the 48.8 kJ mol^–1^ obtained for the SPC/E model at 301 K.^72^ Including self-polarization energy corrections^45^ to the Δ*H*_vap_ of SPC/E leads to 43.6 kJ mol^–1^ at the same *T*.^72^ The Δ*H*_vap_ results are shown in panel e of Figure 3. As expected, Δ*H*_vap_ increases with wt % and decreases with *T*. In addition, the Δ*H*_vap_ change with wt % increases with wt %. Our H_2_O_2_ model and its mixtures with the SPC/E one follow the general trend of the experimental determinations of Giguère et al.^63^ but overestimate Δ*H*_vap_ for all wt % at *T* = 273 K. Note also that, as for SPC/E water, this overestimation may be explained in terms of the self-polarization correction. The experimental dipole moment of the isolated H_2_O_2_ molecule is reported to range between 2.05 and 2.26 D (Constantine and Cain handbook;^4^ Gross and Taylor^66^). Considering the H_2_O_2_ polarizability is 30% larger than that of water^73^ and the 2.69 D value obtained for our model at 273 K, the self-polarization correction to the energy predicts a range of 6.6 to 3.0 kJ mol^–1^. This range covers the difference between the predicted and experimental^63^ Δ*H*_vap_ values for pure H_2_O_2_. Finally, it is worth mentioning that, although we did not consider the Δ*H*_vap_ as a target property, we took into account the vapor pressure and the surface tension, which are also strongly related to the intermolecular attraction.

The diffusion coefficient, *D*, is measured from the linear relation appearing between the mean square displacement, <|**r**(*t*) – **r**(0)|^2^>, and time, by making use of the Einstein relation, lim_*t*→*∞*_ < |**r**(*t*) – **r**(0)|^2^ > = 6*Dt*.^53^ Here, **r**(*t*) and **r**(0) are the vectors defining the position of the center of mass of the molecule at time *t* and 0, respectively. The computation is carried out with the gromacs tool *msd*. *D* is measured for H_2_O_2_ and H_2_O for the different compositions. The main panel f of Figure 3 shows the results for H_2_O_2_, and the inset depicts those for H_2_O. The values obtained for pure SPC/E at *T* = 293 K, (2.38 ± 0.05) × 10^–9^ m^2^ s^–1^, and *T* = 303 K, (2.91 ± 0.04) × 10^–9^ m^2^ s^–1^, agree with the one given by Mark and Nilson^74^ at *T* = 298 K, 2.75 × 10^–9^ m^2^ s^–1^. Also, the experimental diffusion coefficients of diluted peroxide in water^75^ are (0.88 ± 0.04) × 10^–9^ m^2^ s^–1^, (1.35 ± 0.04) × 10^–9^ m^2^ s^–1^, (1.75 ± 0.05) × 10^–9^ m^2^ s^–1^, and (2.2 ± 0.04) × 10^–9^ m^2^ s^–1^ at *T* = 283, 293, 303, 313 K, which compare well with our data for wt % = 10 of (1.12 ± 0.05) × 10^–9^ m^2^ s^–1^, (1.46 ± 0.05) × 10^–9^ m^2^ s^–1^, (1.79 ± 0.05) × 10^–9^ m^2^ s^–1^, and (2.19 ± 0.05) × 10^–9^ m^2^ s^–1^ at the same temperatures. The experimental values reported by van Stroe-Biezen et al.^76^ are also similar. As expected, *D* for H_2_O_2_ and H_2_O decreases with increasing wt % and decreasing *T*.

As mentioned in the Simulations Details section, viscosities are calculated from nonequilibrium simulations^54^ with an acceleration amplitude of 0.05 nm ps^–2^. Results are shown in panel g of Figure 3. The μ values we have obtained for the pure SPC/E at *T* = 293 and 303 K are (0.756 ± 0.006) cP and (0.631 ± 0.005) cP, respectively, which can be compared with the value at 298 K of 0.729 cP obtained by González et al.^77^ (the experimental value is 0.896 cP^78^). μ increases with H_2_O_2_ concentration and decreases with *T*. The inset compares the simulation results with the experimental outcomes reported by Phibbs and Giguère^55^ for pure H_2_O_2_. The disagreement with experiments diminishes with increasing *T*, but for *T* = 273 K, the overestimation reaches around 100%. In addition, viscosities were obtained by constraining the distance *d*_OH_ and the angle *a*_HOO_. Without these constraints, the overestimation gets even worse. Thus, we can only claim that the μ trends are captured with our model. Note that the μ values given in Tables S1 and S2 frequently overestimate the experimental values.^55^

The last panel of Figure 3 shows the average number of hydrogen bonds per frame, *n*_h_, formed among H_2_O_2_ molecules (leftmost inset), H_2_O molecules (left middle inset), H_2_O_2_ with H_2_O molecules (right middle inset), and the total (rightmost inset). *n*_h_ is given in terms of the number of oxygen atoms of H_2_O_2_ molecules except for hydrogen bonds formed among H_2_O molecules, given per number of H_2_O molecules, and for the total, given in terms of the total number of oxygen atoms. The hydrogen bonds are computed by setting a cutoff distance for the donor–acceptor of 0.35 nm and a cutoff angle for the hydrogen-donor–acceptor sites of 30°. For the pure substances, *n*_h_ is larger for H_2_O (1.86 at *T* = 273 K) than for H_2_O_2_ (1.35 at *T* = 273 K). In addition, the *n*_h_ ratio between the two quantities is practically constant when temperature increases from 273 to 343 K. On the other hand, *n*_h_ among H_2_O_2_ and H_2_O molecules reaches 2.24 at *T* = 273 K at high H_2_O_2_ dilution. Overall, the total *n*_h_ smoothly decreases with increasing wt %. Note that the *n*_h_ dependence with *T* is small and similar for all hydrogen bonds. Note also that the increase of Δ*H*_vap_, λ, and *T*_c_ with augmenting wt % is not due to the formation of more hydrogen bonds per oxygen atom but per molecule (from 1.86 to 2.7 at *T* = 273 K). Indeed, also the density of hydrogen bonds increases with wt %. In addition, the larger oxygen density also yields a larger LJ contribution. For *T* = 273 K, the ratio between *n*_h_ for the pure substances is 1.375 in favor of H_2_O, but the density ratio is 1.442 in favor of H_2_O_2_. Thus, *n*_h_ in terms of volume increases 1.05 times when going from pure H_2_O toward pure H_2_O_2_, which partially explains the 1.16 ratio of Δ*H*_vap_. The former quantity slightly diminishes with increasing *T*, as also happens with the Δ*H*_vap_ ratio. Finally, these results generally agree with the ones reported by Orabi and English,^18^ although they report a larger *n*_h_ for the pure H_2_O_2_ substance (around 1.7 at 273 K).

### Water–H_2_O_2_ Liquid–Vapor Mixtures

**Figure 4:**
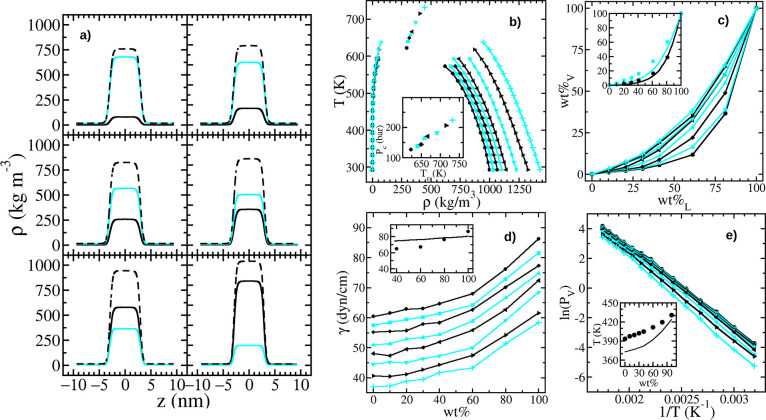
(a) Density profiles for H_2_O_2_ (solid black lines), H_2_O (solid cyan lines), and total (black dashed lines). All curves correspond to *T* = 533 K, and the wt % increases from left to right and from top to bottom (10, 20, 30, 40, 60, and 80). (b) Vapor–liquid phase diagram. Critical points are shown as unconnected symbols. The inset shows the critical pressure against the critical temperature. (c) wt %_v_ against wt %_l_ of the coexisting phases. The inset compares simulation results for *T* = 313 K (circles) and 393 K (squares) with experimental data^79^ (black lines for *T* = 313 K and cyan lines for *T* = 394 K). (d) Surface tension as a function of wt %. The inset compares the outcomes from the model (circles) with experimental data^55^ (solid line) for *T* = 293 K. (e) Natural logarithm of the vapor pressure against the reciprocal temperature. The inset compares the predicted boiling point temperatures (circles) with the experimental values^80^ (solid line) as a function of wt %. In panels (b) and (e), circles, squares, diamonds, triangles (up, left, down, right), and plus symbols correspond to 0, 10, 20, 30, 40, 60, 80, and 100 of wt %. In panels (c) and (d), circles, squares, diamonds, triangles (up, left, down, right), and plus symbols correspond to *T* values from 293 to 433 K in steps of 10 K. Insets without labeled axes share the same axes as the main panel. Simulation outcomes are given in Tables S14 and S15 of the SI.

As explained in the Simulations Details section, we access the vapor–liquid coexistence by placing a slab of liquid surrounded by its vapor in a prismatic cell with a height (*z*-axis) 3 times larger than its other two sides.^56,57^ This implementation is an alternative to the Gibbs ensemble technique, with the advantage that it allows accessing the surface tension (we do it following the virial route,^53^ but one can also introduce area changes for this purpose^81^). The obtained density profiles for H_2_O_2_, H_2_O, and the total are shown in panel a of Figure 4. The panel makes clear that these water and peroxide models produce fully mixed mixtures for all wt %. That is, there are no H_2_O_2_ or H_2_O aggregates leading to bumps in the liquid profiles. There is, however, a slight preference for water to place at the liquid–vapor interface, displacing peroxide toward the bulk liquid. Indeed, the proportion of peroxide in the vapor phase is lower than in the liquid.

Panel b of Figure 4 is built from liquid–vapor profiles such as those shown in panel a. Here, the inner liquid–vapor coexistence corresponds to the pure SPC/E model and the outermost to the pure H_2_O_2_ one. The critical points are obtained by using the law of rectilinear diameters^82^ and a scaling law with a critical exponent of 0.32.^83,84^ For the SPC/E model, we get a critical point with *T*_c_ = 622.8 K, ρ_c_ = 291.9 kg m^–3^, and *P*_c_ = 126.6 bar, which is in good agreement with the ones previously reported.^85,86^ On the other hand, the critical point for the pure peroxide model leads to *T*_c_ = (733 ± 8) K, *P*_c_ = (224 ± 6) bar, and ρ*c* = (446 ± 10) kg m^–3^, which matches the experimental data reported by Nikitin et al.,^87^*T*_c_ = 728 K and *P*_c_ = 220 bar (see the references therein for values obtained by other authors). In between the pure water and peroxide, the critical density and temperature smoothly vary as shown in Figure 4b. The inset of this same panel depicts the critical pressure against its temperature for the different peroxide concentrations.

For a fixed overall wt % of a simulation cell, H_2_O_2_ (H_2_O) increases (decreases) its concentration in the bulk liquid and decreases (augments) it in the vapor phase. Thus, when plotting the vapor weight percentage of H_2_O_2_, wt %_v_, against the liquid one, wt %_l_, the points shift from the bisector of the axes to the right and bottom (see Figure 4c). The smaller the temperature, the larger the deviation from the wt %_v_ = wt %_l_ line. This behavior is in qualitative agreement with the experiments reported by Scatchard et al.^79^ In addition, the wt %_v_(wt %_l_) curves from experiments^4,79^ and simulations practically coincide at low temperatures (see the black line and the circles obtained at *T* = 313 K of the inset). However, the good quantitative agreement worsens when increasing temperature (see the cyan line and the squares obtained at *T* = 393 K of the inset).

Panel d of Figure 4 shows the surface tension, λ, as a function of wt %, whereas its inset provides a comparison with experimental data from Phibbs and Giguère^55^ for *T* = 293 K. The surface tension values for the pure SPC/E model agree with previously reported data.^57,81^ The surface tension increases with wt % and decreases with *T* as a consequence of the varying strength of the intermolecular interactions, following a trend similar to that of Δ*H*_vap_. Thus, it is not very surprising that our H_2_O_2_ model overestimates the λ value. At *T* = 293 K, our value is (86.3 ± 1.2) dyn cm^–1^, which is 7% larger than the 80.5 dyn cm^–1^ value reported by Phibbs and Giguère.^55^ However, the overestimation disappears for 80 wt % and changes sign for wt % < 80, as the SPC/E model underestimates the water surface tension.

Finally, the vapor pressure is depicted in panel e of Figure 4 as ln(*P*_v_) as a function of 1/*T*. This representation is common due to the Clausius–Clapeyron equation, ln(*P*_v1_/*P*_v2_) = Δ*H*_vap_(1/*T*_2_ – 1/*T*_1_)/*R*, from where Δ*H*_vap_ can be computed. However, the error in the determination of *P*_v_ does not allow for a good estimation of Δ*H*_vap_ through this path. Indeed, the relative error of *P*_v_ strongly increases with decreasing *T* when employing the virial route. Thus, for *P*_v_ < 2 bar, we are computing *P*_v_ from ρ_v_ by assuming an ideal behavior for the vapor phase. In turn, these data are employed to estimate the boiling temperature by fitting a second-order polynomial to *P*_v_(*T*) for low *P*_v_ and its intersection with *P*_v_ = 1.013 bar. The resulting boiling temperature is depicted against wt % and compared with experimental data from Giguère and Maass^80^ in the inset. Note that the agreement is good when close to 100 wt %.

## Conclusions

We used an ANN-driven approach to optimize a classical model for hydrogen peroxide based on the OPLS-aa force field equations.^26−28^ The model does not include an LJ contribution to hydrogen sites and includes an RB dihedral function. The ANN optimization allowed us to consider several thermodynamic properties of water–hydrogen peroxide mixtures evaluated at different temperatures and compositions as targets. We explored the results of several ANNs and selected the ones that minimized the differences between computations and experimental data. For this last step, we assigned weights to each property based on the uncertainties of the simulation results and composition. We set lower weights when considering mixtures to avoid a strong dependence on the selected water model (SPC/E^45^). However, we believe it is important to target not only properties for pure substances but also mixtures.

Overall, the model produces outcomes that are in good agreement with experimental data.^4,55,62−66,79,80^ The best matches are found for properties such as density as a function of temperature, adiabatic compressibility, relative dielectric constant, diffusion coefficient for diluted mixtures in water, critical properties, and vapor composition at low temperatures. However, there are larger mismatches observed for viscosities at low temperatures and vapor composition at high temperatures, which are overestimated by the model. We believe that the model predictions for mixtures could improve with the consideration of more sophisticated water models such as TIP4P,^24^ TIP4P-2005,^88^ or TIP4P-ϵ.^25^ Note that creating a model that can fit all properties under all conditions is a challenging task. For example, decreasing the attraction of the LJ parameter between oxygen atoms could improve the enthalpy of vapor formation but worsen the vapor composition. Furthermore, the interplay between all parameters is not trivial. Fortunately, ANNs can take this complex interplay into account and may become the preferred tool for designing new molecular models.

## Supporting Information Available

The *itp* file defining the H_2_O_2_ model; tables to train (Table S1) and test (Table S2) the artificial neural networks (ANNs), results from different ANNs (Table S3), optimized parameters from the ANNs (Table S4), and the final results from a local search around the outcomes of the ANNs (Table S5). Tables S6 to S15 contain the simulation results shown in all panels of Figures 3 and 4 (PDF)
