# Supplementary material for: A Neural-Network-Optimized Hydrogen Peroxide Pairwise Additive Model for Classical Simulations
Source: J Chem Theory Comput. 2023 Jun 12;19(13):4172–81. doi: 10.1021/acs.jctc.3c00287 (PMC10921400; doi:10.1021/acs.jctc.3c00287)
Supplement: Supplementary file 1 — ct3c00287_si_001.pdf [file ct3c00287_si_001.pdf]

Supporting Information
A neural network optimized hydrogen peroxide model for classical simulations

Alvaro Ramos Peralta and Gerardo Odriozola*
Área de Física de Procesos Irreversibles, División de Ciencias Básicas e Ingeniería,
Universidad Autónoma Metropolitana-Azcapotzalco,
Av. San Pablo 180, 02200 Ciudad de México, Mexico
(Dated: May 25, 2023)

In this supporting information, we include the *itp* file defining the H_2O_2 model, the tables we employed to train (Table S1) and test (Table S2) the artificial neural networks (ANNs), the results we obtain from different ANNs (Table S3), the optimized parameters from the ANNs (Table S4), and the final results obtained by performing a local search around the outcomes from the ANNs with extra molecular dynamics simulations (Table S5). Finally, Tables S6 to S15 include the results shown in all panels of Figs. 3 and 4 of the manuscript.

The following is the hperoxide.itp file we employed to define the final peroxide model.

```
[ moleculetype ]
; molname nrexcl
PER 3

[ atoms ]
; nr type resnr residue atom cgnr charge mass
1 opls_per 1 PER O1 1 -0.4323
2 opls_per 1 PER O2 1 -0.4323
3 opls_117 1 PER H1 1 0.4323
4 opls_117 1 PER H2 1 0.4323

[ bonds ]
; i j funct length force. c.
1 2 1 0.1463 267776
1 3 1 0.0979 502416
2 4 1 0.0979 502416

[ angles ]
; i j k funct angle force. c.
1 2 4 1 95.79 628.02
2 1 3 1 95.79 628.02

[ dihedrals ]
3 1 2 4 3 8.404 -26.91 26.91 -8.081 0.0 0.0

[ exclusions ]
1 2 3 4
2 1 3 4
3 1 2 4
4 1 2 3
```

The following line is needed in the ffnonbonded.itp file of the force field (it could be placed at the end). Note that it is best to copy and paste the complete force field directory to make changes.

```
; H2O2 Oxygen atom
opls_per 8 15.99940 -0.4503 A 2.9989e-01 8.9120e-01
```

---

*Electronic address: godriozo@azc.uam.mx

Table S1. Simulation results employed for training the artificial neural networks (ANNs). Legth units are given in nm, charge in elementary charge unit, angles in degrees, energy in kJ/mol, ρ in kg/m^3, ϵ is dimensionless, μ in cP, P_v in bar, and λ in 10^{-4} N/m. The first line shows the target experimental values taken from Easton et al. [1] and Giguère and Geoffrion [2] for ρ, Gross and Taylor [3] for ϵ, Phibbs and Giguère [4] for μ and λ, and Scatchard et al. [5] and Giguère and Maass [6] for P_v and wt%$_v$. Note that most of these experimental data are summarized in the Constantine and Cain handbook [7].

Model Parameters							70wt%												100wt%									
							293K						373K			398K			293K					373K			423K	
c_2	q_H	σ_{OO}	ϵ_{OO}	d_{OO}	d_{OH}	a_{HOO}	ρ	ϵ	μ	P_v	λ	wt%$_v$	ρ	ϵ	μ	P_v	λ	wt%$_v$	ρ	ϵ	μ	P_v	λ	ρ	ϵ	μ	P_v	λ
experimental →							1288	73.0	1.24	0.007	772	18	1209	73.0	0.42	1.04	549	34	1450	76.0	1.25	0.002	802	1362	64.0	0.48	1.00	599
25.5	0.4317	0.2981	0.8570	0.1459	0.0976	95.3	1271	56.1	1.21	0.0070	635	33	1179	51.8	0.37	0.99	429	46	1452	46.9	1.54	0.0007	863	1361	37.8	0.51	1.17	545
25.5	0.4319	0.2962	0.8488	0.1469	0.0955	100.8	1282	48.5	1.15	0.0070	699	72	1183	48.1	0.36	1.41	443	56	1463	31.4	1.52	0.0026	780	1369	28.4	0.51	1.24	515
25.5	0.4322	0.2966	0.8038	0.1465	0.0951	98.4	1276	54.2	1.10	0.0050	668	53	1176	48.8	0.34	1.48	408	58	1454	41.4	1.25	0.0043	770	1357	30.8	0.46	1.78	517
25.5	0.4332	0.2994	0.8668	0.1463	0.0967	94.6	1259	66.8	1.02	0.0030	617	44	1165	54.6	0.32	1.40	431	62	1424	47.5	1.12	0.0014	765	1329	34.8	0.43	1.67	573
25.5	0.4354	0.2985	0.7296	0.1479	0.0988	98.3	1263	59.2	2.03	0.0090	613	6	1180	51.1	0.47	0.95	439	32	1454	48.2	4.02	0.0000	795	1370	41.5	0.77	0.67	587
25.5	0.4365	0.2967	0.7028	0.1460	0.0976	94.8	1285	84.1	1.94	0.0050	611	0	1195	60.4	0.44	1.12	463	36	1486	58.8	3.36	0.0007	811	1400	45.6	0.71	0.83	563
25.5	0.4370	0.2984	0.8148	0.1470	0.0964	96.8	1265	54.8	1.22	0.0040	592	16	1172	46.1	0.36	1.12	455	50	1441	45.6	1.66	0.0023	810	1349	32.8	0.51	1.22	537
25.5	0.4376	0.2997	0.8310	0.1467	0.0983	101.7	1257	52.2	1.75	0.0060	626	0	1175	47.9	0.42	0.83	440	24	1439	35.9	3.04	0.0000	822	1356	34.5	0.68	0.54	583
25.5	0.4385	0.2971	0.8314	0.1462	0.0951	97.8	1277	68.7	1.20	0.0040	671	28	1180	45.8	0.36	1.40	457	52	1458	44.0	1.49	0.0034	804	1365	33.2	0.51	1.37	505
25.5	0.4391	0.2995	0.7880	0.1469	0.0980	97.2	1258	48.9	1.62	0.0020	617	0	1176	52.3	0.41	0.92	453	37	1442	51.1	2.57	0.0000	777	1357	41.1	0.65	0.74	557
25.5	0.4409	0.2982	0.7204	0.1451	0.0963	95.7	1272	56.4	1.44	0.0070	621	29	1182	54.8	0.39	1.16	457	46	1461	59.0	2.25	0.0005	779	1372	41.5	0.60	1.13	535
25.5	0.4411	0.2987	0.7262	0.1458	0.0979	98.8	1271	76.4	2.09	0.0090	648	0	1186	47.8	0.47	0.89	475	30	1464	45.5	4.64	0.0006	783	1381	40.7	0.81	0.46	596
25.5	0.4412	0.2983	0.8682	0.1466	0.0961	98.3	1272	55.6	1.39	0.0040	654	18	1179	45.1	0.38	1.03	425	38	1449	43.8	1.84	0.0000	817	1362	36.7	0.57	0.87	603
25.5	0.4412	0.2996	0.8084	0.1467	0.0959	98.5	1254	53.3	1.20	0.0060	608	33	1166	51.8	0.36	1.16	474	55	1427	46.4	1.69	0.0006	791	1337	33.5	0.52	1.14	575
25.5	0.4414	0.2968	0.7382	0.1467	0.0942	97.4	1279	66.1	1.26	0.0090	646	12	1178	47.7	0.36	1.34	412	57	1458	47.4	1.62	0.0000	743	1364	34.1	0.50	1.63	502
25.5	0.4422	0.2975	0.7350	0.1451	0.0989	98.0	1288	73.2	3.16	0.0040	661	0	1204	58.1	0.57	0.88	448	17	1495	78.0	9.02	0.0000	833	1417	51.5	1.07	0.27	667
25.5	0.4424	0.2988	0.7556	0.1452	0.0946	94.2	1262	57.1	1.02	0.0050	630	64	1168	51.8	0.33	1.56	433	66	1436	53.6	1.23	0.0058	754	1339	36.6	0.44	2.21	504
25.5	0.4428	0.2963	0.8256	0.1468	0.0968	98.5	1295	75.3	2.17	0.0070	683	0	1202	52.6	0.47	1.03	450	21	1489	46.5	3.78	0.0014	855	1406	42.4	0.78	0.53	633
25.5	0.4440	0.2997	0.8848	0.1457	0.0948	100.6	1259	50.1	1.15	0.0070	639	84	1169	44.7	0.35	1.17	447	46	1428	38.5	1.47	0.0000	797	1338	30.3	0.50	1.13	543
25.5	0.4442	0.2975	0.7272	0.1472	0.0985	95.3	1284	74.5	3.25	0.0090	621	0	1199	65.2	0.54	0.90	473	30	1486	51.1	6.86	0.0003	815	1407	55.8	0.99	0.34	640
25.5	0.4445	0.2990	0.7338	0.1458	0.0989	97.8	1274	77.2	2.67	0.0050	599	0	1193	56.0	0.54	0.82	493	25	1471	64.2	7.58	0.0000	871	1393	52.0	1.04	0.31	595
25.5	0.4448	0.2962	0.8296	0.1479	0.0978	96.4	1291	86.1	2.59	0.0080	662	0	1205	61.8	0.53	0.85	482	20	1496	58.3	5.90	0.0000	866	1416	47.3	0.93	0.34	613
25.5	0.4451	0.2965	0.7440	0.1464	0.0963	98.0	1293	73.7	2.19	0.0030	653	23	1199	52.6	0.48	0.97	463	31	1490	53.6	4.42	0.0000	784	1406	43.5	0.81	0.52	583
25.5	0.4453	0.2974	0.8802	0.1461	0.0971	96.7	1288	76.2	1.87	0.0030	664	0	1198	51.9	0.45	0.96	478	28	1481	56.0	3.31	0.0000	864	1398	44.7	0.76	0.49	679
25.5	0.4470	0.3000	0.8924	0.1455	0.0979	98.0	1266	69.6	1.80	0.0080	657	61	1186	53.8	0.45	0.85	478	22	1451	52.6	3.46	0.0000	853	1370	45.4	0.75	0.42	665
25.5	0.4480	0.2979	0.7604	0.1460	0.0978	100.7	1282	69.3	3.07	0.0060	666	24	1199	50.2	0.57	0.95	474	16	1483	52.8	9.34	0.0000	856	1406	45.6	1.12	0.26	649
25.5	0.4488	0.2996	0.7816	0.1451	0.0973	100.9	1269	72.4	2.26	0.0010	621	0	1187	47.8	0.50	0.94	444	23	1459	48.2	5.13	0.0000	817	1379	37.2	0.85	0.38	613
25.5	0.4488	0.2998	0.7848	0.1451	0.0983	94.2	1270	76.1	2.05	0.0030	633	0	1191	63.9	0.48	0.86	495	22	1463	59.4	4.32	0.0004	828	1382	53.8	0.81	0.43	627
25.5	0.4494	0.2988	0.8442	0.1455	0.0955	97.4	1274	67.8	1.51	0.0050	640	0	1183	50.7	0.40	1.04	483	37	1455	53.0	2.28	0.0000	801	1369	41.0	0.62	0.81	594
25.5	0.4511	0.2995	0.8226	0.1457	0.0950	101.5	1265	62.1	1.50	0.0070	617	32	1178	48.2	0.40	1.12	424	29	1442	46.3	2.42	0.0000	787	1357	34.2	0.64	0.69	580
25.5	0.4512	0.2988	0.8430	0.1461	0.0977	101.0	1275	57.1	2.72	0.0030	653	0	1195	52.8	0.53	0.79	500	10	1472	44.3	7.56	0.0000	906	1394	41.3	1.00	0.26	702
25.5	0.4517	0.2996	0.8454	0.1472	0.0984	98.7	1269	60.1	2.58	0.0150	652	0	1190	54.3	0.53	0.77	479	11	1459	51.6	7.18	0.0000	901	1382	44.1	1.00	0.21	702
25.5	0.4540	0.2999	0.8782	0.1475	0.0970	99.7	1262	64.5	2.13	0.0050	633	0	1183	55.4	0.47	0.89	475	18	1445	52.4	4.53	0.0000	851	1367	42.3	0.84	0.33	620
25.5	0.4541	0.2961	0.7758	0.1471	0.0943	101.0	1294	55.5	2.39	0.0080	691	0	1203	52.5	0.48	1.00	448	26	1489	45.7	4.38	0.0006	846	1406	40.0	0.81	0.56	616
25.5	0.4547	0.2965	0.8172	0.1467	0.0947	94.6	1291	71.9	1.67	0.0080	687	0	1202	57.2	0.44	1.27	469	41	1488	63.6	3.02	0.0009	855	1402	49.2	0.72	0.68	627
25.5	0.4553	0.2967	0.8506	0.1452	0.0945	97.0	1299	80.1	1.92	0.0080	701	37	1206	54.9	0.44	1.09	446	31	1492	58.8	3.13	0.0008	886	1407	48.8	0.72	0.58	623
25.5	0.4570	0.2999	0.7582	0.1470	0.0988	98.6	1272	68.3	4.07	0.0030	662	0	1195	62.1	0.66	0.73	551	7	1468	65.2	20.48	0.0000	891	1396	56.5	1.47	0.17	708
25.5	0.4574	0.2982	0.8374	0.1460	0.0940	101.6	1280	62.5	1.80	0.0080	647	0	1189	50.6	0.44	1.11	444	28	1463	43.5	3.11	0.0000	862	1380	34.4	0.72	0.58	628
25.5	0.4584	0.2997	0.8150	0.1458	0.0944	99.2	1267	63.5	1.64	0.0030	654	26	1180	53.6	0.42	1.07	468	39	1444	51.1	2.64	0.0010	802	1361	39.5	0.67	0.65	572
25.5	0.4586	0.2981	0.8358	0.1454	0.0944	99.6	1284	73.1	1.97	0.0080	688	0	1194	60.2	0.45	1.11	474	32	1472	48.6	3.53	0.0009	859	1390	38.8	0.76	0.43	646
25.5	0.4590	0.3000	0.7848	0.1466	0.0967	96.8	1270	85.6	2.42	0.0040	642	0	1188	63.2	0.51	0.88	454	24	1456	68.5	5.71	0.0000	839	1377	51.1	0.93	0.34	633
25.5	0.4592	0.2962	0.7018	0.1477	0.0951	98.9	1292	74.0	3.80	0.0050	666	0	1208	63.6	0.60	0.87	487	19	1501	53.7	11.65	0.0000	838	1424	46.9	1.19	0.30	639
25.5	0.4596	0.2988	0.8636	0.1470	0.0965	101.3	1280	60.2	3.06	0.0020	648	0	1196	49.5	0.55	0.82	496	12	1470	51.0	8.55	0.0000	935	1394	43.2	1.06	0.18	699
25.5	0.4606	0.2971	0.7516	0.1474	0.0971	100.9	1293	36.7	5.94	0.0040	649	0	1212	57.7	0.74	0.96	394	6	1504	47.1	37.05	0.0000	897	1431	52.6	1.82	0.11	743
25.5	0.4622	0.2969	0.7012	0.1463	0.0987	100.8	1308	58.9	17.17	0.0030	664	0	1227	57.9	1.06	0.78	490	2	1526	16.7	280.52	0.0000	989	1460	59.8	3.66	0.03	758

Continuation of Table S1.

c_2	q_H	σ_{OO}	ϵ_{OO}	d_{OO}	d_{OH}	a_{HOO}	ρ	ϵ	μ	P_v	λ	wt%$_v$	ρ	ϵ	μ	P_v	λ	wt%$_v$	ρ	ϵ	μ	P_v	λ	ρ	ϵ	μ	P_v	λ
25.5	0.4623	0.2986	0.7270	0.1464	0.0950	98.2	1278	66.2	2.62	0.0060	638	0	1194	55.1	0.52	0.87	501	22	1471	59.1	6.58	0.0000	828	1393	45.0	0.97	0.37	599
25.5	0.4625	0.2981	0.8652	0.1470	0.0965	102.0	1284	11.6	4.33	0.0080	674	0	1204	50.3	0.62	0.97	483	9	1485	43.5	14.31	0.0000	930	1411	44.4	1.29	0.16	729
25.5	0.4630	0.2973	0.8724	0.1452	0.0980	100.2	1305	58.4	5.92	0.0050	705	0	1223	62.0	0.77	0.86	519	5	1518	65.5	45.41	0.0000	957	1447	56.8	1.86	0.06	816
25.5	0.4658	0.3000	0.7298	0.1451	0.0987	95.2	1286	109.6	5.47	0.0020	643	0	1211	73.3	0.78	0.64	503	4	1492	56.3	38.81	0.0000	915	1422	78.8	1.95	0.07	762
25.5	0.4661	0.2997	0.7286	0.1463	0.0988	97.0	1284	70.7	5.86	0.0060	634	0	1210	67.9	0.85	0.76	496	3	1490	91.6	75.67	0.0000	923	1422	70.5	2.32	0.09	747
25.5	0.4665	0.2969	0.7234	0.1479	0.0984	98.5	1305	30.8	14.77	0.0030	676	0	1225	68.9	1.07	0.77	542	1	1523	35.2	197.07	0.0000	925	1456	72.5	3.40	0.04	815
25.5	0.4667	0.2982	0.8362	0.1480	0.0951	96.8	1281	98.7	2.91	0.0020	666	0	1198	67.0	0.54	0.88	548	22	1475	68.5	6.47	0.0000	874	1397	48.7	0.99	0.24	665
25.5	0.4668	0.2990	0.8518	0.1475	0.0957	99.8	1278	70.8	3.07	0.0040	670	0	1196	58.0	0.55	0.75	468	12	1469	57.2	9.43	0.0000	937	1393	46.3	1.12	0.18	659
25.5	0.4668	0.2991	0.7160	0.1471	0.0956	94.2	1277	103.1	2.92	0.0030	616	0	1196	74.0	0.56	0.84	491	21	1473	71.4	8.10	0.0000	829	1396	64.2	1.08	0.33	625
25.5	0.4671	0.2999	0.8902	0.1475	0.0986	94.2	1281	1.0	7.45	0.0050	656	0	1205	73.8	0.67	0.57	528	3	1476	73.1	17.05	0.0003	985	1405	73.3	1.47	0.09	776
25.5	0.4693	0.2970	0.8532	0.1473	0.0957	98.6	1301	77.8	4.63	0.0060	725	0	1216	66.1	0.66	0.92	509	5	1504	83.5	15.99	0.0000	968	1431	53.7	1.43	0.12	724
25.5	0.4319	0.2986	0.7286	0.1477	0.0951	100.8	1253	53.0	1.01	0.0080	1150	71	1155	43.2	0.32	1.54	400	63	1417	35.2	1.20	0.0030	714	1319	26.0	0.43	2.55	459
25.5	0.4600	0.2963	0.8664	0.1472	0.0968	100.5	1305	73.7	4.83	0.0060	1421	0	1220	60.1	0.68	0.91	505	9	1511	36.8	17.77	0.0000	954	1438	46.6	1.45	0.10	741
25.5	0.4309	0.2962	0.7112	0.1466	0.0952	100.5	1280	61.7	1.22	0.0100	1237	40	1178	47.8	0.36	1.42	398	60	1461	32.3	1.64	0.0058	755	1366	27.8	0.50	1.65	481
25.5	0.4690	0.2968	0.7946	0.1461	0.0943	100.8	1301	79.9	4.09	0.0040	1407	0	1216	56.6	0.60	0.97	532	18	1504	52.2	13.39	0.0000	921	1428	48.3	1.23	0.28	689
25.5	0.4322	0.2992	0.8972	0.1478	0.0984	97.9	1261	61.2	1.35	0.0030	1262	0	1172	46.7	0.38	0.98	494	41	1433	41.9	1.84	0.0028	836	1345	31.6	0.56	0.92	564
25.5	0.4428	0.2967	0.7636	0.1464	0.0975	101.1	1289	73.5	2.84	0.0050	1324	0	1201	50.1	0.53	0.81	491	18	1492	40.8	6.91	0.0000	885	1411	37.0	0.98	0.36	629
25.5	0.4441	0.2981	0.8922	0.1455	0.0951	99.3	1279	60.9	1.28	0.0090	1342	61	1183	48.2	0.37	1.27	474	42	1456	42.6	1.77	0.0005	861	1367	37.1	0.56	0.83	619
25.5	0.4327	0.2996	0.7782	0.1454	0.0972	98.7	1255	56.1	1.25	0.0060	1257	30	1167	48.8	0.35	1.26	481	57	1430	40.2	1.60	0.0000	749	1338	35.0	0.50	1.29	506
25.5	0.4474	0.2962	0.7270	0.1466	0.0986	94.6	1302	72.5	4.42	0.0050	1247	0	1217	69.0	0.64	0.86	423	9	1518	50.8	13.26	0.0000	915	1441	66.6	1.29	0.19	665
25.5	0.4372	0.2999	0.8720	0.1463	0.0963	98.9	1253	61.2	1.14	0.0040	1234	18	1165	45.2	0.35	1.23	436	52	1421	38.6	1.35	0.0015	810	1331	30.0	0.49	1.27	565
25.5	0.4621	0.2968	0.7686	0.1463	0.0966	95.7	1303	50.6	4.32	0.0030	1324	0	1218	74.0	0.65	0.89	457	11	1513	87.7	15.16	0.0007	916	1438	64.3	1.38	0.15	661
25.5	0.4666	0.2961	0.7490	0.1458	0.0977	99.2	1316	51.1	15.02	0.0070	1441	0	1236	71.3	1.03	0.76	565	2	1544	24.8	202.87	0.0000	1042	1473	59.7	3.03	0.05	778
25.5	0.4367	0.2983	0.7746	0.1477	0.0941	94.8	1252	74.7	0.90	0.0120	1208	52	1155	46.6	0.30	2.17	359	73	1417	38.1	0.91	0.0067	708	1315	31.2	0.38	3.16	432
25.5	0.4461	0.2987	0.8508	0.1450	0.0984	94.1	1283	69.0	2.02	0.0030	1260	0	1197	64.4	0.47	0.83	476	24	1476	67.8	3.73	0.0000	901	1395	53.9	0.80	0.34	634
25.5	0.4353	0.2960	0.7776	0.1452	0.0951	101.6	1289	62.8	1.40	0.0100	1323	70	1190	41.4	0.38	1.27	445	54	1477	37.2	1.92	0.0030	790	1385	26.5	0.56	1.19	565
25.5	0.4578	0.2983	0.8648	0.1465	0.0967	95.8	1284	78.3	2.55	0.0030	1361	0	1199	69.6	0.51	0.92	506	18	1478	61.4	5.28	0.0000	896	1400	55.7	0.92	0.25	702
25.5	0.4573	0.2988	0.8302	0.1463	0.0961	94.1	1279	84.2	1.89	0.0070	1294	0	1192	62.2	0.46	0.97	442	34	1467	69.4	3.50	0.0014	849	1386	58.8	0.78	0.42	667
25.5	0.4682	0.2982	0.7830	0.1467	0.0988	98.6	1299	96.5	10.89	0.0040	1353	0	1221	77.9	0.95	0.77	533	1	1512	81.9	172.81	0.0000	995	1444	63.0	3.00	0.05	736
25.5	0.4651	0.2976	0.7714	0.1473	0.0949	98.7	1284	79.5	3.18	0.0030	1305	0	1202	58.1	0.56	1.04	478	9	1484	61.7	9.23	0.0000	849	1407	52.4	1.09	0.27	672
25.5	0.4658	0.2995	0.7736	0.1461	0.0945	98.0	1271	65.4	2.03	0.0080	1284	10	1188	59.2	0.49	0.97	458	27	1458	63.0	4.89	0.0009	846	1378	50.4	0.86	0.50	608
25.5	0.4622	0.2999	0.8760	0.1473	0.0980	95.4	1275	25.7	3.05	0.0040	1307	0	1197	54.2	0.57	0.70	477	8	1465	64.7	8.44	0.0000	899	1392	59.6	1.15	0.16	710
25.5	0.4457	0.2999	0.8858	0.1466	0.0967	94.4	1262	64.1	1.29	0.0050	1288	0	1175	56.3	0.38	1.10	456	41	1434	57.4	1.72	0.0000	831	1347	44.6	0.56	0.91	569
25.5	0.4583	0.2962	0.8674	0.1463	0.0950	97.4	1302	77.8	2.27	0.0070	1395	0	1215	66.4	0.51	0.97	445	17	1502	60.8	4.93	0.0000	867	1421	49.2	0.89	0.34	674
25.5	0.4443	0.2974	0.8854	0.1468	0.0968	96.2	1283	66.1	1.66	0.0060	1302	0	1193	57.0	0.42	1.09	453	30	1473	50.5	2.61	0.0000	889	1388	45.5	0.68	0.58	620
25.5	0.4696	0.2980	0.8822	0.1467	0.0958	94.4	1292	82.7	3.04	0.0040	1424	0	1210	69.3	0.56	0.88	508	10	1492	84.1	7.96	0.0000	938	1417	64.8	1.11	0.16	708
25.5	0.4593	0.2974	0.7472	0.1455	0.0950	98.6	1288	61.8	2.62	0.0070	1299	0	1203	57.5	0.53	0.91	490	15	1491	50.5	6.61	0.0000	798	1411	48.1	0.98	0.36	641
25.5	0.4473	0.2972	0.7854	0.1464	0.0957	96.8	1280	65.3	1.74	0.0040	1310	0	1192	58.0	0.43	1.04	484	31	1476	50.7	2.85	0.0007	832	1390	45.1	0.69	0.73	600
25.5	0.4326	0.2975	0.7880	0.1453	0.0983	96.3	1279	59.4	1.70	0.0050	1284	0	1188	49.6	0.41	1.08	447	36	1473	52.1	2.54	0.0004	854	1385	38.6	0.63	0.85	566
25.5	0.4589	0.2992	0.8948	0.1451	0.0957	94.9	1282	88.3	1.78	0.0060	1309	0	1194	60.5	0.44	0.95	471	29	1465	75.2	3.06	0.0000	909	1384	53.8	0.72	0.46	645
25.5	0.4608	0.2991	0.7456	0.1456	0.0949	97.6	1275	81.1	2.04	0.0060	1250	12	1189	62.1	0.47	0.98	455	28	1463	66.1	4.70	0.0000	822	1382	49.4	0.81	0.48	593
25.5	0.4682	0.2990	0.7832	0.1462	0.0979	101.2	1286	75.6	6.95	0.0070	1360	0	1211	63.9	0.84	0.76	488	4	1493	59.4	91.92	0.0000	947	1425	53.5	2.28	0.06	764
25.5	0.4556	0.2969	0.7454	0.1456	0.0959	94.4	1298	81.7	2.62	0.0010	1277	0	1208	58.3	0.51	0.97	458	26	1501	84.4	5.75	0.0000	860	1420	56.5	0.92	0.50	640
25.5	0.4560	0.2986	0.8606	0.1478	0.0941	95.7	1272	70.5	1.37	0.0090	1320	0	1179	52.8	0.38	1.22	449	40	1445	55.1	1.91	0.0008	850	1358	44.3	0.58	0.88	579
25.5	0.4626	0.2982	0.7792	0.1460	0.0979	98.7	1292	78.3	5.40	0.0010	1344	0	1212	55.7	0.75	0.72	498	6	1500	69.3	36.29	0.0004	979	1429	59.0	1.75	0.08	736
25.5	0.4476	0.2969	0.8128	0.1463	0.0950	98.9	1286	57.9	1.76	0.0040	1352	0	1192	46.8	0.43	1.07	439	35	1475	50.7	2.75	0.0024	830	1389	33.4	0.66	0.64	565
25.5	0.4477	0.2993	0.7706	0.1479	0.0962	94.2	1261	79.1	1.41	0.0040	1208	30	1174	51.8	0.40	1.10	463	45	1440	60.3	2.16	0.0017	779	1352	40.8	0.60	1.00	541
25.5	0.4386	0.2962	0.8744	0.1453	0.0989	96.0	1299	59.9	2.24	0.0040	1333	14	1212	64.4	0.51	0.95	474	19	1508	61.7	4.46	0.0000	906	1427	53.7	0.89	0.31	687
25.5	0.4508	0.2975	0.8236	0.1460	0.0983	95.1	1290	70.1	3.22	0.0070	1354	0	1208	67.5	0.56	0.90	463	13	1498	77.1	7.69	0.0000	896	1421	59.1	1.08	0.26	707
25.5	0.4633	0.2966	0.8874	0.1468	0.0963	95.5	1302	77.5	3.47	0.0040	1452	0	1220	59.7	0.61	0.94	476	12	1510	71.3	9.39	0.0000	927	1434	64.7	1.17	0.12	747
25.5	0.4586	0.2971	0.7620	0.1469	0.0966	95.6	1294	57.7	3.70	0.0050	1283	0	1209	70.5	0.59	0.97	456	15	1501	70.4	10.67	0.0013	857	1424	58.6	1.17	0.31	670
25.5	0.4604	0.2995	0.8834	0.1460	0.0946	98.0	1272	28.3	1.72	0.0050	1311	0	1185	51.3	0.42	0.89	530	30	1451	52.9	2.75	0.0010	869	1368	42.5	0.69	0.58	657

Continuation of Table S1.

c_2	q_H	σ_{OO}	ϵ_{OO}	d_{OO}	d_{OH}	a_{HOO}	ρ	ϵ	μ	P_v	λ	wt%$_v$	ρ	ϵ	μ	P_v	λ	wt%$_v$	ρ	ϵ	μ	P_v	λ	ρ	ϵ	μ	P_v	λ
25.5	0.4658	0.2987	0.7012	0.1473	0.0943	98.6	1279	73.5	2.88	0.0050	1287	0	1191	60.4	0.53	0.92	466	23	1467	66.4	7.37	0.0000	838	1388	47.4	0.99	0.47	613
25.5	0.4451	0.2999	0.7862	0.1463	0.0968	98.1	1258	63.9	1.54	0.0050	1183	0	1174	48.3	0.40	0.98	462	37	1438	46.0	2.52	0.0005	789	1353	38.3	0.65	0.71	534
25.5	0.4664	0.2988	0.7266	0.1462	0.0973	95.1	1289	96.1	4.77	0.0130	1262	0	1210	78.8	0.70	0.76	419	10	1495	97.0	23.26	0.0000	877	1423	80.9	1.62	0.15	700
25.5	0.4566	0.2970	0.8016	0.1475	0.0968	95.9	1292	82.3	3.11	0.0060	1362	0	1206	55.7	0.56	0.90	445	14	1495	75.2	8.42	0.0000	886	1418	56.7	1.10	0.22	663
25.5	0.4557	0.2973	0.7968	0.1452	0.0980	101.4	1299	68.9	5.92	0.0030	1367	0	1215	53.2	0.69	0.87	475	4	1509	56.1	28.00	0.0000	900	1436	50.2	1.59	0.19	711
25.5	0.4578	0.2974	0.7496	0.1471	0.0989	96.9	1297	73.7	6.30	0.0090	1291	0	1216	59.1	0.81	0.69	473	11	1512	62.8	48.62	0.0000	883	1441	64.3	2.03	0.07	737
25.5	0.4399	0.2998	0.8498	0.1468	0.0981	98.8	1256	67.0	1.63	0.0060	1223	31	1176	58.8	0.42	0.82	337	31	1437	49.1	2.55	0.0000	799	1354	36.8	0.64	0.56	594
25.5	0.4410	0.2989	0.7686	0.1478	0.0956	94.5	1258	75.9	1.11	0.0100	1238	39	1165	49.4	0.34	0.59	743	88	1430	51.8	1.44	0.0000	761	1336	37.2	0.48	1.75	522
25.5	0.4464	0.2993	0.7984	0.1476	0.0968	94.9	1263	71.7	1.57	0.0050	1210	0	1177	54.1	0.41	0.96	442	38	1443	53.8	2.36	0.0005	767	1357	43.2	0.62	0.93	538
25.5	0.4394	0.3000	0.7952	0.1477	0.0956	96.7	1263	73.4	1.55	0.0040	1201	30	1177	59.3	0.40	1.01	463	39	1443	55.2	2.46	0.0050	820	1358	41.7	0.63	0.76	581
25.5	0.4310	0.2990	0.8794	0.1475	0.0963	101.1	1258	50.0	1.05	0.0120	1231	19	1163	47.3	0.34	1.39	407	53	1422	35.9	1.28	0.0009	781	1328	24.3	0.47	1.34	563
25.5	0.4618	0.2964	0.7112	0.1459	0.0951	97.4	1304	63.9	3.85	0.0060	1402	0	1215	68.5	0.60	0.83	490	13	1511	62.5	12.47	0.0000	908	1433	52.3	1.21	0.20	641
25.5	0.4312	0.2986	0.7874	0.1476	0.0966	94.7	1253	54.4	1.02	0.0040	1213	44	1162	54.0	0.33	1.44	435	63	1425	46.6	1.16	0.0121	736	1329	34.0	0.43	2.07	493
25.5	0.4430	0.2988	0.7194	0.1472	0.0970	101.0	1262	58.3	2.12	0.0060	1241	25	1179	41.3	0.45	0.99	430	34	1451	43.6	4.16	0.0027	782	1368	35.6	0.76	0.52	545
25.5	0.4629	0.2979	0.7200	0.1471	0.0969	100.6	1289	90.3	6.24	0.0050	1322	0	1208	55.8	0.74	0.73	499	6	1495	54.7	35.71	0.0000	885	1424	50.5	1.76	0.09	686
25.5	0.4476	0.2970	0.7932	0.1456	0.0978	96.3	1290	66.7	2.64	0.0070	1343	21	1207	60.6	0.54	0.93	457	15	1500	81.1	6.84	0.0006	914	1420	54.7	0.98	0.31	661
25.5	0.4471	0.2973	0.7420	0.1452	0.0941	97.8	1280	54.0	1.35	0.0110	1276	29	1185	49.6	0.38	1.35	415	50	1466	47.7	1.98	0.0000	750	1377	37.7	0.57	1.27	559
25.5	0.4439	0.2978	0.7442	0.1471	0.0952	94.9	1267	60.7	1.30	0.0050	1253	24	1176	51.1	0.37	1.21	452	52	1452	55.3	1.81	0.0006	747	1360	40.3	0.53	1.40	507
25.5	0.4691	0.2968	0.8810	0.1475	0.0951	97.3	1296	17.7	2.81	0.0040	1385	0	1216	65.1	0.60	0.91	524	10	1502	76.4	10.50	0.0000	948	1426	59.0	1.20	0.19	726
25.5	0.4367	0.2976	0.7464	0.1471	0.0967	97.9	1275	66.4	1.57	0.0080	1232	35	1179	48.9	0.40	1.11	437	46	1457	47.4	2.32	0.0014	764	1368	35.0	0.60	0.94	506
25.5	0.4698	0.2978	0.8464	0.1454	0.0967	100.4	1301	75.5	6.05	0.0040	1413	0	1219	60.4	0.75	0.82	516	6	1509	70.9	36.55	0.0000	1018	1438	56.5	1.84	0.06	735
25.5	0.4373	0.2976	0.7546	0.1467	0.0954	99.7	1272	59.0	1.34	0.0050	1229	12	1174	51.0	0.37	1.35	440	50	1449	39.9	1.75	0.0008	746	1358	34.3	0.53	1.23	527
25.5	0.4679	0.2971	0.8972	0.1479	0.0951	98.9	1297	84.9	3.47	0.0030	1506	0	1210	65.9	0.59	0.88	463	10	1492	71.8	9.29	0.0000	940	1417	53.4	1.14	0.16	756
25.5	0.4536	0.2985	0.7816	0.1472	0.0960	101.5	1273	58.1	2.31	0.0040	1292	0	1188	49.2	0.49	0.87	459	20	1462	47.9	5.50	0.0000	797	1383	38.8	0.88	0.40	614
25.5	0.4495	0.2994	0.7654	0.1472	0.0956	98.4	1260	52.1	1.60	0.0020	1196	0	1173	52.9	0.41	1.11	469	40	1439	56.3	2.56	0.0000	793	1354	39.6	0.64	0.81	545
25.5	0.4360	0.2970	0.8532	0.1463	0.0985	97.6	1284	64.7	2.01	0.0090	1299	11	1196	54.6	0.45	0.88	466	23	1482	53.1	3.44	0.0000	847	1399	39.7	0.75	0.40	630
25.5	0.4569	0.2994	0.8888	0.1471	0.0942	100.2	1263	58.3	1.44	0.0060	1233	15	1178	50.5	0.40	1.08	471	32	1438	43.9	2.26	0.0007	798	1354	37.1	0.63	0.66	588
25.5	0.4502	0.2984	0.8064	0.1473	0.0944	101.5	1266	58.1	1.53	0.0010	1219	0	1177	46.6	0.41	1.00	449	37	1446	39.6	2.37	0.0000	834	1361	32.1	0.63	0.80	528
27	0.4301	0.2999	0.8818	0.1471	0.0959	95.4	1254	78.6	0.87	0.0070	625	79	1157	60.8	0.31	1.46	418	62	1408	56.5	0.93	0.0048	757	1311	44.9	0.40	1.83	554
27	0.4306	0.2983	0.8406	0.1470	0.0953	98.8	1265	69.5	1.00	0.0090	653	32	1165	53.1	0.32	1.52	432	49	1428	51.9	1.11	0.0016	771	1335	42.4	0.43	1.63	498
27	0.4310	0.2999	0.8950	0.1480	0.0962	98.0	1290	124.3	5.19	0.0030	644	0	1211	81.9	0.71	0.69	509	4	1495	152.4	28.95	0.0005	901	1424	78.8	1.72	0.09	713
27	0.4320	0.2996	0.7928	0.1477	0.0943	98.4	1247	69.7	0.84	0.0110	608	84	1148	53.5	0.30	1.68	373	66	1398	49.5	0.86	0.0049	708	1298	37.3	0.37	2.57	498
27	0.4321	0.2994	0.8146	0.1453	0.0988	96.6	1268	88.2	1.55	0.0060	633	14	1183	71.4	0.41	0.72	427	29	1452	80.1	2.35	0.0000	783	1367	60.1	0.63	0.56	609
27	0.4333	0.2997	0.7118	0.1454	0.0951	95.2	1252	77.7	0.93	0.0040	601	85	1158	61.0	0.31	1.54	406	68	1418	68.6	1.03	0.0023	692	1319	47.5	0.39	2.41	467
27	0.4336	0.2963	0.7820	0.1462	0.0964	95.6	1292	86.8	1.43	0.0030	638	0	1194	63.3	0.39	1.23	433	43	1482	96.1	1.96	0.0000	788	1392	59.1	0.59	0.92	585
27	0.4337	0.2985	0.7464	0.1475	0.0946	100.7	1257	68.5	1.07	0.0070	598	15	1160	51.7	0.33	1.41	440	57	1423	57.0	1.28	0.0019	691	1329	37.4	0.45	1.76	461
27	0.4346	0.2968	0.8196	0.1471	0.0979	98.2	1284	77.7	1.95	0.0050	680	27	1196	70.9	0.46	0.98	479	21	1481	80.3	3.44	0.0000	822	1398	57.2	0.74	0.43	624
27	0.4362	0.2998	0.7364	0.1460	0.0964	101.5	1256	77.2	1.41	0.0060	605	39	1168	57.7	0.38	1.07	423	46	1429	57.6	2.04	0.0026	734	1343	42.3	0.57	0.96	514
27	0.4369	0.2982	0.7776	0.1476	0.0958	98.1	1269	85.9	1.34	0.0080	633	16	1174	58.7	0.37	1.22	438	50	1443	60.1	1.75	0.0010	762	1354	49.6	0.54	1.01	487
27	0.4378	0.2992	0.7054	0.1451	0.0982	94.8	1273	112.5	1.89	0.0060	592	0	1189	75.7	0.45	0.84	454	30	1466	93.8	3.34	0.0016	734	1382	78.8	0.73	0.59	605
27	0.4384	0.2978	0.7814	0.1454	0.0953	100.4	1279	75.5	1.38	0.0110	643	59	1181	60.5	0.38	1.16	420	43	1457	67.7	1.95	0.0002	774	1370	45.5	0.57	0.96	545
27	0.4408	0.2996	0.8364	0.1459	0.0969	100.1	1266	86.7	1.58	0.0060	636	0	1179	64.7	0.41	0.96	465	26	1444	68.6	2.51	0.0000	785	1360	50.0	0.65	0.60	619
27	0.4410	0.2989	0.7516	0.1466	0.0984	99.7	1273	83.1	2.55	0.0060	654	10	1189	66.1	0.51	0.90	488	17	1464	71.8	6.00	0.0004	829	1386	60.4	0.93	0.32	602
27	0.4424	0.2983	0.8544	0.1462	0.0986	100.2	1280	85.7	2.49	0.0040	640	0	1198	61.5	0.53	0.83	539	7	1477	88.7	6.02	0.0000	898	1401	61.6	0.98	0.24	720
27	0.4432	0.2998	0.8136	0.1462	0.0970	96.5	1264	89.6	1.62	0.0080	622	0	1181	65.8	0.41	0.98	461	30	1446	91.7	2.49	0.0004	830	1362	60.6	0.65	0.61	601
27	0.4442	0.2996	0.8036	0.1479	0.0973	96.2	1262	100.9	1.69	0.0040	600	35	1180	75.1	0.43	0.85	457	28	1444	90.0	2.88	0.0012	799	1361	73.3	0.70	0.50	607
27	0.4444	0.2963	0.7582	0.1479	0.0970	97.3	1294	99.0	2.79	0.0070	640	0	1205	72.9	0.53	0.94	462	15	1495	99.8	6.07	0.0000	845	1415	63.6	0.94	0.35	614
27	0.4446	0.2987	0.8758	0.1466	0.0987	99.7	1281	85.1	2.62	0.0060	648	0	1197	65.3	0.53	0.86	511	13	1473	89.5	6.36	0.0000	894	1397	67.6	0.97	0.16	708
27	0.4446	0.2990	0.8870	0.1466	0.0952	101.0	1270	80.8	1.40	0.0030	654	0	1178	62.5	0.39	1.06	452	31	1442	61.8	1.99	0.0007	805	1359	44.8	0.60	0.66	622
27	0.4449	0.2997	0.7538	0.1477	0.0962	100.0	1258	83.4	1.76	0.0020	610	0	1173	61.7	0.42	1.25	499	26	1435	77.7	2.99	0.0000	776	1352	52.2	0.68	0.60	558
27	0.4453	0.2989	0.8254	0.1453	0.0970	96.0	1278	89.7	1.81	0.0060	623	0	1192	80.6	0.45	0.84	482	23	1467	89.8	2.99	0.0022	805	1385	69.2	0.74	0.47	598
27	0.4458	0.2963	0.7064	0.1453	0.0943	101.6	1295	74.6	1.97	0.0040	659	0	1199	61.5	0.45	1.07	454	33	1488	61.6	3.80	0.0003	781	1403	50.7	0.74	0.59	594

Continuation of Table S1.

c_2	q_H	σ_{OO}	ϵ_{OO}	d_{OO}	d_{OH}	a_{HOO}	ρ	ϵ	μ	P_v	λ	wt%$_v$	ρ	ϵ	μ	P_v	λ	wt%$_v$	ρ	ϵ	μ	P_v	λ	ρ	ϵ	μ	P_v	λ
27	0.4460	0.2970	0.7152	0.1473	0.0952	94.7	1289	116.3	1.76	0.0040	644	20	1193	73.4	0.43	0.93	452	36	1480	108.7	2.86	0.0014	770	1393	74.3	0.68	0.75	609
27	0.4460	0.2999	0.8910	0.1458	0.0954	101.4	1265	70.1	1.39	0.0040	651	0	1176	56.9	0.39	1.12	485	36	1435	56.2	2.04	0.0004	825	1351	49.7	0.60	0.57	589
27	0.4468	0.2962	0.7376	0.1452	0.0986	96.9	1308	118.6	4.87	0.0350	646	0	1225	86.0	0.70	0.88	486	7	1531	118.7	22.70	0.0000	898	1457	89.2	1.50	0.10	715
27	0.4471	0.2965	0.8316	0.1473	0.0952	97.1	1293	76.7	1.75	0.0050	712	19	1201	68.7	0.44	1.05	441	31	1484	82.1	2.96	0.0000	804	1400	59.9	0.71	0.56	639
27	0.4478	0.2997	0.7742	0.1458	0.0967	98.1	1268	97.5	1.90	0.0040	611	31	1186	68.3	0.46	0.86	488	23	1454	79.7	3.57	0.0000	829	1373	65.8	0.76	0.41	601
27	0.4482	0.2990	0.7198	0.1460	0.0977	98.6	1278	101.6	3.13	0.0060	643	0	1196	77.7	0.55	0.75	488	16	1474	93.5	8.70	0.0004	832	1398	74.4	1.08	0.29	609
27	0.4484	0.2995	0.8290	0.1472	0.0965	101.4	1266	77.9	1.99	0.0080	639	0	1182	56.5	0.46	0.83	463	22	1446	58.2	3.88	0.0000	848	1367	53.2	0.78	0.33	631
27	0.4486	0.2962	0.7406	0.1470	0.0948	98.0	1296	101.5	2.17	0.0080	674	0	1201	70.2	0.46	1.12	446	26	1490	84.2	3.87	0.0024	809	1407	57.3	0.78	0.49	609
27	0.4510	0.2985	0.8696	0.1466	0.0980	95.5	1286	109.5	2.65	0.0030	643	0	1204	79.3	0.54	0.93	461	14	1483	130.6	5.95	0.0000	895	1408	91.2	1.00	0.17	731
27	0.4516	0.2973	0.8820	0.1476	0.0957	100.9	1290	88.4	2.28	0.0020	684	0	1200	65.9	0.50	0.88	468	16	1477	74.0	4.67	0.0000	883	1399	56.1	0.87	0.27	683
27	0.4520	0.2985	0.7060	0.1464	0.0982	98.6	1285	99.2	4.26	0.0100	630	0	1206	84.0	0.67	0.85	496	8	1490	81.3	20.90	0.0000	894	1417	77.5	1.49	0.11	642
27	0.4532	0.2986	0.8062	0.1469	0.0941	100.6	1274	83.9	1.62	0.0070	635	0	1183	62.4	0.42	1.08	444	33	1451	72.8	2.71	0.0000	812	1369	56.1	0.67	0.66	605
27	0.4532	0.2987	0.7980	0.1462	0.0948	99.7	1276	84.0	1.83	0.0060	661	29	1188	64.9	0.44	0.95	430	27	1460	80.1	3.33	0.0000	804	1378	54.1	0.72	0.46	664
27	0.4536	0.2986	0.7950	0.1473	0.0941	95.2	1290	90.7	2.29	0.0040	700	0	1199	69.9	0.49	0.93	507	16	1477	68.6	4.50	0.0000	913	1399	59.6	0.87	0.24	643
27	0.4538	0.2968	0.8126	0.1453	0.0947	99.6	1299	99.7	2.25	0.0060	709	0	1207	73.4	0.48	1.05	498	24	1494	76.7	4.23	0.0000	842	1414	64.9	0.85	0.30	648
27	0.4538	0.2979	0.8350	0.1456	0.0957	95.0	1292	107.5	2.04	0.0040	654	0	1203	85.0	0.47	0.90	469	20	1486	115.6	3.65	0.0000	879	1405	81.6	0.82	0.38	621
27	0.4540	0.2966	0.7472	0.1466	0.0940	101.5	1294	78.0	2.39	0.0060	699	8	1202	61.8	0.49	0.99	475	18	1487	75.3	4.96	0.0007	815	1405	48.1	0.84	0.50	601
27	0.4548	0.2965	0.7918	0.1459	0.0986	95.5	1311	108.1	5.92	0.0070	681	0	1229	107.1	0.76	0.75	535	4	1532	104.8	28.40	0.0000	973	1461	117.4	1.78	0.07	761
27	0.4548	0.2988	0.8034	0.1473	0.0942	98.9	1273	97.6	1.62	0.0080	622	0	1183	68.9	0.42	1.03	413	31	1451	71.4	2.73	0.0002	803	1368	60.1	0.69	0.65	615
27	0.4586	0.2984	0.8814	0.1462	0.0975	101.9	1288	79.2	4.31	0.0040	650	0	1209	77.9	0.65	0.88	503	6	1489	88.9	17.71	0.0000	963	1417	65.0	1.41	0.08	793
27	0.4592	0.2961	0.7156	0.1451	0.0962	101.6	1312	115.2	6.96	0.0050	678	0	1229	76.6	0.78	0.78	543	7	1528	126.4	43.20	0.0000	895	1456	72.2	1.89	0.07	684
27	0.4592	0.2987	0.7698	0.1457	0.0979	99.6	1291	102.6	5.28	0.0040	640	0	1211	80.4	0.72	0.68	540	5	1495	129.2	29.31	0.0000	874	1425	84.1	1.72	0.09	754
27	0.4594	0.2963	0.8120	0.1451	0.0978	101.9	1314	86.8	8.65	0.0040	696	0	1234	74.9	0.85	0.92	510	3	1531	51.6	74.64	0.0000	981	1463	85.8	2.27	0.03	779
27	0.4594	0.2977	0.8796	0.1478	0.0980	100.7	1294	110.4	5.34	0.0060	704	0	1212	71.7	0.72	0.85	490	3	1496	71.7	25.76	0.0000	928	1427	74.3	1.70	0.06	753
27	0.4598	0.2991	0.7416	0.1473	0.0950	96.6	1275	117.8	2.14	0.0060	626	0	1191	74.4	0.50	0.85	508	21	1463	99.8	4.93	0.0000	849	1384	85.1	0.88	0.34	637
27	0.4600	0.2976	0.8178	0.1475	0.0955	95.5	1292	111.5	2.60	0.0050	666	16	1205	91.8	0.53	0.92	465	15	1488	149.3	5.94	0.0000	877	1411	87.2	0.99	0.22	639
27	0.4604	0.2966	0.8754	0.1455	0.0965	96.7	1310	153.0	3.87	0.0020	721	0	1226	86.6	0.63	0.86	505	5	1521	101.5	11.35	0.0000	937	1448	96.7	1.32	0.10	759
27	0.4614	0.2980	0.8638	0.1463	0.0977	99.4	1297	96.7	4.96	0.0070	696	0	1217	88.3	0.70	0.79	486	4	1502	95.8	25.01	0.0000	953	1432	84.2	1.64	0.06	730
27	0.4615	0.2998	0.8248	0.1461	0.0969	100.2	1275	89.9	3.21	0.0070	669	0	1199	78.6	0.60	0.76	463	10	1469	75.1	12.22	0.0000	916	1397	75.1	1.24	0.12	693
27	0.4621	0.2967	0.8124	0.1455	0.0961	101.8	1304	110.0	5.57	0.0050	691	0	1224	74.7	0.72	0.98	504	5	1514	80.5	23.76	0.0000	973	1443	66.2	1.60	0.09	742
27	0.4626	0.2987	0.8436	0.1457	0.0951	95.1	1286	98.4	2.18	0.0090	678	0	1202	83.1	0.50	0.95	486	14	1481	140.5	4.29	0.0000	888	1402	90.8	0.88	0.30	702
27	0.4641	0.2964	0.8810	0.1463	0.0970	100.8	1311	103.0	7.18	0.0060	750	0	1232	85.7	0.79	0.85	520	4	1525	71.7	39.85	0.0000	1010	1455	79.8	1.91	0.05	771
27	0.4645	0.2993	0.8294	0.1472	0.0940	96.7	1277	123.0	1.94	0.0080	653	0	1189	83.5	0.45	0.91	430	23	1456	113.7	3.38	0.0000	873	1376	74.1	0.77	0.38	623
27	0.4651	0.2979	0.8814	0.1451	0.0969	99.3	1303	107.2	4.83	0.0020	734	0	1222	91.2	0.70	0.80	473	10	1510	100.5	22.98	0.0003	976	1439	94.3	1.56	0.05	765
27	0.4652	0.2987	0.7692	0.1468	0.0961	97.5	1287	109.4	4.08	0.0050	668	0	1207	87.1	0.63	0.83	541	8	1486	135.5	15.52	0.0000	872	1414	96.4	1.33	0.15	697
27	0.4657	0.2971	0.8744	0.1454	0.0944	100.3	1304	86.8	3.28	0.0070	735	0	1218	75.2	0.58	0.99	490	9	1502	123.0	8.24	0.0000	938	1427	78.8	1.11	0.14	750
27	0.4659	0.2991	0.7316	0.1474	0.0968	94.3	1286	108.8	3.98	0.0020	662	0	1209	109.6	0.67	0.78	504	9	1489	152.9	17.76	0.0000	875	1418	135.3	1.52	0.11	702
27	0.4662	0.2979	0.7730	0.1479	0.0988	100.5	1300	79.9	14.50	0.0030	705	0	1222	98.6	1.08	0.71	504	0	1510	17.6	488.96	0.0000	917	1447	87.7	3.60	0.03	781
27	0.4664	0.2961	0.7014	0.1460	0.0958	98.0	1318	146.7	7.86	0.0040	714	0	1235	97.1	0.86	0.93	490	4	1538	52.9	65.04	0.0000	933	1466	120.6	2.12	0.06	748
27	0.4671	0.3000	0.7780	0.1473	0.0975	95.3	1281	144.2	4.02	0.0030	659	0	1206	98.3	0.67	0.76	515	6	1478	218.9	18.98	0.0005	863	1410	115.9	1.57	0.08	698
27	0.4680	0.2965	0.8040	0.1476	0.0990	99.9	1314	92.3	18.34	0.0060	679	0	1237	99.5	1.24	0.87	511	1	1535	13.7	577.57	0.0000	908	1474	102.7	4.68	0.03	871
27	0.4691	0.2980	0.8932	0.1456	0.0967	101.2	1300	111.4	6.52	0.0090	708	0	1221	84.1	0.77	0.82	505	3	1506	119.5	45.39	0.0000	932	1437	82.6	1.81	0.06	782
27	0.4399	0.2995	0.7800	0.1452	0.0962	100.0	1265	75.7	1.45	0.0050	1270	57	1176	55.3	0.39	1.01	472	37	1442	70.6	2.12	0.0000	768	1355	44.4	0.59	0.83	576
27	0.4498	0.2987	0.8780	0.1464	0.0950	99.9	1277	97.0	1.63	0.0080	1305	57	1186	63.6	0.41	1.09	488	28	1453	73.1	2.46	0.0000	856	1371	52.1	0.65	0.49	606
27	0.4664	0.2988	0.7508	0.1479	0.0958	99.5	1283	105.7	4.50	0.0220	1262	0	1202	79.3	0.65	0.82	525	12	1478	91.8	19.60	0.0000	867	1406	85.3	1.45	0.10	715
27	0.4675	0.2993	0.7726	0.1470	0.0984	97.6	1290	114.0	7.49	0.0040	1294	0	1215	91.1	0.86	0.79	503	3	1494	72.8	106.89	0.0000	1022	1430	103.2	2.43	0.03	782
27	0.4305	0.2970	0.7352	0.1480	0.0979	98.4	1277	75.4	1.85	0.0060	1255	16	1186	63.5	0.44	1.00	476	33	1468	64.0	3.16	0.0007	760	1382	59.8	0.69	0.73	571
27	0.4336	0.2974	0.7426	0.1479	0.0957	101.6	1271	72.8	1.45	0.0060	1251	54	1174	55.9	0.38	1.20	475	43	1446	59.5	1.97	0.0000	755	1358	39.9	0.56	1.01	518
27	0.4466	0.2965	0.7750	0.1463	0.0944	101.7	1292	73.7	1.93	0.0020	1430	34	1196	58.8	0.44	1.20	409	37	1481	58.2	3.19	0.0006	808	1397	47.8	0.72	0.60	615
27	0.4590	0.2982	0.7896	0.1465	0.0964	95.0	1291	125.3	2.89	0.0040	1258	0	1207	90.2	0.56	0.88	533	3	1491	131.4	6.87	0.0000	875	1415	103.5	1.06	0.21	672
27	0.4598	0.2989	0.8682	0.1469	0.0943	98.3	1279	101.7	1.82	0.0070	1403	0	1189	70.8	0.45	0.96	481	24	1458	81.2	3.30	0.0000	874	1378	69.8	0.74	0.40	657

Continuation of Table S1.

c_2	q_H	σ_{OO}	ϵ_{OO}	d_{OO}	d_{OH}	a_{HOO}	ρ	ϵ	μ	P_v	λ	wt%$_v$	ρ	ϵ	μ	P_v	λ	wt%$_v$	ρ	ϵ	μ	P_v	λ	ρ	ϵ	μ	P_v	λ
27	0.4355	0.2976	0.8538	0.1455	0.0961	101.9	1282	76.8	1.42	0.0110	1321	13	1187	51.3	0.40	1.08	422	39	1462	55.2	2.08	0.0005	822	1376	42.5	0.60	0.62	613
27	0.4326	0.2996	0.8842	0.1465	0.0981	96.1	1264	90.5	1.30	0.0070	1306	48	1177	64.7	0.38	1.14	475	40	1437	83.1	1.69	0.0003	805	1351	53.2	0.55	0.79	642
27	0.4435	0.2979	0.7546	0.1452	0.0945	97.1	1281	85.2	1.32	0.0080	1289	45	1184	64.7	0.38	1.10	459	48	1462	78.7	1.82	0.0006	736	1372	53.1	0.55	1.01	558
27	0.4390	0.2960	0.7730	0.1479	0.0980	98.7	1293	89.5	2.74	0.0050	1312	0	1206	66.2	0.55	0.97	454	15	1498	76.6	6.73	0.0000	848	1419	67.1	1.00	0.24	689
27	0.4331	0.2972	0.7702	0.1471	0.0947	96.6	1273	79.9	1.06	0.0090	1232	48	1172	57.9	0.33	1.50	412	60	1446	63.6	1.22	0.0096	740	1350	44.5	0.45	1.93	467
27	0.4475	0.2996	0.8704	0.1471	0.0965	97.2	1267	101.7	1.63	0.0050	1264	0	1182	75.1	0.42	0.98	474	30	1445	87.1	2.53	0.0006	848	1363	61.2	0.69	0.42	654
27	0.4322	0.2968	0.8148	0.1478	0.0988	98.1	1283	82.6	2.20	0.0090	1269	0	1196	66.1	0.48	1.06	463	23	1481	79.2	3.89	0.0000	826	1399	56.5	0.79	0.38	644
27	0.4665	0.2989	0.8452	0.1452	0.0972	97.3	1293	101.5	4.41	0.0040	1302	0	1217	101.4	0.70	0.77	514	6	1500	102.2	23.88	0.0000	933	1429	105.7	1.53	0.08	773
27	0.4500	0.2998	0.8592	0.1471	0.0982	99.9	1269	89.2	2.64	0.0080	1246	0	1191	66.0	0.54	0.76	514	10	1457	79.8	6.66	0.0000	867	1382	66.6	0.99	0.19	700
27	0.4632	0.2999	0.8788	0.1468	0.0971	94.3	1280	141.3	2.73	0.0030	1342	0	1202	95.2	0.56	0.82	499	7	1472	183.4	7.03	0.0000	946	1398	124.3	1.07	0.13	735
27	0.4429	0.2999	0.8100	0.1453	0.0959	100.3	1262	70.4	1.40	0.0110	1205	0	1174	59.6	0.39	0.99	449	42	1436	62.6	2.10	0.0004	792	1351	47.8	0.59	0.77	583
27	0.4512	0.2992	0.8760	0.1477	0.0972	96.7	1273	90.7	2.15	0.0070	1241	0	1192	78.7	0.49	0.86	481	12	1459	121.2	4.11	0.0000	872	1382	67.9	0.85	0.27	694
27	0.4458	0.2975	0.7300	0.1457	0.0969	101.1	1288	76.7	2.96	0.0080	1287	7	1201	66.7	0.55	0.86	474	14	1487	67.3	7.37	0.0000	854	1410	59.8	1.02	0.25	621
27	0.4451	0.2975	0.8238	0.1468	0.0974	96.0	1289	95.4	2.26	0.0030	1304	0	1201	85.0	0.49	0.86	488	24	1485	115.4	4.27	0.0009	870	1405	69.1	0.85	0.29	628
27	0.4424	0.2996	0.7914	0.1461	0.0960	95.4	1264	92.7	1.30	0.0070	1265	12	1176	72.4	0.37	1.16	439	44	1440	104.8	1.79	0.0016	808	1352	60.9	0.54	0.99	546
27	0.4459	0.2971	0.7814	0.1469	0.0979	99.5	1291	82.7	3.28	0.0060	1267	0	1206	72.5	0.59	0.95	491	12	1494	74.1	10.19	0.0000	894	1419	67.7	1.15	0.20	670
27	0.4440	0.2991	0.7644	0.1468	0.0957	98.0	1267	93.4	1.52	0.0030	1249	0	1177	64.5	0.40	1.03	471	47	1445	75.7	2.26	0.0003	783	1359	58.4	0.61	0.71	609
27	0.4624	0.2999	0.8330	0.1458	0.0950	97.8	1277	103.8	2.17	0.0050	1279	0	1192	85.3	0.48	0.87	494	22	1459	102.7	4.20	0.0000	858	1381	78.2	0.84	0.35	674
27	0.4507	0.2995	0.7184	0.1452	0.0973	100.5	1274	99.0	3.22	0.0100	1314	19	1194	71.9	0.57	0.89	512	14	1468	79.4	9.46	0.0000	798	1393	66.9	1.09	0.25	635
27	0.4312	0.2967	0.7744	0.1471	0.0956	94.1	1280	86.9	1.11	0.0080	1283	35	1179	67.6	0.34	1.37	462	58	1459	76.7	1.29	0.0022	744	1362	53.6	0.47	1.73	506
27	0.4558	0.2971	0.8276	0.1479	0.0988	96.7	1303	99.5	5.78	0.0040	1347	0	1220	97.6	0.76	0.78	531	3	1512	148.3	30.60	0.0000	934	1442	101.4	1.76	0.06	782
27	0.4593	0.2965	0.7970	0.1462	0.0947	95.8	1305	124.3	2.35	0.0060	1388	0	1218	86.3	0.53	0.91	586	27	1507	121.9	5.45	0.0000	880	1427	82.1	0.94	0.27	654
27	0.4677	0.3000	0.8632	0.1474	0.0978	96.4	1281	108.7	4.08	0.0050	1364	0	1207	91.6	0.68	0.69	506	5	1478	99.9	21.37	0.0000	970	1408	98.9	1.56	0.07	789
27	0.4631	0.2993	0.7150	0.1454	0.0988	99.8	1294	136.1	9.18	0.0030	1339	0	1217	92.4	0.97	0.81	516	3	1502	39.6	152.11	0.0000	946	1436	95.0	2.78	0.03	769
27	0.4585	0.2992	0.8526	0.1464	0.0959	99.7	1278	109.8	2.35	0.0050	1312	0	1195	73.3	0.51	0.89	448	16	1465	97.0	5.71	0.0016	889	1389	68.4	0.93	0.23	687
27	0.4648	0.2976	0.7706	0.1472	0.0979	95.7	1302	182.8	6.59	0.0070	1296	0	1225	120.4	0.84	0.83	517	3	1519	102.6	48.70	0.0000	960	1450	110.6	2.20	0.05	778
27	0.4605	0.2988	0.7786	0.1460	0.0989	100.0	1290	78.3	6.99	0.0130	1350	0	1215	85.2	0.85	0.74	524	3	1499	103.9	82.88	0.0000	978	1432	95.0	2.29	0.05	749
27	0.4557	0.2961	0.8252	0.1466	0.0979	98.2	1312	89.8	5.94	0.0040	1400	0	1229	94.1	0.74	0.90	468	2	1528	63.2	27.59	0.0000	967	1456	100.2	1.73	0.09	757
27	0.4318	0.2987	0.8888	0.1473	0.0952	95.9	1264	80.0	0.91	0.0120	1287	25	1164	58.3	0.32	1.57	371	61	1422	60.1	0.96	0.0030	731	1326	41.4	0.41	1.79	534
27	0.4692	0.2977	0.7622	0.1450	0.0944	95.3	1305	128.8	3.14	0.0030	1368	0	1217	94.4	0.59	0.95	484	11	1509	141.2	8.99	0.0000	872	1433	115.3	1.18	0.18	663
27	0.4374	0.2993	0.7992	0.1454	0.0989	96.0	1273	90.6	1.93	0.0030	1249	21	1190	71.1	0.46	0.81	473	23	1463	92.4	3.39	0.0000	804	1382	70.1	0.76	0.39	604
27	0.4389	0.2967	0.7656	0.1471	0.0984	98.1	1293	80.0	2.74	0.0070	1361	0	1204	79.2	0.54	0.79	438	15	1494	78.1	6.45	0.0000	873	1416	60.9	1.02	0.23	652
27	0.4493	0.2969	0.7082	0.1451	0.0970	98.2	1299	96.6	3.82	0.0090	1273	0	1214	76.9	0.60	0.90	446	13	1510	109.0	11.90	0.0000	869	1434	82.4	1.20	0.19	647
27	0.4587	0.2990	0.8252	0.1477	0.0955	100.9	1276	100.4	2.56	0.0060	1324	0	1190	65.5	0.51	0.85	467	16	1459	77.0	5.98	0.0002	849	1383	60.9	0.94	0.23	676
27	0.4527	0.2994	0.7998	0.1454	0.0964	94.4	1277	115.5	1.90	0.0020	1250	0	1194	87.7	0.46	0.88	470	26	1468	115.5	3.42	0.0000	852	1387	85.1	0.78	0.32	631
27	0.4585	0.2969	0.8310	0.1471	0.0985	100.0	1305	100.7	7.15	0.0100	1418	0	1223	78.9	0.81	0.82	507	5	1518	85.1	56.97	0.0000	990	1449	88.9	2.18	0.03	806
27	0.4488	0.2972	0.8408	0.1454	0.0940	97.8	1289	82.4	1.44	0.0060	1361	0	1195	65.2	0.40	1.17	478	34	1474	80.4	2.02	0.0012	855	1388	54.6	0.61	0.75	628
27	0.4439	0.2991	0.8708	0.1476	0.0943	100.9	1262	73.9	1.18	0.0080	1300	30	1169	56.1	0.36	1.20	486	41	1428	60.9	1.53	0.0017	769	1340	42.3	0.52	0.97	536
27	0.4454	0.2987	0.8302	0.1469	0.0964	94.1	1277	97.5	1.52	0.0030	1287	0	1186	83.9	0.42	1.10	418	50	1458	101.0	2.27	0.0010	793	1373	73.9	0.63	0.64	561
27	0.4538	0.2974	0.7654	0.1453	0.0970	100.3	1299	95.9	4.14	0.0100	1292	0	1213	69.1	0.63	0.83	485	8	1503	78.4	15.85	0.0000	931	1430	68.6	1.33	0.13	698
27	0.4520	0.2984	0.8430	0.1463	0.0958	99.6	1280	83.2	2.03	0.0010	1324	64	1194	67.9	0.48	0.90	495	16	1469	79.5	4.04	0.0000	851	1389	66.4	0.82	0.34	670
27	0.4449	0.2968	0.7270	0.1479	0.0972	100.0	1291	97.7	3.27	0.0030	1247	0	1201	68.0	0.57	0.83	492	8	1488	83.0	8.83	0.0000	840	1410	65.5	1.06	0.23	648
27	0.4423	0.2976	0.8070	0.1466	0.0969	96.0	1285	100.0	1.89	0.0020	1292	0	1195	73.6	0.45	0.90	515	27	1477	92.6	3.12	0.0005	828	1394	63.5	0.73	0.46	631
27	0.4394	0.2969	0.8124	0.1450	0.0986	98.8	1299	84.9	2.85	0.0030	1326	0	1210	72.2	0.54	0.74	487	12	1503	93.4	6.55	0.0012	886	1425	66.5	1.00	0.23	713
27	0.4308	0.2966	0.8386	0.1454	0.0947	99.8	1283	70.9	1.07	0.0060	1395	26	1184	56.8	0.34	1.47	446	52	1461	55.2	1.26	0.0009	779	1366	39.7	0.47	1.48	539
27	0.4433	0.2999	0.7798	0.1464	0.0977	101.8	1263	83.6	2.07	0.0030	1242	0	1181	62.1	0.48	1.23	479	14	1446	71.6	4.64	0.0000	860	1367	48.6	0.82	0.45	623
27	0.4568	0.2994	0.8260	0.1456	0.0950	95.5	1276	105.6	1.67	0.0050	1320	0	1190	80.2	0.43	0.90	497	24	1460	119.2	2.76	0.0008	878	1378	81.8	0.71	0.48	629
27	0.4312	0.2985	0.7968	0.1454	0.0972	99.4	1272	71.3	1.37	0.0090	1242	36	1179	58.1	0.38	1.01	451	37	1451	65.4	1.91	0.0003	767	1363	50.1	0.57	0.83	542
27	0.4423	0.2984	0.7570	0.1468	0.0948	94.6	1271	103.9	1.22	0.0030	1205	36	1175	72.5	0.36	1.41	430	52	1446	81.6	1.50	0.0011	731	1354	61.8	0.51	1.43	539
27	0.4629	0.2971	0.7766	0.1471	0.0942	97.0	1298	114.0	2.62	0.0050	1392	0	1208	73.9	0.53	0.97	467	21	1493	124.6	6.06	0.0000	852	1414	83.0	0.96	0.28	666

Continuation of Table S1.

c_2	q_H	σ_{OO}	ϵ_{OO}	d_{OO}	d_{OH}	a_{HOO}	ρ	ϵ	μ	P_v	λ	wt$\%_v$	ρ	ϵ	μ	P_v	λ	wt$\%_v$	ρ	ϵ	μ	P_v	λ	ρ	ϵ	μ	P_v	λ
28.5	0.4304	0.2960	0.7184	0.1455	0.0975	100.4	1292	69.4	2.08	0.0060	626	0	1199	56.3	0.46	1.09	457	27	1492	61.5	3.77	0.0000	786	1407	44.7	0.75	0.53	630
28.5	0.4307	0.2990	0.7332	0.1470	0.0944	99.7	1250	67.6	0.90	0.0080	567	63	1151	46.2	0.30	1.67	429	68	1408	44.3	0.96	0.0066	671	1310	33.4	0.39	2.68	427
28.5	0.4309	0.2972	0.7974	0.1472	0.0958	97.1	1275	80.5	1.11	0.0100	601	40	1174	55.2	0.35	1.35	401	52	1449	59.7	1.38	0.0023	758	1355	40.5	0.47	1.45	506
28.5	0.4334	0.2997	0.7042	0.1478	0.0944	94.4	1242	71.9	0.84	0.0100	587	75	1146	58.5	0.29	1.98	397	75	1398	60.1	0.85	0.0070	684	1296	44.3	0.36	3.62	410
28.5	0.4336	0.2963	0.7710	0.1469	0.0988	95.7	1295	85.9	2.29	0.0060	652	0	1203	68.7	0.49	0.96	473	24	1497	83.7	4.30	0.0000	838	1416	63.1	0.85	0.43	629
28.5	0.4336	0.2963	0.8700	0.1466	0.0942	98.0	1282	71.3	0.99	0.0090	700	0	1186	54.0	0.34	1.42	428	55	1458	56.3	1.14	0.0013	814	1363	36.6	0.45	1.55	569
28.5	0.4341	0.2972	0.7400	0.1477	0.0965	98.3	1277	70.5	1.56	0.0050	605	13	1181	53.7	0.40	1.19	437	42	1460	62.8	2.32	0.0015	786	1372	45.8	0.60	0.93	536
28.5	0.4345	0.2961	0.7480	0.1470	0.0940	96.8	1282	80.3	1.08	0.0060	621	34	1177	46.1	0.34	1.51	402	61	1459	59.7	1.26	0.0031	739	1362	43.0	0.45	1.96	504
28.5	0.4352	0.2992	0.7872	0.1478	0.0951	94.1	1255	73.6	0.93	0.0080	610	51	1158	58.4	0.31	1.64	397	65	1415	65.2	1.02	0.0013	719	1318	48.8	0.40	2.26	474
28.5	0.4353	0.2982	0.8684	0.1451	0.0950	94.0	1274	78.0	0.97	0.0070	661	72	1176	57.6	0.33	1.51	432	56	1444	65.5	1.05	0.0037	776	1349	48.8	0.43	1.76	522
28.5	0.4360	0.2997	0.7126	0.1458	0.0945	101.9	1249	63.8	1.07	0.0060	615	23	1157	48.2	0.33	1.39	410	59	1413	42.2	1.28	0.0020	728	1319	34.0	0.44	1.98	462
28.5	0.4362	0.2996	0.7226	0.1458	0.0967	97.4	1258	74.4	1.38	0.0040	602	0	1171	58.6	0.38	1.14	425	49	1436	61.5	1.93	0.0025	753	1347	46.2	0.55	1.17	512
28.5	0.4364	0.2963	0.8704	0.1478	0.0984	94.3	1296	104.8	1.93	0.0040	704	17	1202	67.0	0.47	1.03	488	23	1490	91.1	3.15	0.0000	863	1408	67.0	0.75	0.36	620
28.5	0.4364	0.2973	0.7834	0.1467	0.0951	95.7	1278	80.0	1.15	0.0090	641	82	1178	64.9	0.35	1.41	428	53	1454	64.3	1.40	0.0005	739	1361	52.3	0.49	1.43	498
28.5	0.4367	0.2994	0.8164	0.1471	0.0976	97.9	1260	73.6	1.48	0.0080	653	34	1176	61.1	0.40	0.90	472	38	1440	62.0	2.22	0.0004	786	1355	52.5	0.61	0.74	568
28.5	0.4368	0.2990	0.8944	0.1457	0.0981	99.1	1273	79.3	1.59	0.0080	628	29	1187	57.1	0.43	0.97	487	26	1455	73.5	2.60	0.0000	889	1373	47.3	0.67	0.39	613
28.5	0.4380	0.2965	0.7038	0.1477	0.0977	97.1	1291	97.3	2.51	0.0050	629	17	1199	68.1	0.51	0.91	501	22	1490	89.2	5.43	0.0009	780	1409	63.6	0.91	0.42	596
28.5	0.4381	0.2995	0.7236	0.1457	0.0962	98.2	1261	78.8	1.34	0.0050	610	13	1171	52.6	0.38	1.07	421	47	1436	61.9	1.87	0.0015	727	1348	44.0	0.56	0.94	517
28.5	0.4382	0.2973	0.7824	0.1479	0.0982	94.4	1283	94.7	2.01	0.0050	618	0	1194	73.0	0.47	0.99	445	29	1478	109.0	3.40	0.0000	801	1395	62.3	0.78	0.44	616
28.5	0.4382	0.2984	0.7528	0.1455	0.0964	97.7	1273	84.3	1.44	0.0060	605	14	1182	62.3	0.39	1.12	463	41	1457	66.2	2.20	0.0000	757	1371	50.4	0.60	0.84	542
28.5	0.4388	0.2963	0.8540	0.1464	0.0950	100.2	1289	77.7	1.42	0.0060	717	33	1196	56.8	0.40	1.15	431	33	1475	50.2	2.00	0.0000	839	1387	41.7	0.58	0.71	593
28.5	0.4392	0.2990	0.8850	0.1454	0.0963	97.8	1274	76.1	1.27	0.0060	654	18	1181	54.4	0.38	1.08	444	36	1447	70.0	1.73	0.0000	840	1361	50.2	0.56	0.76	598
28.5	0.4399	0.2987	0.7730	0.1479	0.0986	97.1	1270	93.9	2.08	0.0050	626	0	1186	64.5	0.49	0.86	473	19	1459	79.6	4.55	0.0000	822	1379	64.0	0.82	0.44	616
28.5	0.4402	0.2993	0.7252	0.1471	0.0959	100.3	1256	62.6	1.49	0.0050	606	0	1170	53.9	0.39	1.05	470	43	1433	55.2	2.29	0.0007	758	1346	42.7	0.60	0.97	562
28.5	0.4406	0.2982	0.8756	0.1475	0.0974	95.5	1278	94.0	1.60	0.0040	650	8	1188	65.5	0.42	1.01	488	28	1461	81.5	2.50	0.0002	834	1377	60.6	0.66	0.49	632
28.5	0.4412	0.2978	0.7270	0.1453	0.0967	96.3	1286	107.4	1.84	0.0090	627	0	1193	62.7	0.45	1.06	457	34	1478	87.8	3.15	0.0000	792	1393	58.0	0.70	0.72	581
28.5	0.4422	0.2994	0.8708	0.1472	0.0961	100.5	1264	75.3	1.40	0.0040	655	0	1174	58.3	0.39	1.04	463	36	1435	55.2	2.04	0.0000	828	1350	45.7	0.60	0.67	614
28.5	0.4423	0.2984	0.7568	0.1467	0.0989	97.4	1280	85.3	2.92	0.0050	609	0	1197	75.1	0.54	0.86	459	14	1478	86.8	7.62	0.0003	816	1401	70.9	1.01	0.19	689
28.5	0.4429	0.2969	0.7706	0.1459	0.0951	95.4	1292	83.2	1.44	0.0020	652	0	1192	70.0	0.40	1.19	469	41	1478	66.1	2.15	0.0000	819	1389	58.6	0.59	0.76	541
28.5	0.4431	0.2974	0.7678	0.1475	0.0973	100.0	1284	83.3	2.53	0.0040	631	0	1194	60.0	0.51	0.92	472	17	1476	64.3	5.68	0.0000	844	1397	53.5	0.91	0.31	651
28.5	0.4431	0.2988	0.8478	0.1462	0.0963	98.7	1274	75.8	1.54	0.0080	625	32	1183	56.1	0.41	1.10	446	30	1451	68.3	2.29	0.0000	803	1367	50.2	0.63	0.62	585
28.5	0.4434	0.2973	0.7106	0.1459	0.0976	100.9	1292	80.0	3.43	0.0080	656	7	1203	67.6	0.58	0.89	456	20	1492	62.2	10.60	0.0007	845	1415	58.4	1.12	0.27	673
28.5	0.4437	0.2970	0.8630	0.1478	0.0975	95.2	1290	89.1	2.10	0.0080	676	11	1201	73.5	0.48	1.00	471	21	1484	84.3	3.81	0.0000	900	1404	70.3	0.81	0.38	637
28.5	0.4448	0.2999	0.7520	0.1466	0.0968	98.2	1262	92.3	1.76	0.0090	610	0	1178	63.3	0.43	0.91	475	31	1442	72.7	3.09	0.0000	796	1359	59.5	0.70	0.61	580
28.5	0.4458	0.2962	0.7248	0.1473	0.0947	97.8	1294	97.1	1.88	0.0060	632	0	1196	65.1	0.44	1.12	440	34	1484	74.6	3.21	0.0003	771	1398	57.5	0.70	0.71	586
28.5	0.4460	0.2982	0.8160	0.1454	0.0949	97.3	1279	85.4	1.37	0.0080	679	8	1185	65.8	0.39	1.17	456	42	1460	69.0	1.98	0.0018	783	1374	55.9	0.59	0.89	565
28.5	0.4470	0.2990	0.7314	0.1452	0.0970	101.0	1276	83.3	2.63	0.0050	640	0	1192	64.8	0.52	0.80	439	22	1468	63.8	6.44	0.0000	793	1389	54.5	0.93	0.28	607
28.5	0.4472	0.2968	0.8084	0.1462	0.0977	98.8	1298	107.4	3.29	0.0060	683	0	1210	72.2	0.59	0.90	477	10	1502	87.3	8.63	0.0000	902	1426	65.9	1.11	0.18	644
28.5	0.4477	0.2989	0.8388	0.1458	0.0943	95.2	1273	94.5	1.16	0.0030	635	19	1179	52.4	0.37	1.17	465	46	1446	83.7	1.49	0.0000	821	1356	54.9	0.51	1.03	518
28.5	0.4481	0.2984	0.7326	0.1451	0.0987	98.4	1287	98.4	3.59	0.0030	632	0	1207	78.8	0.63	0.75	511	11	1495	91.4	15.15	0.0000	934	1420	81.9	1.34	0.17	662
28.5	0.4490	0.2960	0.8268	0.1455	0.0941	99.3	1300	78.4	1.81	0.0060	729	19	1208	62.1	0.44	1.12	442	31	1492	75.1	2.72	0.0006	821	1407	55.6	0.69	0.59	599
28.5	0.4493	0.2961	0.8178	0.1465	0.0943	96.0	1299	95.2	1.63	0.0130	709	8	1204	70.3	0.43	1.20	470	33	1490	89.0	2.53	0.0000	826	1403	64.7	0.66	0.72	573
28.5	0.4495	0.2995	0.8946	0.1469	0.0940	99.6	1264	75.4	1.20	0.0090	648	8	1173	51.9	0.37	1.11	464	40	1430	62.1	1.57	0.0011	802	1344	44.8	0.53	0.87	566
28.5	0.4503	0.2970	0.8142	0.1458	0.0975	98.6	1299	95.3	3.41	0.0050	657	41	1211	70.1	0.58	0.92	496	8	1503	103.8	9.89	0.0000	949	1428	71.6	1.17	0.19	694
28.5	0.4503	0.2998	0.8276	0.1452	0.0940	99.0	1265	71.0	1.20	0.0050	652	52	1173	58.1	0.37	1.24	452	46	1434	70.2	1.65	0.0000	769	1346	46.0	0.53	1.00	554
28.5	0.4514	0.2984	0.8430	0.1475	0.0947	101.1	1275	78.1	1.72	0.0140	648	10	1183	56.7	0.43	0.97	459	28	1453	64.4	2.85	0.0004	861	1370	44.9	0.69	0.57	566
28.5	0.4518	0.2988	0.8624	0.1469	0.0990	95.8	1285	110.0	3.38	0.0020	661	0	1205	86.2	0.60	0.76	467	6	1484	89.9	10.32	0.0000	947	1411	89.2	1.20	0.15	734
28.5	0.4518	0.2990	0.7420	0.1477	0.0987	101.4	1276	80.8	5.07	0.0060	680	0	1197	66.1	0.68	0.74	510	7	1473	92.2	27.33	0.0000	848	1401	63.5	1.52	0.11	680
28.5	0.4523	0.2965	0.8582	0.1464	0.0957	100.1	1301	90.0	2.64	0.0050	715	0	1212	63.9	0.53	0.98	489	13	1498	82.0	5.56	0.0000	894	1419	57.6	0.95	0.27	708
28.5	0.4527	0.2991	0.7280	0.1451	0.0981	95.7	1284	105.3	3.28	0.0040	631	0	1205	79.4	0.60	0.73	490	9	1488	149.4	10.35	0.0007	881	1413	78.1	1.22	0.22	685

Continuation of Table S1.

c_2	q_H	σ_{OO}	ϵ_{OO}	d_{OO}	d_{OH}	a_{HOO}	ρ	ϵ	μ	P_v	λ	wt%$_v$	ρ	ϵ	μ	P_v	λ	wt%$_v$	ρ	ϵ	μ	P_v	λ	ρ	ϵ	μ	P_v	λ
28.5	0.4529	0.2971	0.7302	0.1475	0.0953	96.7	1291	83.5	2.43	0.0080	639	14	1199	69.0	0.49	0.97	443	23	1485	102.1	4.93	0.0000	826	1404	69.2	0.87	0.41	637
28.5	0.4530	0.2992	0.7394	0.1455	0.0952	96.8	1272	81.5	1.76	0.0060	644	0	1187	71.7	0.44	0.96	436	30	1459	84.1	3.13	0.0000	816	1376	60.6	0.71	0.53	583
28.5	0.4531	0.2977	0.7536	0.1477	0.0988	98.8	1291	112.5	5.10	0.0030	645	0	1210	78.9	0.75	0.86	524	4	1498	138.3	31.00	0.0000	923	1428	76.0	1.76	0.08	699
28.5	0.4532	0.2983	0.8314	0.1468	0.0989	98.3	1287	92.1	4.28	0.0040	663	0	1208	72.6	0.68	0.77	497	10	1492	91.2	18.42	0.0000	924	1420	75.8	1.46	0.09	753
28.5	0.4532	0.2995	0.8372	0.1480	0.0988	95.0	1274	106.3	2.97	0.0090	653	0	1198	89.4	0.57	0.77	496	11	1469	104.2	8.06	0.0000	906	1395	93.6	1.12	0.17	694
28.5	0.4544	0.2997	0.8544	0.1475	0.0975	94.6	1273	105.2	2.29	0.0090	658	0	1193	74.1	0.50	0.80	480	16	1460	98.8	4.53	0.0000	855	1382	82.2	0.89	0.29	654
28.5	0.4547	0.2976	0.8250	0.1477	0.0961	94.4	1291	97.9	2.21	0.0050	675	0	1202	74.5	0.50	0.98	500	18	1483	118.6	4.33	0.0000	884	1403	85.3	0.86	0.33	648
28.5	0.4548	0.2998	0.8994	0.1473	0.0964	96.0	1271	99.1	1.86	0.0030	652	0	1188	74.1	0.45	0.88	491	19	1451	92.6	3.32	0.0000	880	1371	69.3	0.77	0.38	675
28.5	0.4549	0.2976	0.7150	0.1457	0.0960	94.9	1297	103.4	2.81	0.0070	652	0	1207	75.6	0.54	0.98	504	20	1497	108.7	6.21	0.0000	834	1418	80.7	0.98	0.41	630
28.5	0.4561	0.2987	0.7946	0.1479	0.0984	95.9	1285	103.9	3.90	0.0040	678	0	1204	82.1	0.65	0.81	509	13	1484	105.1	13.66	0.0000	916	1412	93.0	1.37	0.12	702
28.5	0.4566	0.2989	0.7848	0.1476	0.0966	97.0	1276	100.0	2.79	0.0060	631	0	1195	74.2	0.53	0.89	492	23	1468	97.1	6.46	0.0000	838	1392	77.4	1.01	0.25	654
28.5	0.4571	0.2965	0.8678	0.1463	0.0969	100.2	1303	81.6	4.59	0.0020	731	0	1223	77.7	0.67	0.94	486	9	1512	78.8	14.14	0.0000	985	1439	68.7	1.35	0.11	743
28.5	0.4578	0.2975	0.7120	0.1471	0.0988	97.3	1301	122.2	7.73	0.0040	663	0	1221	84.1	0.89	0.85	484	5	1516	109.5	87.40	0.0000	976	1447	93.0	2.37	0.06	709
28.5	0.4586	0.2999	0.7362	0.1452	0.0972	95.1	1280	106.0	2.90	0.0090	653	0	1201	91.2	0.58	0.78	478	11	1476	108.9	8.68	0.0000	858	1401	100.5	1.14	0.30	675
28.5	0.4592	0.2997	0.8560	0.1475	0.0946	102.0	1268	66.6	1.95	0.0050	640	0	1181	56.4	0.46	0.92	460	24	1442	68.0	3.94	0.0000	852	1363	51.5	0.77	0.40	631
28.5	0.4594	0.2979	0.8634	0.1453	0.0963	94.6	1300	121.4	2.62	0.0050	665	0	1213	84.0	0.54	0.92	509	12	1499	118.1	6.15	0.0000	941	1422	96.8	1.00	0.26	758
28.5	0.4599	0.2963	0.8148	0.1457	0.0989	96.4	1316	54.5	8.78	0.0020	705	0	1237	98.3	0.93	0.88	543	2	1544	53.4	107.96	0.0000	1044	1474	114.0	2.41	0.04	834
28.5	0.4611	0.2986	0.7606	0.1470	0.0972	100.7	1283	91.5	5.15	0.0020	644	0	1205	76.6	0.70	0.78	481	7	1484	60.1	29.34	0.0000	866	1414	77.2	1.63	0.10	699
28.5	0.4612	0.2968	0.7068	0.1465	0.0944	98.0	1300	88.4	3.34	0.0050	665	0	1209	82.6	0.56	0.93	481	14	1499	67.7	8.37	0.0000	818	1422	73.3	1.08	0.24	632
28.5	0.4612	0.2996	0.7444	0.1450	0.0947	95.7	1278	108.6	2.07	0.0030	640	0	1192	76.2	0.46	0.85	472	26	1465	97.8	3.69	0.0002	817	1385	80.5	0.80	0.44	595
28.5	0.4613	0.2985	0.7120	0.1469	0.0961	100.8	1284	98.1	4.60	0.0040	630	0	1202	68.0	0.65	0.85	523	9	1483	112.3	20.54	0.0000	848	1409	69.5	1.41	0.14	640
28.5	0.4624	0.2987	0.8444	0.1477	0.0978	100.9	1283	85.9	5.05	0.0090	671	0	1206	61.0	0.70	0.78	523	11	1483	55.7	31.33	0.0000	981	1413	72.1	1.70	0.06	762
28.5	0.4635	0.2998	0.8254	0.1470	0.0960	101.1	1273	87.1	3.00	0.0080	627	0	1193	63.8	0.57	0.85	499	13	1460	83.2	9.42	0.0000	891	1386	62.0	1.14	0.14	680
28.5	0.4647	0.2982	0.8716	0.1478	0.0971	98.5	1292	103.2	4.60	0.0030	653	0	1210	80.0	0.67	0.77	518	8	1490	82.5	19.23	0.0000	949	1419	88.2	1.48	0.08	736
28.5	0.4652	0.2974	0.7062	0.1456	0.0973	94.9	1311	135.8	6.79	0.0050	636	0	1229	105.0	0.85	0.77	513	5	1528	124.4	49.21	0.0000	921	1459	117.6	2.15	0.05	726
28.5	0.4652	0.2997	0.8996	0.1470	0.0970	97.9	1280	111.6	3.35	0.0020	677	0	1202	81.1	0.61	0.76	486	5	1471	89.8	10.99	0.0000	945	1399	78.2	1.24	0.12	739
28.5	0.4655	0.2994	0.7930	0.1451	0.0979	97.0	1291	103.7	4.95	0.0040	628	0	1214	101.6	0.74	0.73	522	4	1495	80.5	30.92	0.0000	909	1427	100.0	1.76	0.07	744
28.5	0.4656	0.2986	0.8052	0.1461	0.0964	95.8	1292	120.8	3.55	0.0050	656	0	1211	84.0	0.62	0.82	521	9	1493	134.4	12.39	0.0000	909	1420	89.0	1.28	0.15	711
28.5	0.4658	0.2968	0.7588	0.1450	0.0988	94.8	1323	146.7	10.79	0.0110	677	0	1244	131.7	1.08	0.89	514	1	1549	111.1	138.48	0.0000	982	1485	143.5	3.17	0.03	835
28.5	0.4668	0.2962	0.7378	0.1457	0.0963	100.8	1315	78.3	9.32	0.0040	719	0	1235	82.9	0.91	0.88	496	5	1533	31.7	85.03	0.0000	953	1464	79.8	2.41	0.06	759
28.5	0.4672	0.2980	0.8412	0.1454	0.0941	99.9	1293	89.6	2.70	0.0060	682	0	1208	70.9	0.55	0.88	488	15	1488	70.0	6.45	0.0000	892	1411	70.3	1.01	0.22	693
28.5	0.4686	0.2971	0.7740	0.1452	0.0981	98.3	1313	108.1	13.85	0.0030	727	0	1236	93.9	1.04	0.84	523	1	1537	23.0	177.71	0.0000	1104	1472	112.2	3.36	0.02	814
28.5	0.4691	0.2979	0.7448	0.1474	0.0978	94.6	1305	155.0	8.40	0.0050	685	0	1225	114.3	0.90	0.81	516	6	1518	100.0	67.65	0.0000	940	1451	136.3	2.45	0.04	765
28.5	0.4698	0.2991	0.8232	0.1473	0.0963	97.7	1282	83.0	4.99	0.0060	652	0	1206	85.6	0.65	0.79	482	5	1481	63.1	17.96	0.0000	938	1410	83.4	1.49	0.12	740
28.5	0.4699	0.2987	0.8328	0.1468	0.0942	97.5	1287	116.7	2.62	0.0020	672	0	1200	75.7	0.53	0.90	449	16	1475	98.7	6.14	0.0000	911	1399	75.4	0.99	0.20	691
28.5	0.4324	0.2981	0.7030	0.1471	0.0961	97.4	1266	79.7	1.30	0.0120	1182	36	1171	57.7	0.36	1.38	435	53	1442	63.2	1.67	0.0017	735	1351	42.1	0.52	1.40	455
28.5	0.4660	0.2970	0.7580	0.1455	0.0988	95.1	1319	120.4	11.40	0.0010	1355	0	1240	113.7	1.06	0.75	506	2	1545	82.7	138.16	0.0000	931	1479	133.5	3.20	0.02	828
28.5	0.4498	0.2981	0.7906	0.1465	0.0946	100.9	1280	71.4	1.66	0.0040	1284	15	1185	59.1	0.42	1.08	449	29	1459	67.6	2.80	0.0000	806	1375	46.9	0.68	0.60	583
28.5	0.4622	0.2963	0.7648	0.1465	0.0944	94.5	1307	116.5	2.61	0.0100	1369	10	1215	89.2	0.54	0.96	488	15	1511	132.4	5.98	0.0000	869	1431	103.4	0.97	0.30	667
28.5	0.4513	0.2977	0.7570	0.1458	0.0990	100.2	1295	72.0	5.70	0.0090	1296	0	1214	69.1	0.74	0.77	483	2	1505	104.5	33.16	0.0000	939	1434	77.7	1.78	0.09	739
28.5	0.4300	0.2992	0.7262	0.1458	0.0959	95.1	1257	77.1	0.99	0.0110	1185	63	1161	59.2	0.31	1.66	423	68	1423	67.7	1.07	0.0033	671	1325	46.1	0.41	2.49	480
28.5	0.4556	0.2987	0.8012	0.1460	0.0970	96.4	1285	113.3	2.77	0.0070	1294	0	1202	83.4	0.55	0.83	498	12	1481	98.9	7.21	0.0000	897	1405	74.3	1.03	0.20	674
28.5	0.4504	0.2992	0.8472	0.1454	0.0972	96.0	1277	97.0	2.03	0.0020	1264	0	1196	70.1	0.48	0.86	470	19	1469	95.8	3.91	0.0000	880	1389	75.7	0.82	0.30	633
28.5	0.4391	0.2973	0.7220	0.1458	0.0981	98.5	1286	94.0	2.72	0.0030	1223	0	1200	65.7	0.53	0.93	488	18	1490	69.1	6.20	0.0000	816	1410	62.2	0.94	0.32	618
28.5	0.4668	0.2988	0.7852	0.1476	0.0972	94.9	1290	118.4	4.54	0.0040	1348	0	1211	102.0	0.69	0.79	509	9	1491	122.0	18.62	0.0000	885	1420	106.9	1.52	0.10	732
28.5	0.4432	0.2975	0.8944	0.1478	0.0969	95.9	1286	81.7	1.69	0.0050	1325	0	1194	64.1	0.43	0.99	462	23	1470	87.8	2.75	0.0002	858	1388	56.8	0.70	0.44	618
28.5	0.4414	0.2989	0.8856	0.1456	0.0957	95.6	1274	91.8	1.19	0.0040	1341	0	1180	61.0	0.36	1.17	446	40	1447	68.6	1.48	0.0006	787	1358	55.0	0.52	0.93	562
28.5	0.4622	0.2990	0.7374	0.1451	0.0962	98.5	1286	88.6	3.58	0.0050	1260	0	1205	69.2	0.61	0.80	458	10	1485	83.7	12.71	0.0000	878	1411	85.1	1.29	0.15	688
28.5	0.4594	0.2963	0.8010	0.1457	0.0986	99.3	1315	91.6	11.11	0.0050	1349	0	1233	90.6	0.94	0.89	476	2	1537	68.8	90.40	0.0000	969	1469	98.6	2.60	0.04	791

Continuation of Table S1.

c_2	q_H	σ_{OO}	ϵ_{OO}	d_{OO}	d_{OH}	a_{HOO}	ρ	ϵ	μ	P_v	λ	wt%$_v$	ρ	ϵ	μ	P_v	λ	wt%$_v$	ρ	ϵ	μ	P_v	λ	ρ	ϵ	μ	P_v	λ
28.5	0.4416	0.2971	0.8192	0.1471	0.0963	97.2	1285	84.0	1.75	0.0060	1325	0	1191	58.7	0.42	1.07	443	34	1473	79.0	2.67	0.0019	811	1388	55.4	0.68	0.54	619
28.5	0.4543	0.2997	0.8742	0.1476	0.0959	95.2	1269	101.3	1.61	0.0040	1243	0	1184	61.8	0.44	0.91	461	25	1446	99.2	2.71	0.0000	867	1365	74.2	0.69	0.48	616
28.5	0.4576	0.2993	0.8848	0.1466	0.0978	94.0	1285	111.1	2.73	0.0050	1262	0	1203	80.1	0.57	0.77	508	10	1478	148.7	6.75	0.0000	931	1403	88.3	1.04	0.16	779
28.5	0.4382	0.2979	0.8326	0.1455	0.0943	96.0	1276	90.0	1.01	0.0180	1278	43	1176	58.6	0.33	1.46	439	58	1447	70.0	1.16	0.0055	759	1352	46.6	0.45	1.65	531
28.5	0.4326	0.2991	0.7518	0.1479	0.0963	95.5	1254	86.4	1.06	0.0050	1177	0	1162	63.3	0.34	1.38	412	59	1424	68.4	1.34	0.0000	743	1329	45.9	0.45	1.68	526
28.5	0.4380	0.2961	0.8984	0.1470	0.0959	101.8	1294	63.1	1.68	0.0030	1430	0	1199	48.7	0.43	1.12	464	30	1481	53.6	2.60	0.0000	827	1396	41.7	0.67	0.48	620
28.5	0.4594	0.2973	0.8848	0.1468	0.0979	101.7	1299	91.8	6.05	0.0070	1326	0	1217	71.8	0.74	0.85	500	3	1505	59.2	33.59	0.0000	1021	1436	65.8	1.72	0.06	792
28.5	0.4389	0.2979	0.8490	0.1469	0.0973	99.0	1277	61.4	1.77	0.0140	1319	6	1188	60.5	0.44	0.94	483	26	1465	57.7	2.95	0.0000	835	1382	51.2	0.70	0.44	616
28.5	0.4543	0.2990	0.7146	0.1479	0.0942	95.9	1263	80.5	1.62	0.0040	1256	0	1178	70.4	0.40	1.09	451	39	1446	96.0	2.55	0.0000	761	1361	59.1	0.65	0.83	514
28.5	0.4521	0.2990	0.8868	0.1477	0.0982	94.6	1277	71.2	2.28	0.0060	1314	0	1198	81.1	0.52	0.79	496	13	1471	117.1	5.48	0.0000	915	1395	87.7	0.96	0.20	699
28.5	0.4486	0.2961	0.8400	0.1479	0.0950	99.5	1287	43.9	2.03	0.0070	1430	8	1204	62.1	0.46	1.00	440	26	1484	63.9	3.27	0.0000	855	1402	55.7	0.75	0.46	665
28.5	0.4646	0.2998	0.7042	0.1479	0.0942	101.2	1264	83.2	2.62	0.0110	1265	5	1182	62.3	0.52	0.80	479	24	1447	70.9	7.46	0.0000	811	1369	54.9	0.97	0.27	587
28.5	0.4441	0.2995	0.7216	0.1460	0.0955	95.8	1262	89.2	1.42	0.0030	1203	0	1175	67.1	0.38	1.20	435	48	1441	73.1	1.96	0.0007	753	1352	59.9	0.56	1.04	500
28.5	0.4423	0.2990	0.8926	0.1451	0.0945	100.1	1272	74.5	1.14	0.0060	1296	34	1177	59.5	0.36	1.18	440	44	1440	55.1	1.44	0.0002	832	1352	36.7	0.50	1.05	573
28.5	0.4488	0.2973	0.7280	0.1467	0.0982	96.6	1294	101.9	3.92	0.0050	1287	0	1210	79.7	0.64	0.95	468	11	1503	119.7	14.27	0.0000	871	1428	86.8	1.33	0.18	670
28.5	0.4426	0.2964	0.8178	0.1456	0.0982	98.1	1303	95.3	3.07	0.0090	1333	32	1213	73.6	0.57	0.85	475	18	1510	79.7	7.48	0.0000	892	1431	69.5	1.05	0.18	658
28.5	0.4502	0.2973	0.7500	0.1472	0.0984	98.6	1292	97.2	4.04	0.0070	1303	0	1210	74.4	0.68	0.78	479	7	1501	95.6	22.10	0.0000	906	1428	71.2	1.47	0.09	682
28.5	0.4681	0.2960	0.8126	0.1476	0.0960	97.2	1313	112.4	6.48	0.0050	1462	0	1230	96.6	0.78	0.88	512	5	1525	73.7	33.97	0.0000	885	1455	94.2	1.78	0.07	772
28.5	0.4558	0.2973	0.8374	0.1458	0.0954	101.4	1295	78.6	2.85	0.0080	1347	0	1205	61.9	0.53	0.86	475	14	1489	71.6	6.38	0.0000	936	1412	54.7	0.98	0.26	664
28.5	0.4496	0.2971	0.8752	0.1465	0.0974	95.4	1299	109.4	2.50	0.0050	1389	0	1210	82.1	0.52	0.87	521	14	1497	95.7	5.39	0.0000	918	1419	72.5	0.95	0.23	726
28.5	0.4663	0.2975	0.7262	0.1477	0.0948	97.6	1294	104.7	3.85	0.0030	1331	0	1208	75.1	0.62	0.86	458	11	1493	107.3	12.54	0.0000	895	1418	82.2	1.26	0.20	691
28.5	0.4449	0.2990	0.8916	0.1456	0.0952	99.6	1273	73.3	1.33	0.0030	1329	0	1181	58.8	0.38	1.10	462	37	1447	57.4	1.87	0.0000	819	1361	45.3	0.58	0.71	607
28.5	0.4630	0.2970	0.8422	0.1478	0.0979	94.6	1307	132.1	5.36	0.0100	1355	0	1224	99.7	0.75	0.69	491	1	1518	130.2	27.59	0.0000	933	1447	112.4	1.77	0.07	781
28.5	0.4684	0.2976	0.8836	0.1466	0.0975	98.6	1306	115.1	6.51	0.0060	1404	0	1225	94.7	0.78	0.74	497	3	1514	96.9	54.90	0.0000	988	1446	97.0	2.07	0.04	813
28.5	0.4332	0.2984	0.7006	0.1454	0.0951	94.3	1267	73.9	1.03	0.0110	1235	46	1167	54.7	0.32	1.75	403	61	1438	73.0	1.13	0.0091	685	1339	49.7	0.42	2.47	443
28.5	0.4310	0.2999	0.7058	0.1469	0.0950	94.5	1244	74.3	0.85	0.0140	1140	91	1147	58.9	0.29	1.96	426	75	1400	62.4	0.84	0.0044	687	1298	41.0	0.36	3.52	407
30	0.4309	0.2997	0.7620	0.1457	0.0976	98.1	1258	70.8	1.29	0.0160	631	26	1169	52.5	0.37	1.20	412	52	1431	59.7	1.65	0.0004	757	1341	43.1	0.52	1.18	564
30	0.4313	0.2980	0.8738	0.1470	0.0948	100.4	1266	64.0	0.93	0.0080	655	0	1167	47.8	0.32	1.45	407	60	1429	40.5	1.06	0.0008	765	1334	31.7	0.43	1.75	535
30	0.4314	0.2973	0.8818	0.1465	0.0948	100.1	1274	67.0	0.98	0.0070	675	64	1175	49.2	0.33	1.42	404	52	1442	43.3	1.12	0.0016	777	1349	32.0	0.45	1.54	554
30	0.4316	0.2962	0.8158	0.1457	0.0953	96.4	1287	71.3	1.11	0.0100	694	19	1186	54.7	0.34	1.51	430	56	1468	57.3	1.32	0.0037	797	1373	41.7	0.48	1.57	534
30	0.4321	0.2970	0.7074	0.1471	0.0963	101.0	1275	61.5	1.52	0.0040	611	0	1179	49.3	0.40	1.10	438	43	1460	49.8	2.41	0.0000	750	1371	36.0	0.60	1.12	555
30	0.4322	0.2995	0.7930	0.1469	0.0956	99.5	1253	58.7	0.99	0.0080	592	24	1156	56.7	0.32	1.39	424	59	1413	44.3	1.16	0.0021	750	1318	34.6	0.43	1.85	492
30	0.4334	0.2963	0.7470	0.1476	0.0977	97.5	1286	72.1	1.93	0.0040	625	0	1193	58.5	0.45	0.99	431	32	1482	63.2	3.36	0.0002	804	1396	53.4	0.73	0.55	584
30	0.4334	0.2990	0.7960	0.1477	0.0957	96.6	1254	73.1	1.01	0.0050	624	35	1160	54.5	0.32	1.40	436	61	1420	57.1	1.18	0.0011	750	1323	34.6	0.43	1.90	475
30	0.4339	0.2995	0.7170	0.1455	0.0952	94.3	1255	80.6	0.92	0.0040	575	83	1159	50.2	0.31	1.74	404	71	1420	62.4	1.02	0.0039	705	1320	40.6	0.39	2.78	429
30	0.4341	0.2964	0.7102	0.1467	0.0947	96.4	1280	75.6	1.17	0.0100	623	51	1179	56.9	0.35	1.48	391	64	1462	55.7	1.46	0.0042	712	1365	45.0	0.48	1.93	513
30	0.4347	0.2994	0.7966	0.1468	0.0961	94.7	1257	80.9	1.01	0.0090	591	74	1163	55.4	0.33	1.50	431	59	1422	58.4	1.18	0.0030	740	1328	50.4	0.44	1.78	544
30	0.4349	0.2981	0.7224	0.1480	0.0948	97.5	1262	72.9	1.06	0.0050	592	20	1163	50.2	0.33	1.57	421	62	1429	52.8	1.30	0.0004	691	1333	36.5	0.45	2.04	474
30	0.4350	0.2968	0.8650	0.1474	0.0948	100.2	1278	67.4	1.14	0.0070	683	33	1181	47.2	0.35	1.38	419	51	1453	44.3	1.41	0.0005	811	1361	35.4	0.50	1.31	527
30	0.4351	0.2977	0.8318	0.1452	0.0964	101.6	1281	63.4	1.47	0.0020	627	0	1185	54.6	0.38	1.19	452	37	1463	49.9	2.11	0.0000	831	1375	36.5	0.60	0.76	576
30	0.4357	0.2985	0.8626	0.1452	0.0975	95.6	1277	77.9	1.36	0.0080	626	0	1186	64.8	0.39	1.07	464	41	1459	74.2	1.90	0.0007	789	1371	52.0	0.57	0.69	614
30	0.4358	0.2970	0.8320	0.1474	0.0989	101.4	1285	74.3	2.69	0.0040	666	0	1197	56.0	0.52	0.90	439	14	1482	53.8	5.85	0.0000	869	1403	43.6	0.92	0.27	675
30	0.4373	0.2979	0.8844	0.1472	0.0982	97.6	1279	74.0	1.78	0.0060	634	0	1191	57.6	0.44	0.92	515	25	1466	68.3	2.96	0.0000	880	1384	51.0	0.72	0.43	637
30	0.4374	0.2974	0.8750	0.1455	0.0955	101.3	1284	56.7	1.31	0.0060	725	0	1187	50.7	0.38	1.14	481	41	1463	50.3	1.81	0.0000	802	1374	36.7	0.57	0.82	601
30	0.4377	0.2964	0.8654	0.1469	0.0943	100.5	1284	69.5	1.19	0.0100	702	60	1185	53.0	0.36	1.36	436	53	1461	45.9	1.48	0.0112	829	1371	35.4	0.51	1.15	564
30	0.4383	0.2976	0.8022	0.1477	0.0976	95.1	1279	83.4	1.70	0.0070	642	9	1188	61.2	0.43	1.04	449	30	1468	70.0	2.69	0.0000	807	1383	57.6	0.68	0.56	588
30	0.4384	0.2999	0.8458	0.1457	0.0977	100.1	1260	64.9	1.54	0.0030	634	0	1177	56.6	0.41	0.99	443	33	1440	55.4	2.48	0.0000	807	1356	45.1	0.64	0.59	571
30	0.4386	0.2967	0.7352	0.1468	0.0972	96.8	1289	80.8	2.02	0.0050	638	16	1196	62.8	0.46	0.96	477	34	1485	63.9	3.78	0.0006	796	1401	51.8	0.76	0.53	627
30	0.4398	0.2992	0.7900	0.1480	0.0989	96.1	1265	81.7	2.05	0.0020	648	0	1184	66.3	0.47	0.85	449	25	1453	73.1	3.86	0.0006	823	1372	59.0	0.79	0.34	609
30	0.4406	0.2985	0.8484	0.1468	0.0962	95.4	1273	75.3	1.32	0.0090	642	12	1179	66.5	0.38	1.16	461	42	1448	67.6	1.71	0.0000	806	1360	51.8	0.55	1.02	581
30	0.4414	0.2979	0.8326	0.1457	0.0987	94.9	1288	88.5	2.18	0.0040	665	0	1202	71.6	0.49	0.93	491	20	1487	83.8	4.31	0.0000	881	1406	60.3	0.86	0.35	696

Continuation of Table S1.

c_2	q_H	σ_{OO}	ϵ_{OO}	d_{OO}	d_{OH}	a_{HOO}	ρ	ϵ	μ	P_v	λ	wt$\%_v$	ρ	ϵ	μ	P_v	λ	wt$\%_v$	ρ	ϵ	μ	P_v	λ	ρ	ϵ	μ	P_v	λ
30	0.4416	0.2976	0.8522	0.1480	0.0976	96.5	1281	76.4	1.93	0.0070	649	12	1191	61.1	0.46	0.95	478	28	1471	71.0	3.22	0.0011	858	1388	57.1	0.76	0.48	628
30	0.4417	0.2998	0.7766	0.1465	0.0947	98.7	1252	65.4	1.09	0.0030	612	23	1161	52.6	0.34	1.35	412	57	1419	53.0	1.35	0.0035	732	1327	44.6	0.47	1.54	521
30	0.4419	0.2998	0.7852	0.1466	0.0946	98.1	1248	58.7	1.04	0.0110	626	81	1162	57.8	0.34	1.40	421	58	1419	62.8	1.31	0.0026	724	1326	40.7	0.45	1.52	505
30	0.4421	0.2986	0.8328	0.1457	0.0963	97.4	1275	80.9	1.41	0.0100	634	0	1184	66.0	0.40	1.08	457	35	1456	67.9	2.10	0.0000	813	1370	53.1	0.61	0.71	608
30	0.4424	0.2965	0.8222	0.1456	0.0971	94.7	1299	97.8	1.88	0.0050	661	53	1207	69.6	0.47	0.98	469	26	1499	79.4	3.27	0.0000	839	1416	63.6	0.76	0.49	662
30	0.4426	0.2971	0.8478	0.1477	0.0952	101.1	1281	74.0	1.52	0.0080	667	16	1186	51.9	0.41	1.01	495	38	1462	46.5	2.37	0.0010	840	1376	39.7	0.63	0.61	589
30	0.4434	0.2994	0.7496	0.1459	0.0943	94.5	1256	81.5	1.04	0.0080	593	55	1165	64.5	0.32	1.47	393	58	1429	69.0	1.19	0.0031	744	1334	46.8	0.45	1.94	448
30	0.4444	0.2961	0.7208	0.1458	0.0971	101.5	1302	77.5	3.60	0.0040	690	0	1210	65.6	0.58	0.96	462	13	1507	63.1	10.88	0.0000	875	1429	49.7	1.14	0.25	641
30	0.4448	0.2994	0.8730	0.1472	0.0966	96.2	1269	90.8	1.46	0.0060	638	9	1179	59.0	0.40	1.04	474	36	1443	71.7	2.16	0.0003	811	1357	53.8	0.61	0.61	595
30	0.4467	0.2969	0.8836	0.1467	0.0947	95.5	1290	83.0	1.39	0.0100	729	18	1194	64.8	0.40	1.11	448	36	1473	68.6	1.86	0.0010	806	1386	52.3	0.58	0.83	614
30	0.4467	0.2995	0.8548	0.1467	0.0967	98.6	1269	80.5	1.70	0.0030	610	0	1182	59.5	0.43	0.89	468	26	1447	67.2	2.87	0.0000	834	1365	47.6	0.70	0.47	633
30	0.4468	0.2964	0.7678	0.1450	0.0952	100.2	1299	81.5	2.14	0.0070	690	0	1204	57.0	0.46	1.03	424	22	1494	62.9	3.92	0.0000	814	1411	47.2	0.79	0.44	622
30	0.4488	0.2982	0.8982	0.1479	0.0963	94.9	1281	96.2	1.62	0.0110	670	0	1190	65.4	0.43	1.00	461	25	1463	81.6	2.63	0.0006	850	1380	58.1	0.69	0.48	654
30	0.4491	0.2963	0.8842	0.1458	0.0947	94.8	1301	90.7	1.53	0.0050	730	0	1208	70.0	0.42	1.13	488	32	1491	84.5	2.19	0.0000	881	1406	60.0	0.64	0.59	627
30	0.4500	0.2999	0.8928	0.1473	0.0944	96.0	1262	85.7	1.14	0.0040	670	0	1170	63.6	0.36	1.16	441	42	1425	63.2	1.47	0.0017	789	1337	51.4	0.51	1.13	585
30	0.4515	0.2961	0.8328	0.1472	0.0983	94.8	1310	133.2	4.01	0.0020	694	0	1222	85.0	0.65	0.91	486	11	1520	97.1	11.89	0.0000	934	1446	89.1	1.34	0.14	770
30	0.4520	0.2990	0.7388	0.1470	0.0954	98.1	1267	81.0	1.87	0.0030	657	0	1183	59.1	0.44	1.02	472	27	1454	68.3	3.48	0.0006	781	1371	53.1	0.74	0.53	583
30	0.4525	0.2985	0.7732	0.1450	0.0987	94.9	1292	91.3	3.33	0.0050	659	0	1211	86.9	0.63	0.80	466	10	1498	108.1	11.06	0.0006	909	1425	88.1	1.27	0.15	702
30	0.4526	0.2994	0.8048	0.1464	0.0976	96.7	1275	83.3	2.57	0.0040	622	0	1194	66.4	0.52	0.83	502	24	1466	88.7	5.86	0.0000	860	1390	69.9	0.96	0.26	680
30	0.4542	0.2965	0.8428	0.1458	0.0970	100.4	1303	90.7	4.03	0.0070	696	0	1218	66.3	0.63	0.94	491	8	1511	66.2	13.98	0.0000	946	1436	60.1	1.30	0.12	726
30	0.4553	0.2961	0.8634	0.1457	0.0987	99.0	1313	103.0	6.55	0.0070	718	0	1231	73.1	0.79	0.84	516	5	1531	63.5	35.55	0.0000	1001	1461	73.6	1.91	0.05	770
30	0.4554	0.2968	0.8420	0.1477	0.0966	101.6	1296	75.8	3.88	0.0070	698	0	1209	64.5	0.60	0.97	497	13	1494	61.0	12.06	0.0000	934	1419	52.8	1.22	0.15	682
30	0.4559	0.2993	0.7728	0.1469	0.0958	100.0	1268	83.5	2.20	0.0090	611	0	1187	55.8	0.49	0.88	479	21	1456	65.8	5.59	0.0000	844	1377	48.7	0.88	0.30	629
30	0.4564	0.2998	0.8478	0.1451	0.0970	101.9	1274	76.7	2.97	0.0060	664	0	1195	59.3	0.55	0.81	498	12	1465	60.4	7.86	0.0000	903	1390	54.9	1.05	0.17	714
30	0.4567	0.2975	0.8860	0.1474	0.0959	101.0	1292	72.5	2.70	0.0050	706	0	1203	63.1	0.55	0.98	434	12	1482	63.9	6.36	0.0009	902	1406	54.9	1.00	0.18	683
30	0.4568	0.2988	0.7844	0.1466	0.0945	98.3	1270	86.0	1.74	0.0020	642	0	1185	62.1	0.44	1.00	451	29	1456	63.8	3.10	0.0004	820	1374	52.0	0.72	0.63	587
30	0.4570	0.2974	0.8404	0.1471	0.0951	94.6	1292	94.8	1.98	0.0040	700	24	1202	69.8	0.47	0.97	478	24	1483	107.6	3.39	0.0000	879	1401	65.8	0.79	0.41	648
30	0.4570	0.2974	0.7202	0.1460	0.0945	95.7	1291	92.9	2.21	0.0020	661	0	1200	66.1	0.48	0.96	455	27	1486	81.1	4.28	0.0000	797	1404	62.3	0.82	0.50	612
30	0.4571	0.2982	0.8314	0.1455	0.0968	100.1	1291	99.4	3.35	0.0080	661	0	1206	68.0	0.59	0.87	497	10	1488	91.0	9.56	0.0000	937	1413	58.1	1.15	0.14	716
30	0.4571	0.2984	0.8094	0.1461	0.0987	101.9	1291	78.2	5.85	0.0040	661	0	1211	61.7	0.76	0.86	522	5	1496	58.7	45.12	0.0000	941	1426	62.0	1.89	0.05	729
30	0.4578	0.2966	0.8450	0.1454	0.0988	99.5	1308	83.3	7.20	0.0090	704	0	1229	76.3	0.85	0.80	519	3	1529	64.7	55.42	0.0000	984	1460	74.5	2.09	0.03	777
30	0.4578	0.2988	0.8056	0.1455	0.0989	94.3	1292	128.6	4.22	0.0030	690	0	1214	85.8	0.67	0.86	488	7	1499	129.5	16.03	0.0000	929	1428	91.8	1.48	0.11	738
30	0.4581	0.2988	0.8232	0.1466	0.0950	94.7	1280	101.5	1.79	0.0000	636	0	1190	80.6	0.44	0.96	444	28	1464	98.7	3.08	0.0017	846	1382	69.1	0.72	0.50	667
30	0.4586	0.2972	0.7200	0.1462	0.0975	99.7	1298	72.4	6.98	0.0040	677	0	1218	77.4	0.79	0.88	479	5	1513	59.9	47.82	0.0000	930	1441	67.5	1.93	0.07	713
30	0.4586	0.2990	0.7792	0.1464	0.0980	100.5	1281	79.3	4.89	0.0030	640	0	1204	68.3	0.69	0.78	480	6	1482	93.7	25.59	0.0000	925	1412	67.8	1.58	0.10	729
30	0.4592	0.2993	0.8844	0.1471	0.0960	98.7	1272	83.8	2.26	0.0030	665	0	1192	67.0	0.50	0.93	538	20	1459	77.8	4.74	0.0000	905	1383	58.0	0.89	0.25	706
30	0.4595	0.2970	0.8538	0.1473	0.0984	101.0	1302	84.2	6.81	0.0020	683	0	1219	80.3	0.80	0.81	512	3	1511	85.7	51.69	0.0000	991	1443	67.8	2.07	0.04	781
30	0.4598	0.2965	0.7980	0.1456	0.0981	94.7	1312	103.4	5.69	0.0030	677	0	1230	97.6	0.78	0.80	483	7	1533	122.8	29.55	0.0000	922	1463	100.4	1.75	0.08	715
30	0.4598	0.2966	0.7906	0.1475	0.0948	94.5	1298	89.2	2.49	0.0050	693	0	1208	82.0	0.51	0.99	448	19	1496	103.9	4.68	0.0021	869	1417	81.0	0.91	0.39	663
30	0.4600	0.2988	0.8122	0.1475	0.0975	100.0	1281	85.8	4.26	0.0050	642	0	1201	69.4	0.65	0.80	491	7	1478	74.2	18.43	0.0000	924	1406	61.5	1.43	0.12	699
30	0.4616	0.2962	0.8924	0.1477	0.0955	97.3	1303	106.9	3.22	0.0050	746	0	1220	72.7	0.58	1.02	459	7	1506	112.9	7.16	0.0000	961	1431	75.7	1.10	0.13	723
30	0.4623	0.2994	0.7994	0.1466	0.0951	97.9	1275	101.5	2.27	0.0050	651	0	1191	68.3	0.49	0.90	448	25	1460	80.4	4.64	0.0004	842	1382	59.3	0.88	0.33	627
30	0.4633	0.2962	0.7440	0.1453	0.0942	99.8	1304	83.3	3.60	0.0030	673	0	1221	76.1	0.60	0.94	465	15	1513	75.8	10.61	0.0000	876	1436	66.9	1.19	0.19	647
30	0.4637	0.2972	0.7404	0.1475	0.0970	101.1	1297	72.6	7.42	0.0060	668	0	1216	63.6	0.82	0.83	515	3	1509	58.1	64.68	0.0000	926	1438	70.7	2.14	0.07	741
30	0.4644	0.2980	0.7244	0.1474	0.0954	100.5	1285	78.2	4.20	0.0050	636	0	1203	56.8	0.64	0.86	484	12	1485	86.3	18.41	0.0000	885	1411	63.1	1.36	0.16	623
30	0.4646	0.2993	0.7568	0.1456	0.0985	98.5	1288	85.9	7.02	0.0050	676	0	1213	79.2	0.84	0.68	510	2	1494	38.5	70.24	0.0000	940	1428	73.4	2.17	0.07	743
30	0.4647	0.2981	0.7146	0.1467	0.0949	94.9	1286	70.7	2.64	0.0050	655	0	1204	75.6	0.56	1.01	447	18	1489	146.5	7.83	0.0028	844	1412	76.5	1.06	0.27	622
30	0.4649	0.2984	0.7064	0.1477	0.0972	101.8	1288	74.2	7.69	0.0040	656	0	1208	64.3	0.84	0.76	471	3	1490	62.7	90.45	0.0000	903	1423	62.9	2.33	0.04	708
30	0.4653	0.2969	0.8344	0.1472	0.0969	101.5	1304	81.9	7.66	0.0050	696	0	1220	75.8	0.80	0.94	521	3	1509	57.7	43.86	0.0000	936	1441	67.3	1.94	0.04	770
30	0.4665	0.2962	0.7562	0.1479	0.0964	101.8	1306	87.2	10.34	0.0040	735	0	1225	69.2	0.89	0.86	485	3	1519	57.0	124.99	0.0000	945	1450	65.9	2.33	0.07	743

Continuation of Table S1.

c_2	q_H	σ_{OO}	ϵ_{OO}	d_{OO}	d_{OH}	a_{HOO}	ρ	ϵ	μ	P_v	λ	wt$\%_v$	ρ	ϵ	μ	P_v	λ	wt$\%_v$	ρ	ϵ	μ	P_v	λ	ρ	ϵ	μ	P_v	λ
30	0.4672	0.3000	0.7220	0.1470	0.0965	94.8	1279	122.6	3.44	0.0060	626	0	1201	91.5	0.62	0.75	496	11	1473	122.8	13.23	0.0000	874	1400	82.6	1.33	0.15	681
30	0.4674	0.2985	0.8584	0.1454	0.0976	94.3	1300	127.3	4.87	0.0030	677	0	1221	99.8	0.70	0.78	498	6	1508	182.2	17.14	0.0000	969	1438	113.1	1.59	0.06	809
30	0.4678	0.2997	0.8038	0.1476	0.0979	101.1	1279	83.3	6.20	0.0060	658	0	1204	70.3	0.79	0.72	498	3	1476	67.9	59.23	0.0000	930	1409	66.1	2.01	0.04	726
30	0.4685	0.2982	0.7654	0.1459	0.0943	101.6	1292	89.4	3.59	0.0040	690	0	1204	62.9	0.58	0.84	485	8	1485	72.5	11.32	0.0008	894	1410	60.1	1.20	0.22	682
30	0.4686	0.2968	0.8040	0.1456	0.0980	100.6	1314	129.4	14.46	0.0050	708	0	1233	72.1	1.02	0.85	524	3	1534	39.5	277.96	0.0000	923	1468	91.9	3.44	0.02	808
30	0.4689	0.2991	0.8576	0.1451	0.0982	100.4	1293	101.5	7.54	0.0030	670	0	1218	85.2	0.84	0.77	493	2	1500	69.0	69.52	0.0000	1021	1434	74.3	2.28	0.05	817
30	0.4693	0.2969	0.8730	0.1477	0.0954	97.0	1300	92.3	3.80	0.0040	712	0	1220	73.2	0.64	0.90	507	5	1505	92.9	12.56	0.0000	938	1432	84.0	1.32	0.11	751
30	0.4580	0.2960	0.8984	0.1474	0.0977	101.1	1310	86.7	6.37	0.0070	1507	0	1228	67.6	0.74	0.86	509	5	1519	47.4	28.93	0.0000	943	1448	67.3	1.71	0.06	760
30	0.4405	0.2960	0.7966	0.1466	0.0949	100.5	1290	67.8	1.55	0.0060	1317	7	1194	54.3	0.41	1.13	423	37	1479	52.9	2.31	0.0004	792	1391	37.9	0.63	0.77	599
30	0.4500	0.2993	0.8636	0.1473	0.0955	100.7	1268	80.8	1.64	0.0100	1332	12	1179	55.3	0.41	1.02	446	32	1442	55.1	2.70	0.0000	842	1360	46.5	0.67	0.54	646
30	0.4323	0.2996	0.7366	0.1475	0.0950	101.3	1247	52.6	1.00	0.0090	1172	44	1150	44.7	0.31	1.58	423	64	1405	43.1	1.13	0.0019	667	1309	33.2	0.42	2.11	455
30	0.4590	0.2989	0.8182	0.1475	0.0941	98.6	1274	81.4	1.73	0.0050	1310	0	1183	55.5	0.43	1.03	462	33	1450	60.9	2.97	0.0000	837	1369	55.5	0.71	0.57	628
30	0.4326	0.2978	0.7638	0.1479	0.0966	98.9	1267	71.7	1.33	0.0020	1242	0	1173	51.1	0.37	1.12	413	44	1445	51.5	1.89	0.0020	764	1355	39.5	0.55	1.11	537
30	0.4617	0.2979	0.7112	0.1462	0.0966	96.0	1297	110.6	4.27	0.0020	1247	0	1214	83.6	0.67	0.77	486	9	1503	80.6	16.80	0.0000	910	1430	87.0	1.46	0.14	680
30	0.4471	0.2994	0.8986	0.1469	0.0947	100.2	1266	71.7	1.26	0.0060	1321	0	1173	50.4	0.37	1.05	452	40	1432	50.3	1.68	0.0000	830	1346	41.0	0.55	0.79	565
30	0.4533	0.2990	0.7046	0.1468	0.0985	95.8	1282	102.8	3.87	0.0020	1188	0	1203	79.6	0.64	0.79	513	11	1484	85.0	15.49	0.0000	824	1411	86.9	1.39	0.18	659
30	0.4463	0.2987	0.8034	0.1462	0.0970	101.4	1277	77.7	2.25	0.0090	1369	0	1189	55.8	0.48	0.98	448	20	1463	54.1	4.70	0.0000	821	1384	45.5	0.84	0.40	617
30	0.4534	0.2987	0.8948	0.1471	0.0950	96.9	1277	80.7	1.55	0.0040	1265	0	1187	62.8	0.42	1.00	463	27	1454	73.9	2.34	0.0000	843	1371	55.5	0.66	0.58	655
30	0.4438	0.2994	0.7724	0.1461	0.0960	98.9	1265	81.1	1.51	0.0070	1303	0	1176	53.8	0.40	1.02	490	42	1442	57.0	2.33	0.0007	784	1357	43.3	0.61	0.75	547
30	0.4350	0.2991	0.7934	0.1462	0.0954	101.7	1260	67.8	1.10	0.0070	1223	12	1165	46.8	0.35	1.20	430	51	1427	42.8	1.44	0.0000	744	1335	32.6	0.48	1.40	517
30	0.4404	0.2968	0.7510	0.1464	0.0969	99.8	1290	70.7	2.27	0.0040	1306	0	1197	56.4	0.48	1.04	438	29	1485	67.2	4.45	0.0006	827	1403	45.5	0.82	0.46	595
30	0.4680	0.2988	0.7710	0.1479	0.0956	97.7	1284	99.5	3.89	0.0060	1279	0	1201	76.1	0.60	0.84	496	12	1477	73.2	13.07	0.0000	903	1404	73.7	1.31	0.15	651
30	0.4632	0.2981	0.7992	0.1466	0.0944	94.2	1288	110.4	1.99	0.0070	1372	0	1198	73.6	0.47	0.99	472	23	1479	103.2	3.70	0.0000	885	1398	80.0	0.82	0.45	592
30	0.4561	0.2994	0.8802	0.1450	0.0967	100.0	1280	89.0	2.50	0.0030	1327	0	1197	66.3	0.51	0.81	499	13	1469	73.8	5.65	0.0000	918	1394	58.4	0.94	0.24	675
30	0.4401	0.2988	0.7564	0.1463	0.0981	101.8	1268	63.7	2.37	0.0060	1238	37	1186	55.1	0.50	0.81	450	29	1460	54.0	5.30	0.0004	833	1381	44.6	0.87	0.35	616
30	0.4391	0.2960	0.7012	0.1464	0.0978	96.0	1300	110.6	2.85	0.0040	1282	0	1208	62.6	0.54	1.01	460	24	1507	73.4	6.14	0.0000	813	1426	62.5	0.96	0.36	583
30	0.4365	0.2977	0.7694	0.1452	0.0943	98.0	1275	71.4	1.06	0.0050	1296	49	1174	52.9	0.34	1.58	409	59	1447	55.0	1.24	0.0033	723	1352	43.2	0.45	1.88	492
30	0.4571	0.2970	0.7660	0.1459	0.0960	101.9	1299	97.4	4.04	0.0060	1305	0	1211	60.6	0.62	0.96	503	8	1501	68.7	14.76	0.0000	897	1425	61.0	1.25	0.16	674
30	0.4488	0.2962	0.8364	0.1479	0.0978	101.2	1296	76.5	4.02	0.0040	1340	0	1213	63.2	0.63	0.89	493	7	1502	54.5	14.17	0.0000	889	1427	57.4	1.27	0.13	696
30	0.4409	0.2965	0.8004	0.1450	0.0982	99.4	1302	83.0	2.85	0.0050	1294	11	1211	55.5	0.56	0.86	477	14	1506	69.1	7.15	0.0000	878	1428	55.0	1.02	0.26	706
30	0.4515	0.2976	0.7296	0.1471	0.0956	96.9	1286	78.7	2.25	0.0070	1225	7	1194	64.9	0.49	0.98	469	24	1476	81.9	4.38	0.0004	796	1395	65.0	0.83	0.49	616
30	0.4498	0.2970	0.7226	0.1467	0.0970	99.9	1292	87.9	3.71	0.0050	1295	0	1206	59.9	0.59	0.84	465	14	1495	78.5	11.77	0.0000	899	1420	57.2	1.21	0.22	647
30	0.4398	0.2985	0.7814	0.1455	0.0988	94.0	1279	98.3	2.03	0.0080	1253	0	1196	75.0	0.48	0.92	485	29	1478	87.3	3.78	0.0009	843	1396	72.7	0.80	0.37	613
30	0.4362	0.2984	0.7136	0.1472	0.0979	101.8	1270	67.6	2.21	0.0080	1215	0	1182	50.7	0.47	0.87	468	23	1456	46.0	4.64	0.0005	725	1375	41.2	0.80	0.43	602
30	0.4540	0.2962	0.7760	0.1457	0.0960	98.9	1304	80.8	3.65	0.0050	1401	0	1217	72.0	0.59	0.98	492	15	1512	63.5	8.95	0.0000	906	1435	63.1	1.10	0.21	635
30	0.4652	0.2996	0.8258	0.1464	0.0950	96.5	1275	107.5	2.22	0.0050	1314	0	1193	76.6	0.49	0.88	523	20	1462	93.6	4.55	0.0000	864	1384	64.7	0.88	0.32	663
30	0.4405	0.2997	0.7040	0.1460	0.0944	99.8	1253	66.2	1.11	0.0100	1198	46	1160	56.7	0.34	1.31	424	59	1419	54.3	1.38	0.0018	667	1326	38.3	0.46	1.79	461
30	0.4564	0.2978	0.8150	0.1463	0.0955	97.5	1289	87.6	2.22	0.0030	1318	0	1201	66.4	0.50	0.97	480	24	1482	79.1	4.70	0.0000	865	1402	60.1	0.87	0.32	677
30	0.4641	0.2978	0.8608	0.1456	0.0964	97.8	1300	98.4	3.97	0.0050	1424	0	1216	76.8	0.63	0.88	492	8	1502	108.1	12.55	0.0000	926	1429	75.7	1.30	0.10	760
30	0.4575	0.2997	0.8336	0.1466	0.0962	97.8	1272	82.1	2.15	0.0050	1345	17	1189	61.0	0.49	0.89	497	16	1456	93.7	4.67	0.0000	863	1378	54.3	0.86	0.28	633
30	0.4510	0.2978	0.7066	0.1480	0.0974	96.1	1284	104.9	3.34	0.0060	1273	0	1200	67.9	0.58	0.78	470	14	1485	78.8	9.26	0.0000	819	1409	70.1	1.16	0.24	608
30	0.4551	0.2962	0.7500	0.1478	0.0940	101.6	1293	74.3	2.58	0.0030	1353	0	1201	56.0	0.50	0.95	457	20	1486	57.1	5.39	0.0000	857	1405	43.8	0.87	0.38	622
30	0.4513	0.3000	0.8552	0.1477	0.0966	94.1	1263	86.5	1.60	0.0050	1235	0	1180	77.4	0.42	1.00	482	32	1441	100.7	2.57	0.0000	833	1358	58.0	0.66	0.57	587
30	0.4464	0.2989	0.8096	0.1472	0.0981	97.0	1273	80.7	2.37	0.0050	1292	0	1191	64.4	0.50	0.82	474	20	1465	73.8	4.80	0.0000	840	1387	61.3	0.89	0.30	669
30	0.4332	0.2966	0.8108	0.1452	0.0955	94.2	1288	78.9	1.09	0.0190	1256	78	1185	62.3	0.35	1.42	419	56	1470	62.1	1.29	0.0029	803	1374	50.1	0.48	1.73	577
30	0.4535	0.2962	0.7690	0.1469	0.0953	97.5	1299	84.4	2.49	0.0060	1314	0	1208	68.0	0.53	0.97	480	18	1500	82.0	5.39	0.0005	847	1419	62.2	0.93	0.38	648
30	0.4339	0.2987	0.8534	0.1458	0.0973	98.2	1270	72.3	1.32	0.0040	1284	0	1179	57.2	0.38	1.06	446	40	1449	56.8	1.83	0.0013	818	1361	45.4	0.57	0.80	563
30	0.4419	0.2993	0.7906	0.1462	0.0943	98.9	1260	68.3	1.11	0.0050	1254	61	1164	53.7	0.34	1.43	434	58	1425	58.5	1.30	0.0026	746	1332	37.0	0.46	1.61	519
30	0.4493	0.2987	0.7842	0.1468	0.0970	95.7	1275	89.6	2.09	0.0010	1253	0	1191	68.7	0.47	0.84	481	21	1467	86.6	4.06	0.0000	847	1385	58.2	0.80	0.49	580
30	0.4679	0.2988	0.7638	0.1479	0.0968	98.1	1286	91.2	5.03	0.0030	1298	0	1206	78.8	0.72	0.83	488	4	1485	69.2	28.90	0.0000	924	1415	80.4	1.73	0.11	721

Continuation of Table S1.

c_2	q_H	σ_{OO}	ϵ_{OO}	d_{OO}	d_{OH}	a_{HOO}	ρ	ϵ	μ	P_v	λ	wt%$_v$	ρ	ϵ	μ	P_v	λ	wt%$_v$	ρ	ϵ	μ	P_v	λ	ρ	ϵ	μ	P_v	λ
31.5	0.4306	0.2972	0.7420	0.1461	0.0943	99.0	1271	69.9	0.95	0.0100	633	69	1166	45.8	0.32	1.82	423	67	1439	39.7	1.08	0.0059	722	1339	33.1	0.40	2.43	457
31.5	0.4312	0.2963	0.8128	0.1474	0.0952	100.6	1279	63.0	1.17	0.0190	676	80	1179	46.0	0.35	1.44	432	51	1457	44.7	1.49	0.0013	757	1363	28.4	0.49	1.45	490
31.5	0.4314	0.2978	0.8900	0.1477	0.0985	94.4	1277	73.4	1.40	0.0040	645	0	1184	60.6	0.39	1.11	458	38	1458	64.7	1.92	0.0000	847	1371	47.1	0.59	0.76	628
31.5	0.4320	0.2995	0.7420	0.1464	0.0982	97.7	1258	73.7	1.43	0.0040	619	0	1172	52.3	0.39	1.03	448	44	1437	53.3	2.27	0.0007	756	1349	43.3	0.59	0.95	564
31.5	0.4321	0.2981	0.8956	0.1477	0.0986	100.3	1295	94.3	2.56	0.0030	717	0	1205	72.1	0.53	0.97	496	19	1489	92.9	5.52	0.0000	900	1411	69.8	0.97	0.30	689
31.5	0.4328	0.2992	0.8228	0.1458	0.0968	98.4	1264	64.7	1.18	0.0060	603	27	1170	53.2	0.35	1.25	422	54	1434	48.6	1.47	0.0007	762	1343	36.5	0.50	1.29	563
31.5	0.4337	0.2967	0.8158	0.1478	0.0986	101.6	1286	62.5	2.35	0.0040	646	0	1194	47.8	0.50	0.89	469	20	1480	43.9	4.58	0.0000	851	1399	37.3	0.85	0.41	667
31.5	0.4353	0.2983	0.8214	0.1464	0.0972	99.0	1272	60.7	1.47	0.0070	616	0	1180	52.5	0.39	1.08	412	36	1453	55.4	2.20	0.0005	809	1366	39.7	0.59	0.76	588
31.5	0.4365	0.2986	0.8656	0.1465	0.0989	96.7	1274	81.0	1.84	0.0080	630	0	1189	60.9	0.44	0.85	456	31	1462	59.3	3.01	0.0000	870	1381	50.3	0.72	0.38	600
31.5	0.4365	0.2990	0.7272	0.1477	0.0980	94.6	1262	79.5	1.58	0.0020	590	0	1176	64.1	0.41	1.08	432	39	1445	68.8	2.42	0.0022	784	1359	55.3	0.63	0.88	564
31.5	0.4382	0.2990	0.7712	0.1464	0.0943	101.7	1259	56.4	1.06	0.0070	598	56	1160	44.5	0.33	1.47	394	60	1422	37.6	1.29	0.0016	758	1328	31.6	0.45	1.65	450
31.5	0.4382	0.2995	0.8114	0.1472	0.0945	96.7	1251	62.3	0.93	0.0180	598	39	1155	53.2	0.31	1.65	382	67	1410	45.9	1.01	0.0033	725	1313	34.6	0.40	2.15	520
31.5	0.4392	0.2974	0.7604	0.1462	0.0970	100.2	1282	71.1	2.04	0.0070	642	0	1190	55.8	0.46	1.02	474	29	1473	50.2	3.58	0.0000	805	1389	40.0	0.75	0.56	593
31.5	0.4396	0.2961	0.7336	0.1469	0.0953	94.1	1290	81.4	1.40	0.0160	616	14	1189	57.6	0.39	1.35	446	50	1478	70.5	1.92	0.0014	763	1387	54.9	0.57	1.28	492
31.5	0.4399	0.2998	0.7108	0.1460	0.0969	100.9	1259	70.0	1.71	0.0060	607	51	1173	52.2	0.42	1.03	439	39	1438	49.9	3.05	0.0003	772	1353	39.5	0.66	0.79	543
31.5	0.4404	0.2997	0.7576	0.1473	0.0955	100.4	1253	59.5	1.25	0.0060	574	33	1162	49.5	0.36	1.16	439	50	1421	46.3	1.74	0.0010	710	1331	35.9	0.52	1.16	512
31.5	0.4436	0.2963	0.8674	0.1476	0.0973	99.2	1295	79.5	2.38	0.0060	703	35	1203	54.6	0.51	0.92	498	22	1491	50.2	4.79	0.0000	894	1411	47.8	0.89	0.34	682
31.5	0.4438	0.2987	0.7598	0.1466	0.0985	96.2	1273	73.1	2.30	0.0040	632	38	1192	63.5	0.50	0.81	477	21	1470	69.2	5.27	0.0000	857	1390	56.8	0.89	0.37	623
31.5	0.4440	0.2962	0.7696	0.1452	0.0951	99.1	1296	69.0	1.81	0.0080	664	0	1199	59.0	0.43	1.13	460	33	1490	55.2	2.82	0.0018	787	1403	44.3	0.68	0.81	588
31.5	0.4449	0.2984	0.7246	0.1468	0.0969	96.6	1274	67.3	2.10	0.0090	636	0	1187	63.7	0.46	0.93	483	32	1465	69.0	3.98	0.0000	771	1382	60.4	0.77	0.54	607
31.5	0.4454	0.2982	0.8386	0.1456	0.0989	94.7	1288	97.2	2.41	0.0020	629	0	1203	71.5	0.52	0.96	478	20	1487	95.9	5.36	0.0000	872	1409	71.5	0.95	0.23	663
31.5	0.4460	0.2974	0.7444	0.1470	0.0987	96.0	1287	81.5	3.29	0.0100	666	0	1205	67.0	0.59	0.89	467	11	1495	64.6	9.67	0.0000	855	1419	63.8	1.16	0.23	651
31.5	0.4463	0.2973	0.8268	0.1457	0.0946	99.2	1284	76.8	1.51	0.0050	671	32	1189	52.1	0.39	1.12	457	39	1468	52.1	2.15	0.0000	811	1381	42.9	0.60	0.92	569
31.5	0.4483	0.2969	0.8884	0.1480	0.0956	96.8	1289	81.6	1.71	0.0080	705	0	1197	63.7	0.44	1.08	464	29	1474	74.0	2.76	0.0000	867	1391	52.2	0.69	0.52	654
31.5	0.4493	0.2982	0.7404	0.1458	0.0985	99.9	1286	76.6	4.23	0.0020	641	0	1205	61.4	0.65	0.77	501	9	1490	70.9	17.24	0.0000	873	1417	57.6	1.39	0.16	698
31.5	0.4498	0.2998	0.8854	0.1470	0.0987	96.3	1271	77.0	2.25	0.0080	637	0	1192	71.2	0.51	0.82	491	15	1458	69.7	5.18	0.0000	928	1382	58.9	0.92	0.29	714
31.5	0.4501	0.2972	0.7490	0.1461	0.0943	94.7	1287	87.1	1.53	0.0040	640	0	1190	56.3	0.40	1.22	457	44	1474	83.1	2.20	0.0009	768	1385	54.6	0.60	0.99	558
31.5	0.4520	0.2967	0.8208	0.1475	0.0940	99.5	1288	70.7	1.71	0.0040	693	0	1194	54.9	0.43	1.06	424	30	1473	61.8	2.82	0.0000	833	1388	44.3	0.67	0.62	602
31.5	0.4521	0.2967	0.8420	0.1454	0.0973	101.4	1304	75.7	3.80	0.0050	657	0	1216	59.5	0.62	0.84	475	10	1508	50.8	11.76	0.0000	922	1433	50.3	1.24	0.15	740
31.5	0.4522	0.2983	0.7896	0.1479	0.0954	95.6	1276	89.1	1.77	0.0070	632	0	1185	64.1	0.43	1.00	476	30	1458	65.3	2.82	0.0005	816	1374	57.8	0.69	0.60	557
31.5	0.4523	0.2973	0.8196	0.1479	0.0980	95.6	1292	96.5	2.99	0.0020	642	0	1207	69.7	0.59	0.86	475	13	1495	89.0	9.20	0.0000	911	1419	64.8	1.15	0.18	682
31.5	0.4540	0.2967	0.8518	0.1472	0.0984	94.8	1302	101.9	3.97	0.0040	686	0	1218	84.3	0.66	0.89	496	8	1512	113.7	12.43	0.0000	943	1438	75.3	1.32	0.18	724
31.5	0.4568	0.2999	0.8050	0.1475	0.0968	95.7	1268	89.4	2.17	0.0030	600	0	1187	72.3	0.49	0.80	500	25	1452	87.7	4.66	0.0000	859	1373	70.6	0.85	0.29	632
31.5	0.4598	0.2998	0.7998	0.1472	0.0965	95.2	1272	83.2	2.36	0.0070	638	0	1190	72.3	0.50	0.78	478	24	1458	73.6	5.16	0.0000	859	1380	72.9	0.90	0.34	647
31.5	0.4603	0.2985	0.7838	0.1466	0.0956	100.0	1283	76.3	2.92	0.0050	650	0	1197	64.2	0.54	0.93	475	18	1475	67.4	8.03	0.0000	884	1397	58.1	1.03	0.28	642
31.5	0.4609	0.2995	0.8450	0.1458	0.0971	99.6	1279	71.0	3.36	0.0100	664	0	1200	68.5	0.57	0.78	481	9	1473	73.7	10.56	0.0000	865	1399	61.8	1.19	0.16	695
31.5	0.4612	0.2971	0.8276	0.1474	0.0957	94.7	1298	100.0	2.79	0.0120	665	4	1209	76.9	0.54	0.96	494	19	1496	103.5	6.24	0.0000	878	1418	76.5	0.99	0.30	707
31.5	0.4616	0.2974	0.8356	0.1475	0.0954	95.5	1294	96.3	2.59	0.0050	664	0	1205	73.1	0.53	0.90	468	17	1489	84.9	5.60	0.0004	846	1411	64.8	0.98	0.27	637
31.5	0.4616	0.2978	0.8842	0.1461	0.0948	99.0	1290	81.8	2.26	0.0060	695	0	1204	64.9	0.50	1.00	484	15	1481	65.1	4.95	0.0000	879	1404	53.1	0.89	0.31	703
31.5	0.4621	0.2993	0.8566	0.1451	0.0976	101.6	1285	78.4	4.24	0.0050	654	0	1207	67.8	0.67	0.80	513	3	1484	90.5	20.44	0.0000	975	1413	60.7	1.44	0.09	784
31.5	0.4624	0.2968	0.8342	0.1466	0.0943	97.6	1300	92.8	2.49	0.0040	685	0	1209	66.2	0.52	0.94	487	17	1494	74.8	5.05	0.0000	909	1414	58.8	0.90	0.36	674
31.5	0.4625	0.2971	0.8008	0.1472	0.0964	94.1	1297	98.6	3.24	0.0040	682	0	1214	83.9	0.60	0.87	508	11	1505	85.4	9.63	0.0000	879	1429	87.9	1.21	0.25	679
31.5	0.4628	0.2979	0.8642	0.1461	0.0982	95.4	1299	100.6	4.53	0.0030	672	0	1220	83.0	0.71	0.86	505	3	1509	139.6	20.93	0.0000	975	1438	90.0	1.59	0.09	751
31.5	0.4630	0.2963	0.7540	0.1452	0.0949	94.8	1309	104.8	3.16	0.0040	676	0	1220	83.5	0.58	0.94	503	14	1520	93.1	7.95	0.0000	872	1441	76.6	1.09	0.26	695
31.5	0.4638	0.2979	0.7248	0.1470	0.0956	101.5	1288	77.3	4.84	0.0040	667	0	1205	59.0	0.64	0.82	516	8	1487	53.1	21.19	0.0000	920	1414	52.6	1.44	0.20	657
31.5	0.4639	0.2970	0.7946	0.1470	0.0953	98.4	1299	103.0	3.63	0.0070	695	0	1211	76.3	0.61	0.89	499	11	1499	82.9	10.69	0.0000	902	1423	67.5	1.21	0.19	689
31.5	0.4646	0.2966	0.8202	0.1475	0.0981	99.8	1306	86.7	9.59	0.0040	705	0	1226	71.7	0.91	0.88	509	3	1523	121.9	106.71	0.0000	974	1454	74.4	2.58	0.04	807
31.5	0.4652	0.2997	0.7590	0.1463	0.0940	95.4	1269	87.5	1.84	0.0080	623	0	1186	69.2	0.45	0.92	460	28	1455	81.5	3.46	0.0003	800	1372	60.9	0.74	0.55	607
31.5	0.4659	0.2971	0.7664	0.1478	0.0960	101.1	1297	78.1	6.43	0.0090	656	0	1214	66.0	0.75	0.89	479	5	1501	104.9	31.79	0.0000	903	1429	64.3	1.73	0.10	698
31.5	0.4660	0.2976	0.7272	0.1454	0.0941	100.4	1294	84.6	3.46	0.0040	679	0	1207	58.8	0.57	0.88	458	16	1493	88.1	10.07	0.0000	866	1416	60.6	1.12	0.28	680

Continuation of Table S1.

c_2	q_H	σ_{OO}	ϵ_{OO}	d_{OO}	d_{OH}	a_{HOO}	ρ	ϵ	μ	P_v	λ	wt$\%_v$	ρ	ϵ	μ	P_v	λ	wt$\%_v$	ρ	ϵ	μ	P_v	λ	ρ	ϵ	μ	P_v	λ
31.5	0.4664	0.2983	0.8740	0.1469	0.0948	99.1	1288	91.0	2.64	0.0050	674	0	1201	62.3	0.52	0.99	488	17	1476	74.9	6.15	0.0000	890	1400	57.8	0.99	0.20	691
31.5	0.4666	0.2996	0.8732	0.1469	0.0969	101.7	1278	74.6	3.91	0.0090	665	0	1200	62.5	0.64	0.75	516	5	1471	77.2	17.76	0.0000	928	1400	54.2	1.41	0.08	747
31.5	0.4675	0.2982	0.7088	0.1452	0.0965	101.0	1297	76.2	7.87	0.0030	658	0	1217	73.5	0.82	0.89	485	6	1505	46.6	62.55	0.0000	827	1436	63.8	2.13	0.06	706
31.5	0.4678	0.2996	0.8596	0.1468	0.0953	97.7	1277	103.9	2.60	0.0010	658	0	1195	68.2	0.52	0.80	505	16	1463	79.8	6.04	0.0000	888	1387	68.2	0.99	0.23	683
31.5	0.4686	0.2976	0.7128	0.1458	0.0950	97.3	1298	98.4	4.90	0.0030	675	0	1214	79.6	0.66	0.88	486	7	1504	77.8	16.74	0.0000	882	1430	81.6	1.44	0.17	698
31.5	0.4699	0.2997	0.8594	0.1476	0.0969	98.7	1279	96.9	4.22	0.0070	650	0	1201	70.1	0.65	0.78	493	4	1471	93.8	17.15	0.0000	963	1401	73.3	1.46	0.08	750
31.5	0.4534	0.2984	0.8438	0.1464	0.0989	101.8	1286	73.5	4.26	0.0060	1320	0	1206	60.9	0.69	0.80	532	10	1489	68.7	26.00	0.0000	919	1418	52.9	1.51	0.08	727
31.5	0.4369	0.2981	0.8124	0.1454	0.0941	95.6	1271	74.1	0.92	0.0070	1275	39	1169	52.6	0.31	1.71	403	67	1436	55.6	0.97	0.0018	752	1339	38.6	0.40	2.53	494
31.5	0.4400	0.2991	0.7372	0.1471	0.0989	98.4	1267	78.1	2.39	0.0030	1246	0	1186	53.4	0.51	0.79	470	20	1459	62.3	5.59	0.0000	827	1380	53.8	0.89	0.33	567
31.5	0.4511	0.2994	0.7592	0.1473	0.0941	98.4	1259	72.9	1.35	0.0040	1220	20	1169	56.2	0.38	1.12	418	49	1431	55.9	1.90	0.0000	777	1343	44.7	0.55	1.11	535
31.5	0.4418	0.2999	0.7124	0.1463	0.0971	96.2	1260	81.3	1.56	0.0060	1231	14	1175	59.2	0.42	0.99	430	39	1441	58.5	2.66	0.0002	762	1354	48.4	0.63	0.86	555
31.5	0.4556	0.2965	0.8798	0.1453	0.0957	95.3	1306	95.3	2.28	0.0030	1461	0	1217	66.7	0.50	0.97	484	17	1506	99.0	4.43	0.0000	881	1426	64.0	0.87	0.36	678
31.5	0.4586	0.2971	0.7116	0.1459	0.0977	96.9	1304	75.0	6.14	0.0040	1306	0	1221	74.3	0.76	0.88	520	8	1519	53.8	35.44	0.0000	888	1447	77.4	1.86	0.11	697
31.5	0.4403	0.2964	0.7236	0.1479	0.0969	96.2	1290	79.1	2.12	0.0040	1268	0	1196	60.2	0.47	1.07	473	29	1484	69.1	3.92	0.0008	828	1401	52.6	0.78	0.54	574
31.5	0.4619	0.2985	0.8136	0.1462	0.0974	100.8	1288	79.9	4.80	0.0080	1324	0	1207	65.4	0.70	0.82	508	4	1490	61.8	22.68	0.0000	884	1419	56.2	1.58	0.11	737
31.5	0.4475	0.2979	0.7154	0.1456	0.0970	96.9	1287	85.6	2.52	0.0060	1276	0	1198	58.5	0.52	0.98	470	25	1485	68.9	5.88	0.0000	822	1405	59.6	0.93	0.41	588
31.5	0.4448	0.2969	0.8986	0.1463	0.0959	96.5	1293	78.8	1.54	0.0060	1436	6	1198	60.2	0.43	1.02	471	33	1479	69.8	2.34	0.0000	885	1395	48.0	0.66	0.54	672
31.5	0.4392	0.2991	0.7886	0.1456	0.0945	101.0	1262	58.0	1.12	0.0050	1262	21	1165	25.9	0.33	1.51	421	57	1427	43.2	1.32	0.0032	733	1335	30.6	0.46	1.63	521
31.5	0.4396	0.2975	0.7840	0.1464	0.0949	101.5	1275	72.8	1.35	0.0070	1321	25	1179	46.8	0.38	1.20	422	46	1454	42.8	1.93	0.0003	759	1364	34.5	0.56	1.10	547
31.5	0.4436	0.2985	0.7746	0.1450	0.0949	98.5	1274	65.4	1.35	0.0120	1244	10	1178	57.3	0.37	1.28	444	48	1451	58.2	1.81	0.0011	761	1361	44.3	0.54	1.07	537
31.5	0.4556	0.2967	0.8870	0.1454	0.0981	100.0	1308	79.7	4.58	0.0070	1401	0	1224	66.3	0.69	0.84	493	3	1519	61.8	21.44	0.0000	986	1447	61.6	1.54	0.07	758
31.5	0.4342	0.2965	0.8244	0.1469	0.0965	96.8	1286	71.7	1.37	0.0020	1320	14	1188	57.2	0.39	1.19	426	44	1471	55.7	1.92	0.0000	782	1381	42.2	0.57	0.98	590
31.5	0.4346	0.2970	0.7376	0.1460	0.0966	94.9	1285	65.4	1.42	0.0060	1263	36	1186	58.3	0.39	1.25	418	51	1472	61.7	1.96	0.0012	773	1380	44.1	0.57	1.15	533
31.5	0.4639	0.2973	0.7082	0.1462	0.0943	98.5	1294	96.0	3.27	0.0050	1309	0	1207	60.7	0.57	0.95	454	18	1496	76.4	9.95	0.0000	833	1417	61.6	1.10	0.28	605
31.5	0.4343	0.2961	0.8258	0.1467	0.0963	94.5	1291	70.6	1.28	0.0060	1309	0	1192	62.3	0.38	1.26	449	47	1478	56.9	1.71	0.0000	813	1387	47.3	0.55	0.98	587
31.5	0.4373	0.2961	0.7482	0.1466	0.0963	94.9	1291	80.0	1.54	0.0100	1315	47	1194	62.2	0.41	1.25	468	43	1484	61.1	2.32	0.0010	805	1396	50.6	0.62	0.92	570
31.5	0.4560	0.2984	0.8426	0.1475	0.0947	94.7	1277	87.2	1.61	0.0040	1330	0	1186	66.5	0.42	0.99	450	37	1458	72.0	2.32	0.0014	820	1374	56.7	0.65	0.69	576
31.5	0.4414	0.2980	0.7782	0.1454	0.0980	96.3	1284	82.2	2.10	0.0040	1275	0	1196	61.9	0.47	0.92	451	27	1480	81.9	3.87	0.0000	843	1398	56.2	0.80	0.44	609
31.5	0.4494	0.2991	0.8448	0.1461	0.0983	98.0	1277	74.3	2.62	0.0040	1340	0	1196	68.3	0.52	0.81	500	13	1469	75.4	6.17	0.0006	884	1393	58.7	0.97	0.19	660
31.5	0.4605	0.2987	0.8448	0.1463	0.0959	94.5	1286	91.7	2.13	0.0040	1287	0	1199	76.6	0.49	0.90	487	17	1475	83.9	4.42	0.0007	896	1397	68.9	0.87	0.31	675
31.5	0.4536	0.2975	0.8786	0.1462	0.0989	97.8	1299	88.4	4.54	0.0050	1367	0	1215	69.8	0.66	0.83	524	6	1505	78.0	16.47	0.0000	912	1432	67.7	1.42	0.09	774
31.5	0.4420	0.2987	0.8482	0.1463	0.0971	101.0	1274	70.6	1.83	0.0050	1254	0	1184	58.0	0.44	0.97	452	21	1456	54.6	3.18	0.0002	856	1373	36.8	0.71	0.48	621
31.5	0.4463	0.2988	0.8126	0.1472	0.0948	99.8	1266	66.9	1.38	0.0100	1264	47	1173	52.6	0.38	1.13	450	43	1438	51.2	1.90	0.0000	785	1351	36.8	0.57	1.01	553
31.5	0.4514	0.2984	0.7078	0.1461	0.0952	98.5	1274	77.8	1.99	0.0050	1301	0	1188	61.1	0.45	1.03	460	28	1464	59.7	3.95	0.0000	758	1381	54.7	0.76	0.60	562
31.5	0.4548	0.2961	0.8484	0.1476	0.0942	99.5	1297	74.3	2.06	0.0040	1448	0	1206	65.9	0.46	0.97	465	25	1488	67.7	3.70	0.0000	857	1406	51.1	0.79	0.39	667
31.5	0.4427	0.2994	0.7304	0.1476	0.0989	101.4	1263	62.5	2.89	0.0030	1254	0	1184	50.2	0.55	0.82	449	17	1454	54.2	8.97	0.0000	840	1378	45.0	1.05	0.26	625
31.5	0.4357	0.2988	0.7114	0.1455	0.0976	96.6	1270	76.0	1.64	0.0040	1198	77	1181	58.6	0.41	1.16	458	45	1456	63.3	2.58	0.0000	732	1370	47.9	0.64	0.83	566
31.5	0.4543	0.2978	0.7026	0.1450	0.0953	95.9	1291	79.3	2.30	0.0040	1284	0	1200	70.4	0.49	0.97	451	32	1487	89.8	4.58	0.0000	827	1405	66.9	0.85	0.49	606
31.5	0.4650	0.2997	0.7542	0.1471	0.0979	97.5	1277	91.7	4.69	0.0050	1266	0	1203	76.2	0.72	0.82	471	5	1478	45.8	31.10	0.0000	935	1409	83.3	1.70	0.09	727
31.5	0.4366	0.2983	0.7888	0.1462	0.0988	95.3	1274	81.4	1.85	0.0050	1283	9	1190	64.5	0.45	0.97	452	28	1469	75.0	3.35	0.0000	849	1385	47.1	0.74	0.49	631
31.5	0.4341	0.2979	0.7368	0.1477	0.0989	101.1	1271	72.3	2.39	0.0040	1226	0	1186	55.0	0.50	0.90	439	30	1464	50.5	5.31	0.0000	776	1383	41.4	0.87	0.34	630
31.5	0.4328	0.2998	0.8448	0.1478	0.0967	95.1	1250	73.1	1.02	0.0100	1234	60	1160	53.0	0.33	1.36	422	59	1413	55.6	1.15	0.0015	718	1318	42.2	0.44	1.72	564
31.5	0.4430	0.2972	0.8764	0.1473	0.0951	99.6	1282	67.4	1.46	0.0060	1403	64	1187	53.8	0.40	1.14	451	39	1461	49.8	2.03	0.0009	817	1375	38.4	0.59	0.76	561
31.5	0.4338	0.2989	0.8538	0.1457	0.0970	94.9	1269	78.6	1.12	0.0070	1259	41	1175	58.1	0.35	1.31	443	53	1441	59.1	1.40	0.0006	765	1350	46.7	0.50	1.31	571
31.5	0.4397	0.2978	0.7160	0.1461	0.0988	97.3	1282	84.7	2.74	0.0060	1226	0	1198	62.4	0.54	0.91	459	20	1485	73.0	7.46	0.0000	816	1406	56.0	0.99	0.28	612
31.5	0.4478	0.2979	0.7418	0.1458	0.0976	98.2	1285	72.1	2.84	0.0070	1244	0	1201	60.6	0.55	0.86	486	21	1487	77.4	7.61	0.0000	830	1410	58.2	1.04	0.28	642
31.5	0.4511	0.2984	0.8790	0.1465	0.0957	97.0	1281	81.2	1.68	0.0050	1346	0	1190	60.9	0.43	0.95	477	29	1463	65.7	2.69	0.0003	831	1379	53.6	0.69	0.49	654
31.5	0.4579	0.2964	0.8816	0.1480	0.0965	100.1	1301	81.2	3.86	0.0080	1379	0	1215	63.7	0.62	0.87	478	6	1501	90.4	11.08	0.0000	980	1428	57.4	1.22	0.14	694
31.5	0.4604	0.2972	0.8822	0.1458	0.0949	96.5	1300	82.8	2.35	0.0050	1406	0	1210	73.4	0.49	0.93	463	17	1493	84.5	4.45	0.0000	913	1414	67.3	0.85	0.29	721
31.5	0.4589	0.2984	0.8568	0.1478	0.0974	97.1	1285	106.8	3.18	0.0040	1324	0	1202	78.2	0.59	0.87	493	10	1480	87.7	9.92	0.0000	927	1406	74.9	1.17	0.13	728

Continuation of Table S1.

c_2	q_H	σ_{OO}	ϵ_{OO}	d_{OO}	d_{OH}	a_{HOO}	ρ	ϵ	μ	P_v	λ	wt%$_v$	ρ	ϵ	μ	P_v	λ	wt%$_v$	ρ	ϵ	μ	P_v	λ	ρ	ϵ	μ	P_v	λ
31.5	0.4473	0.2984	0.8954	0.1471	0.0965	100.9	1282	91.1	3.24	0.0020	1301	0	1202	70.8	0.59	0.82	489	10	1480	87.7	10.08	0.0000	899	1406	62.1	1.16	0.14	694
31.5	0.4385	0.2988	0.8182	0.1464	0.0950	96.8	1265	68.4	1.04	0.0080	1216	38	1167	54.6	0.33	1.53	426	53	1430	54.7	1.21	0.0018	768	1337	35.4	0.45	1.66	527
31.5	0.4636	0.2971	0.7476	0.1472	0.0969	99.9	1300	76.0	6.77	0.0080	1342	0	1217	64.8	0.78	0.91	477	5	1509	42.7	54.40	0.0000	894	1439	69.1	1.97	0.08	743
31.5	0.4488	0.2999	0.7564	0.1460	0.0967	94.4	1264	86.4	1.69	0.0050	1259	0	1181	67.2	0.43	1.01	461	33	1447	78.9	2.74	0.0006	799	1365	57.8	0.68	0.64	554
31.5	0.4590	0.2965	0.8074	0.1474	0.0942	95.6	1298	88.4	2.07	0.0040	1391	0	1205	66.0	0.48	1.07	468	24	1491	74.3	3.77	0.0012	860	1409	65.4	0.80	0.48	632
31.5	0.4436	0.2960	0.7026	0.1477	0.0941	101.8	1286	57.2	1.76	0.0040	1293	0	1189	47.6	0.42	1.16	404	38	1473	46.8	2.79	0.0028	748	1385	34.8	0.65	0.84	566
31.5	0.4349	0.2991	0.8246	0.1462	0.0988	97.8	1267	68.5	1.72	0.0040	1218	27	1183	55.3	0.44	0.89	456	27	1453	57.1	2.83	0.0000	835	1370	44.3	0.68	0.56	608
31.5	0.4476	0.2996	0.8424	0.1458	0.0981	96.0	1272	84.0	2.09	0.0040	1270	0	1191	59.9	0.47	0.88	485	21	1462	74.0	3.98	0.0000	849	1382	61.7	0.82	0.33	656
31.5	0.4372	0.2967	0.7590	0.1469	0.0971	100.1	1287	67.0	2.09	0.0070	1275	0	1193	49.5	0.46	1.00	449	25	1479	48.2	3.63	0.0000	800	1395	40.9	0.75	0.57	553
31.5	0.4437	0.2973	0.7814	0.1465	0.0967	100.4	1287	69.1	2.22	0.0100	1283	12	1193	56.0	0.48	0.98	474	23	1476	49.7	4.35	0.0000	831	1396	45.9	0.82	0.45	619
31.5	0.4535	0.2967	0.7774	0.1467	0.0961	94.9	1297	110.8	2.53	0.0070	1340	0	1207	66.5	0.52	0.92	480	22	1500	89.7	5.54	0.0000	854	1419	58.8	0.91	0.34	647
31.5	0.4441	0.2999	0.7876	0.1477	0.0974	94.3	1259	93.5	1.56	0.0030	1186	0	1175	65.3	0.41	0.96	430	39	1437	66.6	2.34	0.0002	791	1353	56.7	0.63	0.73	570
31.5	0.4442	0.2969	0.8806	0.1461	0.0943	99.4	1286	70.3	1.35	0.0060	1370	32	1192	49.1	0.38	1.31	446	38	1467	47.3	1.75	0.0002	829	1378	39.9	0.56	0.95	601
31.5	0.4513	0.2962	0.7162	0.1450	0.0943	97.0	1301	84.2	2.05	0.0050	1353	0	1206	62.4	0.46	1.03	425	34	1499	67.5	3.74	0.0000	781	1414	50.4	0.76	0.69	559
31.5	0.4335	0.2979	0.8606	0.1468	0.0975	100.3	1275	71.2	1.53	0.0050	1248	19	1182	52.3	0.40	1.03	445	31	1456	44.0	2.31	0.0002	826	1370	40.4	0.62	0.66	590
31.5	0.4523	0.2969	0.7738	0.1454	0.0964	95.3	1302	93.7	2.59	0.0020	1373	0	1209	65.3	0.52	0.90	468	22	1504	85.8	5.76	0.0000	841	1423	60.4	0.93	0.27	665
31.5	0.4322	0.2987	0.7624	0.1472	0.0990	99.4	1264	70.4	1.89	0.0050	1212	0	1179	52.0	0.44	0.92	462	28	1452	55.8	3.28	0.0000	832	1368	44.4	0.72	0.57	574
31.5	0.4538	0.2984	0.7734	0.1470	0.0949	100.6	1274	69.2	2.00	0.0060	1262	0	1185	52.6	0.45	1.05	464	27	1459	50.2	3.84	0.0000	826	1377	44.0	0.77	0.50	594
31.5	0.4529	0.2971	0.7046	0.1469	0.0986	96.4	1299	92.7	5.23	0.0040	1270	0	1217	79.7	0.75	0.78	483	9	1512	112.1	33.23	0.0000	908	1441	79.7	1.76	0.11	700
31.5	0.4435	0.2975	0.8046	0.1465	0.0975	99.0	1289	77.6	2.34	0.0040	1268	0	1197	56.8	0.50	0.87	502	20	1481	61.5	4.89	0.0000	841	1400	44.6	0.86	0.32	647
31.5	0.4559	0.3000	0.7580	0.1480	0.0989	99.3	1269	80.7	4.30	0.0040	1336	0	1194	66.2	0.66	0.76	506	6	1462	77.0	21.75	0.0000	882	1392	58.1	1.50	0.10	687
31.5	0.4352	0.2980	0.7840	0.1460	0.0976	100.6	1277	66.6	1.70	0.0130	1322	25	1185	51.4	0.43	1.04	447	35	1462	48.2	2.97	0.0014	805	1377	38.8	0.68	0.62	572
31.5	0.4463	0.2980	0.7386	0.1462	0.0986	98.2	1285	68.9	3.60	0.0040	1228	0	1202	62.7	0.60	0.80	469	15	1487	61.5	10.96	0.0000	887	1413	61.4	1.16	0.20	650
31.5	0.4454	0.2980	0.8600	0.1474	0.0977	99.2	1281	89.6	2.33	0.0050	1239	0	1194	59.2	0.49	0.90	492	23	1471	53.3	4.71	0.0000	890	1392	51.4	0.88	0.31	670
33	0.4303	0.2994	0.7996	0.1474	0.0983	95.5	1256	68.9	1.23	0.0040	600	10	1167	48.3	0.36	1.16	443	52	1429	51.4	1.62	0.0007	780	1338	44.0	0.52	1.20	530
33	0.4306	0.2983	0.8700	0.1474	0.0980	96.6	1271	68.5	1.30	0.0060	633	0	1176	47.4	0.37	1.15	479	45	1445	49.3	1.70	0.0008	836	1355	38.9	0.53	0.98	613
33	0.4317	0.2967	0.7368	0.1461	0.0958	99.6	1281	62.6	1.29	0.0060	643	7	1181	50.8	0.37	1.38	423	49	1463	42.4	1.78	0.0038	745	1370	32.9	0.53	1.28	519
33	0.4320	0.2963	0.8210	0.1457	0.0949	94.6	1285	63.0	0.97	0.0140	689	53	1181	48.1	0.32	1.79	407	63	1462	49.0	1.07	0.0051	779	1364	35.9	0.43	2.22	498
33	0.4321	0.2967	0.8492	0.1455	0.0973	101.4	1290	64.2	1.63	0.0090	654	55	1193	48.4	0.42	1.00	461	33	1477	47.4	2.54	0.0005	855	1391	32.9	0.65	0.65	570
33	0.4327	0.2996	0.7096	0.1465	0.0957	101.4	1248	55.6	1.11	0.0050	615	34	1157	41.6	0.34	1.39	419	61	1415	36.8	1.39	0.0013	712	1320	27.1	0.45	1.86	481
33	0.4338	0.2993	0.8600	0.1454	0.0950	101.8	1260	54.1	0.99	0.0140	669	51	1163	43.2	0.33	1.46	452	57	1421	34.9	1.11	0.0030	749	1326	27.6	0.43	1.81	508
33	0.4350	0.2960	0.8874	0.1477	0.0987	94.0	1298	87.7	1.94	0.0120	684	0	1203	59.9	0.46	1.00	479	23	1493	76.0	3.07	0.0000	876	1409	54.7	0.73	0.47	654
33	0.4354	0.2988	0.8302	0.1459	0.0967	101.5	1267	56.3	1.39	0.0050	607	46	1175	45.8	0.38	1.11	429	42	1442	38.0	1.97	0.0015	792	1355	30.8	0.56	0.99	581
33	0.4355	0.2999	0.7450	0.1465	0.0978	96.2	1253	66.2	1.37	0.0100	596	42	1169	48.3	0.38	1.06	430	48	1431	54.9	2.01	0.0000	734	1342	43.6	0.55	1.11	539
33	0.4358	0.2987	0.8246	0.1455	0.0980	101.9	1273	64.0	1.74	0.0100	639	0	1184	48.5	0.44	0.90	432	31	1457	42.4	2.92	0.0000	815	1374	37.3	0.70	0.55	594
33	0.4360	0.2976	0.7012	0.1471	0.0945	98.1	1266	66.1	1.11	0.0060	583	68	1167	49.7	0.34	1.63	376	63	1439	45.1	1.36	0.0019	696	1342	35.3	0.45	2.19	505
33	0.4362	0.2973	0.7760	0.1464	0.0959	96.8	1277	67.5	1.30	0.0050	629	0	1180	49.7	0.37	1.32	434	50	1459	58.5	1.71	0.0016	750	1368	38.0	0.53	1.33	540
33	0.4363	0.2987	0.7884	0.1450	0.0989	100.9	1275	60.2	2.16	0.0040	588	0	1189	51.5	0.47	0.89	473	22	1467	49.4	4.42	0.0000	816	1386	41.3	0.81	0.38	635
33	0.4364	0.2966	0.8144	0.1473	0.0974	97.2	1288	70.8	1.77	0.0050	652	0	1191	53.2	0.43	1.05	460	34	1477	55.8	2.90	0.0008	847	1392	46.0	0.67	0.63	604
33	0.4366	0.2979	0.7832	0.1454	0.0980	99.4	1280	66.1	1.98	0.0030	630	11	1190	54.5	0.45	0.96	442	28	1472	52.0	3.43	0.0062	828	1388	37.7	0.74	0.48	599
33	0.4388	0.2979	0.8354	0.1472	0.0973	95.9	1277	65.7	1.51	0.0040	613	0	1186	56.4	0.41	0.88	443	34	1460	66.4	2.27	0.0010	809	1375	48.7	0.63	0.72	607
33	0.4389	0.2982	0.7426	0.1462	0.0952	96.8	1270	65.1	1.19	0.0070	582	5	1172	50.2	0.35	1.53	407	58	1445	50.1	1.52	0.0029	713	1351	38.1	0.48	1.66	526
33	0.4390	0.2961	0.8408	0.1480	0.0941	96.0	1283	69.7	1.11	0.0050	670	0	1182	49.6	0.34	1.56	411	56	1459	53.3	1.26	0.0067	780	1364	36.4	0.47	1.81	569
33	0.4392	0.2991	0.8484	0.1465	0.0946	101.6	1260	58.2	1.05	0.0070	621	68	1164	52.2	0.34	1.38	439	54	1424	32.7	1.29	0.0000	789	1332	31.3	0.46	1.35	536
33	0.4394	0.2963	0.7914	0.1477	0.0951	96.4	1283	72.6	1.31	0.0090	659	78	1184	54.0	0.37	1.35	435	49	1466	54.6	1.74	0.0004	788	1375	42.6	0.54	1.25	531
33	0.4396	0.2986	0.7602	0.1454	0.0972	100.3	1273	60.3	1.77	0.0010	610	0	1183	52.1	0.43	0.99	468	26	1459	50.1	3.21	0.0007	785	1375	41.2	0.70	0.60	598
33	0.4404	0.2995	0.7142	0.1467	0.0986	101.9	1262	64.2	2.58	0.0010	585	0	1182	50.3	0.51	0.82	462	17	1452	49.8	6.60	0.0000	800	1374	39.9	0.93	0.36	620
33	0.4420	0.2990	0.7942	0.1466	0.0953	101.0	1263	59.9	1.34	0.0080	639	34	1170	45.5	0.37	1.16	447	48	1435	43.4	1.85	0.0005	776	1346	32.6	0.55	1.03	541
33	0.4424	0.2990	0.7184	0.1455	0.0975	101.5	1268	63.6	2.27	0.0030	639	0	1186	47.1	0.47	0.91	457	25	1460	48.7	4.87	0.0000	778	1380	37.9	0.85	0.46	582
33	0.4428	0.2962	0.7100	0.1461	0.0962	95.9	1297	83.1	2.09	0.0020	645	0	1201	61.7	0.46	1.04	447	39	1494	62.0	3.68	0.0004	781	1409	52.3	0.77	0.63	563

Continuation of Table S1.

c_2	q_H	σ_{OO}	ϵ_{OO}	d_{OO}	d_{OH}	a_{HOO}	ρ	ϵ	μ	P_v	λ	wt%$_v$	ρ	ϵ	μ	P_v	λ	wt%$_v$	ρ	ϵ	μ	P_v	λ	ρ	ϵ	μ	P_v	λ
33	0.4430	0.2985	0.8594	0.1465	0.0956	100.8	1273	56.6	1.42	0.0070	654	0	1179	45.0	0.38	1.11	425	37	1448	43.8	2.08	0.0011	819	1362	34.3	0.60	0.81	604
33	0.4434	0.2963	0.7312	0.1455	0.0945	100.6	1291	68.7	1.66	0.0070	639	15	1192	50.4	0.41	1.23	432	43	1480	50.2	2.58	0.0013	755	1392	35.7	0.64	0.84	503
33	0.4436	0.2977	0.7004	0.1467	0.0940	94.7	1272	74.8	1.17	0.0170	617	62	1172	54.2	0.35	1.59	400	62	1448	69.7	1.47	0.0033	730	1353	41.8	0.47	2.07	451
33	0.4437	0.2985	0.8514	0.1464	0.0945	97.8	1270	68.4	1.14	0.0050	661	18	1174	54.4	0.35	1.32	419	46	1440	53.1	1.39	0.0018	795	1349	37.5	0.49	1.34	563
33	0.4439	0.2964	0.7928	0.1467	0.0952	94.1	1290	76.3	1.42	0.0060	631	0	1192	59.7	0.40	1.23	467	46	1479	62.9	2.04	0.0003	853	1388	47.2	0.58	0.99	557
33	0.4442	0.2961	0.8044	0.1467	0.0978	95.5	1303	85.4	2.66	0.0060	723	0	1210	64.1	0.52	0.92	459	21	1505	76.7	5.48	0.0000	883	1426	57.4	0.95	0.32	636
33	0.4450	0.2996	0.8968	0.1455	0.0952	94.9	1266	72.1	1.07	0.0070	640	24	1174	52.5	0.34	1.15	436	51	1433	59.6	1.32	0.0021	794	1343	41.7	0.48	1.27	569
33	0.4451	0.2975	0.7220	0.1467	0.0942	96.7	1276	75.1	1.35	0.0010	648	0	1178	49.6	0.37	1.29	416	57	1455	55.7	1.84	0.0014	763	1363	41.0	0.53	1.42	523
33	0.4460	0.2990	0.8338	0.1466	0.0979	96.1	1273	80.9	2.01	0.0020	637	0	1189	62.1	0.46	0.90	447	23	1461	60.9	3.69	0.0000	833	1380	55.6	0.77	0.40	613
33	0.4467	0.2991	0.7014	0.1467	0.0983	100.9	1270	75.0	3.17	0.0050	611	0	1191	54.6	0.57	0.71	478	14	1466	63.6	11.84	0.0000	831	1390	49.4	1.14	0.23	610
33	0.4469	0.2990	0.7930	0.1457	0.0986	100.6	1276	69.2	3.01	0.0040	651	0	1195	55.1	0.56	0.65	474	14	1473	59.2	9.12	0.0000	902	1396	46.8	1.08	0.20	657
33	0.4479	0.2964	0.8102	0.1466	0.0945	99.0	1291	74.1	1.61	0.0040	668	0	1195	54.4	0.42	1.17	446	34	1480	51.2	2.52	0.0000	805	1393	42.8	0.65	0.79	585
33	0.4484	0.2962	0.8872	0.1458	0.0961	100.7	1303	63.4	2.32	0.0080	740	0	1212	53.3	0.51	1.06	463	17	1500	62.8	4.58	0.0000	906	1420	42.1	0.87	0.26	662
33	0.4484	0.2969	0.8118	0.1472	0.0966	101.7	1291	65.4	2.71	0.0050	676	0	1200	49.3	0.52	0.92	475	16	1486	49.1	6.60	0.0000	864	1407	41.5	0.95	0.31	649
33	0.4490	0.3000	0.8588	0.1468	0.0968	97.8	1264	72.9	1.62	0.0100	649	42	1179	55.4	0.43	0.86	472	30	1441	59.9	2.82	0.0019	844	1359	44.2	0.68	0.51	643
33	0.4496	0.2967	0.8944	0.1452	0.0950	99.8	1297	71.5	1.75	0.0040	723	0	1208	52.3	0.45	1.05	481	25	1488	55.0	2.90	0.0022	867	1405	40.6	0.72	0.45	679
33	0.4499	0.2994	0.7998	0.1466	0.0953	95.8	1264	83.3	1.37	0.0030	631	18	1176	59.4	0.38	1.10	445	44	1441	64.1	1.99	0.0019	808	1354	45.8	0.58	0.92	573
33	0.4503	0.2979	0.7068	0.1469	0.0975	98.9	1284	76.5	3.52	0.0060	675	0	1200	59.8	0.60	0.84	460	11	1485	59.9	12.14	0.0005	845	1408	53.3	1.19	0.23	668
33	0.4508	0.2977	0.7222	0.1476	0.0951	96.1	1280	73.7	1.89	0.0040	617	0	1187	55.5	0.43	1.12	432	39	1466	61.9	3.21	0.0000	774	1381	50.5	0.71	0.84	532
33	0.4508	0.2998	0.8078	0.1480	0.0971	95.8	1264	83.5	1.89	0.0030	650	0	1180	65.7	0.45	0.87	519	32	1443	71.4	3.43	0.0000	823	1362	52.8	0.74	0.52	622
33	0.4525	0.2966	0.7590	0.1452	0.0956	97.5	1298	75.5	2.48	0.0060	623	7	1208	56.7	0.51	0.97	477	21	1501	63.0	5.34	0.0000	838	1420	55.4	0.89	0.40	613
33	0.4536	0.2965	0.7850	0.1468	0.0944	98.0	1294	74.6	1.94	0.0080	663	0	1201	54.6	0.46	1.09	439	29	1486	60.9	3.42	0.0003	824	1402	47.0	0.74	0.64	606
33	0.4544	0.2971	0.7428	0.1467	0.0951	98.0	1291	82.8	2.23	0.0050	668	0	1198	65.6	0.49	0.94	460	31	1485	65.3	4.81	0.0004	831	1403	50.5	0.86	0.42	615
33	0.4546	0.2990	0.8672	0.1458	0.0956	100.8	1277	68.2	1.97	0.0040	665	0	1189	58.1	0.45	0.98	475	23	1460	51.8	3.74	0.0003	856	1380	42.7	0.77	0.36	684
33	0.4547	0.2985	0.8678	0.1476	0.0965	99.2	1280	81.1	2.51	0.0030	679	0	1193	54.6	0.50	0.92	483	16	1467	61.7	5.29	0.0000	923	1388	47.7	0.90	0.31	609
33	0.4550	0.2972	0.8890	0.1459	0.0951	96.1	1296	74.3	1.76	0.0100	698	0	1203	61.5	0.45	0.98	485	28	1485	77.3	3.07	0.0013	852	1403	57.6	0.74	0.51	634
33	0.4557	0.2999	0.7456	0.1457	0.0954	94.2	1266	84.7	1.67	0.0070	628	13	1182	63.2	0.42	1.06	448	36	1449	76.8	2.73	0.0005	780	1365	58.2	0.66	0.80	597
33	0.4561	0.2979	0.8330	0.1478	0.0975	101.9	1284	59.5	3.99	0.0060	675	0	1202	52.9	0.62	0.83	484	4	1482	66.4	16.97	0.0000	885	1409	51.1	1.31	0.12	705
33	0.4564	0.2963	0.7082	0.1477	0.0974	98.6	1304	77.5	6.65	0.0030	658	0	1219	71.7	0.77	0.85	493	6	1516	40.1	36.85	0.0000	859	1443	62.9	1.79	0.18	725
33	0.4564	0.2990	0.7912	0.1463	0.0988	94.7	1284	106.2	3.34	0.0050	651	0	1206	73.7	0.63	0.87	500	12	1487	101.3	13.20	0.0000	938	1414	76.6	1.34	0.15	735
33	0.4568	0.2988	0.7902	0.1462	0.0970	100.6	1279	80.8	3.29	0.0060	617	0	1196	53.7	0.54	0.86	506	13	1475	58.8	10.72	0.0000	884	1400	50.0	1.17	0.15	658
33	0.4568	0.2991	0.7956	0.1456	0.0977	97.0	1283	70.0	2.59	0.0050	624	0	1195	51.4	0.51	0.93	459	25	1475	50.0	5.99	0.0000	832	1395	43.4	0.92	0.41	659
33	0.4570	0.2994	0.8862	0.1455	0.0959	94.9	1280	84.6	1.73	0.0060	655	28	1192	65.3	0.44	0.92	493	20	1461	71.8	2.95	0.0000	880	1380	55.7	0.72	0.41	648
33	0.4582	0.2963	0.7976	0.1466	0.0972	99.5	1304	74.9	5.28	0.0030	699	0	1222	63.7	0.71	0.88	478	5	1517	56.8	24.65	0.0000	900	1444	57.6	1.60	0.08	687
33	0.4588	0.2973	0.8034	0.1465	0.0963	97.2	1294	83.5	3.23	0.0040	675	0	1209	63.0	0.57	0.94	493	12	1497	63.4	9.16	0.0005	853	1420	55.8	1.10	0.21	689
33	0.4595	0.2964	0.7412	0.1451	0.0956	94.7	1310	88.0	3.24	0.0070	661	0	1219	71.1	0.57	0.93	491	18	1518	87.8	8.07	0.0000	848	1440	71.3	1.10	0.24	609
33	0.4601	0.2971	0.7000	0.1458	0.0957	102.0	1296	75.0	4.94	0.0040	636	0	1211	55.5	0.66	0.79	475	11	1502	81.4	24.36	0.0000	815	1427	47.9	1.45	0.16	662
33	0.4612	0.2988	0.8146	0.1460	0.0953	98.2	1281	75.6	2.49	0.0030	639	0	1194	54.3	0.49	0.89	462	20	1470	65.2	4.92	0.0000	832	1391	53.9	0.87	0.29	632
33	0.4628	0.2986	0.8322	0.1461	0.0972	95.5	1289	99.4	3.23	0.0090	649	7	1209	70.9	0.61	0.91	476	4	1491	84.7	11.62	0.0000	879	1417	80.3	1.22	0.15	747
33	0.4651	0.2997	0.8704	0.1478	0.0972	98.6	1276	83.8	3.35	0.0080	652	0	1197	63.5	0.59	0.80	505	12	1464	73.1	11.94	0.0000	916	1392	60.9	1.24	0.19	725
33	0.4653	0.2980	0.7496	0.1468	0.0965	96.6	1293	80.7	4.51	0.0040	643	0	1211	72.2	0.67	0.82	501	7	1498	66.1	19.64	0.0000	902	1425	69.3	1.49	0.12	696
33	0.4654	0.2965	0.7974	0.1476	0.0960	99.6	1301	99.9	5.13	0.0060	692	0	1217	65.5	0.72	0.93	471	7	1509	64.4	24.19	0.0000	947	1437	53.7	1.58	0.12	739
33	0.4668	0.2980	0.8550	0.1470	0.0973	100.6	1297	93.2	6.16	0.0030	663	0	1214	62.0	0.75	0.90	470	4	1498	61.6	33.61	0.0000	941	1428	59.0	1.86	0.05	759
33	0.4672	0.2987	0.7776	0.1470	0.0974	95.1	1289	100.5	4.80	0.0060	665	0	1210	78.8	0.70	0.73	476	4	1494	78.7	24.34	0.0000	901	1423	85.5	1.59	0.09	738
33	0.4678	0.2998	0.8456	0.1457	0.0969	96.2	1281	93.9	3.33	0.0030	635	0	1204	82.2	0.61	0.81	523	11	1479	86.0	12.01	0.0000	919	1405	70.5	1.22	0.12	729
33	0.4687	0.2998	0.7632	0.1454	0.0982	95.0	1288	104.0	5.29	0.0030	662	0	1213	83.6	0.77	0.74	516	6	1494	78.1	35.28	0.0000	908	1425	90.5	1.86	0.09	722
33	0.4689	0.2991	0.8898	0.1454	0.0959	95.0	1292	97.0	2.83	0.0080	659	0	1207	74.1	0.56	0.82	490	15	1484	96.4	7.10	0.0000	928	1409	76.7	1.05	0.15	740
33	0.4694	0.2960	0.7520	0.1475	0.0945	94.9	1310	103.0	4.04	0.0030	695	26	1221	83.3	0.64	0.94	447	10	1517	94.9	12.75	0.0002	907	1441	81.3	1.27	0.21	668
33	0.4694	0.2984	0.8406	0.1465	0.0979	95.3	1295	79.8	5.53	0.0050	673	0	1219	76.8	0.79	0.73	502	10	1505	93.9	34.63	0.0000	1005	1436	92.5	1.90	0.08	784
33	0.4696	0.2972	0.7804	0.1475	0.0973	98.3	1302	79.3	8.93	0.0050	648	0	1222	66.5	0.86	0.85	501	3	1516	41.2	143.05	0.0000	924	1448	77.3	2.42	0.05	796

Continuation of Table S1.

c_2	q_H	σ_{OO}	ϵ_{OO}	d_{OO}	d_{OH}	a_{HOO}	ρ	ϵ	μ	P_v	λ	wt%$_v$	ρ	ϵ	μ	P_v	λ	wt%$_v$	ρ	ϵ	μ	P_v	λ	ρ	ϵ	μ	P_v	λ
33	0.4474	0.2965	0.7594	0.1450	0.0955	99.4	1297	69.7	2.23	0.0030	1349	21	1203	52.5	0.48	1.07	473	25	1494	58.0	3.83	0.0000	809	1411	45.9	0.78	0.48	607
33	0.4496	0.2986	0.8438	0.1480	0.0973	96.3	1274	83.0	2.09	0.0990	1298	0	1189	64.0	0.47	0.84	507	18	1461	64.8	4.11	0.0000	861	1381	52.8	0.80	0.34	617
33	0.4315	0.2964	0.7084	0.1451	0.0957	96.3	1284	63.4	1.24	0.0070	1266	54	1183	57.9	0.36	1.57	385	60	1471	52.3	1.58	0.0006	745	1376	37.8	0.49	1.74	548
33	0.4325	0.2990	0.7230	0.1480	0.0948	100.2	1249	57.2	0.96	0.0100	1153	76	1151	38.5	0.31	1.62	408	67	1408	38.6	1.13	0.0086	689	1310	27.2	0.41	2.49	487
33	0.4520	0.2975	0.8900	0.1470	0.0965	98.4	1288	75.1	2.20	0.0060	1286	0	1201	56.7	0.50	0.88	456	16	1480	58.9	4.53	0.0000	903	1401	50.3	0.87	0.30	680
33	0.4516	0.2968	0.7314	0.1469	0.0952	94.1	1290	87.3	1.99	0.0090	1268	33	1198	63.4	0.44	1.10	434	35	1485	68.5	3.37	0.0000	797	1401	56.1	0.74	0.76	558
33	0.4684	0.2988	0.8600	0.1471	0.0956	95.3	1286	100.1	2.74	0.0060	1302	0	1201	71.8	0.54	0.86	466	12	1477	87.2	7.25	0.0000	904	1401	70.6	1.04	0.24	696
33	0.4342	0.2976	0.8770	0.1469	0.0971	99.7	1279	59.7	1.45	0.0040	1299	0	1181	40.5	0.38	1.06	445	36	1457	44.2	1.97	0.0000	822	1371	36.9	0.60	0.82	608
33	0.4560	0.2961	0.7344	0.1456	0.0981	96.4	1313	92.8	6.52	0.0070	1382	0	1227	58.7	0.74	0.83	483	7	1536	90.9	46.37	0.0000	856	1462	73.2	1.88	0.11	752
33	0.4500	0.2963	0.7758	0.1471	0.0979	101.5	1300	73.8	4.83	0.0060	1353	0	1212	59.9	0.68	0.93	450	8	1508	42.0	19.59	0.0000	900	1434	46.1	1.44	0.13	697
33	0.4560	0.2981	0.7048	0.1475	0.0957	97.0	1279	82.5	2.58	0.0060	1264	0	1194	70.5	0.52	0.91	464	25	1474	62.9	6.21	0.0000	815	1394	53.5	0.95	0.39	596
33	0.4514	0.2988	0.7022	0.1458	0.0970	101.3	1274	80.3	3.13	0.0030	1258	0	1194	49.9	0.56	0.79	477	16	1474	59.9	10.49	0.0000	794	1396	49.2	1.10	0.25	661
33	0.4590	0.2997	0.8552	0.1461	0.0955	96.8	1273	78.0	1.80	0.0050	1275	0	1188	61.4	0.45	0.97	467	28	1454	67.1	3.30	0.0000	854	1374	55.4	0.76	0.49	679
33	0.4688	0.2977	0.7842	0.1458	0.0962	98.9	1299	70.5	6.26	0.0040	1401	0	1217	68.8	0.72	0.83	526	7	1508	70.0	32.10	0.0000	970	1436	62.7	1.68	0.08	716
33	0.4370	0.2965	0.7594	0.1475	0.0959	98.3	1280	54.4	1.52	0.0030	1277	62	1185	53.6	0.40	1.17	419	40	1468	47.8	2.21	0.0008	761	1379	38.4	0.60	0.98	549
33	0.4429	0.2968	0.8844	0.1467	0.0942	96.3	1283	64.9	1.05	0.0070	1423	32	1189	54.4	0.36	1.32	429	49	1462	54.4	1.40	0.0000	829	1370	37.8	0.50	1.33	600
33	0.4448	0.2969	0.8946	0.1480	0.0958	94.9	1280	72.1	1.40	0.0060	1352	22	1194	62.5	0.40	1.13	464	38	1469	60.8	2.01	0.0004	839	1383	43.4	0.62	0.62	611
33	0.4306	0.2980	0.8100	0.1453	0.0947	101.9	1267	52.9	0.98	0.0110	1285	67	1167	48.8	0.32	1.69	448	64	1435	32.6	1.10	0.0057	756	1339	27.1	0.43	2.02	510
33	0.4593	0.2961	0.7916	0.1450	0.0956	101.6	1309	71.4	4.49	0.0020	1378	0	1221	54.6	0.64	0.99	481	7	1519	63.4	14.27	0.0000	921	1443	47.7	1.31	0.16	688
33	0.4593	0.2963	0.7444	0.1463	0.0970	100.4	1304	74.7	6.30	0.0080	1411	0	1221	71.9	0.77	0.92	491	5	1520	42.8	43.23	0.0000	933	1449	52.8	1.89	0.07	722
33	0.4470	0.2999	0.7908	0.1480	0.0945	99.5	1252	60.5	1.21	0.0060	1227	42	1161	47.1	0.35	1.16	414	53	1416	46.3	1.60	0.0006	724	1326	34.4	0.50	1.39	508
33	0.4462	0.2995	0.7648	0.1462	0.0940	100.2	1255	63.6	1.21	0.0060	1224	26	1164	53.5	0.36	1.41	390	56	1425	45.7	1.52	0.0018	731	1334	34.9	0.49	1.63	509
33	0.4640	0.2975	0.8510	0.1464	0.0966	101.0	1297	82.6	4.66	0.0070	1387	0	1214	62.9	0.67	0.83	496	7	1500	56.9	20.89	0.0000	912	1428	51.4	1.48	0.11	727
33	0.4452	0.2970	0.7876	0.1478	0.0971	100.1	1286	62.9	2.59	0.0080	1298	0	1195	55.9	0.51	0.95	471	21	1480	46.3	5.90	0.0000	808	1400	46.2	0.91	0.31	657
33	0.4389	0.2987	0.8132	0.1452	0.0960	94.1	1271	81.2	1.12	0.0080	1241	22	1176	59.1	0.35	1.25	472	53	1447	61.3	1.40	0.0008	764	1353	38.5	0.49	1.43	509
33	0.4348	0.2979	0.8308	0.1451	0.0970	96.7	1279	63.3	1.30	0.0080	1313	7	1184	54.7	0.38	1.09	428	48	1461	56.8	1.80	0.0062	827	1372	40.0	0.56	1.07	563
33	0.4465	0.2987	0.8974	0.1480	0.0959	101.1	1271	61.5	1.65	0.0050	1328	0	1180	42.8	0.41	1.07	462	32	1445	47.2	2.55	0.0004	845	1362	37.6	0.66	0.59	629
33	0.4510	0.2985	0.7090	0.1465	0.0949	98.6	1276	75.1	1.83	0.0080	1188	11	1182	54.5	0.43	1.08	451	36	1456	56.5	3.19	0.0000	775	1372	44.2	0.70	0.75	539
33	0.4678	0.2989	0.8174	0.1452	0.0954	96.7	1286	85.8	2.94	0.0030	1357	0	1204	68.9	0.55	0.84	491	10	1483	88.5	7.71	0.0000	892	1407	70.3	1.05	0.19	715
33	0.4387	0.2989	0.8050	0.1452	0.0977	98.3	1271	68.1	1.65	0.0030	1181	0	1184	50.3	0.42	0.93	475	32	1457	51.9	2.64	0.0000	826	1372	44.0	0.67	0.58	558
33	0.4572	0.2998	0.8372	0.1478	0.0959	98.7	1266	71.7	2.03	0.0080	1269	0	1181	57.9	0.46	0.82	478	27	1443	62.2	3.96	0.0000	854	1364	45.7	0.78	0.38	633
33	0.4512	0.2986	0.8180	0.1465	0.0953	97.4	1275	77.8	1.62	0.0100	1303	10	1184	57.7	0.41	0.99	463	36	1457	56.1	2.68	0.0005	813	1372	46.5	0.66	0.64	602
33	0.4606	0.2985	0.7696	0.1462	0.0977	97.6	1290	88.9	4.34	0.0040	1262	0	1208	74.9	0.68	0.78	536	9	1492	85.1	19.95	0.0000	870	1421	67.2	1.52	0.11	704
33	0.4610	0.2993	0.7340	0.1462	0.0948	98.8	1272	93.1	2.24	0.0040	1245	0	1188	53.4	0.48	0.99	451	21	1459	59.1	4.95	0.0003	823	1379	52.3	0.84	0.44	598
33	0.4571	0.2968	0.8542	0.1452	0.0986	100.8	1306	83.7	6.68	0.0020	1388	0	1226	58.1	0.78	0.94	509	6	1523	56.1	50.49	0.0000	1024	1453	56.6	1.93	0.07	755
33	0.4336	0.2989	0.8724	0.1468	0.0988	98.1	1267	68.1	1.58	0.0120	1263	39	1181	52.5	0.42	0.95	472	37	1449	47.2	2.31	0.0000	831	1365	37.9	0.64	0.56	594
33	0.4588	0.2987	0.7222	0.1469	0.0975	95.5	1284	100.1	3.83	0.0070	1287	0	1204	69.3	0.63	0.79	474	19	1485	79.9	14.64	0.0000	891	1412	69.3	1.32	0.18	669
33	0.4590	0.2991	0.7564	0.1474	0.0970	100.2	1274	84.5	3.65	0.0020	1318	0	1195	59.5	0.61	0.74	515	9	1469	59.7	13.96	0.0000	865	1395	54.5	1.27	0.19	652
33	0.4498	0.2982	0.8240	0.1470	0.0989	94.3	1284	100.8	2.95	0.0070	1265	0	1203	67.9	0.57	0.87	462	12	1486	70.5	7.27	0.0000	876	1411	71.1	1.09	0.23	697
33	0.4681	0.2999	0.7578	0.1480	0.0956	99.3	1268	80.8	3.11	0.0070	1242	12	1190	63.0	0.58	0.82	492	14	1456	60.6	10.99	0.0000	855	1382	57.0	1.19	0.24	655
33	0.4369	0.2974	0.7420	0.1470	0.0973	100.5	1279	59.1	1.95	0.0040	1276	0	1186	53.0	0.45	0.98	438	36	1468	44.3	3.60	0.0000	795	1383	37.1	0.73	0.60	570
33	0.4440	0.2989	0.8770	0.1453	0.0981	94.8	1280	74.0	1.75	0.0030	1286	0	1193	60.7	0.44	0.97	461	27	1468	69.1	2.95	0.0000	899	1386	59.3	0.73	0.52	632
33	0.4622	0.2999	0.7494	0.1452	0.0977	95.4	1280	85.7	3.57	0.0070	1345	10	1203	82.2	0.62	0.71	481	10	1479	80.2	14.05	0.0000	900	1406	73.9	1.30	0.16	693
33	0.4581	0.2996	0.8600	0.1456	0.0942	94.6	1273	80.0	1.34	0.0080	1263	0	1181	68.0	0.38	1.11	450	44	1445	69.9	1.87	0.0009	812	1359	55.8	0.58	0.97	602
33	0.4457	0.2976	0.8726	0.1460	0.0979	98.8	1287	70.4	2.52	0.0070	1332	0	1201	56.3	0.50	0.88	500	17	1486	60.3	5.35	0.0000	942	1407	48.8	0.92	0.25	703
33	0.4601	0.2972	0.7864	0.1459	0.0956	102.0	1295	72.5	3.89	0.0030	1366	0	1208	57.3	0.60	0.85	491	11	1496	51.6	12.76	0.0000	857	1420	42.8	1.20	0.16	677
33	0.4411	0.2964	0.8654	0.1463	0.0948	97.3	1289	67.6	1.25	0.0050	1438	30	1193	56.4	0.38	1.26	449	46	1472	53.4	1.70	0.0003	837	1382	40.1	0.54	1.07	585
33	0.4327	0.2962	0.7100	0.1464	0.0950	97.4	1282	61.0	1.22	0.0100	1210	53	1180	47.4	0.35	1.49	418	59	1464	48.4	1.51	0.0008	705	1368	34.0	0.48	1.86	435
33	0.4451	0.2969	0.8590	0.1476	0.0975	98.0	1291	67.9	2.43	0.0070	1362	0	1199	58.3	0.50	0.94	487	20	1485	56.4	4.69	0.0007	893	1404	50.6	0.88	0.27	661
33	0.4670	0.2964	0.7506	0.1463	0.0988	101.0	1316	68.0	19.48	0.0150	1394	0	1236	73.1	1.27	0.84	506	1	1538	16.7	3091.95	0.0000	1094	1476	75.6	4.99	0.02	780

Continuation of Table S1.

c_2	q_H	σ_{OO}	ϵ_{OO}	d_{OO}	d_{OH}	a_{HOO}	ρ	ϵ	μ	P_v	λ	wt$\%_v$	ρ	ϵ	μ	P_v	λ	wt$\%_v$	ρ	ϵ	μ	P_v	λ	ρ	ϵ	μ	P_v	λ
34.5	0.4300	0.2965	0.7526	0.1460	0.0944	96.6	1274	61.2	0.92	0.0090	635	59	1169	47.9	0.31	2.07	392	70	1448	40.9	0.99	0.0077	710	1345	32.1	0.39	2.78	469
34.5	0.4303	0.2971	0.8536	0.1461	0.0977	94.4	1282	67.7	1.26	0.0040	634	0	1186	56.6	0.37	1.13	451	44	1466	50.2	1.66	0.0007	808	1374	41.6	0.53	1.02	546
34.5	0.4307	0.2967	0.8336	0.1472	0.0986	98.8	1281	69.6	1.71	0.0030	619	0	1191	50.6	0.44	0.98	459	32	1475	48.5	2.92	0.0000	859	1391	33.3	0.69	0.54	625
34.5	0.4307	0.2969	0.8568	0.1458	0.0956	99.8	1280	58.2	1.10	0.0070	694	51	1181	42.6	0.34	1.45	402	54	1457	32.8	1.33	0.0015	777	1363	30.9	0.48	1.38	546
34.5	0.4311	0.2993	0.8170	0.1468	0.0944	95.7	1248	56.2	0.75	0.0130	594	68	1147	46.5	0.28	2.39	399	77	1398	42.8	0.73	0.0060	719	1294	27.0	0.34	3.91	463
34.5	0.4334	0.2963	0.8700	0.1456	0.0971	98.0	1294	58.5	1.46	0.0050	701	14	1197	51.8	0.41	1.07	471	38	1484	45.5	2.15	0.0000	860	1395	34.6	0.60	0.81	627
34.5	0.4343	0.2976	0.7496	0.1475	0.0983	99.7	1273	66.5	2.01	0.0040	630	0	1184	46.7	0.45	0.97	463	32	1464	45.2	3.98	0.0000	806	1381	34.9	0.76	0.54	578
34.5	0.4343	0.2994	0.8354	0.1455	0.0948	100.3	1253	55.3	0.92	0.0170	618	53	1159	45.0	0.32	1.69	405	66	1416	34.7	1.03	0.0023	764	1319	28.4	0.41	2.19	485
34.5	0.4346	0.2966	0.8618	0.1459	0.0968	96.0	1290	69.9	1.34	0.0070	689	0	1192	50.7	0.39	1.16	453	41	1476	55.5	1.87	0.0000	833	1386	40.7	0.57	0.93	599
34.5	0.4368	0.2997	0.8778	0.1456	0.0982	99.4	1263	59.8	1.55	0.0050	627	17	1179	48.9	0.41	0.86	436	37	1443	48.2	2.31	0.0003	830	1358	36.1	0.63	0.65	607
34.5	0.4378	0.2994	0.7466	0.1461	0.0965	100.5	1260	55.7	1.41	0.0040	611	55	1169	44.4	0.38	1.07	420	45	1435	40.5	2.15	0.0000	733	1346	35.3	0.57	0.94	525
34.5	0.4384	0.2998	0.7850	0.1458	0.0952	94.1	1255	65.5	0.94	0.0090	610	73	1159	49.8	0.31	1.56	402	67	1416	51.1	1.01	0.0029	722	1318	36.5	0.40	2.55	466
34.5	0.4385	0.2986	0.7744	0.1470	0.0969	95.1	1267	70.6	1.36	0.0080	615	8	1175	48.8	0.38	1.16	455	48	1445	61.8	1.91	0.0038	768	1356	39.5	0.55	1.03	546
34.5	0.4398	0.2970	0.7946	0.1475	0.0976	101.1	1286	62.2	2.30	0.0050	638	0	1193	48.2	0.49	0.99	459	27	1477	46.0	4.69	0.0000	836	1395	38.4	0.85	0.39	656
34.5	0.4404	0.2984	0.8360	0.1478	0.0950	95.1	1266	68.9	1.04	0.0080	628	58	1167	50.8	0.33	1.47	408	57	1431	49.9	1.23	0.0018	776	1337	40.7	0.45	1.79	516
34.5	0.4413	0.2960	0.8944	0.1456	0.0943	99.7	1294	58.8	1.27	0.0080	744	11	1202	47.6	0.38	1.47	441	45	1478	43.4	1.68	0.0013	852	1388	36.4	0.55	1.03	614
34.5	0.4417	0.2992	0.7808	0.1467	0.0970	101.2	1263	61.6	1.79	0.0050	614	0	1177	46.1	0.43	0.98	440	30	1444	45.2	3.09	0.0004	828	1360	35.6	0.69	0.68	563
34.5	0.4448	0.2982	0.8222	0.1454	0.0988	101.2	1283	66.6	3.13	0.0030	637	0	1200	52.3	0.55	0.85	503	13	1484	45.6	9.11	0.0000	906	1407	44.4	1.10	0.19	739
34.5	0.4460	0.2989	0.8250	0.1480	0.0970	98.4	1269	64.6	1.84	0.0110	629	77	1181	50.8	0.44	0.90	454	31	1448	51.4	3.14	0.0000	833	1366	41.6	0.72	0.51	583
34.5	0.4470	0.2994	0.8732	0.1459	0.0966	97.0	1271	76.6	1.56	0.0030	625	17	1183	57.7	0.41	1.08	456	37	1449	56.8	2.36	0.0000	824	1365	37.2	0.64	0.53	588
34.5	0.4476	0.2993	0.8062	0.1473	0.0950	98.3	1261	64.1	1.32	0.0040	614	12	1171	49.9	0.38	1.16	444	45	1432	49.6	1.85	0.0006	769	1344	38.1	0.55	1.11	520
34.5	0.4482	0.2972	0.7634	0.1467	0.0966	95.5	1288	78.8	2.18	0.0020	658	0	1198	67.4	0.48	0.95	433	24	1484	73.3	4.23	0.0010	832	1402	53.0	0.83	0.53	585
34.5	0.4482	0.2976	0.8086	0.1457	0.0960	97.4	1283	76.2	1.79	0.0040	631	22	1194	51.2	0.44	0.97	431	26	1476	56.1	3.16	0.0009	854	1392	43.7	0.72	0.61	637
34.5	0.4488	0.2996	0.7644	0.1480	0.0955	98.0	1257	65.4	1.52	0.0020	626	60	1170	55.1	0.39	1.15	444	44	1431	57.4	2.31	0.0004	785	1345	41.6	0.60	0.87	533
34.5	0.4490	0.2969	0.8806	0.1460	0.0946	101.5	1291	61.7	1.68	0.0030	698	0	1197	49.8	0.43	1.09	462	29	1475	41.2	2.66	0.0000	847	1391	34.7	0.67	0.58	647
34.5	0.4498	0.2994	0.7472	0.1463	0.0966	95.2	1268	65.3	1.74	0.0150	608	0	1183	61.3	0.44	0.93	467	38	1454	74.4	3.31	0.0011	806	1370	51.4	0.71	0.71	586
34.5	0.4504	0.2980	0.7794	0.1467	0.0942	97.9	1277	72.5	1.45	0.0060	636	37	1180	53.7	0.39	1.17	433	44	1454	55.8	2.07	0.0027	785	1366	39.6	0.58	0.94	540
34.5	0.4510	0.2994	0.7080	0.1474	0.0952	96.5	1261	70.0	1.60	0.0040	592	0	1174	55.8	0.41	1.04	449	41	1439	62.9	2.52	0.0000	733	1353	44.3	0.63	0.97	544
34.5	0.4542	0.2974	0.7782	0.1465	0.0984	98.7	1293	69.6	4.88	0.0050	665	0	1211	58.6	0.68	0.77	473	7	1504	55.8	23.71	0.0000	894	1430	57.2	1.53	0.09	711
34.5	0.4550	0.2997	0.8380	0.1452	0.0947	95.6	1270	78.5	1.34	0.0150	635	12	1180	58.2	0.38	1.04	457	42	1444	68.2	1.94	0.0000	801	1358	43.5	0.58	0.86	575
34.5	0.4555	0.2977	0.8910	0.1467	0.0988	98.1	1292	84.1	4.48	0.0050	665	0	1213	66.0	0.67	0.78	500	10	1499	78.2	17.46	0.0000	948	1427	54.6	1.46	0.11	720
34.5	0.4559	0.2992	0.7444	0.1473	0.0989	98.0	1277	72.6	4.23	0.0160	657	0	1200	63.6	0.70	0.79	473	7	1478	95.5	23.65	0.0000	909	1406	59.4	1.57	0.12	681
34.5	0.4565	0.2966	0.8090	0.1452	0.0975	98.8	1307	89.4	4.63	0.0040	701	0	1222	66.8	0.68	0.92	480	9	1519	59.5	18.23	0.0000	926	1445	54.8	1.46	0.11	763
34.5	0.4566	0.2962	0.8454	0.1461	0.0955	100.6	1300	77.8	3.19	0.0050	732	0	1213	57.7	0.55	0.93	483	15	1504	44.8	7.17	0.0000	926	1426	48.5	1.02	0.27	682
34.5	0.4570	0.2970	0.8592	0.1469	0.0968	101.4	1295	89.1	3.66	0.0080	688	0	1210	54.8	0.61	0.86	474	12	1497	63.5	12.59	0.0000	984	1422	48.5	1.27	0.16	729
34.5	0.4572	0.2970	0.8734	0.1453	0.0964	95.4	1302	83.6	2.72	0.0050	714	13	1215	67.1	0.54	0.86	466	18	1505	89.1	5.62	0.0000	941	1427	65.5	1.01	0.23	724
34.5	0.4584	0.2992	0.7090	0.1460	0.0943	95.0	1272	75.4	1.69	0.0090	623	45	1183	68.8	0.42	1.17	423	42	1455	73.4	2.77	0.0011	758	1369	59.3	0.67	0.88	558
34.5	0.4591	0.2975	0.7154	0.1465	0.0963	100.6	1290	68.3	4.19	0.0020	640	0	1207	61.2	0.65	0.93	468	10	1495	62.1	19.22	0.0000	854	1420	46.0	1.40	0.18	683
34.5	0.4612	0.2992	0.8140	0.1456	0.0989	94.4	1287	92.8	4.17	0.0150	688	0	1212	75.6	0.69	0.70	514	9	1494	90.6	20.51	0.0000	946	1422	76.6	1.50	0.08	726
34.5	0.4613	0.2986	0.8690	0.1464	0.0964	95.7	1282	90.5	2.39	0.0030	637	0	1201	69.0	0.53	0.83	467	18	1478	68.9	5.66	0.0000	943	1401	59.3	0.97	0.23	675
34.5	0.4616	0.2982	0.7362	0.1459	0.0967	101.2	1290	77.8	5.00	0.0090	664	0	1207	56.4	0.68	0.73	492	5	1492	51.8	29.70	0.0000	861	1419	52.7	1.57	0.11	657
34.5	0.4620	0.2996	0.8424	0.1475	0.0972	95.8	1275	95.0	2.90	0.0090	617	0	1194	62.2	0.55	0.82	482	15	1465	78.4	7.84	0.0004	868	1390	68.0	1.07	0.26	696
34.5	0.4621	0.2971	0.7212	0.1465	0.0969	100.7	1300	59.3	7.06	0.0080	710	0	1216	57.1	0.79	0.80	493	4	1509	55.4	45.19	0.0000	921	1438	50.8	1.94	0.07	687
34.5	0.4622	0.2997	0.8564	0.1471	0.0950	98.2	1270	81.1	2.00	0.0030	639	30	1185	60.1	0.46	0.96	466	21	1448	63.6	3.54	0.0005	846	1368	45.4	0.77	0.43	608
34.5	0.4636	0.2986	0.8136	0.1478	0.0946	100.6	1276	77.7	2.46	0.0090	659	0	1189	56.3	0.49	1.03	473	20	1461	46.5	5.61	0.0000	883	1383	43.2	0.91	0.28	644
34.5	0.4638	0.2989	0.8264	0.1475	0.0984	98.8	1284	78.7	5.14	0.0040	671	0	1207	69.1	0.74	0.79	498	4	1487	77.0	44.72	0.0000	941	1416	58.9	1.81	0.14	766
34.5	0.4639	0.2990	0.7818	0.1453	0.0985	101.2	1290	84.0	7.21	0.0040	661	0	1212	58.4	0.84	0.73	535	3	1497	33.2	70.59	0.0000	998	1428	59.8	2.22	0.07	787
34.5	0.4642	0.2968	0.8690	0.1455	0.0977	95.8	1309	103.0	5.42	0.0040	697	0	1228	79.6	0.71	0.85	500	6	1525	111.5	22.72	0.0000	1001	1454	82.4	1.65	0.09	772
34.5	0.4643	0.2980	0.8334	0.1468	0.0969	97.5	1289	94.9	4.28	0.0080	680	0	1210	65.1	0.65	0.85	494	14	1494	83.4	15.85	0.0000	967	1421	60.7	1.37	0.12	744
34.5	0.4648	0.2966	0.8546	0.1453	0.0951	96.8	1305	81.4	3.10	0.0030	718	0	1222	62.7	0.57	1.00	488	13	1512	78.6	7.54	0.0000	956	1434	61.7	1.06	0.25	705

Continuation of Table S1.

c_2	q_H	σ_{OO}	ϵ_{OO}	d_{OO}	d_{OH}	a_{HOO}	ρ	ϵ	μ	P_v	λ	wt%$_v$	ρ	ϵ	μ	P_v	λ	wt%$_v$	ρ	ϵ	μ	P_v	λ	ρ	ϵ	μ	P_v	λ
34.5	0.4649	0.2972	0.8148	0.1470	0.0988	101.5	1302	76.7	11.49	0.0040	686	0	1224	65.0	1.02	0.76	504	1	1520	53.0	173.21	0.0000	970	1452	63.3	3.05	0.02	825
34.5	0.4656	0.2974	0.8660	0.1458	0.0941	95.6	1297	85.2	1.99	0.0040	706	0	1209	65.9	0.49	1.02	473	19	1490	62.9	3.91	0.0000	882	1409	54.5	0.83	0.40	629
34.5	0.4664	0.2970	0.7622	0.1453	0.0968	94.9	1308	98.7	5.38	0.0080	636	0	1225	77.9	0.73	0.84	491	6	1524	77.6	23.55	0.0000	870	1452	70.5	1.65	0.11	738
34.5	0.4670	0.2963	0.8422	0.1472	0.0957	99.6	1308	82.3	5.19	0.0060	726	0	1224	62.3	0.71	0.89	507	8	1513	60.7	18.53	0.0000	897	1440	59.5	1.48	0.35	725
34.5	0.4679	0.2967	0.8590	0.1470	0.0958	101.5	1303	82.3	5.39	0.0020	751	0	1220	61.6	0.72	0.98	504	5	1508	60.9	24.13	0.0000	977	1437	49.4	1.58	0.08	714
34.5	0.4418	0.2962	0.8486	0.1468	0.0978	98.3	1298	80.7	2.46	0.0070	1346	0	1206	54.4	0.50	0.97	483	19	1497	59.5	5.03	0.0009	881	1417	45.7	0.88	0.30	648
34.5	0.4571	0.2965	0.7380	0.1453	0.0955	95.6	1301	78.4	2.83	0.0040	1326	0	1212	67.3	0.53	0.97	450	23	1508	76.4	6.52	0.0000	858	1429	55.8	1.01	0.33	642
34.5	0.4641	0.2975	0.7778	0.1477	0.0950	99.2	1288	71.6	3.36	0.0020	1352	0	1202	55.6	0.57	0.95	476	14	1485	70.3	9.58	0.0000	813	1408	50.8	1.10	0.29	655
34.5	0.4314	0.2969	0.8712	0.1470	0.0987	101.0	1283	59.1	1.86	0.0130	1288	0	1192	48.6	0.45	0.98	459	28	1475	38.7	3.22	0.0000	856	1392	33.7	0.74	0.42	632
34.5	0.4649	0.2964	0.7160	0.1473	0.0958	99.0	1301	80.3	5.69	0.0040	1389	0	1218	63.7	0.74	0.89	459	10	1513	41.1	31.63	0.0000	900	1441	56.0	1.75	0.12	674
34.5	0.4384	0.2993	0.7534	0.1476	0.0983	96.9	1259	70.8	1.72	0.0030	1236	0	1177	53.5	0.43	0.92	489	39	1443	57.4	2.99	0.0019	766	1360	40.7	0.70	0.63	537
34.5	0.4344	0.2991	0.7628	0.1470	0.0974	95.1	1256	77.6	1.25	0.0040	1186	26	1169	54.5	0.36	1.39	431	52	1435	44.7	1.64	0.0000	763	1343	36.1	0.52	1.32	513
34.5	0.4527	0.2965	0.8478	0.1455	0.0948	94.1	1298	80.1	1.63	0.0010	1427	0	1205	62.3	0.42	1.08	486	37	1492	68.6	2.50	0.0004	847	1406	53.9	0.67	0.73	615
34.5	0.4306	0.2983	0.7632	0.1461	0.0954	98.5	1262	58.4	1.01	0.0080	1272	80	1162	42.5	0.32	1.49	423	65	1430	39.1	1.12	0.0028	700	1331	29.3	0.42	2.41	479
34.5	0.4397	0.2971	0.8242	0.1462	0.0940	95.3	1277	63.6	0.99	0.0070	1287	49	1177	53.8	0.33	1.54	451	60	1450	48.1	1.11	0.0023	770	1353	39.9	0.44	2.09	498
34.5	0.4700	0.2981	0.8708	0.1469	0.0976	99.7	1295	68.8	6.83	0.0070	1401	0	1217	68.8	0.81	0.81	501	3	1502	48.8	58.83	0.0000	920	1434	60.9	2.14	0.16	781
34.5	0.4645	0.2971	0.8906	0.1457	0.0987	101.2	1306	70.4	8.67	0.0060	1339	0	1228	65.6	0.89	0.76	524	2	1522	68.6	86.01	0.0000	965	1457	57.2	2.58	0.03	813
34.5	0.4665	0.2983	0.8826	0.1474	0.0968	96.9	1288	83.2	4.04	0.0050	1310	0	1207	69.1	0.63	0.88	507	8	1487	65.8	11.69	0.0000	951	1415	66.0	1.30	0.12	744
34.5	0.4561	0.2967	0.7552	0.1466	0.0990	97.2	1304	85.7	6.96	0.0070	1379	0	1223	72.2	0.83	0.87	484	2	1522	83.6	58.83	0.0000	902	1452	67.7	2.05	0.06	728
34.5	0.4687	0.2990	0.8764	0.1461	0.0973	101.8	1287	64.8	5.29	0.0060	1348	0	1209	64.1	0.73	0.76	510	4	1488	55.1	34.41	0.0000	1004	1418	53.9	1.75	0.08	792
34.5	0.4605	0.2974	0.8310	0.1474	0.0971	96.0	1294	108.9	3.76	0.0050	1319	0	1210	61.9	0.62	0.83	472	11	1497	74.6	11.95	0.0000	912	1422	64.4	1.23	0.12	696
34.5	0.4395	0.2987	0.7298	0.1469	0.0981	94.5	1269	73.2	1.82	0.0060	1232	20	1184	58.0	0.44	0.96	472	37	1459	62.0	3.25	0.0000	813	1374	50.2	0.70	0.70	567
34.5	0.4506	0.2970	0.7710	0.1459	0.0972	97.8	1297	85.7	3.22	0.0090	1297	0	1208	64.2	0.56	0.91	488	15	1500	57.4	8.52	0.0000	872	1422	48.9	1.07	0.30	658
34.5	0.4614	0.2960	0.8792	0.1457	0.0984	95.5	1317	102.8	5.98	0.0080	1439	0	1234	75.5	0.81	0.91	512	3	1538	99.1	35.93	0.0000	1017	1468	73.7	1.85	0.06	767
34.5	0.4499	0.2964	0.8486	0.1459	0.0943	97.9	1292	64.9	1.56	0.0040	1429	27	1202	59.5	0.42	1.06	449	33	1484	52.4	2.28	0.0005	848	1397	46.1	0.64	0.77	636
34.5	0.4575	0.2998	0.8138	0.1469	0.0968	94.2	1269	85.0	2.06	0.0050	1246	0	1189	60.8	0.48	0.90	478	24	1457	86.4	4.31	0.0000	847	1377	64.3	0.82	0.35	690
34.5	0.4320	0.2989	0.7320	0.1470	0.0973	100.0	1256	55.4	1.46	0.0080	1211	0	1168	46.4	0.38	1.07	438	46	1436	41.9	2.09	0.0020	746	1346	32.6	0.55	1.07	504
34.5	0.4480	0.2970	0.8968	0.1466	0.0968	100.8	1292	71.4	2.37	0.0050	1380	0	1203	53.6	0.51	0.96	449	17	1487	48.8	4.93	0.0000	929	1407	36.4	0.88	0.30	696
34.5	0.4467	0.2963	0.8156	0.1462	0.0958	100.0	1297	62.4	2.19	0.0070	1383	0	1203	51.2	0.47	1.03	443	24	1490	54.1	3.92	0.0000	853	1408	38.6	0.80	0.48	643
34.5	0.4592	0.2998	0.7426	0.1470	0.0981	97.6	1274	77.3	4.03	0.0030	1271	0	1197	65.7	0.65	0.75	478	7	1470	55.1	17.91	0.0000	854	1398	64.1	1.40	0.17	690
34.5	0.4664	0.2985	0.8874	0.1464	0.0984	94.2	1297	88.2	4.60	0.0030	1300	0	1217	81.4	0.70	0.74	504	8	1501	83.5	21.18	0.0000	1015	1432	74.0	1.64	0.11	794
34.5	0.4309	0.2988	0.8384	0.1454	0.0948	96.2	1261	59.9	0.82	0.0150	1242	79	1157	44.5	0.29	1.92	378	71	1418	39.6	0.82	0.0033	716	1316	30.9	0.37	3.05	442
34.5	0.4440	0.2969	0.7322	0.1471	0.0989	99.3	1290	67.8	4.10	0.0020	1310	0	1206	53.5	0.63	0.87	492	10	1498	60.4	15.69	0.0000	858	1423	50.3	1.31	0.17	653
34.5	0.4415	0.2997	0.8478	0.1467	0.0942	94.9	1254	67.8	0.89	0.0160	1187	69	1157	53.1	0.30	1.72	433	65	1411	44.5	0.91	0.0067	740	1314	36.3	0.40	2.56	459
34.5	0.4397	0.2979	0.7400	0.1472	0.0966	96.1	1274	75.3	1.52	0.0080	1298	63	1181	51.3	0.40	1.05	424	38	1457	58.0	2.33	0.0012	807	1369	42.3	0.62	1.01	547
34.5	0.4594	0.2964	0.7460	0.1462	0.0988	100.7	1310	89.1	11.04	0.0020	1446	0	1229	56.4	1.01	0.75	509	3	1532	36.2	255.80	0.0000	942	1463	55.5	3.01	0.05	747
34.5	0.4645	0.2996	0.8802	0.1458	0.0980	96.0	1283	87.1	3.68	0.0030	1332	0	1206	68.9	0.63	0.79	524	8	1481	63.3	12.86	0.0000	936	1409	66.8	1.31	0.12	747
34.5	0.4662	0.2997	0.8114	0.1450	0.0944	97.3	1278	83.4	2.04	0.0080	1279	0	1191	59.3	0.46	0.95	461	21	1461	66.5	3.97	0.0000	861	1381	55.4	0.79	0.37	651
34.5	0.4687	0.2965	0.8836	0.1480	0.0975	100.1	1307	84.2	8.45	0.0100	1478	0	1226	65.2	0.87	0.96	478	4	1520	36.9	94.34	0.0000	981	1451	64.6	2.35	0.03	794
34.5	0.4304	0.2988	0.8570	0.1464	0.0961	98.4	1261	58.1	0.99	0.0040	1248	34	1164	47.1	0.32	1.44	448	58	1425	39.8	1.10	0.0011	769	1331	28.6	0.43	1.86	521
34.5	0.4496	0.2996	0.7756	0.1468	0.0946	101.2	1258	53.6	1.41	0.0030	1244	63	1169	44.7	0.39	1.11	452	42	1430	40.4	2.17	0.0019	789	1344	32.8	0.59	0.97	493
34.5	0.4631	0.2990	0.7048	0.1468	0.0966	99.8	1279	76.5	4.25	0.0020	1255	0	1200	52.4	0.67	0.81	522	7	1479	52.9	25.05	0.0000	842	1405	57.1	1.50	0.16	656
34.5	0.4331	0.2997	0.8218	0.1455	0.0963	100.5	1255	63.6	1.08	0.0060	1198	59	1163	46.0	0.34	1.40	433	54	1422	36.6	1.31	0.0051	782	1328	31.3	0.46	1.43	523
34.5	0.4584	0.2999	0.7640	0.1478	0.0979	98.0	1267	76.3	3.37	0.0050	1318	0	1191	59.3	0.60	0.83	481	7	1459	55.9	12.09	0.0000	842	1386	56.3	1.23	0.19	667
34.5	0.4389	0.2962	0.7054	0.1452	0.0988	97.6	1299	76.8	3.59	0.0060	1401	0	1212	52.4	0.59	0.92	431	16	1512	53.8	9.93	0.0000	865	1434	46.8	1.14	0.31	668
34.5	0.4626	0.3000	0.8082	0.1479	0.0958	99.8	1264	69.8	2.41	0.0050	1279	0	1184	52.2	0.51	0.92	445	18	1447	57.7	6.47	0.0006	841	1370	48.2	0.95	0.27	631
34.5	0.4343	0.2968	0.7284	0.1469	0.0988	101.4	1284	58.3	2.68	0.0080	1274	0	1196	49.3	0.53	0.95	450	26	1486	43.9	7.23	0.0000	805	1405	36.5	0.96	0.33	603
34.5	0.4671	0.2972	0.8680	0.1476	0.0971	99.3	1299	60.2	6.03	0.0030	1392	0	1218	66.6	0.74	0.81	534	7	1507	76.7	37.15	0.0000	984	1437	57.1	1.75	0.06	763
34.5	0.4564	0.2982	0.8820	0.1462	0.0952	98.8	1285	71.5	1.99	0.0060	1342	0	1194	53.4	0.46	1.00	399	23	1470	60.1	3.54	0.0000	851	1389	46.2	0.77	0.38	652
34.5	0.4500	0.2983	0.8984	0.1458	0.0980	94.9	1288	84.9	2.26	0.0040	1348	0	1202	63.7	0.49	0.84	504	27	1482	74.5	4.48	0.0000	921	1403	57.9	0.87	0.39	687

Continuation of Table S1.

c_2	q_H	σ_{OO}	ϵ_{OO}	d_{OO}	d_{OH}	a_{HOO}	ρ	ϵ	μ	P_v	λ	wt%$_v$	ρ	ϵ	μ	P_v	λ	wt%$_v$	ρ	ϵ	μ	P_v	λ	ρ	ϵ	μ	P_v	λ
34.5	0.4355	0.2997	0.8506	0.1462	0.0963	99.7	1259	55.2	1.11	0.0040	1247	42	1165	45.5	0.34	1.27	415	56	1423	40.8	1.40	0.0020	771	1331	29.8	0.48	1.37	566
34.5	0.4305	0.2987	0.7934	0.1462	0.0973	96.6	1262	60.4	1.14	0.0090	1292	30	1170	50.3	0.35	1.27	435	57	1437	44.1	1.44	0.0012	789	1344	34.0	0.49	1.46	495
34.5	0.4661	0.2977	0.7450	0.1456	0.0965	101.9	1298	60.7	6.23	0.0050	1289	0	1215	58.8	0.78	0.82	507	8	1506	50.2	47.86	0.0000	892	1434	58.1	1.90	0.05	722
34.5	0.4483	0.2974	0.8786	0.1464	0.0954	99.9	1287	65.3	1.77	0.0050	1436	0	1194	54.4	0.43	1.10	463	32	1472	49.2	2.77	0.0000	872	1387	37.8	0.69	0.55	639
34.5	0.4602	0.2967	0.7138	0.1477	0.0967	100.5	1298	67.6	6.41	0.0050	1378	0	1214	52.9	0.75	0.92	486	6	1507	53.8	39.19	0.0000	894	1434	47.9	1.76	0.10	693
34.5	0.4492	0.2987	0.8732	0.1462	0.0960	97.2	1277	76.8	1.60	0.0060	1320	38	1187	59.8	0.42	1.04	488	32	1458	51.9	2.61	0.0010	874	1373	44.6	0.65	0.58	579
34.5	0.4509	0.2971	0.8082	0.1477	0.0964	96.8	1289	67.2	2.24	0.0060	1272	0	1199	63.3	0.50	0.96	460	23	1482	59.1	4.46	0.0003	840	1402	54.0	0.87	0.41	592
34.5	0.4539	0.2969	0.7818	0.1472	0.0984	96.2	1297	88.0	4.24	0.0040	1275	0	1214	67.8	0.67	0.78	495	9	1508	73.4	19.41	0.0000	896	1434	58.5	1.42	0.12	710
34.5	0.4475	0.2978	0.7804	0.1453	0.0966	99.2	1285	75.7	2.20	0.0050	1206	12	1196	55.0	0.47	0.95	473	24	1479	53.5	4.54	0.0000	834	1398	45.8	0.82	0.41	655
34.5	0.4571	0.2968	0.7714	0.1463	0.0958	97.2	1295	66.9	2.95	0.0050	1284	0	1209	60.2	0.55	0.96	473	17	1500	56.3	7.46	0.0000	869	1420	53.4	1.02	0.30	659
34.5	0.4547	0.2987	0.8156	0.1456	0.0948	96.9	1277	83.6	1.62	0.0090	1293	61	1186	55.4	0.42	0.97	446	34	1459	51.6	2.46	0.0000	863	1374	45.3	0.66	0.67	626
34.5	0.4556	0.2987	0.8518	0.1458	0.0960	98.3	1283	75.9	2.15	0.0030	1251	0	1194	57.3	0.47	0.89	472	23	1469	63.8	4.08	0.0000	876	1390	44.4	0.82	0.33	663
34.5	0.4618	0.2979	0.8928	0.1468	0.0968	95.8	1292	84.5	3.03	0.0140	1361	8	1209	69.9	0.58	0.91	490	9	1491	75.3	7.70	0.0000	931	1416	65.0	1.12	0.15	725
34.5	0.4410	0.2970	0.8794	0.1478	0.0989	100.2	1291	72.8	2.87	0.0050	1342	0	1200	48.9	0.54	0.85	484	15	1486	49.7	6.69	0.0000	923	1409	40.1	1.00	0.22	695
34.5	0.4352	0.2975	0.7494	0.1478	0.0940	100.3	1265	58.1	1.00	0.0100	1243	78	1161	42.9	0.32	1.73	440	65	1430	37.6	1.19	0.0043	685	1332	25.2	0.43	2.22	471
34.5	0.4568	0.2990	0.8422	0.1471	0.0984	100.1	1276	85.7	3.88	0.0040	1246	0	1200	56.0	0.63	0.79	492	10	1475	53.9	16.45	0.0000	935	1403	46.7	1.34	0.16	731
34.5	0.4492	0.2981	0.8484	0.1472	0.0984	94.9	1285	74.8	2.51	0.0030	1275	0	1200	66.4	0.52	0.81	507	21	1481	66.1	5.72	0.0000	927	1403	54.5	0.96	0.32	645
34.5	0.4613	0.2997	0.7394	0.1472	0.0966	98.6	1269	83.2	3.01	0.0050	1272	0	1191	58.0	0.58	0.71	478	15	1462	71.2	10.69	0.0000	881	1387	56.1	1.16	0.25	632
34.5	0.4332	0.2964	0.7854	0.1461	0.0952	101.3	1283	61.0	1.24	0.0050	1247	60	1182	47.0	0.36	1.45	440	53	1464	35.0	1.61	0.0011	757	1371	31.2	0.52	1.23	520
34.5	0.4372	0.2967	0.7146	0.1469	0.0945	101.7	1277	57.4	1.33	0.0060	1249	21	1177	48.5	0.37	1.31	400	54	1457	36.6	1.86	0.0016	736	1364	28.3	0.54	1.40	504
34.5	0.4581	0.2964	0.7140	0.1467	0.0942	98.2	1298	69.4	2.56	0.0050	1344	14	1204	54.2	0.51	0.98	454	26	1494	53.7	5.72	0.0009	803	1413	47.4	0.90	0.49	624
34.5	0.4527	0.2975	0.8480	0.1467	0.0981	99.4	1292	73.2	3.69	0.0080	1293	67	1207	64.9	0.62	0.82	506	9	1494	63.3	11.82	0.0005	942	1419	52.3	1.23	0.15	727
34.5	0.4468	0.2960	0.7550	0.1475	0.0958	98.6	1293	59.8	2.25	0.0030	1328	0	1200	54.1	0.48	1.08	474	28	1491	51.1	4.45	0.0000	862	1408	42.8	0.81	0.45	607
34.5	0.4505	0.2977	0.8424	0.1474	0.0971	94.4	1287	73.4	2.11	0.0180	1386	57	1197	65.7	0.48	0.96	468	27	1478	62.2	4.01	0.0003	872	1397	56.6	0.82	0.42	645
34.5	0.4690	0.2977	0.7730	0.1475	0.0959	95.5	1293	81.7	4.21	0.0030	1320	0	1210	67.6	0.65	0.83	485	9	1497	103.0	16.15	0.0000	899	1423	81.8	1.40	0.15	702
36	0.4312	0.2981	0.7216	0.1464	0.0951	100.1	1261	52.5	1.05	0.0120	588	64	1162	39.1	0.33	1.63	383	65	1431	34.6	1.25	0.0035	701	1332	25.7	0.43	2.32	458
36	0.4319	0.2966	0.7222	0.1459	0.0958	99.0	1279	58.5	1.30	0.0070	611	7	1179	48.8	0.38	1.39	394	51	1464	38.9	1.73	0.0026	752	1371	30.3	0.53	1.55	483
36	0.4320	0.2996	0.8644	0.1456	0.0960	94.2	1257	68.1	0.87	0.0080	609	77	1159	45.3	0.30	1.71	415	67	1416	42.0	0.91	0.0039	763	1315	30.0	0.38	2.41	498
36	0.4333	0.2960	0.8670	0.1466	0.0990	96.6	1297	63.5	1.97	0.0050	667	30	1204	55.0	0.47	0.82	381	28	1496	48.7	3.56	0.0000	835	1413	38.9	0.77	0.40	648
36	0.4336	0.2968	0.7830	0.1476	0.0985	96.1	1280	61.8	1.82	0.0060	625	26	1191	53.2	0.44	0.99	471	34	1476	54.2	3.15	0.0006	807	1391	41.2	0.70	0.67	644
36	0.4336	0.2998	0.8910	0.1456	0.0974	97.0	1261	58.5	1.13	0.0050	636	12	1170	50.7	0.35	1.16	452	51	1429	41.4	1.39	0.0038	830	1338	32.2	0.49	1.23	587
36	0.4342	0.2984	0.8382	0.1473	0.0970	99.1	1267	57.5	1.31	0.0040	644	58	1173	48.8	0.38	1.29	440	45	1441	37.5	1.73	0.0011	770	1352	34.3	0.54	0.97	565
36	0.4348	0.2996	0.8296	0.1462	0.0950	98.4	1254	62.0	0.89	0.0130	596	39	1155	44.2	0.30	1.58	411	67	1410	37.9	0.98	0.0044	750	1313	27.9	0.40	2.31	469
36	0.4351	0.2965	0.8010	0.1464	0.0976	101.4	1289	61.3	1.99	0.0040	661	10	1195	45.2	0.45	0.98	448	29	1482	43.4	3.54	0.0000	836	1398	32.6	0.75	0.48	616
36	0.4351	0.2984	0.7706	0.1475	0.0977	98.3	1264	63.2	1.60	0.0070	617	0	1176	45.9	0.40	1.05	468	40	1447	42.7	2.41	0.0004	782	1360	38.9	0.61	0.80	525
36	0.4353	0.2984	0.8884	0.1460	0.0976	98.5	1272	67.0	1.44	0.0040	638	0	1182	46.7	0.40	1.08	442	39	1453	46.1	2.02	0.0000	817	1366	34.4	0.59	0.78	598
36	0.4357	0.2964	0.7386	0.1478	0.0972	96.0	1282	58.3	1.73	0.0070	646	36	1188	54.4	0.43	1.08	448	41	1476	47.5	2.78	0.0019	795	1388	37.9	0.66	0.90	551
36	0.4358	0.2992	0.7898	0.1459	0.0982	100.4	1264	60.2	1.65	0.0100	644	10	1178	47.2	0.42	1.04	462	35	1448	37.2	2.91	0.0000	799	1363	34.2	0.67	0.63	558
36	0.4367	0.2966	0.7794	0.1456	0.0982	95.9	1293	71.8	2.06	0.0030	661	0	1199	57.8	0.46	1.01	459	28	1493	60.8	3.65	0.0000	815	1408	41.5	0.76	0.53	566
36	0.4367	0.2968	0.8486	0.1454	0.0970	95.8	1291	60.3	1.48	0.0070	653	16	1194	51.6	0.41	1.23	449	43	1480	48.8	2.09	0.0007	834	1392	39.7	0.61	0.84	637
36	0.4370	0.2997	0.7942	0.1468	0.0949	100.6	1250	54.4	1.02	0.0090	572	58	1155	39.6	0.32	1.47	422	63	1410	37.0	1.17	0.0066	718	1315	25.5	0.43	2.09	493
36	0.4377	0.2984	0.7156	0.1476	0.0949	97.2	1258	60.6	1.10	0.0030	587	48	1162	50.1	0.33	1.58	404	65	1427	41.9	1.33	0.0071	730	1331	29.6	0.45	2.19	485
36	0.4382	0.2974	0.8228	0.1452	0.0988	101.0	1288	52.7	2.47	0.0080	644	0	1200	47.4	0.51	0.84	431	19	1487	48.8	5.67	0.0000	883	1408	35.6	0.92	0.30	638
36	0.4384	0.2960	0.7388	0.1458	0.0965	98.2	1294	66.5	1.95	0.0050	668	33	1197	57.3	0.45	1.14	425	32	1491	52.3	3.18	0.0003	777	1404	37.8	0.71	0.75	588
36	0.4384	0.2993	0.7250	0.1468	0.0949	95.2	1254	62.8	0.98	0.0100	599	55	1157	33.3	0.34	1.75	412	66	1417	43.4	1.12	0.0016	647	1319	32.4	0.41	2.69	446
36	0.4386	0.2976	0.8250	0.1466	0.0943	100.4	1273	62.9	1.08	0.0070	657	41	1172	43.1	0.34	1.37	412	55	1443	33.9	1.34	0.0031	784	1349	31.3	0.48	1.55	553
36	0.4399	0.2971	0.8642	0.1468	0.0961	95.1	1284	71.0	1.31	0.0040	677	30	1186	53.6	0.38	1.15	430	44	1465	57.9	1.76	0.0010	810	1375	40.4	0.55	1.07	569
36	0.4400	0.2965	0.8978	0.1469	0.0965	95.1	1291	67.7	1.49	0.0030	714	0	1195	54.6	0.40	1.17	465	42	1478	54.8	2.06	0.0003	835	1390	40.4	0.61	0.79	628
36	0.4409	0.2987	0.8536	0.1453	0.0976	94.7	1275	73.6	1.43	0.0040	631	8	1187	60.9	0.40	1.08	451	42	1461	57.0	2.23	0.0007	847	1373	42.1	0.61	0.72	618
36	0.4423	0.2977	0.8822	0.1474	0.0974	94.6	1282	67.7	1.61	0.0030	668	0	1190	55.5	0.42	0.97	454	39	1467	59.2	2.44	0.0000	844	1382	48.9	0.66	0.68	623

Continuation of Table S1.

c_2	q_H	σ_{OO}	ϵ_{OO}	d_{OO}	d_{OH}	a_{HOO}	ρ	ϵ	μ	P_v	λ	wt%$_v$	ρ	ϵ	μ	P_v	λ	wt%$_v$	ρ	ϵ	μ	P_v	λ	ρ	ϵ	μ	P_v	λ
36	0.4434	0.2966	0.7764	0.1472	0.0974	96.5	1291	73.6	2.31	0.0050	645	21	1199	59.3	0.48	0.95	459	24	1489	60.1	4.61	0.0008	829	1407	45.1	0.84	0.44	598
36	0.4437	0.2972	0.8770	0.1473	0.0950	94.2	1277	90.4	1.23	0.0160	663	22	1184	51.1	0.36	1.22	460	52	1458	45.6	1.48	0.0010	829	1366	40.5	0.51	1.16	588
36	0.4437	0.2984	0.8274	0.1459	0.0954	98.8	1273	65.1	1.34	0.0080	649	31	1177	50.0	0.37	1.10	444	42	1449	44.9	1.82	0.0000	810	1360	31.5	0.56	1.05	579
36	0.4438	0.2993	0.8214	0.1468	0.0955	96.2	1260	61.0	1.18	0.0040	609	23	1168	57.3	0.35	1.24	426	56	1431	47.3	1.49	0.0014	752	1340	40.4	0.51	1.32	524
36	0.4440	0.2975	0.7380	0.1464	0.0985	100.9	1284	68.8	3.46	0.0060	618	0	1201	51.8	0.59	0.80	461	14	1489	57.2	12.49	0.0008	834	1412	42.7	1.18	0.26	656
36	0.4445	0.2963	0.7902	0.1465	0.0954	97.2	1291	63.6	1.65	0.0070	651	42	1195	54.6	0.42	1.11	430	39	1482	50.2	2.62	0.0003	828	1394	42.4	0.65	0.75	596
36	0.4452	0.2976	0.8486	0.1462	0.0976	98.1	1287	67.6	2.23	0.0040	668	26	1197	57.9	0.48	0.87	491	21	1481	56.3	4.38	0.0000	878	1399	45.6	0.82	0.37	632
36	0.4452	0.2983	0.8190	0.1464	0.0969	100.7	1268	22.7	2.34	0.0040	632	0	1188	53.1	0.45	0.95	475	28	1464	52.1	3.78	0.0003	871	1382	36.1	0.76	0.48	622
36	0.4454	0.2996	0.7832	0.1463	0.0987	98.8	1266	63.6	2.40	0.0070	612	0	1187	50.5	0.51	0.85	475	21	1458	61.6	5.92	0.0000	865	1380	44.1	0.93	0.40	608
36	0.4454	0.2999	0.8908	0.1464	0.0961	94.0	1261	64.8	1.14	0.0050	650	44	1172	57.3	0.36	1.10	440	51	1430	54.5	1.48	0.0014	840	1340	40.6	0.50	1.15	569
36	0.4460	0.2995	0.8646	0.1463	0.0967	101.4	1268	63.3	1.69	0.0020	631	0	1180	44.1	0.42	0.82	450	32	1444	42.2	2.96	0.0000	848	1361	34.3	0.69	0.58	662
36	0.4473	0.2999	0.8552	0.1462	0.0953	95.0	1259	70.0	1.11	0.0050	609	0	1170	55.8	0.35	1.24	443	50	1427	53.9	1.41	0.0011	802	1337	37.8	0.49	1.30	542
36	0.4483	0.2971	0.8088	0.1465	0.0971	99.0	1292	66.8	2.69	0.0060	650	32	1201	55.0	0.52	0.91	468	19	1488	54.1	5.87	0.0009	851	1408	44.0	0.94	0.33	621
36	0.4487	0.2976	0.7572	0.1478	0.0976	98.1	1281	78.5	2.82	0.0060	630	0	1195	59.6	0.55	0.87	468	24	1479	55.1	7.46	0.0000	857	1400	47.7	1.01	0.29	610
36	0.4502	0.2999	0.7036	0.1451	0.0974	98.7	1268	74.2	2.60	0.0050	645	30	1188	56.1	0.52	0.89	487	24	1459	56.5	6.62	0.0000	819	1380	44.9	0.92	0.36	589
36	0.4507	0.2974	0.8436	0.1475	0.0967	101.3	1286	61.3	2.55	0.0120	674	0	1197	50.5	0.52	0.93	440	21	1478	48.1	6.02	0.0000	884	1400	40.1	0.94	0.36	668
36	0.4515	0.2966	0.8114	0.1472	0.0972	97.5	1293	67.2	2.98	0.0040	674	0	1207	64.1	0.55	0.93	472	15	1498	56.3	7.56	0.0000	891	1420	52.4	1.05	0.28	674
36	0.4525	0.2972	0.8394	0.1455	0.0987	96.7	1301	84.4	4.12	0.0100	655	0	1216	68.2	0.65	0.85	498	9	1510	60.1	13.16	0.0000	906	1436	57.9	1.35	0.13	729
36	0.4526	0.2972	0.8608	0.1468	0.0951	97.1	1288	66.6	1.75	0.0060	673	0	1196	60.2	0.44	1.12	454	28	1475	54.8	2.77	0.0000	838	1391	42.8	0.70	0.56	588
36	0.4530	0.2997	0.8018	0.1473	0.0981	97.0	1266	75.1	2.52	0.0080	647	0	1188	61.1	0.52	0.81	466	18	1457	67.4	6.62	0.0000	841	1380	49.8	0.98	0.28	627
36	0.4535	0.2998	0.8200	0.1470	0.0977	100.6	1265	58.0	2.58	0.0100	639	0	1187	56.7	0.53	0.77	472	13	1454	50.4	7.26	0.0000	883	1377	40.2	1.00	0.24	618
36	0.4556	0.2984	0.8738	0.1463	0.0959	98.2	1284	62.8	2.11	0.0040	657	14	1193	57.3	0.46	0.99	465	21	1470	48.8	3.91	0.0000	891	1390	43.8	0.80	0.38	643
36	0.4562	0.2990	0.8100	0.1452	0.0990	96.2	1285	77.6	3.70	0.0040	651	0	1208	66.8	0.65	0.68	511	8	1491	82.5	15.09	0.0000	954	1418	60.0	1.37	0.20	704
36	0.4572	0.2977	0.7618	0.1450	0.0966	99.9	1292	71.4	3.58	0.0090	675	0	1207	53.0	0.59	0.87	467	11	1497	59.6	12.60	0.0000	900	1420	54.5	1.23	0.20	622
36	0.4574	0.2992	0.7522	0.1455	0.0977	98.9	1281	80.1	3.78	0.0040	643	0	1200	57.7	0.62	0.83	476	15	1479	57.6	14.19	0.0000	916	1404	53.6	1.29	0.19	685
36	0.4583	0.2986	0.7040	0.1453	0.0972	96.9	1285	72.5	3.86	0.0040	634	0	1205	68.1	0.63	0.78	482	14	1490	72.3	14.38	0.0000	872	1415	56.4	1.30	0.31	625
36	0.4587	0.2990	0.7876	0.1458	0.0983	96.2	1285	106.2	3.79	0.0060	658	0	1205	68.1	0.64	0.76	487	11	1486	58.5	14.83	0.0000	914	1412	55.6	1.34	0.19	699
36	0.4592	0.2975	0.7450	0.1454	0.0980	100.5	1299	74.2	6.73	0.0050	655	0	1217	60.7	0.79	0.88	473	6	1512	60.6	55.19	0.0000	1007	1441	48.6	1.96	0.08	685
36	0.4594	0.2972	0.7726	0.1463	0.0941	97.2	1291	73.4	2.04	0.0100	660	0	1198	56.3	0.46	0.99	452	31	1482	61.0	3.83	0.0000	809	1399	45.5	0.78	0.57	615
36	0.4607	0.2968	0.7474	0.1470	0.0941	95.5	1293	84.7	2.19	0.0050	678	14	1200	60.6	0.48	1.06	477	32	1487	65.9	4.25	0.0000	831	1404	48.8	0.81	0.56	612
36	0.4607	0.2975	0.8596	0.1477	0.0975	95.3	1296	86.7	3.65	0.0040	660	0	1210	63.8	0.62	0.86	478	11	1497	63.8	11.20	0.0000	910	1422	57.5	1.28	0.14	742
36	0.4633	0.2998	0.8208	0.1459	0.0944	97.3	1269	79.1	1.75	0.0050	621	21	1183	57.1	0.43	0.94	460	33	1449	58.2	3.04	0.0013	815	1368	49.3	0.71	0.50	605
36	0.4642	0.2992	0.8934	0.1468	0.0983	97.0	1282	78.2	3.87	0.0080	650	0	1207	64.9	0.66	0.76	523	5	1483	56.8	16.59	0.0000	975	1413	62.3	1.48	0.10	792
36	0.4648	0.2978	0.7120	0.1473	0.0974	96.3	1296	94.8	6.08	0.0030	636	0	1214	69.0	0.77	0.87	472	8	1504	61.5	39.70	0.0000	885	1433	64.1	1.89	0.11	740
36	0.4654	0.2968	0.7738	0.1479	0.0957	100.6	1296	66.2	5.13	0.0030	680	0	1211	60.6	0.68	0.91	492	8	1501	56.9	23.76	0.0000	920	1427	49.0	1.52	0.12	692
36	0.4660	0.2999	0.8702	0.1476	0.0986	97.8	1276	82.2	4.55	0.0030	658	0	1202	64.0	0.71	0.75	526	2	1472	75.2	27.02	0.0000	917	1402	60.2	1.63	0.09	722
36	0.4664	0.2971	0.8866	0.1468	0.0972	95.8	1304	85.5	4.82	0.0070	672	0	1221	75.7	0.69	0.84	512	5	1512	87.4	19.43	0.0000	983	1440	70.8	1.51	0.09	770
36	0.4668	0.2975	0.7250	0.1467	0.0941	98.7	1293	67.8	3.20	0.0050	671	0	1203	55.0	0.55	1.01	454	21	1487	62.5	8.87	0.0000	795	1410	54.1	1.07	0.33	626
36	0.4669	0.2995	0.7932	0.1460	0.0952	96.1	1277	79.3	2.37	0.0060	645	0	1194	62.4	0.50	0.86	472	19	1466	65.8	5.73	0.0000	869	1388	58.6	0.93	0.33	676
36	0.4672	0.2985	0.8996	0.1473	0.0958	95.5	1289	78.2	2.78	0.0050	665	0	1202	66.5	0.53	0.86	490	14	1479	79.8	6.43	0.0000	928	1402	54.4	1.02	0.25	740
36	0.4672	0.2991	0.7610	0.1474	0.0983	96.1	1285	83.4	5.80	0.0020	686	0	1209	67.1	0.77	0.72	496	6	1490	58.0	49.03	0.0000	903	1421	72.9	2.01	0.06	705
36	0.4679	0.2985	0.7670	0.1453	0.0950	95.6	1286	86.8	2.71	0.0060	658	13	1204	59.2	0.53	0.91	466	18	1487	66.8	7.38	0.0000	834	1410	66.4	1.04	0.32	659
36	0.4682	0.2999	0.8030	0.1467	0.0978	98.8	1278	89.5	4.94	0.0050	635	0	1202	59.0	0.71	0.76	531	7	1476	56.7	33.15	0.0000	950	1406	55.2	1.73	0.09	751
36	0.4692	0.2995	0.8164	0.1462	0.0944	94.7	1276	94.7	1.99	0.0070	643	0	1191	65.4	0.47	0.95	444	25	1461	70.0	3.96	0.0000	854	1381	57.3	0.81	0.50	647
36	0.4507	0.2979	0.8296	0.1477	0.0988	95.3	1285	82.2	3.19	0.0060	640	0	1203	58.5	0.57	0.81	484	11	1486	52.4	8.95	0.0000	878	1410	54.8	1.12	0.19	667
36	0.4516	0.2968	0.8576	0.1459	0.0978	98.7	1303	76.9	3.46	0.0040	716	0	1213	57.0	0.59	0.86	486	10	1506	65.8	9.83	0.0000	953	1429	49.2	1.17	0.19	733
36	0.4679	0.2961	0.8800	0.1472	0.0988	100.5	1315	76.8	15.49	0.0040	735	0	1236	72.0	1.08	0.88	522	1	1536	30.4	249.73	0.0000	1008	1470	63.2	3.42	0.02	837
36	0.4525	0.2981	0.8172	0.1456	0.0986	101.0	1290	67.2	4.36	0.0030	657	32	1207	54.3	0.65	0.92	511	7	1492	42.6	18.11	0.0000	943	1419	45.0	1.38	0.12	695
36	0.4390	0.2961	0.8652	0.1478	0.0940	96.1	1282	63.8	1.04	0.0100	691	43	1185	47.4	0.34	1.58	431	58	1457	41.1	1.19	0.0033	809	1361	31.4	0.45	1.81	483
36	0.4311	0.2999	0.7648	0.1472	0.0957	98.8	1242	57.6	0.92	0.0050	582	49	1149	45.2	0.31	1.76	422	67	1401	35.0	1.02	0.0090	683	1302	25.9	0.39	2.58	454

Continuation of Table S1.

c_2	q_H	σ_{OO}	ϵ_{OO}	d_{OO}	d_{OH}	a_{HOO}	ρ	ϵ	μ	P_v	λ	wt%$_v$	ρ	ϵ	μ	P_v	λ	wt%$_v$	ρ	ϵ	μ	P_v	λ	ρ	ϵ	μ	P_v	λ
36	0.4460	0.2989	0.7446	0.1474	0.0956	97.8	1264	62.3	1.51	0.0020	595	0	1173	47.9	0.40	1.15	455	48	1441	54.2	2.32	0.0002	787	1354	39.6	0.62	0.98	544
36	0.4534	0.2995	0.7628	0.1452	0.0964	100.1	1271	66.8	2.27	0.0040	616	0	1187	56.6	0.48	0.86	467	20	1459	49.4	5.12	0.0004	829	1379	45.3	0.85	0.38	623
36	0.4685	0.2967	0.7340	0.1476	0.0968	100.2	1304	91.3	10.24	0.0070	711	0	1222	61.2	0.89	0.91	468	6	1516	25.8	120.13	0.0000	897	1448	55.4	2.58	0.06	756
36	0.4405	0.2991	0.8502	0.1472	0.0985	99.1	1265	59.8	1.94	0.0070	636	0	1182	47.1	0.45	0.86	485	30	1451	51.0	3.52	0.0000	852	1370	39.3	0.74	0.47	627
36	0.4343	0.2978	0.8356	0.1451	0.0965	101.3	1277	57.6	1.35	0.0070	637	22	1181	44.5	0.38	1.19	436	46	1458	36.6	1.89	0.0017	792	1369	31.2	0.56	0.95	570
36	0.4677	0.2984	0.8386	0.1468	0.0951	97.7	1285	84.0	2.77	0.0060	694	0	1201	62.2	0.54	0.91	489	16	1478	59.7	7.31	0.0000	926	1401	51.4	1.02	0.28	702
36	0.4548	0.2993	0.8766	0.1457	0.0948	99.2	1274	63.9	1.56	0.0080	652	0	1183	47.0	0.40	0.93	454	34	1449	44.5	2.46	0.0000	841	1365	40.0	0.63	0.70	600
36	0.4646	0.2978	0.8080	0.1454	0.0958	99.8	1294	79.9	3.74	0.0060	688	0	1209	54.4	0.61	0.79	507	12	1496	53.0	12.67	0.0000	878	1421	47.6	1.25	0.20	725
36	0.4621	0.2970	0.8116	0.1464	0.0942	99.6	1295	70.7	2.47	0.0060	701	37	1203	49.1	0.50	0.93	447	18	1488	54.6	5.24	0.0000	858	1408	41.6	0.89	0.33	635
36	0.4381	0.2996	0.7512	0.1464	0.0966	98.2	1255	56.4	1.30	0.0070	628	6	1167	45.0	0.37	1.13	438	51	1430	38.9	1.82	0.0020	738	1340	35.5	0.53	1.17	497
36	0.4684	0.2991	0.7040	0.1452	0.0949	96.8	1287	76.4	2.98	0.0040	624	0	1200	60.5	0.56	0.90	452	21	1480	81.8	9.39	0.0000	827	1403	57.2	1.11	0.36	669
36	0.4412	0.2993	0.7514	0.1467	0.0975	102.0	1261	59.3	1.88	0.0060	611	0	1177	49.4	0.45	0.93	466	28	1446	38.0	3.83	0.0000	821	1363	35.5	0.74	0.56	575
36	0.4366	0.2981	0.8442	0.1480	0.0967	96.1	1269	61.4	1.28	0.0080	632	20	1173	45.2	0.37	1.21	469	50	1443	46.4	1.64	0.0022	805	1352	35.1	0.52	1.23	519
36	0.4467	0.2994	0.7098	0.1478	0.0985	98.5	1264	57.2	2.79	0.0030	628	0	1186	54.7	0.55	0.82	429	21	1457	52.6	7.84	0.0000	789	1379	45.8	1.04	0.29	588
36	0.4422	0.2985	0.7924	0.1460	0.0974	96.3	1271	60.3	1.64	0.0060	622	0	1185	51.8	0.42	1.07	458	33	1461	52.1	2.84	0.0000	828	1376	42.6	0.67	0.73	612
36	0.4528	0.2982	0.8910	0.1466	0.0955	98.2	1279	63.9	1.72	0.0070	672	0	1190	52.5	0.43	1.00	484	30	1464	55.5	2.89	0.0000	844	1382	39.8	0.71	0.46	623
36	0.4345	0.2993	0.7008	0.1456	0.0958	100.4	1252	48.2	1.12	0.0070	609	65	1163	41.4	0.35	1.34	427	59	1427	37.0	1.56	0.0004	713	1333	26.8	0.48	1.81	431
36	0.4570	0.2972	0.7290	0.1476	0.0954	101.1	1284	56.6	3.12	0.0100	611	0	1198	52.0	0.56	0.94	479	15	1483	43.2	9.04	0.0000	847	1404	42.0	1.08	0.30	643
36	0.4320	0.2986	0.8640	0.1468	0.0984	95.6	1269	67.2	1.33	0.0140	648	7	1178	50.7	0.38	1.09	446	40	1447	53.9	1.77	0.0000	825	1359	36.7	0.56	1.06	561
36	0.4587	0.2974	0.8038	0.1454	0.0943	97.2	1290	72.6	1.97	0.0070	676	0	1198	56.0	0.45	0.97	474	30	1482	57.2	3.47	0.0000	848	1399	46.3	0.75	0.54	659
36	0.4666	0.2973	0.8148	0.1467	0.0961	97.1	1299	75.3	4.19	0.0040	681	0	1214	68.8	0.63	0.94	489	11	1503	63.3	15.08	0.0000	951	1429	58.7	1.32	0.12	750
36	0.4517	0.2971	0.7788	0.1466	0.0980	94.5	1298	81.7	3.51	0.0030	657	0	1210	63.5	0.58	0.82	476	16	1502	64.5	8.93	0.0000	919	1426	59.9	1.13	0.28	667
36	0.4439	0.2980	0.8384	0.1466	0.0951	94.3	1275	67.5	1.16	0.0040	653	0	1178	54.0	0.35	1.26	405	53	1449	52.7	1.45	0.0016	781	1357	41.0	0.50	1.43	546
36	0.4556	0.2987	0.8700	0.1462	0.0951	97.6	1280	63.0	1.69	0.0060	677	46	1188	55.2	0.42	1.00	458	29	1460	57.1	2.83	0.0000	845	1376	43.9	0.69	0.61	614
36	0.4529	0.2996	0.8122	0.1474	0.0965	98.3	1262	69.5	2.03	0.0030	625	0	1180	53.4	0.45	0.87	480	26	1446	51.7	3.78	0.0000	825	1364	44.1	0.76	0.46	627
36	0.4465	0.2968	0.7906	0.1472	0.0950	96.9	1285	62.8	1.54	0.0120	652	18	1187	51.9	0.40	1.23	430	38	1470	50.6	2.31	0.0000	815	1382	40.3	0.62	1.04	550
36	0.4348	0.2976	0.7368	0.1478	0.0942	97.3	1262	50.4	0.98	0.0160	605	74	1160	45.2	0.31	1.82	386	69	1428	41.8	1.09	0.0045	660	1328	29.1	0.41	2.72	433
36	0.4471	0.2964	0.7182	0.1466	0.0959	96.9	1292	70.6	2.20	0.0040	642	0	1200	54.4	0.48	1.07	437	32	1491	57.3	4.43	0.0000	806	1408	47.1	0.82	0.67	584
36	0.4383	0.2998	0.7434	0.1456	0.0950	101.7	1251	54.4	1.11	0.0060	610	16	1159	43.7	0.33	1.44	416	58	1418	32.6	1.41	0.0055	708	1324	25.2	0.47	1.79	458
36	0.4670	0.2979	0.7826	0.1457	0.0978	99.6	1296	78.8	7.20	0.0040	653	0	1220	58.2	0.88	0.77	527	3	1512	35.1	83.27	0.0000	979	1442	62.2	2.32	0.06	748
36	0.4423	0.2988	0.8180	0.1466	0.0941	97.1	1263	57.0	0.99	0.0070	611	37	1163	50.0	0.32	1.57	452	64	1426	43.5	1.14	0.0017	759	1329	31.0	0.44	2.21	494
36	0.4422	0.2993	0.8166	0.1471	0.0978	96.2	1265	77.1	1.66	0.0030	640	19	1179	54.1	0.42	1.03	432	37	1446	53.3	2.61	0.0018	825	1361	45.2	0.66	0.71	615
36	0.4578	0.2999	0.8544	0.1469	0.0973	98.7	1270	72.5	2.59	0.0030	646	0	1189	55.9	0.52	0.81	481	18	1456	57.8	6.77	0.0000	880	1380	46.8	0.98	0.30	683
36	0.4372	0.2983	0.8078	0.1462	0.0967	98.5	1272	65.3	1.39	0.0040	647	0	1178	49.7	0.38	1.08	448	44	1450	44.9	1.96	0.0000	796	1362	33.0	0.57	0.99	556
36	0.4460	0.2991	0.7112	0.1462	0.0951	96.7	1264	61.2	1.35	0.0030	590	47	1171	48.8	0.37	1.20	440	52	1439	51.2	1.95	0.0017	761	1349	37.1	0.55	1.42	487
36	0.4587	0.2973	0.7418	0.1462	0.0969	97.7	1294	73.1	3.91	0.0060	663	0	1211	59.9	0.65	0.86	492	8	1504	66.0	16.37	0.0003	848	1429	54.7	1.37	0.19	668
36	0.4433	0.2967	0.8034	0.1474	0.0970	100.1	1284	60.3	2.31	0.0030	644	0	1196	51.9	0.49	1.12	489	22	1482	47.0	4.45	0.0000	827	1400	37.2	0.83	0.40	637
36	0.4313	0.2996	0.7972	0.1473	0.0943	96.8	1243	56.0	0.77	0.0180	595	82	1142	45.0	0.27	2.44	361	75	1391	34.2	0.73	0.0037	674	1286	25.6	0.33	4.09	399
36	0.4628	0.2996	0.8156	0.1465	0.0948	98.4	1272	71.6	2.07	0.0040	650	0	1185	57.9	0.46	0.87	475	24	1452	62.1	4.04	0.0000	827	1372	46.6	0.79	0.49	597
36	0.4399	0.2981	0.8016	0.1451	0.0974	95.4	1282	72.0	1.59	0.0050	624	0	1188	47.7	0.41	1.07	472	37	1468	54.7	2.43	0.0003	795	1382	47.0	0.64	0.75	587
36	0.4484	0.2985	0.8142	0.1479	0.0948	98.0	1267	63.7	1.34	0.0020	624	0	1173	46.1	0.38	1.11	445	41	1440	47.4	1.94	0.0010	789	1352	35.1	0.56	1.02	585
36	0.4534	0.2976	0.8736	0.1461	0.0973	94.2	1295	73.8	2.32	0.0050	639	0	1205	56.0	0.52	0.96	481	20	1490	72.8	4.72	0.0007	888	1411	52.3	0.92	0.37	696
36	0.4360	0.2969	0.8168	0.1450	0.0942	99.6	1280	59.5	1.06	0.0060	638	80	1178	41.3	0.33	1.59	416	60	1455	35.6	1.24	0.0031	784	1359	25.7	0.45	1.67	511
36	0.4498	0.2981	0.8078	0.1472	0.0947	99.9	1272	58.1	1.54	0.0040	658	0	1178	54.6	0.39	1.09	432	38	1452	44.6	2.39	0.0000	782	1366	34.1	0.62	0.77	573
36	0.4582	0.2993	0.7668	0.1469	0.0959	94.1	1271	76.5	1.97	0.0080	642	0	1187	63.7	0.46	0.92	462	31	1458	75.7	3.83	0.0002	784	1376	52.6	0.77	0.50	616
36	0.4337	0.2971	0.7298	0.1467	0.0967	96.4	1276	64.1	1.39	0.0100	603	64	1180	51.3	0.39	1.23	418	48	1462	50.9	2.02	0.0057	752	1370	38.1	0.56	1.23	564
36	0.4592	0.2987	0.8740	0.1462	0.0954	97.2	1282	80.9	2.01	0.0040	664	0	1193	57.9	0.46	1.05	481	24	1466	58.8	3.80	0.0000	831	1385	47.7	0.79	0.40	694
36	0.4604	0.2991	0.7460	0.1476	0.0988	100.3	1281	67.4	6.65	0.0040	665	0	1203	55.4	0.79	0.77	511	7	1483	71.0	59.87	0.0000	913	1413	58.2	2.07	0.07	667
36	0.4681	0.2995	0.8638	0.1455	0.0988	95.8	1289	92.8	5.49	0.0070	670	0	1214	68.6	0.74	0.81	522	3	1493	88.3	33.41	0.0000	949	1425	69.2	1.86	0.06	772
36	0.4387	0.2966	0.8422	0.1450	0.0958	95.9	1291	71.3	1.33	0.0050	658	33	1193	47.5	0.39	1.26	403	46	1478	50.9	1.76	0.0009	826	1387	37.0	0.55	1.09	565

Table S2. Simulation results employed for measuring the deviations of the ANNs' predictions. Legth units are given in nm, charge in elementary charge unit, angles in degrees, energy in kJ/mol, ρ in kg/m^3, ϵ is dimensionless, μ in cP, P_v in bar, and λ in 10^{-4} N/m.

Model Parameters							70wt%												100wt%									
							293K						373K			398K			293K					373K			423K	
c_2	q_H	σ_{OO}	ϵ_{OO}	d_{OO}	d_{OH}	a_{HOO}	ρ	ϵ	μ	P_v	λ	wt%$_v$	ρ	ϵ	μ	P_v	λ	wt%$_v$	ρ	ϵ	μ	P_v	λ	ρ	ϵ	μ	P_v	λ
25.5	0.4400	0.2985	0.7260	0.1462	0.0969	99.3	1267	44.3	1.64	0.0050	645	0	1180	61.6	0.42	0.92	427	37	1455	39.8	3.14	0.0023	782	1369	35.9	0.67	0.81	538
25.5	0.4310	0.2975	0.7342	0.1476	0.0968	95.7	1269	65.0	1.24	0.0080	578	52	1172	49.2	0.36	1.35	459	59	1447	44.6	1.58	0.0002	709	1352	33.6	0.50	1.59	525
25.5	0.4464	0.2961	0.7612	0.1476	0.0952	98.1	1291	63.5	1.87	0.0140	656	0	1195	51.5	0.44	1.08	442	35	1482	44.1	3.15	0.0004	782	1397	39.5	0.72	0.65	591
25.5	0.4512	0.2980	0.7060	0.1474	0.0979	96.4	1281	92.8	3.35	0.0270	636	0	1200	59.0	0.58	0.76	474	18	1484	64.7	11.63	0.0000	816	1408	55.8	1.20	0.28	650
25.5	0.4452	0.2978	0.7322	0.1479	0.0950	95.4	1269	67.9	1.41	0.0140	603	25	1175	51.2	0.38	1.26	431	52	1450	54.4	1.94	0.0000	734	1359	37.6	0.56	1.37	542
25.5	0.4330	0.2989	0.8244	0.1453	0.0951	94.1	1259	68.7	0.83	0.0110	621	53	1159	51.2	0.29	2.04	423	72	1421	44.2	0.85	0.0031	722	1319	31.7	0.37	3.15	462
25.5	0.4400	0.2983	0.8354	0.1457	0.0958	96.9	1271	59.8	1.24	0.0060	643	7	1177	52.5	0.37	1.29	448	47	1449	51.6	1.59	0.0023	817	1358	35.2	0.52	1.17	520
25.5	0.4502	0.2961	0.8870	0.1469	0.0974	98.4	1296	62.9	3.09	0.0020	727	0	1218	50.2	0.58	1.07	513	16	1507	56.3	7.47	0.0000	957	1429	43.2	1.05	0.21	696
27	0.4433	0.2992	0.8678	0.1459	0.0980	101.0	1273	75.5	2.21	0.0050	610	0	1191	66.1	0.49	0.78	466	16	1461	68.0	4.51	0.0000	925	1383	52.6	0.84	0.27	668
27	0.4304	0.2972	0.8130	0.1457	0.0972	100.7	1285	75.5	1.53	0.0120	635	24	1187	56.3	0.40	1.01	458	33	1469	59.1	2.30	0.0000	799	1382	45.2	0.63	0.71	576
27	0.4333	0.2971	0.8196	0.1472	0.0955	97.0	1278	87.9	1.16	0.0100	648	45	1179	64.0	0.36	1.24	438	49	1454	66.5	1.42	0.0000	794	1362	54.0	0.49	1.22	570
27	0.4351	0.2971	0.7192	0.1480	0.0986	94.7	1284	101.8	2.27	0.0040	576	0	1196	75.3	0.49	0.91	478	25	1482	99.4	4.25	0.0000	766	1401	73.5	0.84	0.48	621
27	0.4394	0.2962	0.7734	0.1452	0.0965	100.6	1298	82.0	2.30	0.0070	666	16	1204	59.8	0.47	1.02	499	25	1497	87.0	4.36	0.0000	810	1415	57.0	0.81	0.42	623
27	0.4326	0.2968	0.7690	0.1458	0.0953	98.1	1282	83.0	1.22	0.0090	655	31	1182	57.7	0.36	1.35	447	55	1463	68.9	1.53	0.0034	757	1371	48.0	0.51	1.34	523
27	0.4357	0.2979	0.7538	0.1473	0.0945	101.1	1267	70.5	1.14	0.0100	621	26	1167	52.4	0.34	1.36	443	56	1436	49.1	1.45	0.0006	761	1344	40.3	0.48	1.38	523
27	0.4451	0.2985	0.7470	0.1455	0.0968	98.6	1280	90.5	2.21	0.0050	614	0	1192	72.5	0.47	0.83	474	22	1471	87.3	4.35	0.0000	838	1390	64.3	0.81	0.40	593
27	0.4417	0.2973	0.7064	0.1465	0.0950	94.2	1283	94.7	1.37	0.0080	594	0	1186	73.4	0.39	1.29	446	52	1469	90.5	1.95	0.0019	755	1378	62.6	0.56	1.21	533
27	0.4684	0.2965	0.7198	0.1467	0.0972	98.1	1316	86.9	12.93	0.0040	705	0	1236	96.9	1.05	0.89	498	2	1537	59.6	174.01	0.0000	977	1472	117.3	3.27	0.03	803
27	0.4518	0.2964	0.8534	0.1454	0.0944	97.5	1302	94.2	1.84	0.0110	724	0	1210	69.4	0.46	0.98	441	27	1495	91.2	2.93	0.0000	862	1412	58.5	0.73	0.41	636
27	0.4670	0.2962	0.7926	0.1471	0.0962	101.6	1309	99.2	8.14	0.0030	699	0	1229	81.6	0.87	0.94	502	3	1522	59.9	67.99	0.0000	935	1453	81.8	2.16	0.06	759
27	0.4329	0.2988	0.8852	0.1456	0.0942	100.8	1263	63.1	0.89	0.0210	666	19	1165	47.4	0.31	1.59	428	61	1425	49.0	1.01	0.0040	795	1329	33.6	0.41	1.77	508
27	0.4610	0.2984	0.8532	0.1465	0.0962	101.3	1289	90.0	3.66	0.0060	663	0	1204	76.8	0.60	0.79	501	10	1482	78.9	11.60	0.0000	908	1409	67.9	1.23	0.13	724
27	0.4511	0.2976	0.7590	0.1464	0.0941	100.7	1282	78.5	1.86	0.0040	643	21	1189	68.7	0.45	0.99	438	37	1467	73.4	3.15	0.0000	800	1383	44.6	0.70	0.52	579
27	0.4523	0.2965	0.7000	0.1453	0.0954	98.6	1299	107.0	3.28	0.0060	659	0	1212	79.4	0.56	0.88	487	22	1507	83.7	7.51	0.0000	817	1429	77.4	1.05	0.28	661
27	0.4426	0.2963	0.8794	0.1456	0.0986	97.7	1306	92.1	3.00	0.0120	684	0	1217	76.6	0.57	0.84	491	6	1513	104.2	7.04	0.0000	948	1436	76.5	1.07	0.14	723
27	0.4690	0.2962	0.7304	0.1457	0.0944	94.8	1316	145.9	4.61	0.0050	713	0	1230	104.5	0.68	0.92	468	10	1531	157.0	14.61	0.0000	876	1456	133.9	1.38	0.12	675
27	0.4606	0.2972	0.7052	0.1452	0.0982	97.2	1312	138.6	9.55	0.0060	675	0	1232	106.0	0.93	0.86	493	9	1535	79.7	110.12	0.0000	955	1464	113.3	2.50	0.05	748
27	0.4547	0.2987	0.7782	0.1470	0.0943	101.4	1273	82.4	1.86	0.0050	644	8	1184	61.4	0.45	0.92	461	25	1453	69.4	3.42	0.0000	805	1372	51.0	0.74	0.46	584
27	0.4306	0.2986	0.8154	0.1469	0.0955	97.8	1262	76.7	0.98	0.0120	609	81	1164	58.3	0.33	1.44	413	60	1427	58.5	1.12	0.0013	792	1332	39.2	0.43	1.62	524
27	0.4592	0.2990	0.8222	0.1460	0.0948	98.3	1280	102.1	2.06	0.0050	645	56	1193	75.1	0.47	0.88	462	19	1465	89.5	3.76	0.0000	856	1387	63.6	0.81	0.30	638
27	0.4316	0.2998	0.7368	0.1457	0.0957	96.5	1251	79.2	0.98	0.0040	617	81	1159	61.2	0.32	1.52	435	61	1418	65.1	1.11	0.0015	687	1321	46.8	0.41	2.02	464
27	0.4616	0.2979	0.7150	0.1453	0.0971	99.3	1300	101.9	6.12	0.0040	640	0	1218	88.0	0.78	0.78	493	4	1510	87.3	42.44	0.0000	892	1440	87.9	1.94	0.08	696
27	0.4689	0.2986	0.8914	0.1476	0.0966	97.2	1292	104.8	4.31	0.0010	661	0	1212	86.5	0.66	0.81	513	6	1489	116.9	15.60	0.0000	969	1419	96.4	1.45	0.06	771
28.5	0.4407	0.2993	0.8584	0.1474	0.0951	94.7	1262	78.9	1.01	0.0050	619	52	1167	64.7	0.34	1.26	422	50	1425	67.8	1.20	0.0024	771	1334	50.2	0.46	1.52	504
28.5	0.4329	0.2972	0.8618	0.1469	0.0983	96.6	1285	84.8	1.72	0.0110	665	3	1193	59.4	0.43	1.13	474	31	1474	68.2	2.59	0.0000	905	1390	57.7	0.68	0.48	630
28.5	0.4389	0.2976	0.7826	0.1471	0.0990	96.3	1284	89.8	2.45	0.0040	647	0	1199	67.4	0.52	0.88	489	18	1485	83.6	5.51	0.0000	872	1406	66.5	0.94	0.25	608
28.5	0.4308	0.2980	0.7638	0.1468	0.0944	98.2	1263	67.9	0.91	0.0030	610	59	1160	53.0	0.31	1.76	401	66	1426	50.0	1.00	0.0100	704	1327	37.4	0.40	2.61	450
28.5	0.4645	0.2990	0.7888	0.1468	0.0941	94.2	1280	106.1	1.90	0.0120	662	10	1192	76.2	0.45	0.97	473	28	1465	114.4	3.47	0.0000	830	1384	84.7	0.78	0.42	613
28.5	0.4380	0.2985	0.8610	0.1457	0.0971	100.8	1276	76.1	1.65	0.0090	633	0	1186	56.1	0.42	0.89	436	28	1459	56.5	2.62	0.0010	831	1375	46.1	0.66	0.47	630
28.5	0.4386	0.2962	0.7034	0.1475	0.0978	94.4	1298	100.3	2.43	0.0050	631	0	1204	71.0	0.52	0.88	464	29	1499	105.0	5.07	0.0000	783	1417	68.7	0.88	0.44	599
28.5	0.4688	0.2972	0.8466	0.1470	0.0944	96.9	1301	114.4	3.14	0.0080	679	0	1216	88.9	0.58	0.89	473	13	1500	114.5	7.89	0.0000	917	1424	82.6	1.10	0.18	665
28.5	0.4603	0.2998	0.7838	0.1455	0.0957	96.8	1277	88.1	2.20	0.0020	617	0	1193	79.9	0.50	0.79	483	19	1464	112.7	5.02	0.0000	874	1386	73.0	0.89	0.29	641
28.5	0.4491	0.2972	0.7060	0.1464	0.0948	95.7	1289	96.1	1.83	0.0030	622	0	1194	73.3	0.44	1.08	430	32	1480	83.5	3.03	0.0000	761	1394	68.9	0.71	0.72	546
28.5	0.4372	0.2969	0.8488	0.1454	0.0988	94.4	1299	102.2	2.12	0.0040	629	0	1208	77.2	0.49	0.92	472	17	1499	83.1	3.76	0.0000	869	1418	69.8	0.82	0.37	653
28.5	0.4551	0.2993	0.7276	0.1453	0.0963	100.7	1276	80.4	2.84	0.0040	652	0	1194	70.9	0.54	0.86	462	16	1469	65.5	8.53	0.0000	865	1392	58.0	1.03	0.26	611
28.5	0.4686	0.2997	0.8458	0.1479	0.0976	101.8	1278	82.7	5.82	0.0050	680	0	1203	71.4	0.76	0.77	512	3	1472	89.4	45.31	0.0000	927	1405	72.3	1.94	0.05	754
28.5	0.4556	0.2978	0.7090	0.1468	0.0990	97.6	1298	101.9	7.33	0.0040	690	0	1218	86.6	0.85	0.79	499	4	1511	89.8	67.80	0.0000	926	1442	90.8	2.23	0.07	709
28.5	0.4333	0.2961	0.8768	0.1464	0.0988	101.7	1298	74.2	2.53	0.0080	709	0	1207	56.6	0.51	0.84	461	13	1497	55.3	5.04	0.0000	915	1418	45.3	0.89	0.22	699
28.5	0.4615	0.2974	0.7998	0.1469	0.0980	102.0	1298	70.7	8.08	0.0060	664	0	1219	76.7	0.85	0.83	507	3	1509	39.1	105.27	0.0000	935	1440	67.8	2.25	0.05	746

Continuation of Table S2.

c_2	q_H	σ_{OO}	ϵ_{OO}	d_{OO}	d_{OH}	a_{HOO}	ρ	ϵ	μ	P_v	λ	wt$\%_v$	ρ	ϵ	μ	P_v	λ	wt$\%_v$	ρ	ϵ	μ	P_v	λ	ρ	ϵ	μ	P_v	λ
28.5	0.4399	0.3000	0.8782	0.1468	0.0946	101.9	1255	59.3	1.07	0.0070	634	17	1161	49.0	0.33	1.25	439	50	1413	43.8	1.30	0.0061	783	1323	35.0	0.47	1.33	537
28.5	0.4332	0.2966	0.8308	0.1461	0.0958	96.0	1287	72.1	1.18	0.0030	707	25	1189	56.8	0.37	1.39	431	45	1469	65.1	1.51	0.0010	812	1377	50.5	0.52	1.19	580
28.5	0.4381	0.2977	0.8952	0.1476	0.0965	96.7	1288	88.6	1.21	0.0070	679	35	1188	61.1	0.37	1.32	429	48	1469	60.5	1.51	0.0000	803	1377	48.0	0.52	1.19	561
28.5	0.4684	0.2982	0.8878	0.1475	0.0947	96.2	1291	120.2	2.57	0.0040	700	0	1205	78.2	0.53	0.90	454	14	1481	137.6	5.73	0.0000	924	1405	79.6	0.99	0.18	665
28.5	0.4363	0.2999	0.7764	0.1479	0.0959	98.6	1250	76.3	1.14	0.0090	607	27	1159	53.6	0.34	1.19	428	52	1413	56.9	1.42	0.0003	746	1322	44.7	0.48	1.34	540
28.5	0.4448	0.2982	0.7586	0.1451	0.0948	100.1	1278	73.2	1.51	0.0040	629	73	1183	62.0	0.40	1.08	444	39	1459	64.1	2.33	0.0000	794	1373	45.2	0.62	0.81	569
28.5	0.4430	0.2965	0.8770	0.1461	0.0960	99.5	1295	85.7	1.84	0.0040	705	0	1204	58.3	0.45	1.02	466	21	1487	64.2	2.94	0.0021	870	1404	48.8	0.72	0.42	630
28.5	0.4481	0.2972	0.7900	0.1463	0.0958	95.0	1292	103.9	1.79	0.0050	647	8	1199	70.6	0.45	0.99	468	31	1485	95.4	3.13	0.0000	827	1402	69.8	0.75	0.59	618
28.5	0.4688	0.2973	0.7050	0.1458	0.0943	94.6	1307	125.9	3.64	0.0040	661	0	1217	94.6	0.61	0.94	485	12	1511	171.0	10.47	0.0000	867	1434	100.5	1.20	0.22	698
28.5	0.4571	0.2977	0.8172	0.1469	0.0988	100.4	1296	100.4	6.50	0.0020	692	0	1215	77.5	0.78	0.81	474	3	1506	57.1	47.96	0.0000	928	1436	86.7	2.00	0.06	743
28.5	0.4364	0.2973	0.7488	0.1474	0.0965	98.5	1279	81.7	1.66	0.0020	634	0	1183	54.5	0.41	1.11	473	36	1462	73.5	2.52	0.0007	754	1376	49.5	0.64	0.75	586
28.5	0.4550	0.2976	0.7424	0.1457	0.0967	96.5	1296	96.6	3.43	0.0070	668	0	1210	74.3	0.60	0.90	473	16	1500	86.1	9.47	0.0000	852	1424	77.7	1.15	0.21	672
28.5	0.4689	0.2999	0.8688	0.1470	0.0984	96.7	1283	178.3	4.84	0.0050	658	0	1211	96.6	0.77	0.74	526	6	1483	167.1	30.11	0.0000	974	1416	96.2	1.91	0.04	770
28.5	0.4462	0.2998	0.8500	0.1474	0.0943	100.8	1257	71.4	1.19	0.0140	615	4	1165	57.9	0.36	1.09	441	47	1419	47.2	1.56	0.0016	790	1332	41.0	0.51	0.99	530
28.5	0.4475	0.2985	0.8788	0.1456	0.0986	98.7	1284	88.8	2.81	0.0050	668	0	1204	70.3	0.55	0.78	514	8	1483	85.6	7.54	0.0000	906	1407	65.3	1.06	0.18	689
28.5	0.4325	0.2963	0.8196	0.1452	0.0960	96.6	1292	85.6	1.29	0.0120	672	10	1193	58.3	0.38	1.31	436	46	1480	64.1	1.69	0.0020	781	1388	50.6	0.53	1.02	565
28.5	0.4438	0.2985	0.8820	0.1462	0.0951	101.5	1276	75.1	1.46	0.0040	678	0	1182	55.1	0.39	1.11	464	32	1449	51.3	1.98	0.0004	853	1364	40.1	0.59	0.74	630
28.5	0.4651	0.2989	0.8044	0.1462	0.0958	94.7	1287	137.0	2.79	0.0040	639	0	1205	89.3	0.56	0.85	500	13	1484	122.0	7.32	0.0000	885	1409	95.2	1.08	0.24	699
28.5	0.4620	0.2966	0.8052	0.1471	0.0983	96.5	1310	149.8	8.43	0.0060	692	0	1229	90.1	0.87	0.88	530	3	1529	78.3	58.63	0.0000	936	1460	105.7	2.26	0.06	780
28.5	0.4495	0.2969	0.8980	0.1478	0.0988	100.3	1298	69.7	4.14	0.0060	673	0	1212	72.5	0.65	0.84	512	4	1501	62.3	16.53	0.0000	973	1429	64.2	1.37	0.10	718
28.5	0.4458	0.2969	0.8632	0.1471	0.0987	99.6	1296	93.5	3.21	0.0050	688	0	1210	67.3	0.60	0.82	508	9	1500	83.3	11.51	0.0000	873	1425	69.9	1.22	0.15	722
28.5	0.4493	0.2976	0.8602	0.1451	0.0941	96.3	1288	95.9	1.37	0.0100	667	6	1193	69.5	0.38	1.17	460	41	1470	79.0	1.83	0.0024	852	1382	52.0	0.58	0.82	564
28.5	0.4471	0.2966	0.8812	0.1465	0.0977	99.3	1299	90.4	3.10	0.0240	654	0	1212	62.8	0.57	0.93	484	9	1502	84.4	7.52	0.0000	931	1425	63.2	1.06	0.13	706
28.5	0.4617	0.2967	0.8654	0.1472	0.0948	97.6	1302	104.8	2.69	0.0050	719	0	1216	77.4	0.54	0.91	483	11	1498	119.9	6.09	0.0000	941	1421	66.8	1.00	0.21	680
28.5	0.4416	0.2979	0.7624	0.1461	0.0963	94.2	1280	97.9	1.50	0.0040	618	17	1188	73.0	0.41	1.04	449	40	1467	84.7	2.17	0.0004	762	1380	63.3	0.61	0.95	527
28.5	0.4407	0.2991	0.7124	0.1459	0.0973	94.7	1269	110.4	1.66	0.0030	596	0	1185	76.3	0.43	1.12	450	37	1458	88.9	2.87	0.0008	780	1373	64.1	0.67	0.72	564
28.5	0.4659	0.2997	0.8012	0.1473	0.0943	94.4	1273	113.1	1.85	0.0050	661	0	1188	83.1	0.46	0.97	467	21	1454	101.6	3.33	0.0000	813	1375	83.0	0.77	0.40	595
28.5	0.4382	0.2976	0.8578	0.1479	0.0943	99.8	1270	70.3	1.10	0.0110	652	39	1172	50.6	0.35	1.43	423	49	1438	51.0	1.32	0.0000	772	1347	38.2	0.48	1.32	542
28.5	0.4448	0.2996	0.8892	0.1462	0.0953	99.2	1266	78.7	1.23	0.0050	637	26	1175	59.1	0.37	1.07	462	42	1435	64.1	1.72	0.0009	807	1349	46.5	0.55	0.84	614
28.5	0.4548	0.2999	0.7516	0.1454	0.0944	97.2	1267	90.9	1.51	0.0080	632	26	1178	63.6	0.40	0.99	445	39	1444	96.6	2.46	0.0000	777	1359	57.7	0.63	0.77	582
28.5	0.4477	0.2996	0.8104	0.1452	0.0990	97.1	1277	98.4	2.80	0.0010	680	0	1199	69.2	0.56	0.73	521	10	1473	91.9	7.29	0.0000	902	1398	75.5	1.05	0.16	666
28.5	0.4630	0.2987	0.7906	0.1464	0.0975	100.4	1287	106.2	5.34	0.0060	662	0	1210	84.5	0.74	0.79	497	6	1491	73.8	38.43	0.0000	919	1421	69.5	1.78	0.06	714
28.5	0.4500	0.2967	0.8738	0.1474	0.0982	100.8	1298	93.4	3.94	0.0040	685	0	1212	69.4	0.63	0.90	499	7	1504	57.0	15.21	0.0000	979	1430	66.0	1.36	0.09	782
28.5	0.4430	0.2976	0.7138	0.1476	0.0975	94.0	1284	101.8	2.22	0.0020	595	14	1196	74.7	0.48	0.87	456	26	1480	100.2	4.34	0.0000	829	1399	72.1	0.84	0.44	596
28.5	0.4568	0.2965	0.8448	0.1466	0.0982	96.4	1311	122.8	5.32	0.0050	705	0	1226	82.4	0.73	0.83	506	5	1524	113.8	21.30	0.0000	998	1453	89.4	1.62	0.07	744
30	0.4529	0.2991	0.7778	0.1461	0.0953	98.1	1274	75.5	1.85	0.0020	623	42	1185	59.3	0.43	0.98	475	31	1455	75.6	3.15	0.0002	808	1373	53.8	0.72	0.59	579
30	0.4360	0.2970	0.8714	0.1465	0.0973	100.2	1287	63.8	1.77	0.0060	660	20	1194	53.9	0.44	1.03	436	29	1474	54.4	2.78	0.0000	891	1390	42.3	0.69	0.45	676
30	0.4548	0.2997	0.7138	0.1456	0.0968	99.2	1272	84.4	2.93	0.0060	631	0	1192	59.9	0.55	0.88	511	14	1464	65.8	8.61	0.0000	842	1388	55.6	1.03	0.33	607
30	0.4490	0.2994	0.8696	0.1473	0.0972	96.4	1269	94.1	1.83	0.0050	626	17	1186	63.7	0.45	0.86	498	25	1453	78.9	3.33	0.0000	859	1372	60.0	0.74	0.36	657
30	0.4614	0.2991	0.8034	0.1479	0.0973	97.6	1277	95.2	3.42	0.0060	628	0	1199	80.0	0.62	0.80	502	9	1473	100.1	14.91	0.0000	940	1401	70.1	1.30	0.12	698
30	0.4331	0.2998	0.7580	0.1474	0.0983	94.9	1254	81.6	1.38	0.0090	610	31	1169	66.1	0.38	1.04	444	44	1431	76.4	1.90	0.0000	774	1342	52.7	0.55	1.03	560
30	0.4664	0.2985	0.8584	0.1463	0.0964	101.2	1289	91.1	4.16	0.0030	658	0	1208	64.6	0.66	0.80	481	7	1488	85.7	18.26	0.0000	935	1417	64.7	1.46	0.08	769
30	0.4640	0.2997	0.8356	0.1455	0.0973	100.3	1280	69.6	4.01	0.0040	691	63	1204	73.0	0.65	0.78	511	7	1478	82.1	18.00	0.0000	891	1407	66.4	1.44	0.09	737
30	0.4571	0.2975	0.7668	0.1463	0.0964	100.4	1289	84.8	4.18	0.0040	662	0	1207	72.1	0.61	0.87	497	11	1494	78.6	12.34	0.0000	862	1420	60.2	1.25	0.16	704
30	0.4302	0.2982	0.8026	0.1461	0.0987	98.0	1268	77.2	1.73	0.0160	633	62	1184	61.8	0.42	1.04	463	38	1460	55.5	2.56	0.0000	823	1374	43.4	0.64	0.72	602
30	0.4470	0.2969	0.8856	0.1453	0.0959	102.0	1291	48.5	2.14	0.0040	710	0	1206	53.7	0.48	0.93	477	20	1488	49.2	3.92	0.0000	887	1408	46.7	0.81	0.31	694
30	0.4432	0.2997	0.7260	0.1474	0.0963	100.8	1255	73.2	1.65	0.0090	615	72	1171	51.6	0.41	0.98	434	39	1432	52.8	2.79	0.0000	753	1349	39.1	0.67	0.70	557
30	0.4345	0.2979	0.7924	0.1474	0.0955	101.6	1270	62.4	1.23	0.0460	637	19	1171	45.5	0.36	1.31	438	47	1441	46.4	1.64	0.0020	761	1350	32.1	0.52	1.23	532
30	0.4394	0.2970	0.8878	0.1470	0.0950	101.6	1282	69.3	1.35	0.0090	684	54	1187	48.4	0.38	1.19	479	39	1459	47.8	1.78	0.0018	815	1373	35.1	0.57	0.84	580
30	0.4494	0.2976	0.8388	0.1470	0.0975	95.8	1292	100.6	2.49	0.0090	691	0	1203	70.6	0.52	0.87	481	13	1486	99.0	5.17	0.0000	865	1407	67.6	0.93	0.25	689
30	0.4460	0.2999	0.7644	0.1479	0.0961	94.5	1258	81.5	1.41	0.0050	630	42	1170	65.1	0.38	1.12	434	47	1430	78.9	2.06	0.0000	779	1343	58.2	0.57	1.00	559

Continuation of Table S2.

c_2	q_H	σ_{OO}	ϵ_{OO}	d_{OO}	d_{OH}	a_{HOO}	ρ	ϵ	μ	P_v	λ	wt$\%_v$	ρ	ϵ	μ	P_v	λ	wt$\%_v$	ρ	ϵ	μ	P_v	λ	ρ	ϵ	μ	P_v	λ
30	0.4579	0.2994	0.7138	0.1462	0.0974	97.3	1281	84.6	3.65	0.0110	632	0	1200	79.9	0.62	0.84	490	9	1477	119.3	13.38	0.0000	879	1404	75.1	1.31	0.16	650
30	0.4692	0.2971	0.8768	0.1479	0.0977	99.1	1303	109.7	7.95	0.0060	712	0	1226	85.0	0.88	0.79	514	2	1514	55.9	89.79	0.0000	1024	1448	85.6	2.28	0.05	818
30	0.4644	0.2983	0.7844	0.1478	0.0975	97.3	1288	77.1	5.02	0.0040	649	0	1210	84.1	0.72	0.80	490	4	1495	95.1	30.39	0.0000	915	1424	80.5	1.73	0.08	763
30	0.4343	0.2981	0.8974	0.1455	0.0944	95.8	1271	70.8	0.86	0.0040	677	68	1173	55.2	0.32	1.71	417	61	1435	50.6	0.93	0.0037	804	1338	40.8	0.40	2.09	548
30	0.4603	0.2992	0.8378	0.1452	0.0986	96.5	1290	90.2	4.35	0.0050	699	0	1213	86.8	0.68	0.80	531	9	1494	86.4	19.41	0.0000	923	1423	84.8	1.52	0.09	754
30	0.4651	0.2994	0.8706	0.1462	0.0982	99.3	1285	72.4	5.04	0.0040	641	0	1209	62.9	0.72	0.79	504	5	1485	90.5	30.43	0.0000	983	1416	85.8	1.73	0.06	762
30	0.4677	0.2984	0.7154	0.1473	0.0942	99.3	1282	105.4	3.24	0.0030	645	0	1198	59.5	0.57	0.90	464	13	1476	89.8	10.59	0.0000	842	1401	62.6	1.14	0.25	655
30	0.4352	0.2965	0.7832	0.1472	0.0959	99.9	1280	62.9	1.45	0.0030	650	22	1185	22.2	0.40	1.22	418	41	1467	49.5	2.10	0.0004	762	1379	37.4	0.59	0.83	523
30	0.4578	0.2969	0.8420	0.1456	0.0956	97.5	1303	82.8	2.84	0.0080	695	0	1213	71.3	0.54	0.92	481	11	1502	73.8	6.18	0.0000	941	1424	64.9	0.99	0.29	689
30	0.4477	0.2974	0.8554	0.1471	0.0958	101.1	1286	70.4	2.11	0.0090	684	0	1195	55.3	0.46	0.96	496	28	1472	56.2	3.72	0.0000	885	1391	48.9	0.78	0.43	634
30	0.4442	0.2999	0.7260	0.1454	0.0964	97.0	1262	79.4	1.59	0.0130	646	17	1178	65.1	0.41	1.14	444	44	1443	72.4	2.53	0.0000	783	1358	56.5	0.63	0.91	597
30	0.4516	0.2962	0.7082	0.1463	0.0946	96.6	1299	86.9	2.16	0.0060	657	0	1204	69.7	0.48	1.05	461	29	1497	73.4	4.28	0.0000	767	1414	62.4	0.82	0.52	602
30	0.4594	0.2973	0.7320	0.1457	0.0948	95.8	1298	92.7	2.50	0.0080	685	0	1207	68.3	0.52	0.97	454	21	1496	85.0	5.32	0.0000	805	1416	75.3	0.94	0.39	593
30	0.4394	0.2962	0.7240	0.1467	0.0981	101.1	1295	67.9	3.23	0.0050	660	0	1207	54.9	0.58	0.90	464	15	1501	65.6	10.31	0.0000	853	1424	47.8	1.13	0.23	620
30	0.4546	0.2970	0.8570	0.1454	0.0946	100.1	1297	81.9	2.00	0.0040	698	19	1208	60.2	0.47	1.05	501	25	1489	67.9	3.81	0.0006	878	1407	50.9	0.79	0.37	651
30	0.4519	0.2972	0.8540	0.1451	0.0947	100.2	1295	75.0	1.90	0.0020	685	0	1203	58.9	0.45	1.06	479	23	1484	65.7	3.30	0.0021	864	1401	48.3	0.74	0.50	625
30	0.4617	0.2968	0.8364	0.1456	0.0945	95.3	1304	103.9	2.37	0.0020	717	0	1213	68.3	0.50	1.01	485	15	1503	103.1	4.56	0.0000	900	1422	79.3	0.89	0.32	689
30	0.4626	0.2982	0.7912	0.1463	0.0963	100.7	1292	94.9	4.37	0.0040	650	0	1207	70.6	0.64	0.84	484	10	1490	74.8	18.12	0.0008	874	1416	56.4	1.38	0.14	707
30	0.4678	0.2998	0.8844	0.1459	0.0971	99.1	1283	94.1	4.07	0.0060	636	0	1206	78.9	0.65	0.76	497	4	1477	73.2	17.02	0.0000	937	1407	72.6	1.40	0.10	763
30	0.4415	0.2990	0.8342	0.1463	0.0975	95.3	1274	85.5	1.62	0.0040	626	0	1185	72.9	0.41	0.95	470	29	1456	79.2	2.50	0.0000	817	1371	60.8	0.65	0.60	618
30	0.4604	0.2971	0.8286	0.1459	0.0952	94.3	1301	107.6	2.30	0.0070	699	0	1212	80.0	0.52	0.95	470	17	1501	115.2	4.77	0.0000	891	1421	73.9	0.92	0.33	649
30	0.4694	0.2997	0.8232	0.1453	0.0949	98.2	1280	110.2	2.80	0.0140	654	0	1199	70.9	0.54	0.88	509	11	1470	86.3	6.80	0.0000	896	1395	65.8	1.03	0.27	704
30	0.4693	0.2992	0.7334	0.1471	0.0984	98.4	1291	125.6	9.72	0.0040	647	0	1215	91.9	0.94	0.65	512	2	1496	93.6	127.41	0.0000	993	1431	106.2	3.02	0.03	772
30	0.4484	0.2980	0.8216	0.1474	0.0988	96.0	1287	85.8	3.22	0.0050	644	0	1204	79.5	0.58	0.76	503	10	1487	91.3	8.88	0.0000	875	1411	73.6	1.12	0.18	692
30	0.4690	0.2996	0.8848	0.1463	0.0984	100.9	1286	79.6	6.61	0.0030	651	0	1212	77.5	0.82	0.73	525	2	1487	68.2	73.37	0.0000	1066	1421	78.8	2.19	0.03	792
30	0.4460	0.2993	0.8480	0.1463	0.0961	95.1	1271	97.1	1.41	0.0060	659	31	1181	64.1	0.39	1.06	499	38	1447	75.3	1.97	0.0000	799	1361	51.5	0.58	0.76	611
30	0.4404	0.2990	0.8610	0.1474	0.0990	100.6	1269	68.4	2.20	0.0050	643	23	1187	57.1	0.50	0.79	470	19	1457	52.6	4.90	0.0000	871	1380	48.6	0.87	0.29	648
30	0.4360	0.2994	0.7316	0.1458	0.0988	96.8	1265	86.8	1.86	0.0060	613	0	1183	65.0	0.45	0.84	476	25	1455	78.7	3.51	0.0007	805	1372	53.2	0.74	0.56	576
30	0.4366	0.2984	0.8162	0.1480	0.0961	101.7	1265	71.7	1.37	0.0060	628	56	1171	51.0	0.38	1.14	456	43	1438	42.7	1.97	0.0007	775	1350	34.9	0.57	1.02	566
30	0.4370	0.2972	0.8820	0.1461	0.0945	101.7	1280	62.3	1.20	0.0090	700	10	1183	45.6	0.35	1.28	436	49	1454	41.6	1.47	0.0020	801	1363	32.6	0.51	1.14	620
30	0.4340	0.2982	0.7522	0.1479	0.0962	94.6	1264	77.6	1.15	0.0070	589	39	1168	61.3	0.34	1.28	437	57	1438	66.9	1.41	0.0007	725	1343	46.8	0.47	1.67	511
30	0.4469	0.2983	0.8530	0.1459	0.0952	94.1	1281	87.1	1.30	0.0050	671	48	1186	69.8	0.38	1.19	482	41	1459	79.7	1.75	0.0015	807	1371	57.4	0.56	0.92	608
30	0.4367	0.2984	0.7150	0.1462	0.0955	100.2	1267	74.9	1.38	0.0110	615	58	1172	54.8	0.37	1.23	426	50	1442	50.7	1.84	0.0006	740	1352	39.6	0.54	1.18	521
30	0.4313	0.2967	0.8022	0.1478	0.0943	96.4	1273	65.7	0.90	0.0170	662	26	1168	53.6	0.31	1.81	396	68	1440	50.9	1.01	0.0051	740	1340	35.8	0.40	2.38	486
30	0.4379	0.2966	0.7128	0.1464	0.0971	94.6	1290	84.1	1.92	0.0080	622	13	1198	63.9	0.46	1.06	444	38	1489	80.5	3.29	0.0000	798	1404	58.5	0.72	0.67	578
30	0.4391	0.2963	0.7682	0.1464	0.0980	99.2	1295	72.7	2.72	0.0040	673	0	1205	56.0	0.53	0.97	460	18	1499	57.0	6.63	0.0000	859	1419	54.7	0.96	0.33	617
30	0.4368	0.2963	0.7444	0.1461	0.0965	97.3	1292	86.6	1.65	0.0050	636	0	1196	60.5	0.43	1.18	455	38	1485	64.0	2.79	0.0079	803	1398	50.0	0.67	0.69	603
30	0.4672	0.2980	0.7512	0.1479	0.0944	94.1	1289	108.8	2.68	0.0040	640	0	1202	86.8	0.53	0.91	458	20	1483	107.0	6.05	0.0000	821	1405	85.9	0.97	0.34	628
30	0.4602	0.2971	0.7994	0.1480	0.0942	99.0	1290	83.8	2.36	0.0030	683	0	1198	65.8	0.50	0.96	474	21	1479	71.9	4.76	0.0000	839	1399	56.6	0.88	0.35	631
30	0.4698	0.2994	0.8384	0.1463	0.0984	96.3	1289	108.1	5.71	0.0070	689	0	1216	102.0	0.81	0.76	523	4	1496	64.9	43.45	0.0000	952	1429	129.9	2.05	0.10	824
30	0.4698	0.2979	0.7908	0.1463	0.0963	99.5	1299	83.5	6.65	0.0050	693	0	1217	78.5	0.76	0.84	498	4	1505	61.8	42.43	0.0000	922	1435	78.1	1.90	0.06	761
30	0.4547	0.2960	0.8866	0.1471	0.0942	96.0	1301	96.6	1.73	0.0050	754	0	1213	67.3	0.45	1.08	480	24	1494	75.8	2.85	0.0000	896	1410	60.5	0.73	0.54	640
30	0.4528	0.2994	0.7982	0.1477	0.0986	99.2	1272	84.1	3.39	0.0030	650	13	1194	68.7	0.61	0.74	492	10	1466	81.4	13.10	0.0000	842	1393	61.8	1.26	0.17	656
30	0.4447	0.2978	0.7382	0.1454	0.0980	98.9	1289	88.6	3.12	0.0060	627	0	1202	65.4	0.55	0.85	507	17	1489	85.7	8.10	0.0000	868	1411	60.8	1.04	0.25	660
30	0.4532	0.2978	0.8314	0.1467	0.0976	96.3	1291	98.5	2.90	0.0070	641	0	1206	73.6	0.57	0.89	484	11	1491	87.1	7.49	0.0000	885	1415	66.3	1.09	0.22	732
30	0.4633	0.2986	0.8190	0.1465	0.0971	99.7	1290	87.0	4.82	0.0050	650	0	1207	73.3	0.67	0.84	514	4	1487	87.6	19.39	0.0000	907	1416	77.5	1.47	0.08	742
30	0.4683	0.2967	0.7646	0.1468	0.0961	100.4	1306	97.7	7.63	0.0030	703	0	1226	69.8	0.83	0.86	495	3	1516	84.3	62.00	0.0000	890	1447	75.4	2.07	0.07	702
30	0.4699	0.2977	0.7356	0.1474	0.0985	94.2	1309	86.9	10.81	0.0040	686	0	1230	115.4	1.03	0.79	482	2	1527	58.4	139.70	0.0000	918	1461	139.2	3.20	0.03	808
30	0.4411	0.2981	0.7998	0.1456	0.0986	101.2	1282	72.2	2.85	0.0050	635	0	1198	53.7	0.54	0.89	457	18	1480	67.5	6.88	0.0005	882	1402	48.7	0.98	0.21	660
30	0.4428	0.2980	0.7452	0.1471	0.0988	94.2	1282	98.3	2.46	0.0040	620	0	1199	73.2	0.53	0.80	504	18	1484	98.7	5.84	0.0000	878	1404	65.8	0.95	0.31	650

Continuation of Table S2.

c_2	q_H	σ_{OO}	ϵ_{OO}	d_{OO}	d_{OH}	a_{HOO}	ρ	ϵ	μ	P_v	λ	wt$\%_v$	ρ	ϵ	μ	P_v	λ	wt$\%_v$	ρ	ϵ	μ	P_v	λ	ρ	ϵ	μ	P_v	λ
30	0.4566	0.2986	0.8734	0.1465	0.0945	95.3	1282	91.9	1.54	0.0100	656	0	1189	68.5	0.41	1.06	454	34	1460	82.9	2.34	0.0000	852	1376	61.5	0.65	0.60	629
30	0.4498	0.2988	0.8190	0.1459	0.0944	100.8	1273	67.6	1.52	0.0080	664	0	1180	52.2	0.39	1.17	450	42	1448	60.8	2.24	0.0004	795	1362	42.7	0.60	0.69	622
30	0.4395	0.2978	0.7064	0.1470	0.0951	96.5	1274	76.3	1.31	0.0040	632	51	1175	64.0	0.37	1.32	407	55	1450	63.5	1.76	0.0016	713	1359	50.5	0.54	1.46	468
30	0.4338	0.2978	0.7512	0.1467	0.0943	100.9	1265	62.2	1.05	0.0150	623	53	1164	47.6	0.33	1.55	388	58	1433	43.0	1.24	0.0017	714	1337	30.4	0.44	1.89	451
30	0.4326	0.2962	0.7062	0.1475	0.0961	97.4	1285	73.7	1.50	0.0070	602	8	1185	55.5	0.39	1.19	422	48	1471	55.0	2.20	0.0000	744	1380	44.2	0.59	1.14	531
31.5	0.4665	0.2969	0.7390	0.1479	0.0967	95.8	1303	107.6	6.17	0.0090	626	0	1222	86.6	0.79	0.84	498	7	1513	99.2	38.73	0.0000	915	1445	89.3	1.92	0.09	727
31.5	0.4648	0.2988	0.7172	0.1473	0.0969	95.3	1286	122.4	4.36	0.0040	662	0	1207	86.5	0.67	0.88	491	10	1488	131.2	19.88	0.0000	905	1416	84.8	1.50	0.13	670
31.5	0.4490	0.2986	0.8886	0.1477	0.0974	94.4	1278	95.0	1.87	0.0030	654	0	1192	64.0	0.46	0.90	499	20	1465	74.7	3.34	0.0000	867	1384	65.5	0.75	0.38	652
31.5	0.4591	0.2993	0.7822	0.1472	0.0961	95.4	1274	82.2	2.33	0.0040	655	0	1191	75.6	0.50	0.79	485	20	1462	79.2	5.09	0.0000	840	1384	65.4	0.89	0.30	620
31.5	0.4610	0.2975	0.7728	0.1470	0.0949	99.0	1287	75.8	2.74	0.0120	674	6	1201	65.8	0.53	0.93	489	16	1483	65.4	6.64	0.0008	828	1406	58.4	1.01	0.29	648
31.5	0.4568	0.2971	0.8702	0.1457	0.0982	94.4	1307	99.3	4.02	0.0060	713	0	1222	82.8	0.64	0.89	513	8	1516	118.6	12.12	0.0000	960	1443	88.7	1.33	0.13	756
31.5	0.4408	0.2975	0.7050	0.1455	0.0953	99.0	1280	68.7	1.55	0.0080	605	9	1184	53.9	0.41	1.18	427	47	1464	53.0	2.37	0.0000	787	1376	43.3	0.61	1.03	543
31.5	0.4602	0.2968	0.8804	0.1468	0.0978	100.4	1304	81.0	5.97	0.0040	680	0	1223	69.4	0.74	0.80	517	2	1513	62.1	31.74	0.0000	1004	1443	60.0	1.75	0.08	821
31.5	0.4462	0.2965	0.8244	0.1476	0.0960	94.9	1293	79.6	1.83	0.0040	683	12	1199	61.5	0.44	1.05	462	30	1484	72.5	2.85	0.0000	852	1400	51.9	0.71	0.57	662
31.5	0.4460	0.2990	0.7456	0.1465	0.0948	97.9	1265	71.3	1.33	0.0070	601	10	1172	54.6	0.37	1.20	461	48	1440	58.9	1.94	0.0007	749	1351	43.6	0.56	1.17	538
31.5	0.4577	0.2990	0.8684	0.1479	0.0963	96.3	1277	89.9	2.17	0.0070	666	0	1191	65.1	0.49	0.90	495	20	1461	81.0	4.33	0.0000	886	1383	67.4	0.86	0.29	671
31.5	0.4623	0.2968	0.7678	0.1461	0.0955	99.2	1302	69.5	4.05	0.0050	678	0	1215	61.3	0.63	0.90	457	11	1507	79.8	14.28	0.0000	859	1431	60.0	1.30	0.20	692
33	0.4644	0.2996	0.7306	0.1451	0.0965	102.0	1279	75.5	4.37	0.0040	651	0	1200	59.0	0.65	0.81	486	7	1476	63.0	24.12	0.0000	872	1404	54.4	1.54	0.16	692
33	0.4647	0.2980	0.7516	0.1470	0.0975	94.9	1294	91.5	4.82	0.0050	624	0	1214	84.3	0.74	0.83	492	4	1504	68.8	24.24	0.0000	877	1432	81.3	1.67	0.11	717
33	0.4313	0.2975	0.8920	0.1462	0.0940	100.1	1270	51.7	0.88	0.0090	658	55	1169	45.9	0.30	1.71	411	62	1433	35.3	0.91	0.0097	781	1335	28.2	0.40	2.12	516
33	0.4360	0.2986	0.8862	0.1455	0.0946	99.8	1266	60.6	0.97	0.0110	654	69	1168	42.5	0.32	1.48	422	57	1431	39.4	1.07	0.0004	785	1337	31.5	0.44	1.77	540
33	0.4552	0.2992	0.7436	0.1459	0.0956	100.2	1269	59.8	2.29	0.0050	623	0	1186	54.7	0.48	0.88	445	24	1458	53.4	4.85	0.0000	804	1379	46.6	0.83	0.39	592
33	0.4549	0.2967	0.8486	0.1455	0.0978	94.9	1302	80.2	3.51	0.0130	673	0	1220	73.3	0.62	0.83	483	8	1517	82.9	9.73	0.0000	932	1442	69.7	1.21	0.16	695
33	0.4363	0.2997	0.7834	0.1471	0.0970	99.0	1251	43.3	1.28	0.0080	583	49	1166	54.1	0.37	1.09	471	45	1427	51.5	1.88	0.0040	747	1338	38.4	0.54	1.11	586
33	0.4454	0.2996	0.8508	0.1461	0.0987	96.0	1271	75.9	2.04	0.0010	631	0	1190	62.8	0.47	0.82	473	23	1459	76.0	4.02	0.0000	831	1380	53.6	0.81	0.36	632
33	0.4305	0.2990	0.7208	0.1461	0.0974	100.5	1259	60.3	1.35	0.0030	594	50	1169	46.5	0.38	1.16	437	52	1436	37.7	2.01	0.0013	730	1347	31.0	0.55	1.21	496
33	0.4549	0.2961	0.8114	0.1460	0.0964	94.6	1308	99.1	2.90	0.0070	695	0	1217	70.6	0.56	0.98	491	19	1515	97.0	6.41	0.0000	863	1436	70.5	1.01	0.24	698
33	0.4418	0.2969	0.8334	0.1474	0.0958	97.7	1285	69.0	1.47	0.0040	627	23	1189	55.5	0.41	1.15	467	38	1468	56.8	2.24	0.0003	820	1381	42.7	0.62	0.77	613
33	0.4350	0.2985	0.7752	0.1477	0.0968	100.3	1263	63.9	1.39	0.0100	619	0	1171	53.2	0.38	1.08	431	41	1439	41.1	2.13	0.0004	758	1350	33.7	0.57	0.94	545
33	0.4316	0.2961	0.8378	0.1477	0.0956	100.1	1282	62.5	1.22	0.0070	663	0	1181	45.9	0.36	1.30	450	49	1461	40.6	1.53	0.0013	787	1370	28.2	0.52	1.22	511
33	0.4322	0.2967	0.8556	0.1466	0.0949	98.4	1280	67.3	1.02	0.0110	678	16	1179	47.0	0.33	1.56	438	57	1452	42.2	1.19	0.0023	787	1356	31.4	0.45	1.68	539
33	0.4637	0.2982	0.7498	0.1466	0.0967	98.1	1288	89.9	4.59	0.0070	637	0	1208	69.9	0.67	0.84	452	9	1494	60.0	19.30	0.0000	832	1421	64.0	1.50	0.17	719
33	0.4598	0.2968	0.7688	0.1475	0.0965	99.8	1297	87.4	4.59	0.0020	691	0	1211	63.0	0.66	0.85	477	12	1502	80.6	18.91	0.0000	889	1428	56.8	1.47	0.11	672
33	0.4381	0.2962	0.7828	0.1478	0.0964	98.3	1288	75.5	1.76	0.0040	753	0	1191	55.8	0.43	1.08	446	36	1477	50.7	2.87	0.0007	818	1390	38.5	0.66	0.74	595
33	0.4575	0.2965	0.7366	0.1467	0.0948	95.0	1301	88.4	2.49	0.0070	682	0	1207	69.9	0.51	0.97	441	26	1498	69.0	4.97	0.0000	820	1417	64.9	0.87	0.48	596
33	0.4325	0.2978	0.7502	0.1456	0.0985	94.3	1277	75.4	1.64	0.0060	625	0	1188	56.2	0.42	1.10	459	38	1471	70.7	2.52	0.0006	794	1383	52.6	0.64	0.81	580
33	0.4415	0.2996	0.7024	0.1455	0.0987	95.5	1268	73.7	2.15	0.0060	621	12	1187	67.0	0.48	0.91	442	26	1461	76.2	4.38	0.0000	746	1380	51.0	0.83	0.52	576
33	0.4516	0.2989	0.8196	0.1476	0.0986	100.3	1275	76.9	3.48	0.0030	652	0	1195	60.4	0.60	0.77	491	11	1471	70.3	13.15	0.0000	870	1397	46.3	1.24	0.16	672
33	0.4496	0.2991	0.7764	0.1479	0.0976	94.2	1269	83.4	2.11	0.0040	675	0	1187	66.8	0.48	0.96	477	28	1458	85.4	4.00	0.0003	843	1379	56.1	0.83	0.47	626
33	0.4327	0.2963	0.7058	0.1472	0.0968	97.1	1286	70.4	1.69	0.0100	616	12	1188	51.0	0.41	1.23	417	39	1475	54.0	2.45	0.0000	750	1387	41.2	0.63	1.03	502
33	0.4615	0.2961	0.7512	0.1472	0.0987	94.6	1316	95.3	8.84	0.0080	681	0	1234	88.3	0.94	0.86	489	4	1539	77.7	95.19	0.0000	956	1470	85.0	2.54	0.05	743
33	0.4698	0.2965	0.7424	0.1472	0.0974	99.6	1311	56.4	14.18	0.0050	703	0	1231	68.4	1.07	0.86	493	2	1531	54.5	217.95	0.0000	985	1463	75.5	3.47	0.03	760
33	0.4487	0.2978	0.8724	0.1463	0.0943	98.5	1280	74.9	1.34	0.0040	676	18	1185	51.0	0.38	1.31	454	41	1457	52.6	1.82	0.0006	797	1370	41.2	0.56	0.96	578
33	0.4508	0.2982	0.7526	0.1458	0.0956	94.6	1280	81.4	1.77	0.0100	626	27	1190	65.8	0.43	1.10	466	38	1470	79.1	2.85	0.0000	799	1385	53.7	0.69	0.72	559
33	0.4516	0.2971	0.8358	0.1454	0.0959	97.7	1296	74.2	2.29	0.0110	723	0	1204	55.4	0.48	1.01	458	22	1492	62.4	4.30	0.0000	879	1410	44.3	0.83	0.35	657
33	0.4523	0.2987	0.7540	0.1474	0.0948	100.4	1270	72.4	1.83	0.0040	650	53	1179	52.2	0.43	1.01	444	33	1448	54.0	3.18	0.0000	786	1365	37.9	0.70	0.71	577
33	0.4542	0.2980	0.7960	0.1459	0.0975	99.8	1288	93.2	3.60	0.0050	664	17	1205	56.1	0.60	0.82	453	13	1490	46.9	11.74	0.0000	907	1415	48.9	1.23	0.18	684
33	0.4536	0.2977	0.8904	0.1477	0.0982	94.5	1292	94.1	2.91	0.0070	629	0	1207	66.1	0.57	0.82	492	11	1490	83.0	7.30	0.0000	943	1414	63.3	1.06	0.19	706
33	0.4652	0.2975	0.8710	0.1463	0.0965	95.4	1301	102.5	3.49	0.0090	703	0	1216	79.0	0.62	0.87	500	7	1504	101.7	11.06	0.0000	920	1429	72.6	1.24	0.13	754

Continuation of Table S2.

c_2	q_H	σ_{OO}	ϵ_{OO}	d_{OO}	d_{OH}	a_{HOO}	ρ	ϵ	μ	P_v	λ	wt%$_v$	ρ	ϵ	μ	P_v	λ	wt%$_v$	ρ	ϵ	μ	P_v	λ	ρ	ϵ	μ	P_v	λ
33	0.4618	0.2965	0.8186	0.1454	0.0985	100.0	1312	98.1	10.18	0.0060	719	0	1231	74.1	0.91	0.82	505	3	1533	64.4	99.09	0.0000	1031	1465	75.6	2.60	0.04	786
33	0.4407	0.2995	0.7558	0.1455	0.0966	98.0	1261	68.6	1.42	0.0020	595	0	1174	51.9	0.39	1.16	445	41	1441	53.6	2.13	0.0000	742	1354	40.1	0.58	0.98	539
33	0.4657	0.2966	0.8866	0.1469	0.0979	95.7	1314	80.1	6.73	0.0040	705	0	1229	76.0	0.79	0.87	499	5	1526	86.7	38.65	0.0000	1047	1456	88.1	1.87	0.06	785
33	0.4562	0.2998	0.7736	0.1466	0.0975	97.3	1271	79.2	2.66	0.0040	640	0	1192	62.8	0.55	0.80	486	17	1462	71.3	7.72	0.0000	854	1386	58.4	1.04	0.26	675
33	0.4629	0.2988	0.8204	0.1458	0.0945	97.5	1284	77.9	2.04	0.0080	655	4	1193	64.1	0.46	0.94	465	23	1468	73.6	4.01	0.0000	845	1387	56.7	0.81	0.37	637
33	0.4500	0.2962	0.8898	0.1464	0.0950	98.0	1300	68.3	1.80	0.0110	737	0	1209	59.5	0.45	1.07	450	25	1491	54.1	2.86	0.0003	886	1407	41.4	0.72	0.46	629
33	0.4515	0.2964	0.7564	0.1464	0.0961	96.4	1301	87.0	2.73	0.0040	682	0	1208	65.6	0.53	0.93	472	19	1502	70.7	5.97	0.0000	844	1421	55.8	0.94	0.39	650
33	0.4435	0.2974	0.7618	0.1479	0.0972	97.3	1282	78.2	2.20	0.0060	613	0	1191	55.1	0.48	1.07	464	33	1473	56.8	4.15	0.0000	803	1391	49.9	0.80	0.43	584
33	0.4506	0.2989	0.7130	0.1456	0.0985	96.3	1281	95.3	3.32	0.0050	640	0	1202	65.8	0.61	0.81	482	18	1485	62.0	12.00	0.0000	854	1409	60.1	1.21	0.32	647
33	0.4576	0.2985	0.7204	0.1463	0.0986	100.3	1287	70.2	6.32	0.0030	642	0	1209	64.5	0.79	0.82	497	5	1495	61.8	64.26	0.0000	872	1425	63.8	2.04	0.07	722
33	0.4504	0.2978	0.7254	0.1460	0.0973	99.1	1287	74.1	3.21	0.0060	634	0	1202	60.6	0.58	0.87	457	22	1488	63.4	9.93	0.0000	828	1411	50.6	1.12	0.21	633
33	0.4498	0.2995	0.8388	0.1455	0.0987	98.5	1275	84.2	2.84	0.0030	642	0	1196	59.8	0.54	0.73	489	11	1469	74.3	7.13	0.0000	912	1393	54.4	1.03	0.22	684
33	0.4617	0.2984	0.7440	0.1472	0.0947	101.8	1280	76.6	2.99	0.0020	676	0	1192	57.2	0.53	0.93	461	18	1467	52.4	7.65	0.0000	847	1389	45.5	1.02	0.27	627
33	0.4560	0.2978	0.8974	0.1474	0.0966	96.9	1288	82.8	2.47	0.0040	670	0	1201	60.1	0.51	0.91	460	19	1480	79.5	5.19	0.0000	894	1402	58.5	0.93	0.25	731
33	0.4610	0.2978	0.8348	0.1476	0.0965	96.9	1291	87.6	3.23	0.0100	657	0	1204	66.9	0.58	0.90	483	14	1486	66.1	8.79	0.0000	933	1410	62.2	1.10	0.19	695
33	0.4346	0.2999	0.8538	0.1456	0.0983	101.5	1260	58.1	1.52	0.0030	611	0	1176	48.8	0.41	0.97	471	33	1439	41.8	2.43	0.0005	855	1354	33.1	0.64	0.62	650
33	0.4368	0.2998	0.7764	0.1461	0.0949	101.8	1251	55.7	1.04	0.0060	591	59	1157	45.4	0.33	1.50	424	60	1413	37.7	1.26	0.0013	732	1319	27.6	0.45	1.78	450
33	0.4409	0.2974	0.7864	0.1470	0.0986	97.5	1286	66.8	2.51	0.0040	653	0	1198	56.7	0.52	0.94	491	21	1484	61.2	5.80	0.0000	866	1404	50.3	0.93	0.38	638
33	0.4525	0.2990	0.8710	0.1469	0.0954	101.5	1273	60.0	1.86	0.0110	649	0	1183	49.0	0.44	1.03	489	26	1450	46.5	3.21	0.0000	859	1369	34.6	0.72	0.48	614
33	0.4503	0.2974	0.7812	0.1476	0.0976	101.4	1285	63.9	3.62	0.0050	662	0	1201	56.5	0.59	0.95	478	15	1486	61.2	12.28	0.0000	908	1410	44.6	1.22	0.20	716
33	0.4426	0.2960	0.8338	0.1459	0.0984	95.2	1305	92.7	2.59	0.0070	702	0	1215	61.6	0.55	0.93	465	15	1512	67.2	5.92	0.0000	878	1432	59.8	0.96	0.27	690
33	0.4672	0.2977	0.7778	0.1451	0.0975	101.3	1302	62.7	9.69	0.0030	693	0	1222	66.2	0.88	0.82	516	2	1517	53.2	121.61	0.0000	1005	1447	61.4	2.48	0.05	810
33	0.4474	0.2988	0.8360	0.1479	0.0961	99.9	1266	67.0	1.69	0.0060	627	0	1179	53.2	0.42	0.94	443	31	1446	45.6	2.88	0.0000	817	1362	41.8	0.68	0.62	621
33	0.4579	0.2972	0.8538	0.1480	0.0963	101.6	1288	82.7	3.40	0.0110	654	0	1204	56.0	0.58	0.96	456	14	1487	54.1	10.06	0.0000	896	1411	45.2	1.16	0.12	708
33	0.4479	0.2960	0.8824	0.1473	0.0984	98.6	1302	80.8	3.64	0.0060	680	0	1216	66.2	0.61	0.86	485	9	1511	67.1	10.10	0.0000	927	1436	52.9	1.22	0.11	695
33	0.4624	0.2967	0.7128	0.1457	0.0966	99.9	1305	48.6	6.80	0.0030	694	0	1223	68.2	0.79	0.92	525	7	1521	47.1	47.80	0.0000	927	1450	61.3	1.94	0.10	733
33	0.4483	0.2968	0.7732	0.1456	0.0945	98.3	1282	42.3	1.64	0.0110	673	63	1194	53.2	0.41	1.27	441	40	1480	52.3	2.63	0.0011	829	1392	45.3	0.65	0.81	597
33	0.4622	0.2994	0.7366	0.1457	0.0947	97.7	1271	85.4	2.23	0.0050	626	0	1189	60.2	0.48	1.03	476	25	1461	76.0	4.71	0.0013	823	1382	58.3	0.84	0.39	627
33	0.4360	0.2999	0.7612	0.1453	0.0941	96.0	1249	64.1	0.80	0.0110	586	64	1150	50.9	0.29	2.05	384	73	1404	49.1	0.83	0.0119	685	1301	32.4	0.35	3.59	449
33	0.4616	0.2965	0.8482	0.1451	0.0957	96.3	1310	99.8	3.25	0.0040	722	0	1222	73.7	0.58	0.98	476	11	1516	72.3	7.92	0.0000	899	1439	65.9	1.10	0.23	703
33	0.4383	0.2993	0.7430	0.1461	0.0948	100.9	1257	57.3	1.10	0.0020	596	86	1161	41.8	0.34	1.36	448	58	1423	39.6	1.40	0.0006	726	1329	30.8	0.48	1.67	489
34.5	0.4432	0.2973	0.8836	0.1455	0.0942	101.3	1283	65.0	1.25	0.0060	716	0	1187	47.5	0.37	1.24	436	40	1460	38.4	1.65	0.0014	837	1372	29.5	0.54	0.99	577
34.5	0.4694	0.2963	0.7486	0.1463	0.0956	100.7	1312	68.4	8.48	0.0070	739	0	1225	60.5	0.80	0.96	508	6	1522	51.8	65.21	0.0000	988	1452	55.7	1.99	0.08	731
34.5	0.4461	0.2968	0.7018	0.1461	0.0954	98.6	1288	65.2	2.10	0.0040	626	0	1194	55.6	0.45	1.08	445	36	1482	52.0	3.72	0.0017	764	1396	41.5	0.74	0.71	524
34.5	0.4387	0.2978	0.7842	0.1463	0.0968	101.3	1275	61.9	1.69	0.0100	605	64	1184	44.1	0.43	1.04	456	36	1462	41.8	2.98	0.0002	803	1377	37.9	0.67	0.70	551
34.5	0.4460	0.2975	0.8602	0.1478	0.0950	99.1	1279	69.7	1.47	0.0090	680	9	1183	50.2	0.39	1.14	464	38	1456	46.4	2.14	0.0000	846	1369	37.3	0.60	0.72	626
34.5	0.4356	0.2984	0.7532	0.1452	0.0948	96.8	1265	68.4	1.00	0.0140	602	73	1166	48.0	0.32	1.73	426	67	1435	43.9	1.16	0.0045	693	1337	34.8	0.43	2.34	448
34.5	0.4343	0.2988	0.7766	0.1472	0.0944	99.8	1254	48.6	0.93	0.0070	604	32	1155	43.8	0.31	1.82	437	70	1413	35.4	1.04	0.0033	676	1314	26.8	0.40	2.54	492
34.5	0.4454	0.2960	0.7902	0.1478	0.0987	94.5	1294	73.6	3.53	0.0050	632	0	1212	71.9	0.58	0.92	500	14	1509	80.0	8.05	0.0000	919	1432	61.5	1.12	0.27	686
34.5	0.4466	0.2976	0.8782	0.1475	0.0941	96.8	1275	64.6	1.17	0.0090	674	0	1180	55.1	0.35	1.23	414	46	1450	50.2	1.49	0.0007	811	1359	36.3	0.51	1.16	624
34.5	0.4401	0.2964	0.7632	0.1479	0.0965	99.1	1281	55.3	1.94	0.0080	640	11	1190	52.4	0.44	0.92	436	33	1477	45.9	3.38	0.0000	797	1392	41.5	0.73	0.65	590
34.5	0.4620	0.2994	0.7008	0.1453	0.0951	101.2	1272	69.0	2.79	0.0010	630	0	1192	53.2	0.54	0.85	475	17	1465	55.5	8.57	0.0000	839	1388	43.3	1.05	0.35	616
34.5	0.4590	0.2965	0.8652	0.1476	0.0945	99.7	1293	65.6	2.38	0.0100	705	0	1205	56.9	0.50	1.06	512	23	1489	62.9	4.64	0.0000	865	1408	46.9	0.89	0.29	659
34.5	0.4375	0.2967	0.7086	0.1478	0.0961	98.0	1275	59.3	1.65	0.0110	644	54	1182	54.3	0.41	1.31	463	48	1466	43.7	2.59	0.0016	789	1378	39.2	0.64	1.07	569
34.5	0.4551	0.2995	0.7916	0.1459	0.0973	94.7	1274	85.3	2.32	0.0070	632	0	1193	74.4	0.50	0.80	455	19	1467	61.6	5.06	0.0000	849	1388	60.6	0.89	0.35	628
34.5	0.4531	0.2962	0.7890	0.1475	0.0947	99.0	1293	68.1	2.12	0.0070	697	25	1200	49.1	0.47	0.91	474	24	1487	52.2	3.99	0.0000	835	1404	40.1	0.81	0.46	631
34.5	0.4526	0.2997	0.8622	0.1466	0.0945	94.7	1263	67.7	1.16	0.0070	631	0	1172	60.6	0.35	1.12	463	51	1432	65.5	1.54	0.0014	801	1343	44.8	0.51	1.22	588
34.5	0.4671	0.2986	0.7622	0.1459	0.0960	98.9	1286	67.1	4.14	0.0090	670	0	1205	69.1	0.64	0.78	451	5	1487	81.2	16.46	0.0000	874	1414	58.9	1.38	0.15	701
34.5	0.4500	0.2986	0.7708	0.1478	0.0957	100.8	1270	56.9	1.88	0.0040	638	0	1180	50.4	0.45	1.07	504	31	1451	43.2	3.74	0.0017	776	1369	38.7	0.74	0.62	577
34.5	0.4391	0.3000	0.7154	0.1470	0.0950	100.2	1244	61.8	1.10	0.0030	593	44	1156	42.6	0.33	1.60	442	60	1410	42.2	1.45	0.0005	704	1316	25.9	0.46	1.93	443
34.5	0.4379	0.2991	0.8756	0.1458	0.0957	99.2	1266	57.1	1.11	0.0030	629	23	1171	50.8	0.35	1.20	443	52	1434	46.6	1.38	0.0007	800	1342	31.4	0.49	1.17	574

Continuation of Table S2.

c_2	q_H	σ_{OO}	ϵ_{OO}	d_{OO}	d_{OH}	a_{HOO}	ρ	ϵ	μ	P_v	λ	wt%$_v$	ρ	ϵ	μ	P_v	λ	wt%$_v$	ρ	ϵ	μ	P_v	λ	ρ	ϵ	μ	P_v	λ
34.5	0.4449	0.2984	0.8768	0.1460	0.0955	95.2	1277	70.5	1.28	0.0040	704	12	1182	53.7	0.37	1.31	439	46	1452	59.7	1.63	0.0014	813	1363	42.4	0.54	0.98	600
34.5	0.4414	0.2971	0.8530	0.1477	0.0955	94.3	1278	65.7	1.25	0.0050	672	52	1183	54.7	0.37	1.26	465	43	1459	57.4	1.58	0.0021	838	1367	38.4	0.52	1.23	540
34.5	0.4501	0.2977	0.7756	0.1464	0.0961	99.1	1279	74.5	2.25	0.0030	652	14	1193	48.6	0.47	0.93	393	40	1475	58.3	4.33	0.0000	840	1393	45.1	0.83	0.42	592
34.5	0.4366	0.2966	0.8078	0.1455	0.0955	99.1	1279	97.4	1.27	0.0090	672	54	1187	46.5	0.38	1.26	515	52	1471	42.3	1.81	0.0011	790	1380	33.2	0.55	1.08	573
34.5	0.4561	0.2968	0.8456	0.1477	0.0969	100.4	1292	68.9	3.35	0.0050	708	0	1208	56.1	0.61	0.42	581	32	1496	52.5	11.68	0.0000	907	1421	42.0	1.23	0.15	732
36	0.4619	0.2991	0.8906	0.1462	0.0963	99.9	1279	71.5	2.71	0.0040	654	0	1197	56.2	0.54	0.89	496	9	1469	56.3	6.82	0.0000	920	1394	47.3	1.02	0.20	698
36	0.4506	0.2969	0.7944	0.1458	0.0983	97.3	1300	72.9	3.71	0.0060	666	0	1214	58.5	0.61	0.95	527	10	1509	60.9	11.73	0.0000	867	1433	52.7	1.28	0.15	692
36	0.4479	0.2976	0.7720	0.1478	0.0956	99.2	1278	68.4	1.86	0.0020	642	0	1185	54.5	0.44	0.98	497	37	1462	46.1	3.19	0.0002	834	1378	38.6	0.71	0.65	520
36	0.4398	0.2995	0.7442	0.1478	0.0942	95.5	1247	62.2	0.91	0.0080	596	69	1150	46.9	0.30	1.88	392	71	1404	46.5	0.97	0.0060	690	1304	29.2	0.38	3.14	458
36	0.4627	0.2999	0.7902	0.1476	0.0982	99.0	1272	66.6	4.47	0.0040	627	0	1197	60.8	0.68	0.71	490	6	1466	51.0	23.08	0.0000	932	1396	56.5	1.52	0.18	714
36	0.4461	0.2972	0.7252	0.1479	0.0984	98.2	1284	77.7	3.54	0.0070	650	0	1200	57.4	0.60	0.85	487	21	1489	62.9	12.17	0.0000	852	1412	50.6	1.20	0.24	620
36	0.4648	0.2967	0.7610	0.1459	0.0955	101.5	1304	75.2	5.78	0.0030	683	0	1218	56.8	0.69	0.86	468	8	1511	44.4	23.33	0.0000	875	1437	49.8	1.51	0.12	688
36	0.4504	0.2992	0.8688	0.1472	0.0958	94.2	1270	71.8	1.40	0.0070	673	25	1179	61.0	0.39	1.06	471	39	1444	59.7	1.94	0.0000	824	1357	46.6	0.59	0.89	605
36	0.4631	0.2987	0.8946	0.1475	0.0958	101.8	1282	71.6	2.97	0.0030	705	0	1196	53.0	0.54	0.94	489	14	1468	46.1	8.20	0.0000	926	1393	43.6	1.04	0.15	704
36	0.4638	0.2985	0.7358	0.1456	0.0964	101.2	1286	72.4	4.93	0.0040	654	0	1205	56.9	0.67	0.81	468	8	1489	57.1	22.48	0.0000	872	1416	48.0	1.51	0.17	640
36	0.4577	0.2964	0.8746	0.1455	0.0960	100.4	1306	72.2	3.12	0.0080	736	0	1220	57.4	0.56	1.04	508	10	1509	51.4	8.44	0.0000	921	1432	46.0	1.11	0.19	707
36	0.4463	0.2967	0.8054	0.1463	0.0977	99.5	1295	62.7	2.88	0.0020	657	0	1206	51.6	0.55	0.86	467	15	1498	60.9	7.81	0.0000	891	1419	45.9	1.04	0.25	658
36	0.4420	0.2982	0.7602	0.1479	0.0970	94.7	1269	83.6	1.62	0.0020	623	0	1179	55.1	0.41	1.09	459	39	1453	55.5	2.49	0.0000	775	1367	45.6	0.64	0.93	523
36	0.4605	0.2969	0.8822	0.1474	0.0985	98.3	1301	78.6	5.86	0.0040	697	0	1220	59.8	0.76	0.97	466	4	1512	48.2	35.32	0.0000	999	1441	62.3	1.77	0.08	800
36	0.4490	0.2965	0.7276	0.1468	0.0960	101.0	1290	54.9	2.79	0.0050	648	17	1200	49.6	0.52	1.05	471	19	1491	46.7	6.57	0.0016	839	1411	35.8	0.98	0.36	614
36	0.4341	0.2986	0.7908	0.1477	0.0971	101.4	1261	50.5	1.50	0.0040	601	20	1170	44.6	0.37	1.18	502	46	1437	37.5	2.02	0.0004	767	1349	28.9	0.58	0.96	525
36	0.4320	0.2996	0.8320	0.1470	0.0960	101.6	1251	49.3	1.04	0.0010	604	0	1158	39.8	0.33	1.36	434	55	1413	32.4	1.20	0.0024	762	1319	25.3	0.45	1.64	531
36	0.4620	0.2970	0.8890	0.1473	0.0977	95.2	1300	78.4	3.94	0.0030	685	0	1218	68.9	0.66	0.86	496	8	1508	79.9	14.55	0.0000	959	1436	66.0	1.39	0.11	812
36	0.4388	0.2997	0.7922	0.1471	0.0962	101.2	1252	58.3	1.25	0.0070	605	0	1164	40.6	0.37	1.14	425	48	1423	36.4	1.79	0.0010	740	1334	30.4	0.54	1.16	522
36	0.4438	0.2978	0.7504	0.1471	0.0973	99.2	1274	66.7	2.33	0.0070	619	10	1190	49.9	0.49	0.94	449	26	1470	48.0	4.70	0.0000	815	1389	40.3	0.84	0.55	625
36	0.4694	0.2969	0.8530	0.1471	0.0972	96.0	1306	55.8	5.97	0.0020	685	0	1224	70.7	0.78	0.82	530	4	1518	103.1	30.39	0.0000	1003	1448	68.4	1.84	0.07	743
36	0.4507	0.2988	0.8912	0.1466	0.0984	100.2	1277	60.0	2.66	0.0060	647	0	1197	55.0	0.55	0.86	491	11	1473	50.6	7.77	0.0000	910	1397	41.4	1.05	0.23	727
36	0.4617	0.2969	0.7824	0.1477	0.0940	99.9	1282	7.1	2.06	0.0060	680	7	1198	53.0	0.50	0.95	457	25	1482	49.2	5.55	0.0000	857	1401	42.7	0.89	0.42	642
36	0.4635	0.2998	0.7980	0.1474	0.0948	97.0	1266	78.1	2.01	0.0040	666	18	1181	58.9	0.45	0.92	468	29	1445	62.7	3.68	0.0000	839	1364	47.3	0.76	0.48	628
36	0.4390	0.2970	0.8208	0.1466	0.0979	94.7	1287	72.9	1.85	0.0060	688	0	1194	55.8	0.44	1.02	439	31	1481	60.2	2.93	0.0000	835	1396	49.2	0.70	0.55	635
36	0.4585	0.2972	0.8688	0.1472	0.0962	100.1	1290	70.2	3.08	0.0040	669	0	1207	52.4	0.55	0.86	491	14	1490	54.0	7.92	0.0000	888	1414	46.8	1.09	0.19	691
36	0.4566	0.2990	0.7310	0.1459	0.0984	101.7	1281	67.1	5.81	0.0020	653	0	1203	53.7	0.71	0.73	534	5	1484	56.9	46.29	0.0000	841	1412	51.5	1.69	0.09	704
36	0.4390	0.2992	0.8314	0.1455	0.0965	98.9	1262	51.5	1.28	0.0100	650	45	1174	47.5	0.38	1.25	424	45	1440	43.4	1.77	0.0028	754	1351	35.1	0.54	1.02	585
36	0.4302	0.2982	0.8832	0.1463	0.0969	98.4	1266	44.9	1.16	0.0070	651	28	1173	44.0	0.35	1.23	354	48	1441	36.5	1.36	0.0002	776	1349	30.3	0.49	1.33	583
36	0.4600	0.2995	0.7430	0.1461	0.0989	95.9	1281	82.5	4.49	0.0030	658	0	1206	72.1	0.72	0.73	530	11	1484	75.0	24.63	0.0000	881	1415	68.1	1.70	0.11	677
36	0.4600	0.2962	0.7764	0.1467	0.0985	94.2	1311	106.8	6.44	0.0030	662	0	1229	77.1	0.82	0.86	484	4	1532	92.0	36.68	0.0000	955	1461	79.3	1.95	0.09	740
36	0.4638	0.2983	0.7474	0.1469	0.0971	98.5	1287	67.9	4.82	0.0040	668	0	1207	53.4	0.71	0.77	503	9	1493	89.2	31.23	0.0000	927	1420	52.8	1.65	0.12	737
36	0.4398	0.2975	0.8118	0.1457	0.0968	98.7	1280	67.5	1.58	0.0040	649	0	1189	51.5	0.42	0.98	445	35	1471	49.8	2.67	0.0000	818	1384	37.9	0.66	0.67	551
36	0.4370	0.2993	0.7226	0.1473	0.0971	95.6	1254	70.2	1.31	0.0070	606	52	1168	51.9	0.37	1.19	390	53	1432	47.4	1.87	0.0012	762	1342	34.3	0.54	1.26	543
36	0.4477	0.2990	0.7258	0.1464	0.0956	101.7	1269	63.4	1.86	0.0060	625	0	1178	47.3	0.43	1.03	436	34	1447	43.8	3.33	0.0013	779	1363	33.4	0.71	0.73	533
36	0.4352	0.2990	0.8794	0.1478	0.0977	96.0	1258	70.2	1.30	0.0070	617	17	1172	47.9	0.36	1.08	435	48	1435	51.5	1.68	0.0019	799	1347	38.8	0.54	1.00	582
36	0.4348	0.2992	0.8414	0.1463	0.0987	96.9	1261	68.8	1.55	0.0070	629	15	1179	51.2	0.41	1.01	459	41	1447	49.3	2.33	0.0003	801	1362	39.5	0.62	0.69	595
36	0.4665	0.2973	0.7946	0.1475	0.0983	96.4	1299	36.4	8.90	0.0030	648	0	1222	67.0	0.86	0.83	532	3	1515	53.6	96.20	0.0000	936	1447	64.3	2.34	0.05	743
36	0.4600	0.2995	0.7430	0.1461	0.0989	95.9	1281	82.5	4.49	0.0030	658	0	1206	72.1	0.72	0.73	530	11	1484	75.0	24.63	0.0000	881	1415	68.1	1.70	0.11	677
36	0.4600	0.2962	0.7764	0.1467	0.0985	94.2	1311	106.8	6.44	0.0030	662	0	1229	77.1	0.82	0.86	484	4	1532	92.0	36.68	0.0000	955	1461	79.3	1.95	0.09	740
36	0.4638	0.2983	0.7474	0.1469	0.0971	98.5	1287	67.9	4.82	0.0040	668	0	1207	53.4	0.71	0.77	503	9	1493	89.2	31.23	0.0000	927	1420	52.8	1.65	0.12	737
36	0.4398	0.2975	0.8118	0.1457	0.0968	98.7	1280	67.5	1.58	0.0040	649	0	1189	51.5	0.42	0.98	445	35	1471	49.8	2.67	0.0000	818	1384	37.9	0.66	0.67	551
36	0.4370	0.2993	0.7226	0.1473	0.0971	95.6	1254	70.2	1.31	0.0070	606	52	1168	51.9	0.37	1.19	390	53	1432	47.4	1.87	0.0012	762	1342	34.3	0.54	1.26	543
36	0.4477	0.2990	0.7258	0.1464	0.0956	101.7	1269	63.4	1.86	0.0060	625	0	1178	47.3	0.43	1.03	436	34	1447	43.8	3.33	0.0013	779	1363	33.4	0.71	0.73	533
36	0.4352	0.2990	0.8794	0.1478	0.0977	96.0	1258	70.2	1.30	0.0070	617	17	1172	47.9	0.36	1.08	435	48	1435	51.5	1.68	0.0019	799	1347	38.8	0.54	1.00	582

Table S3. Results for the different trained ANNs when comparing their outputs with the data of Table S2. The error is computed by using equation (1) of the manuscript with normalized (cero-to-one) input and output vectors.

Model	N_L	N_P	max error	$\langle$error$\rangle$
ReLu 1 Layer	2	82	0.04780	0.02388
	4	142	0.04412	0.02208
	8	262	0.03871	0.02092
	16	502	0.04064	0.02065
ReLu 2 Layers	2	88	0.04780	0.02388
	4	162	0.04000	0.02187
	8	334	0.04505	0.02075
	16	774	0.06148	0.02107
ReLu 3 Layers	2	94	0.08622	0.03344
	4	182	0.04784	0.02389
	8	406	0.38067	0.02218
	16	1046	0.20710	0.02328
Sigm 1 Layer	2	82	0.04440	0.02205
	4	142	0.03868	0.02081
	8	262	0.03901	0.02077
	16	502	0.03901	0.02077
Sigm 2 Layers	2	88	0.04380	0.02205
	4	162	0.03852	0.02077
	8	334	0.04354	0.02059
	16	774	0.04326	0.02142
Sigm 3 Layers	2	94	0.04380	0.02200
	4	182	0.03850	0.02096
	8	406	0.04280	0.02095
	16	1046	0.05030	0.02596

Table S4. Optimized parameters corresponding to the considered 24 ANNs and their predictions. The first row corresponds to the experimental values [1–7]. Legth units are given in nm, charge in elementary charge unit, angles in degrees, energy in kJ/mol, ρ in kg/m^3, ϵ is dimensionless, μ in cP, P_v in bar, and λ in 10^{-4} N/m.

Model Parameters							70wt%												100wt%									
							293K						373K			398K			293K					373K			423K	
c_2	q_H	σ_{OO}	ϵ_{OO}	d_{OO}	d_{OH}	a_{HOO}	ρ	ϵ	μ	P_v	λ	wt%$_v$	ρ	ϵ	μ	P_v	λ	wt%$_v$	ρ	ϵ	μ	P_v	λ	ρ	ϵ	μ	P_v	λ
experimental →							1288	73.0	1.24	0.007	772	18	1209	73.0	0.42	1.04	549	34	1450	76.0	1.25	0.002	802	1362	64.0	0.48	1.00	599
26.66	0.4417	0.2960	0.7377	0.1460	0.0942	96.1	1294	93.9	2.18	0.0100	677	51	1194	69.3	0.51	1.35	425	45	1484	79.0	2.79	0.0066	763	1394	62.5	0.64	0.97	580
32.82	0.4498	0.2960	0.7505	0.1450	0.0940	94.0	1301	88.3	2.48	0.0060	676	0	1202	66.5	0.53	1.21	396	45	1496	76.4	3.21	0.0006	791	1406	52.7	0.68	1.03	603
25.5	0.4300	0.2988	0.7038	0.1455	0.0990	94.0	1274	107.5	2.77	0.0100	616	10	1188	82.0	0.57	0.95	443	41	1466	111.4	3.80	0.0000	799	1380	76.5	0.73	0.70	556
29.02	0.4300	0.2991	0.7477	0.1455	0.0987	95.2	1268	85.5	2.44	0.0150	620	71	1181	69.5	0.53	1.04	430	37	1453	89.2	3.06	0.0000	755	1365	61.9	0.65	0.87	552
25.79	0.4551	0.2996	0.8014	0.1460	0.0947	95.9	1273	92.3	2.49	0.0050	628	14	1183	76.8	0.54	1.11	477	34	1450	95.8	3.38	0.0000	808	1366	68.9	0.72	0.68	564
28.62	0.4507	0.2987	0.8725	0.1462	0.0942	95.4	1277	101.2	2.02	0.0030	668	12	1183	74.7	0.49	1.07	451	38	1452	83.4	2.34	0.0002	811	1365	67.5	0.62	0.81	580
26.53	0.4389	0.2971	0.7430	0.1480	0.0959	96.0	1280	95.9	2.46	0.0050	633	15	1184	57.7	0.52	1.13	430	41	1463	76.9	3.07	0.0003	756	1375	52.8	0.66	0.88	562
29.4	0.4470	0.2987	0.7903	0.1474	0.0954	96.0	1271	82.7	2.25	0.0030	638	35	1179	67.2	0.52	1.10	445	41	1449	82.0	3.00	0.0006	777	1363	56.6	0.66	0.79	576
26.05	0.4600	0.2997	0.8057	0.1471	0.0981	101.1	1274	77.8	7.11	0.0060	641	0	1199	65.0	0.89	0.79	506	9	1470	58.3	36.25	0.0000	913	1400	69.0	1.70	0.08	700
33.54	0.4439	0.2987	0.8565	0.1475	0.0968	96.1	1273	83.1	2.48	0.0040	636	13	1183	64.4	0.54	0.97	452	30	1452	71.9	3.33	0.0000	846	1368	53.2	0.72	0.58	634
27.46	0.4383	0.2998	0.7286	0.1476	0.0963	97.1	1252	71.2	2.06	0.0090	620	40	1164	55.7	0.47	1.14	431	50	1422	58.7	2.41	0.0008	720	1332	45.2	0.58	1.28	533
27.99	0.4320	0.2995	0.7629	0.1450	0.0989	95.3	1265	80.3	2.45	0.0030	613	21	1181	59.9	0.53	0.97	476	41	1451	70.8	3.23	0.0011	805	1364	51.7	0.68	0.79	594
31.18	0.4445	0.2980	0.8942	0.1462	0.0956	98.0	1280	75.1	2.33	0.0050	675	27	1188	59.8	0.51	1.14	443	33	1460	56.5	2.84	0.0000	849	1374	44.6	0.66	0.73	627
31.91	0.4541	0.2991	0.7000	0.1480	0.0952	94.4	1265	92.5	2.81	0.0050	626	0	1180	64.9	0.57	0.99	448	38	1450	81.4	4.26	0.0004	750	1366	57.9	0.77	0.77	567
36.00	0.4620	0.3000	0.7000	0.1480	0.0946	94.0	1265	99.0	2.96	0.0030	638	0	1179	73.9	0.58	0.95	473	33	1444	85.9	4.97	0.0003	786	1361	67.9	0.81	0.68	573
36.00	0.4615	0.3000	0.7000	0.1480	0.0947	94.0	1262	89.1	2.88	0.0050	632	27	1178	68.3	0.57	0.96	478	41	1443	82.0	4.75	0.0000	761	1360	60.8	0.81	0.75	581
25.50	0.4300	0.3000	0.7333	0.1480	0.0968	102.0	1242	50.0	1.83	0.0050	587	39	1153	42.5	0.44	1.25	438	57	1406	35.0	2.08	0.0015	702	1314	28.1	0.52	1.54	439
29.49	0.4321	0.2962	0.7000	0.1468	0.0978	94.0	1291	84.0	2.93	0.0060	603	30	1194	57.8	0.56	1.11	468	42	1487	68.9	3.95	0.0003	770	1399	46.6	0.74	0.93	545
31.57	0.4398	0.2990	0.7000	0.1450	0.0973	94.1	1268	78.6	2.52	0.0050	612	27	1182	62.2	0.53	1.08	436	45	1458	62.6	3.52	0.0003	758	1370	48.9	0.69	0.92	529
25.6	0.4508	0.2999	0.8550	0.1471	0.0954	94.0	1263	71.0	2.02	0.0440	637	16	1172	62.4	0.48	1.05	444	44	1431	60.3	2.32	0.0003	821	1343	44.6	0.59	0.99	578
32.63	0.4320	0.2984	0.7853	0.1475	0.0985	97.0	1266	68.6	2.47	0.0090	617	72	1177	52.1	0.53	0.99	439	41	1449	50.8	3.31	0.0004	801	1361	39.4	0.67	0.92	559
32.66	0.4568	0.2985	0.7586	0.1456	0.0943	94.5	1281	80.3	2.50	0.0040	632	62	1188	66.7	0.53	1.11	442	41	1464	69.5	3.35	0.0010	779	1378	55.0	0.69	0.91	554
32.81	0.4566	0.2979	0.7299	0.1453	0.0942	94.2	1289	87.2	2.61	0.0070	622	6	1193	62.2	0.54	1.17	433	42	1476	81.2	3.67	0.0000	799	1389	54.6	0.72	0.92	587
27.18	0.4378	0.2967	0.8000	0.1473	0.0960	95.9	1287	88.8	2.10	0.0060	648	62	1188	61.7	0.49	1.28	432	48	1471	68.7	2.43	0.0004	779	1381	47.7	0.60	1.29	539

Table S5. Final best parameters found by performing an MC search around the predictions of the ANNs. The first row corresponds to the experimental values [1–7], the second to the weights considered in equation (1), and the six last rows show the best results. Legth units are given in nm, charge in elementary charge unit, angles in degrees, energy in kJ/mol, ρ in kg/m^3, ϵ is dimensionless, μ in cP, P_v in bar, and λ in 10^{-4} N/m.

Model Parameters							70wt%												100wt%										error
							293K						373K			398K			293K					373K			423K		
c_2	q_H	σ_{OO}	ϵ_{OO}	d_{OO}	d_{OH}	a_{HOO}	ρ	ϵ	μ	P_v	λ	wt%$_v$	ρ	ϵ	μ	P_v	λ	wt%$_v$	ρ	ϵ	μ	P_v	λ	ρ	ϵ	μ	P_v	λ	-
experimental →							1288	73.0	2.20	0.007	772	18	1209	73	0.56	1.04	549	34	1450	76.0	1.79	0.0020	802	1362	64.0	0.54	1.00	599	-
weights →							0.25	0.25	0.125	0.125	0.25	0.125	0.25	0.25	0.125	0.25	0.25	0.25	1	1	0.5	0.25	1	1	1	0.5	1	0.5	-
26.91	0.4323	0.2999	0.8912	0.1463	0.0979	95.8	1262	77.5	1.86	0.0060	639	11	1172	60.3	0.47	1.02	459	46	1430	74.2	2.10	0.0019	838	1341	51.5	0.57	1.06	602	0.143
28.65	0.4509	0.2989	0.8612	0.1459	0.0943	95.6	1275	96.9	2.04	0.0080	658	21	1181	65.9	0.49	1.12	435	38	1449	84.5	2.38	0.0022	809	1361	58.7	0.60	0.94	595	0.148
28.67	0.4493	0.2983	0.8808	0.1461	0.0944	94.7	1280	86.5	2.00	0.0060	685	17	1186	69.0	0.48	1.15	462	41	1456	84.7	2.27	0.0013	834	1368	59.7	0.60	0.85	620	0.157
28.63	0.4504	0.2992	0.8623	0.1459	0.0944	94.8	1272	88.1	1.93	0.0060	669	28	1179	65.6	0.47	1.18	436	42	1443	72.2	2.14	0.0022	801	1355	50.5	0.58	1.11	577	0.175
28.63	0.4519	0.2992	0.8636	0.1457	0.0945	94.6	1274	79.6	1.92	0.0080	640	14	1180	61.5	0.47	1.22	434	49	1447	80.6	2.14	0.0018	870	1358	53.6	0.57	1.14	597	0.181
26.98	0.4314	0.2995	0.8810	0.1468	0.0980	96.1	1262	76.8	1.91	0.0060	629	17	1172	58.6	0.48	1.12	455	44	1431	63.8	2.17	0.0020	832	1342	47.2	0.57	0.95	558	0.182

Table S6. Density, ρ (kg m^{-3}), as a function of the weight percent of H_2O_2 in the mixture and temperature, T. Errors are close to 2 kg m^{-3}.

	T (K)							
wt%	273	283	293	303	313	323	333	343
0	1009	1006	1001	996	991	984	978	970
10	1042	1038	1033	1027	1021	1014	1006	999
20	1078	1072	1066	1060	1053	1045	1037	1029
30	1116	1109	1102	1095	1087	1079	1071	1062
40	1156	1149	1141	1133	1124	1116	1107	1098
60	1244	1235	1226	1217	1208	1198	1189	1179
80	1344	1334	1324	1314	1304	1294	1284	1274
100	1455	1445	1435	1424	1414	1404	1394	1383

Table S7. Thermal expansion coefficient, α_P (10^{-4} K^{-1}), as a function of the weight percent of H_2O_2 in the mixture and temperature, T. Errors are close to 0.02 $\times$ 10^{-4} K^{-1}.

	T (K)							
wt%	273	283	293	303	313	323	333	343
0	2.99	3.84	4.63	5.36	6.03	6.65	7.21	7.71
10	4.00	4.69	5.34	5.94	6.51	7.03	7.51	7.94
20	4.93	5.45	5.96	6.44	6.89	7.33	7.74	8.13
30	5.71	6.11	6.49	6.87	7.24	7.59	7.94	8.27
40	6.28	6.60	6.91	7.21	7.50	7.79	8.06	8.33
60	7.05	7.23	7.41	7.59	7.77	7.95	8.13	8.30
80	7.23	7.34	7.45	7.56	7.68	7.80	7.92	8.04
100	6.93	6.98	7.04	7.12	7.21	7.32	7.45	7.59

Table S8. Adiabatic compressibility, κ_S (10^{-5} atm^{-1}), as a function of the weight percent of H_2O_2 in the mixture and temperature, T. Errors are close to 0.02$\times$$10^{-5}$ atm^{-1}.

	T (K)							
wt%	273	283	293	303	313	323	333	343
0	4.35	4.39	4.43	4.47	4.54	4.58	4.70	4.79
10	4.27	4.30	4.34	4.38	4.47	4.52	4.65	4.76
20	4.17	4.20	4.23	4.28	4.34	4.44	4.57	4.65
30	4.00	4.04	4.06	4.15	4.21	4.27	4.40	4.51
40	3.78	3.83	3.89	3.95	4.03	4.13	4.24	4.36
60	3.26	3.29	3.38	3.52	3.57	3.73	3.82	3.93
80	2.72	2.80	2.87	2.99	3.05	3.17	3.29	3.40
100	2.23	2.30	2.36	2.46	2.53	2.60	2.70	2.79

Table S9. Relative dielectric constant, ϵ, as a function of the weight percent of H_2O_2 in the mixture and temperature, T. Errors are close to 3.

	T (K)							
wt%	273	283	293	303	313	323	333	343
0	72	72	71	71	70	69	69	68
10	74	73	72	71	70	69	68	67
20	76	74	73	71	70	68	68	67
30	77	75	74	72	71	69	67	66
40	77	76	75	72	71	68	67	65
60	79	77	76	73	71	68	66	64
80	80	79	76	73	71	68	65	64
100	83	81	78	75	72	68	65	63

Table S10. Enthalpy of vapor formation, ΔH_{vap} (kJ mol^{-1}), as a function of the weight percent of H_2O_2 in the mixture and temperature, T. Errors are close to 0.4 kJ mol^{-1}.

	T (K)							
wt%	273	283	293	303	313	323	333	343
0	50.0	49.5	48.9	48.4	47.9	47.3	46.8	46.3
10	50.5	50.0	49.4	48.9	48.3	47.8	47.3	46.8
20	51.0	50.5	49.9	49.4	48.9	48.4	47.8	47.3
30	51.7	51.1	50.6	50.0	49.5	48.9	48.4	47.9
40	52.4	51.8	51.2	50.6	50.1	49.5	49.0	48.4
60	54.0	53.5	52.8	52.2	51.6	51.1	50.5	50.0
80	56.0	55.4	54.7	54.1	53.4	52.8	52.2	51.7
100	58.1	57.5	56.7	56.1	55.4	54.8	54.1	53.5

Table S11. Hydrogen peroxide and water diffusion coefficients, D (10^{-9} m^{2}s^{-1}), as a function of the weight percent of H_2O_2 in the mixture and temperature, T. Errors are close to 0.05×10^{-9} m^{2}s^{-1}.

	T (K)															
	H_2O_2								H_2O							
wt%	273	283	293	303	313	323	333	343	273	283	293	303	313	323	333	343
0	-	-	-	-	-	-	-	-	1.433	1.877	2.375	2.915	3.568	4.208	4.866	5.688
10	0.920	1.223	1.564	1.893	2.278	2.713	3.112	3.459	1.395	1.810	2.290	2.815	3.336	3.939	4.662	5.406
20	0.910	1.137	1.493	1.850	2.209	2.607	3.035	3.421	1.329	1.716	2.180	2.640	3.224	3.798	4.488	5.132
30	0.832	1.079	1.416	1.766	2.080	2.470	2.881	3.364	1.219	1.586	2.002	2.553	3.030	3.623	4.267	4.829
40	0.777	1.026	1.292	1.622	1.941	2.338	2.701	3.192	1.142	1.468	1.901	2.355	2.768	3.402	3.967	4.681
60	0.640	0.836	1.112	1.410	1.712	2.070	2.401	2.940	0.917	1.223	1.548	1.948	2.404	2.864	3.337	4.011
80	0.496	0.698	0.934	1.176	1.493	1.789	2.120	2.520	0.674	0.945	1.232	1.653	1.916	2.470	2.876	3.345
100	0.396	0.595	0.769	1.025	1.265	1.551	1.830	2.256	-	-	-	-	-	-	-	-

Table S12. Viscosity, μ (cP), as a function of the weight percent of H_2O_2 in the mixture and temperature, T. Errors are close to 0.01 cP.

	T (K)							
wt%	273	283	293	303	313	323	333	343
0	1.19	0.93	0.76	0.63	0.54	0.46	0.41	0.36
10	1.22	0.96	0.79	0.65	0.56	0.48	0.42	0.37
20	1.27	1.01	0.82	0.69	0.58	0.51	0.44	0.39
30	1.34	1.07	0.87	0.73	0.62	0.54	0.47	0.42
40	1.45	1.14	0.93	0.78	0.66	0.57	0.50	0.44
60	1.74	1.37	1.10	0.92	0.78	0.67	0.58	0.52
80	2.19	1.69	1.35	1.11	0.94	0.80	0.70	0.62
100	2.68	2.06	1.65	1.35	1.14	0.97	0.84	0.74

Table S13. Number of hydrogen bonds, n_h, for H_2O_2 - H_2O_2 (per oxygen atom of H_2O_2 molecules), H_2O - H_2O (per oxygen atom of H_2O molecules), H_2O_2 - H_2O (per oxygen atom of H_2O_2 molecules), and total (per oxygen atom of all molecules) as a function of the weight percent of H_2O_2 in the mixture and temperature, T. Errors are close to 0.01.

	T (K)																															
	H_2O_2 - H_2O_2								H_2O - H_2O								H_2O_2 - H_2O								total							
wt%	273	283	293	303	313	323	333	343	273	283	293	303	313	323	333	343	273	283	293	303	313	323	333	343	273	283	293	303	313	323	333	343
0	0.00	0.00	0.00	0.00	0.00	0.00	0.00	0.00	1.86	1.83	1.81	1.79	1.77	1.75	1.72	1.70	0.00	0.00	0.00	0.00	0.00	0.00	0.00	0.00	1.86	1.83	1.81	1.79	1.77	1.75	1.72	1.70
10	0.06	0.06	0.06	0.06	0.06	0.06	0.06	0.06	1.73	1.71	1.69	1.67	1.65	1.63	1.60	1.58	2.24	2.22	2.20	2.17	2.15	2.12	2.10	2.07	1.79	1.77	1.75	1.73	1.71	1.68	1.66	1.64
20	0.14	0.14	0.14	0.14	0.14	0.13	0.13	0.13	1.60	1.58	1.55	1.53	1.51	1.49	1.47	1.45	2.11	2.09	2.07	2.04	2.02	1.99	1.97	1.94	1.73	1.71	1.69	1.67	1.65	1.63	1.60	1.58
30	0.22	0.22	0.22	0.22	0.22	0.22	0.22	0.22	1.44	1.42	1.40	1.38	1.36	1.35	1.33	1.31	1.96	1.94	1.91	1.89	1.86	1.84	1.82	1.79	1.67	1.65	1.63	1.61	1.59	1.57	1.55	1.53
40	0.33	0.33	0.32	0.32	0.32	0.32	0.31	0.31	1.27	1.25	1.23	1.22	1.20	1.18	1.17	1.15	1.78	1.75	1.73	1.71	1.68	1.66	1.64	1.61	1.62	1.60	1.57	1.55	1.53	1.51	1.49	1.47
60	0.60	0.59	0.59	0.58	0.57	0.57	0.56	0.55	0.88	0.87	0.86	0.84	0.83	0.82	0.81	0.80	1.32	1.30	1.28	1.26	1.24	1.22	1.20	1.19	1.51	1.49	1.47	1.45	1.43	1.42	1.40	1.38
80	0.94	0.93	0.92	0.91	0.90	0.88	0.87	0.86	0.44	0.44	0.43	0.43	0.42	0.42	0.41	0.40	0.71	0.70	0.69	0.68	0.67	0.66	0.65	0.64	1.42	1.40	1.39	1.37	1.35	1.33	1.31	1.29
100	1.35	1.33	1.31	1.29	1.27	1.25	1.23	1.22	0.00	0.00	0.00	0.00	0.00	0.00	0.00	0.00	0.00	0.00	0.00	0.00	0.00	0.00	0.00	0.00	1.35	1.33	1.31	1.29	1.27	1.25	1.23	1.22

Table S14. Liquid and vapor densities, ρ_l and ρ_v (kg m^{-3}, weight percent of H_2O_2 in the liquid and vapor phases, wt$\%_l$ and wt$\%_v$, surface tension, γ (dyn cm^{-1}), and vapor pressure, P_v (bar), as a function of temperature, T, and the total weight percent of H_2O_2 in the simulation cell, wt%.

	wt%																							
	0						10						20						30					
T (K)	ρ_l	ρ_v	wt$\%_l$	wt$\%_v$	γ	P_v	ρ_l	ρ_v	wt$\%_l$	wt$\%_v$	γ	P_v	ρ_l	ρ_v	wt$\%_l$	wt$\%_v$	γ	P_v	ρ_l	ρ_v	wt$\%_l$	wt$\%_v$	γ	P_v
293	999	0.01	0.0	0.0	60.4	0.01	1031	0.01	10.3	1.1	61.5	0.01	1066	0.00	20.6	2.1	62.8	0.01	1102	0.00	30.9	3.3	63.1	0.01
313	988	0.02	0.0	0.0	57.5	0.02	1019	0.02	10.3	1.3	58.4	0.02	1052	0.02	20.6	2.3	59.4	0.02	1087	0.01	30.8	3.7	60.2	0.02
333	975	0.05	0.0	0.0	55.2	0.07	1005	0.04	10.3	1.6	55.5	0.07	1036	0.04	20.5	2.8	55.7	0.06	1070	0.04	30.7	4.5	57.9	0.05
353	960	0.12	0.0	0.0	50.9	0.20	988	0.11	10.2	1.7	51.4	0.18	1019	0.10	20.5	3.5	52.9	0.16	1053	0.10	30.8	6.7	53.4	0.15
373	943	0.25	0.0	0.0	48.1	0.43	971	0.24	10.3	1.9	47.4	0.41	1001	0.23	20.4	4.3	49.5	0.39	1033	0.22	30.7	8.9	50.1	0.36
393	924	0.52	0.0	0.0	44.5	0.95	951	0.51	10.3	2.9	45.2	0.91	981	0.50	20.5	6.8	45.0	0.87	1013	0.45	30.7	10.2	46.3	0.79
413	904	1.0	0.0	0.0	40.6	1.9	930	0.98	10.3	2.3	40.5	1.8	960	0.93	20.5	6.9	41.2	1.7	992	0.86	30.8	11.1	42.7	1.6
433	881	1.8	0.0	0.0	37.1	3.6	908	1.7	10.2	3.4	37.2	3.4	937	1.7	20.6	7.4	38.9	3.2	969	1.6	30.8	12.2	39.4	3.0
453	857	3.0	0.0	0.0	33.7	7.0	883	2.9	10.3	3.8	34.6	6.5	912	2.8	20.6	8.3	35.7	5.7	944	2.7	30.8	13.8	37.4	5.3
473	830	4.9	0.0	0.0	29.1	10.4	857	4.8	10.3	3.9	29.7	9.6	886	4.6	20.6	9.0	30.8	8.9	918	4.4	30.9	15.3	32.2	8.0
493	800	7.8	0.0	0.0	24.5	14.9	828	7.5	10.4	4.4	25.3	14.3	858	7.2	20.7	9.9	26.4	13.1	890	7.0	31.0	16.3	27.2	12.8
513	767	12.1	0.0	0.0	20.8	22.4	796	11.6	10.5	4.9	22.5	21.8	826	11.2	20.8	10.8	23.2	19.9	859	10.7	31.1	17.7	23.2	18.1
533	730	18.5	0.0	0.0	15.7	31.7	759	17.8	10.5	5.4	16.4	30.5	791	17.1	20.9	11.7	17.8	29.4	825	16.2	31.2	18.7	18.6	27.4
553	685	28.2	0.0	0.0	10.9	45.1	719	26.7	10.7	6.1	13.0	43.1	751	25.4	21.2	12.6	14.8	41.6	787	24.2	31.5	20.1	16.2	37.9
573	620	43.7	0.0	0.0	7.8	64.5	670	40.5	10.9	6.5	9.3	60.2	707	38.3	21.5	13.6	10.5	57.4	741	35.9	31.8	21.5	11.6	54.5
593	-	-	-	-	-	-	-	-	-	-	-	-	-	-	-	-	-	-	694	53.5	32.5	23.5	8.0	74.2

Continuation of Table S14.

	wt%																							
	40						60						80						100					
T (K)	ρ_l	ρ_v	wt%$_l$	wt%$_v$	γ	P_v	ρ_l	ρ_v	wt%$_l$	wt%$_v$	γ	P_v	ρ_l	ρ_v	wt%$_l$	wt%$_v$	γ	P_v	ρ_l	ρ_v	wt%$_l$	wt%$_v$	γ	P_v
293	1142	0.00	41.0	5.5	64.7	0.00	1228.60	0.00	61.3	11.7	67.9	0.00	1326.57	0.00	81.2	32.5	76.2	0.00	1430	0.00	100	100	86.3	0.00
313	1125	0.01	41.0	6.8	61.4	0.02	1209.94	0.01	61.3	16.5	64.2	0.01	1306.37	0.01	81.1	37.9	72.9	0.01	1410	0.00	100	100	81.6	0.01
333	1107	0.03	40.9	8.7	58.4	0.05	1190.43	0.03	61.2	22.1	62.6	0.04	1285.62	0.02	81.1	48.9	70.1	0.03	1389	0.02	100	100	77.3	0.02
353	1089	0.09	41.0	11.1	54.4	0.14	1169.97	0.07	61.1	28.2	58.9	0.10	1264.50	0.06	81.0	55.0	66.6	0.07	1369	0.05	100	100	74.8	0.04
373	1069	0.21	40.8	14.6	51.9	0.34	1149.46	0.18	61.1	32.5	55.8	0.26	1242.78	0.15	80.9	60.2	64.6	0.19	1347	0.14	100	100	72.4	0.12
393	1048	0.44	40.9	16.1	47.3	0.74	1127.95	0.39	61.1	33.4	50.0	0.60	1220.75	0.31	80.9	60.4	59.1	0.40	1325	0.29	100	100	68.4	0.28
413	1026	0.83	40.9	18.7	44.3	1.4	1105.57	0.72	61.1	35.1	47.5	1.2	1198.20	0.67	80.9	64.6	54.2	0.88	1303	0.61	100	100	61.5	0.61
433	1003	1.5	40.9	19.8	41.7	2.8	1082.14	1.3	61.0	38.1	43.3	2.2	1174.78	1.2	80.9	65.8	51.4	1.7	1280	1.1	100	100	58.4	1.2
453	979	2.6	40.9	21.0	37.5	4.8	1057.72	2.4	61.1	40.3	40.2	4.0	1150.19	2.2	80.8	67.4	47.7	3.1	1256	2.0	100	100	56.0	2.2
473	953	4.2	41.0	22.8	34.4	7.9	1032.10	3.9	61.1	42.3	38.0	6.9	1124.93	3.6	80.9	68.8	42.6	5.4	1231	3.5	100	100	52.1	4.0
493	925	6.7	41.1	24.1	29.9	11.5	1004.43	6.1	61.1	44.1	33.6	10.4	1097.88	5.7	80.9	69.6	39.6	9.1	1205	5.4	100	100	48.7	7.4
513	895	10.3	41.2	25.5	25.2	16.9	975.65	9.4	61.2	45.1	29.0	15.0	1069.98	8.8	80.9	71.0	33.7	12.9	1177	8.5	100	100	43.5	10.6
533	862	15.4	41.3	27.1	21.2	25.0	944.05	14.2	61.3	46.9	24.5	22.2	1039.15	13.3	80.9	72.2	29.4	18.7	1148	12.8	100	100	38.3	16.0
553	827	22.8	41.6	28.3	17.9	36.3	909.76	21.0	61.5	48.9	21.6	31.8	1006.77	19.1	81.0	72.7	26.2	26.3	1117	18.2	100	100	32.6	22.6
573	785	33.8	41.9	30.2	13.6	51.3	871.08	30.2	61.7	50.2	17.8	45.4	972.04	27.5	81.1	73.8	22.8	37.7	1084	26.0	100	100	27.9	30.7
593	740	49.7	42.7	31.9	10.2	70.0	832.87	42.7	62.1	52.9	13.8	60.9	930.76	39.2	81.4	74.8	17.2	49.4	1047	36.5	100	100	23.6	40.5
618	-	-	-	-	-	-	-	-	-	-	-	-	873.00	58.7	82.4	76.7	11.2	72.1	998	53.3	100	100	19.8	62.3
638	-	-	-	-	-	-	-	-	-	-	-	-	-	-	-	-	-	-	948	74.3	100	100	14.0	83.1

Table S15. Critical temperature, T_c, pressure, P_c, and density, ρ_c, as a function of the total weight percent of H_2O_2 in the simulation cell, wt%. The last column shows the boiling temperature, T_b. Errors are close to 1% for T_c, and 2% for the other quantities.

wt%	T_c (K)	P_c (bar)	ρ_c (kg/m^3)	T_b (K)
0	623	127	292	120
10	640	139	304	124
20	650	144	316	126
30	658	164	326	129
40	666	170	340	132
60	691	182	371	139
80	715	206	404	146
100	732	224	446	156

[1] Easton, M.; Mitchell, A.; Wynne-Jones, W. The behaviour of mixtures of hydrogen peroxide and water. Part 1.—Determination of the densities of mixtures of hydrogen peroxide and water. *Trans. Faraday Soc.* **1952**, *48*, 796–801.

[2] Giguère, P. A.; Geoffrion, P. Changes of density of hydrogen peroxide solutions on cooling and freezing. *Can. J. Res.* **1950**, *28*, 599–607.

[3] Gross Jr, P. M.; Taylor, R. C. The Dielectric Constants of Water, Hydrogen Peroxide and Hydrogen Peroxide—Water Mixtures. *J. Am. Chem. Soc.* **1950**, *72*, 2075–2080.

[4] Phibbs, M.; Giguère, P. A. Hydrogen peroxide and its analogues: I. Density, refractive index, viscosity, and surface tension of deuterium peroxide–deuterium oxide solutions. *Can. J. Chem.* **1951**, *29*, 173–181.

[5] Scatchard, G.; Kavanagh, G. M.; Ticknor, L. B. Vapor-Liquid Equilibrium. VIII. Hydrogen Peroxide—Water Mixtures. *J. Am. Chem. Soc.* **1952**, *74*, 3715–3720.

[6] Giguère, P.; Maass, O. Vapour pressures and boiling points of binary mixtures of hydrogen peroxide and water. *Can. J. Res.* **1940**, *18*, 181–193.

[7] Constantine, M.; Cain, E. *Hydrogen peroxide handbook*; Rocketdyne Canoga Park CA Chemical and Material Sciences Dept, 1967.
